# Perceval sutureless bioprosthesis versus Perimount sutured bioprosthesis for aortic valve replacement in patients with aortic stenosis: a retrospective, propensity-matched study

**DOI:** 10.1186/s13019-024-02575-4

**Published:** 2024-02-14

**Authors:** Sharan J Kapadia, Mohammed Yousuf Salmasi, Alicja Zientara, Isabelle Roussin, Cesare Quarto, George Asimakopoulos

**Affiliations:** 1 Imperial College School of Medicine, Exhibition Rd, South Kensington, London, SW7 2BX UK; 2 Department for Cardiac and Vascular Surgery, University of Freiburg, Hugstetter Strasse 55, 79106 Freiburg, Germany; 3 Department of Cardiology, Lister Hospital East and North Hertfordshire NHS Trust, Coreys Mill Ln, Stevenage, SG1 4AB UK; 4 Department of Cardiothoracic Surgery, Royal Brompton and Harefield NHS Foundation Trust, Sydney Street, London, SW3 6NP UK

**Keywords:** Aortic valve stenosis, Aortic valve implantation, Sutureless aortic valve replacement, Minimally invasive aortic valve replacement

## Abstract

**Background:**

Rapid-deployment aortic valve replacement (RDAVR) is an alternative to conventional AVR (cAVR) for aortic stenosis. Benefits include a reduction in operative times, facilitation of minimal access surgery and superior haemodynamics compared to conventional valves. However, further evidence is required to inform guidelines, preferably in the form of propensity-matched studies that include mid-term follow-up data.

**Methods:**

This was a single-centre, retrospective, propensity-matched cohort study comparing the Perceval and conventional Perimount Magna Ease valve for short- and mid-term clinical parameters and size-matched mid-term echocardiographic parameters (*n* = 102 in both groups) from 2014 to 2020. Data were extracted from a nationally managed dataset.

**Results:**

There were no demographic differences between the matched groups. The Perceval group had shorter cross-clamp time (Perceval 62 [49–81] minutes; Perimount 79 [63–102] minutes, *P* < 0.001), shorter bypass time (Perceval 89 [74–114] minutes; Perimount 104 [84–137] minutes, *P* < 0.001), and more frequent minimally-invasive approaches (Perceval 28%; Perimount 5%, *P* < 0.001). Size-matched haemodynamics showed initially higher gradients in the Perceval group, but haemodynamics equalised at 12 + months. The Perceval group had a more favourable % change in the left ventricular posterior wall dimension at 2 + years (Perceval − 4.8 ± 18; Perimount 17 ± 2).

**Conclusions:**

The Perceval facilitated shorter operations, which may benefit intermediate-high-risk, elderly patients with comorbidities requiring concomitant procedures. It also facilitated minimally invasive surgery. Size-matched haemodynamic performance was similar at mid-term follow-up, with the Perceval possibly better facilitating regression of left ventricular hypertrophy.

**Supplementary Information:**

The online version contains supplementary material available at 10.1186/s13019-024-02575-4.

## Background

Surgical aortic valve replacement (AVR) remains the gold standard treatment for severe aortic stenosis [1]. Within bioprosthetic AVR, rapid-deployment AVR (RDAVR) is an emerging alternative to conventional (sutured) AVR (cAVR). The Perceval (Corcym Canada Corp, Burnaby, BC, Canada) valve is effectively the only truly sutureless valve surgically implanted worldwide [2].

Previous work suggests that Perceval RDAVR reduces cross-clamp and cardiopulmonary bypass (CPB) time compared to cAVR [3], thus enhancing many aspects of recovery [4–11]. The Perceval may enable lower peak and mean pressure gradients (PG and MG) than cAVR, and higher effective orifice area (EOA) and therefore lower risk of patient-prosthesis-mismatch. However, the Perceval may increase pacemaker implantation, post-operative thrombocytopaenia, and para-valvular leakage (PVL) [3, 9, 12, 13]. 

There are no definitive recommendations on RDAVR (Perceval or otherwise) in the European Society of Cardiology or American College of Cardiology/American Heart Association guidelines [14, 15], and conventional AVR remains the gold standard. Although NICE Interventional Procedures Guidance validated sutureless valves as an option in 2018 [7], definitive recommendations for clinical decision-making have not been made. A paucity of mid- to long-term data and robust, matched comparisons is one reason for the lack of definitive guidelines.

This retrospective cohort study aimed to compare Perceval RDAVR with Perimount Magna Ease (Edwards Lifesciences, Irvine, CA, USA) cAVR at a single institution, by analysing intra-operative, clinical post-operative, and short- and mid-term echocardiographic post-operative outcomes in a propensity-matched cohort. Survival was also compared.

## Methods

### Aim

To compare Perceval RDAVR with Perimount Magna Ease cAVR at a single institution, using a propensity-matched cohort.

### Study design

This was a retrospective, propensity-matched cohort study of a prospectively collected, nationally managed database, with data extracted from two hospitals in the United Kingdom. Data collection was carried out in the first half of 2022.

### Inclusion and exclusion criteria

Our institution began implanting the Perceval valve in 2014, with most operations carried out by two experienced surgeons in the department. The number of aortic valve replacements with or without concomitant CABG has been constant in the years from 2014 to 2019, with around 630 cases. There were fewer cases in 2020 due to the COVID pandemic. Patient selection for the Perceval was at the surgeons’ discretion following pre-operative assessment and relied on the presence of favourable anatomy for its implantation. All patients who had undergone AVR with the sutureless Perceval or conventional Perimount Magna Ease between 2014 and 2020 were identified (some patients underwent concomitant CABG and very few underwent non-CABG concomitant procedures; further details are provided in the Results section). Redo cardiac surgery was excluded. Patients with aortic annular enlargement were also excluded, due to the longer duration of the operation and potential altered clinical outcome. Propensity matching was conducted on the resulting cohort of 136 Perceval patients and 296 Perimount patients. After matching, the decision was made to exclude pairs in which either patient had predominantly regurgitation rather than stenosis. The final matched population size was 204, with 102 patients in each group.

### Data collection

An encrypted spreadsheet proforma was used to collect patient data from electronic patient records. Demographic, pre-operative, and short-term post-operative data for all patients, as well as mid-term follow-up (> 6 months post-op) echocardiograms were extracted across two hospital sites. Mid-term follow-up echocardiographic outcomes included mean gradients, peak gradients, indexed effective orifice area, maximum velocity through aortic valve, and ventricular dimensions. Follow-up echocardiograms were organised into three categories: 6–12 months, 1–2 years, and 2 + years (following surgery, the usual departmental protocol is to carry out echocardiographic follow-up on the patient between 3 and 6 months, after 1 year and in a second year). If a patient had more than one echocardiogram per category, the latest was used. Intrahospital mortality and mid-term mortality data were collected for all patients.

### Statistical analysis

The groups were propensity-matched by gender, age, ejection fraction (< 30% = poor, 30–50% = moderate, > 50% = normal), concomitant CABG, and valve size (21, 23, 25, 27 mm) using the ‘nearest neighbour’ method. Details of the propensity matching work conducted by our statistician is provided in Supplement 1. Normality was assessed with the Shapiro-Wilk test. For continuous variables, comparisons were performed using unpaired student’s t-tests (parametric), Mann-Whitney *U* tests (non-parametric unpaired), or Wilcoxon signed-rank tests (non-parametric paired). For categorial variables, comparisons were performed using chi-squared tests if non-binary, and two-sample tests of proportion if binary. For echocardiographic comparisons, sample sizes fell with longer follow-up. Mid-term mortality was compared using a log-rank test and the construction of crude Kaplan-Meier curves for visual comparison. Statistical analysis was conducted using Stata BE 17.0 (Stata Corp., TX, USA). The following variables were also compared using sub-group analysis to separate isolated AVR and concomitant CABG patients: cross-clamp time, cardiopulmonary bypass time, and all post-operative clinical parameters at discharge. Parametric continuous variables are expressed as mean ± standard deviation. Non-parametric continuous variables are expressed as median (lower quartile to upper quartile). Binary and categorical variables are expressed as % in text and % (*n*) in tables.

## Results

### Pre-operative characteristics

In the unmatched comparison, patients who received the sutureless Perceval valve were significantly older (*P* < 0.001) and had a different distribution of valve sizes (*P* = 0.004), but the matched cohort showed no differences in clinical or haemodynamic parameters (Tables 1 and 2).

**Table 1:** Unmatched pre-operative demographic, clinical, and echocardiographic parameters (without regurgitation patients)

	Perimount, *n*=250	Perceval, *n*=132	*P*-value
Age / years	71 (65; 76)	74 (69; 79)	<0.001*
% Female (n)	32% (80)	55% (55)	0.060
BMI / kgm^-2^	28.6 ± 5.2	28.6 ± 5.1	0.990
EuroSCORE II	1.69 (1.1; 2.7)	1.89 (1.3; 2.9)	0.081
Diabetes	26% (66)	23% (31)	0.534
Previous or current smoker	54% (136)	56% (74)	0.756
Previous PCI	9.6% (24)	6.1% (8)	0.235
Hypertension	76% (190)	73% (97)	0.589
‘Good’ EF (>50%)	85% (213)	84% (111)	0.508
‘Moderate’ EF (30-49%)	12% (31)	11% (15)	
Poor EF (<30%)	2.4% (6)	4.6% (6)	
Concomitant CABG / %	42% (106)	39% (51)	0.477
Valve size 21 mm / %	18% (44)	17% (22)	0.004*
Valve size 23 mm / %	46% (115)	33% (43)	
Valve size 25 mm / %	31% (78)	36% (48)	
Valve size 27 mm / %	5.2% (13)	14% (19)	

Values are mean ± SD or median (LQ to UQ), and % (n). * *P* <0.05

BMI: body mass index, PCI: percutaneous coronary intervention, LVEF: left ventricular ejection fraction

**Table 2:** Matched pre-operative demographic, clinical, and echocardiographic parameters

	Perimount, *n*=102		Perceval, *n*=102		*P*-value
Age / years	74.0 ± 6.4		73.7 ± 6.7		0.733
% Female	33.3% (34)		39.2% (40)		0.382
BMI / kgm^-2^	27.2 (24; 30)		28.6 (25; 32)		0.163
EuroSCORE II	1.96 (1.3; 3.3)		1.95 (1.5; 3.0)		0.937
Diabetes	26.5% (27)		24.5% (25)		0.748
Previous or current smoker	50% (51)		55.9% (57)		0.400
Previous PCI	12.7% (13)		6.9% (7)		0.158
Hypertension	78.4% (80)		72.5% (74)		0.329
	**Perimount**	***n***	**Perceval**	***n***	***P-value***
LVEF	60 (55; 65)	100	59.5 (55; 65)	102	0.135
PG / mmHg	72.3 (62; 89)	96	76 (63; 95)	97	0.736
MG / mmHg	41 (35; 52)	94	45 (36; 57)	96	0.393
iEOA / cm^2^	0.42 (0.37; 0.52)	75	0.44 (0.36; 0.51)	84	0.730
Vmax aortic valve / ms^-1^	4.3 (4.0; 4.7)	88	4.4 (3.9; 4.9)	94	0.762
Mixed AS + AR / % (n)	10.8% (11)	102	12.7% (13)	102	0.664
LVOT / cm	2.3 (2.2; 2.5)	86	2.3 (2.1; 2.3)	79	0.311
LVDd / cm	4.9 (4.2; 5.3)	87	5.1 (4.7; 5.4)	86	0.280
LVSd / cm	3.1 ± 0.65	84	3.1 ± 0.67	86	0.734
IVSd / cm	1.4 (1.3; 1.5)	87	1.3 (1.0; 1.4)	85	0.300
LVPWd / cm	1.14 ± 0.28	87	1.18 ± 0.23	86	0.292

Values are mean ± SD or median (LQ to UQ), and % (n)

BMI: body mass index, PCI: percutaneous coronary intervention, LVEF: left ventricular ejection fraction, PG: peak gradient, MG: mean gradient, iEOA: indexed effective orifice area, Vmax: maximum velocity across aortic valve, LVDd: left ventricular diastolic dimension, LVSd: left ventricular systolic dimension, IVSd: interventricular septal diastolic dimension, LVOT: left ventricular outflow tract dimension, LVPWd: left ventricular posterior wall dimension

### Intra-operative parameters

In the matched comparison, Perceval patients had a shorter cross-clamp time (Perceval 62 [50–80] minutes; Perimount 79 [64–105] minutes, *P* < 0.001) and CPB time (Perceval 92 [75–116] minutes; Perimount 104 [86–147] minutes, *P* < 0.001) (Table 3). Minimally invasive approaches, mostly mini-sternotomy, were more common in the Perceval group (Perceval 28%; Perimount 5%, *P* < 0.001) (Table 3).

**Table 3:** Intra-operative parameters

	Perimount, *n=*102	Perceval, *n=*102	*P*-value
XCT time isolated AVR ^†^ / minutes	70.0 (58; 82)	52 (43; 63)	<0.001*
CPB time isolated AVR ^†^ / minutes	91 (79; 103)	82 (67; 96)	0.012*
XCT AVR + CABG / minutes	107 (92; 123)	74 (62; 91)	<0.001*
CPB time AVR + CABG / minutes	147 (118; 167)	110 (80; 133)	<0.001*
Minimally invasive / % (n)	5% (5)	28% (29)	<0.001*
Isolated AVR ^†^ / % (n)	58% (59)	53% (54)	0.481
Concomitant CABG / % (n)	42% (43)	47% (48)	
Valve size 21 mm / % (n)	15.7% (16)	16.7% (17)	0.802
Valve size 23 mm / % (n)	36.3% (37)	38.2% (39)	
Valve size 25 mm / % (n)	39.2% (40)	33.3% (34)	
Valve size 27 mm / % (n)	8.8% (9)	11.8% (12)	

^†^ Some patients received other short, non-CABG procedures in addition to AVR: Perimount: 3 x LAA clip + ablation, 1 x LAA clip, 1 x AMVL decalcification, 1 x septal myotomy; Perceval: 3 x LAA clip, 1 x AMVL decalcification, 1 x LAA clip + ablation, 1 x ablation; note that 1 patient with CABG also received ablation

Values: median (LQ to UQ) or % (n). * *P* <0.05

XCT: cross-clamp time, CPB: cardiopulmonary bypass, AVR: aortic valve replacement, CABG: coronary artery bypass graft, MVR: mitral valve replacement

### Post-operative parameters & follow-up

Clinical parameters were compared between the matched patient groups at discharge (Table 4). Echocardiographic parameters were compared by valve size at four time points: discharge (Table 5), 6–12-month follow-up (Table 6), 1-2-year-follow-up (Table 7), and 2+-year-follow-up (Table 8).

**Table 4:** Post-operative (discharge) parameters

	Perimount, *n*=102	Perceval, *n*=102	*P*-value
Operative mortality	2.0% (2)	2.0% (2)	1.000
Need for hemofiltration or dialysis	1.0% (1)	2.9% (3)	0.313
Pacemaker implantation	7.8% (8)	5.9% (6)	0.580
Bleeding req. transfusion^†^	2% (2)	2.9% (3)	0.667
Transient stroke	2.0% (2)	0% (0)	0.155
Permanent stroke	2.9% (3)	0% (0)	0.081
Return to theatre for bleeding / tamponade	5.9% (6)	3.9% (4)	0.517

Values: % (n)

^†^ for this variable, Perimount *n*=100, Perceval *n*=102

**Table 5:** Echocardiographic parameters by size, in propensity-matched population: discharge

	Size / mm	Perimount	n	Perceval	n	*P*-value
**MG**	21	11.5 (9; 15)	16	17 (12; 20)	17	0.010*
	23	11 (9; 14.0)	36	15 (12; 17)	37	<0.001*
	25	10 (8; 12)	39	12 (10; 15)	32	0.013*
	27	10 (7; 11)	9	13 (10; 16)	12	0.195
**PG**	21	21 (17; 26)	16	30 (24; 37)	17	0.007*
	23	21 (18; 24)	36	27 (23; 33)	37	<0.001*
	25	20 (16; 24)	39	23 (18; 28)	32	0.031*
	27	17 (14; 24)	9	25 (18; 28)	12	0.211
**EF % change**	21	4.3 (-11; 8)	15	-1.7 (-4.8; 6.8)	17	0.948
	23	1.6 (-13; 9)	35	1.6 (-1.7; 9.1)	37	0.489
	25	3.4 (-6.7; 11)	39	1.7 (-6.8; 11)	33	0.730
	27	-3.9 (-8.8; 14)	9	1.7 (-5.7; 13)	12	0.589
**Vmax**	21	2.29 (1.9; 2.5)	15	2.91 (2.5; 3.2)	15	0.005*
	23	2.32 (2.1; 2.5)	34	2.58 (2.2; 2.9)	37	0.010*
	25	2.3 (2; 2.5)	37	2.51 (2.0; 2.6)	32	0.609
	27	2.22 (1.6; 2.4)	8	2.3 (2.0; 2.7)	12	0.374
**iEOA**	21	0.81 (0.78; 0.87)	10	0.76 (0.65; 0.88)	8	0.203
	23	0.83 (0.61; 1.1)	25	0.80 (0.70; 1.0)	30	0.874
	25	0.94 (0.89; 1.1)	25	0.77 (0.64; 0.84)	21	0.002*
	27	0.90 (0.8; 1.2)	7	1.06 (0.70; 1.3)	8	0.908
**% PVL (n)**	21	0	16	0	17	-
	23	0	35	5.1% (2)	39	0.174
	25	7.5 (3)	40	0	34	0.103
	27	0	9	0	12	-

Values are mean ± SD or median (LQ to UQ), and % (n). * *P* <0.05

PG: peak gradient, MG: mean gradient, iEOA: indexed effective orifice area, Vmax: maximum velocity across aortic valve, LVDd: left ventricular diastolic dimension, LVSd: left ventricular systolic dimension, IVSd: interventricular septal diastolic dimension, LVOT: left ventricular outflow tract dimension, LVPWd: left ventricular posterior wall dimension

**Table 6:** Echocardiographic parameters & ventricular dimensions by size, in matched patients: 6-12 months

	Size / mm	Perimount	n	Perceval	n	*P*-value
**MG**	21	7 (7; 10)	3	18 (10; 25)	4	0.114
	23	10 (9; 18)	7	13 (11; 14)	7	0.444
	25	10 (8; 12)	11	9 (7; 13)	9	0.809
	27	6.5 (5; 8)	2	10.8 (10; 12)	2	0.333
**PG**	21	16.3 (13; 19)	3	31 (20.0; 43)	4	0.229
	23	19 (17; 33)	7	26 (20.0; 28)	7	0.330
	25	19.5 (16; 23)	11	18.1 (13; 21)	8	0.642
	27	13.5 (9; 18)	2	20.5 (19; 22)	2	0.333
**LVEF % change**	21	-11 (-16; -4.9)	3	21 (1.7; 41)	2	0.200
	23	1.5 (-5; 10)	7	4.6 (1.6; 5.5)	5	0.755
	25	1.7 (-4.5; 10)	11	-1.4 (-7.5; 36)	8	0.732
	27	16 (14; 18)	2	45 (5; 86)	2	1.000
**Vmax**	21	1.99 ± 0.19	3	2.2 ± 1.1	4	0.801
	23	2.30 ± 0.43	7	2.46 ± 0.24	7	0.393
	25	2.21 ± 0.30	11	2.12 ± 0.34	5	0.547
	27	1.81 ± 0.44	2	2.24 (2.2; 2.3)	2	0.306
**iEOA**	21	0.73 ± 0.17	3	0.5 (0.47; 0.82)	3	0.400
	23	0.78 (0.6; 0.97)	6	0.74 (0.73; 0.82)	3	1.000
	25	0.91 (0.84; 0.94)	8	0.97 (0.91; 0.99)	9	0.500
	27	1.30 (1.1; 1.5)	2	1.01	1	0.667
**% PVL (n)**	21	0	3	0	3	-
	23	0	6	0	6	-
	25	0	11	0	6	-
	27	0	2	0	2	-
LVOT % change	21	-8.3 ± 5.4	3	-10.0 ± 5.3	3	0.703
	23	-4.3 ± 11.5	6	0.83 ± 11	4	0.503
	25	-4.7 ± 9	6	-7.50 ± 6.8	6	0.558
	27	-3.8 ± 5.4	2	-4.5	1	-
**LVDd % change**	21	-6.7 (-28; 4.7)	3	-3.5 (-9; 4.7)	4	0.629
	23	-2.1 (-5; 1.1)	7	-11 (-16; -9.6)	6	0.051
	25	-11 (-17; -6)	8	-3.1 (-9.5; 14)	8	0.038*
	27	-9.6 (-9.9; -9.4)	2	-15.4 (-19; -12)	2	0.333
**LVSd % change**	21	-2.24 ± 18	3	6.0 ± 28	4	0.679
	23	2.55 ± 11	6	1.34 ± 21	6	0.701
	25	-8.6 ± 6.9	7	5.3 ± 25	8	0.167
	27	-9.35 ± 0.29	2	-25 ± 11	2	0.301
**IVSd % change**	21	-19 ± 25	3	-10.1 ± 8.4	4	0.515
	23	-4.3 ± 10	7	-14 ± 27	6	0.450
	25	0.83 ± 15	8	-14 ± 15	8	0.061
	27	-9.4 ± 3	2	-17 ± 16	2	0.549
**LVPWd % change**	21	-29 ± 19	3	-8.8 ± 30	4	0.371
	23	-5.6 ± 14	7	-9.3 ± 15	6	0.660
	25	4.1 ± 23	7	-22.5 ± 20	7	0.040*
	27	-4.3 ± 11	2	-21 ± 11	2	0.266

Values are mean ± SD or median (LQ to UQ), and % (n). * *P* <0.05

PG: peak gradient, MG: mean gradient, iEOA: indexed effective orifice area, Vmax: maximum velocity across aortic valve, LVDd: left ventricular diastolic dimension, LVSd: left ventricular systolic dimension, IVSd: interventricular septal diastolic dimension, LVOT: left ventricular outflow tract dimension, LVPWd: left ventricular posterior wall dimension

**Table 7:** Echocardiographic parameters & ventricular dimensions by size, in matched patients: 1-2 years

	Size / mm	Perimount	n	Perceval	n	*P*-value
**MG**	21	13	1	15 (10; 17)	7	0.750
	23	10 (5; 14)	3	11 (10; 14)	12	0.462
	25	11 (9; 16)	11	11 (9; 11)	8	0.525
	27	7	1	8.3 (6; 11)	6	1.000
**PG**	21	20.6	1	28.1 (19; 31)	7	1.000
	23	17.1 (8; 26)	3	22 (19; 27)	12	0.750
	25	26 (16; 27)	11	19.9 (18; 23)	8	0.365
	27	13	1	15.6 (11; 18)	6	0.298
**LVEF % change**	21	-3.2	1	8.6 (-3.3; 11)	5	1.000
	23	35 (-12; 83)	2	-1.6 (-3; 11)	9	0.873
	25	1.7 (0; 9)	11	0.85 (-4.6; 3.6)	4	0.881
	27	29	1	10.3 (-5.5; 22)	6	0.571
**Vmax**	21	2.27	1	2.33 ± 0.97	7	-
	23	2.01 ± 0.58	3	2.29 ± 0.48	12	0.395
	25	2.34 ± 0.41	11	2.04 ± 0.51	8	0.168
	27	1.8	1	1.83 ± 0.47	6	-
**iEOA**	21	0.85	1	0.70 (0.5; 0.9)	4	-
	23	0.87 (0.85; 0.9)	2	0.69 (0.6; 0.9)	4	0.533
	25	0.81 (0.70; 1.0)	11	0.80 (0.70; 0.9)	2	0.769
	27	1.16	1	1.3 (1.1; 1.4)	4	-
**% PVL (n)**	21	0	1	0	7	-
	23	0	3	0	12	-
	25	0	11	0	8	-
	27	0	1	0	6	-
LVOT % change	21	-	0	-6.2 ± 10	6	-
	23	-1.6 ± 2.7	3	-7.5 ± 8.4	6	0.291
	25	-2.5 ± 15	8	-1.9 ± 10	2	0.962
	27	-13.0	1	-3.4 ± 13	5	-
**LVDd % change**	21	-	0	0.96 ± 10	7	-
	23	1.3 ± 3.4	3	-7.1 ± 14	11	0.339
	25	-4.9 ± 12	8	-8.4 ± 9.3	5	0.584
	27	-19	1	-16.5 ± 18	6	-
**LVSd % change**	21	-	0	9.0 ± 23	7	-
	23	4.1 ± 8.5	2	-11 ± 13	10	0.144
	25	3.1 ± 8	7	15.2 ± 12	5	0.064
	27	-10.8	1	-17.3 ± 16	6	-
**IVSd % change**	21	-	0	-14 ± 12	7	-
	23	-0.21 ± 29	3	-8.3 + 15	11	0.500
	25	-15 ± 11	8	-6.8 ± 14	5	0.298
	27	12.4	1	-7.8 ± 13	6	-
**LVPWd % change**	21	-	0	-8.7 ± 20	7	-
	23	-6.1 ± 18	3	-7.2 ± 18	11	0.923
	25	5.6 ± 26	7	-4.2 ± 27	5	0.464
	27	-19.3	1	4.7 ± 26	6	-

Values are mean ± SD or median (LQ to UQ), and % (n)

PG: peak gradient, MG: mean gradient, iEOA: indexed effective orifice area, Vmax: maximum velocity across aortic valve, LVDd: left ventricular diastolic dimension, LVSd: left ventricular systolic dimension, IVSd: interventricular septal diastolic dimension, LVOT: left ventricular outflow tract dimension, LVPWd: left ventricular posterior wall dimension

**Table 8:** Echocardiographic parameters by size in propensity-matched population: 2+ years

	Size / mm	Perimount	n	Perceval	n	*P*-value
**MG**	21	-	0	18 (17; 19)	6	-
	23	11 (9; 11)	6	12.7 (11; 15)	10	0.362
	25	11 (10; 21)	7	10.3 (7.3; 12)	10	0.351
	27	9	1	7.7 (7; 13)	5	1.000
**PG**	21	-	0	34.9 (30; 43)	6	-
	23	20 (16; 21)	6	24 (20; 27)	10	0.274
	25	20 (18 40)	7	20.4 (14; 23)	10	0.550
	27	17.3	1	14.9 (13; 23)	5	1.000
**EF % change**	21	-	0	3.6 (-1.6; 48)	3	-
	23	-5.6 (-17; 3.3)	6	-1.5 (-19; 15)	5	0.970
	25	6.7 (-18; 12)	7	9.7 (3.3; 20)	8	0.220
	27	26	1	17 (0.85; 41)	4	1.000
**Vmax**	21	-	1	2.9 ± 0.44	6	-
	23	2.34 ± 0.55	6	2.44 ± 0.29	10	0.645
	25	2.32 ± 0.75	7	2.1 ± 0.40	10	0.425
	27	2.08	1	2.0 ± 0.36	5	-
**iEOA**	21	-	0	0.69 ± 0.26	3	-
	23	2.3 ± 0.55	6	2.4 ± 0.29	10	0.726
	25	2.3 ± 0.75	7	2.1 ± 0.40	10	0.762
	27	2.1 ± 0.75	1	2.0 ± 0.36	5	-
**% PVL (n)**	21	-	0	16.7 (1)	6	-
	23	0	5	0	10	-
	25	0	6	10 (1)	10	0.625
	27	0	1	0	5	-
LVOT % change	21	-	0	-10 (-26; 0)	3	-
	23	0.95 (-4.8; 5)	6	0 (0; 5)	3	0.976
	25	-5 (-10; -1.8)	5	-8.7 (-30; 2.4)	4	0.730
	27	-7.7	1	-1.3 (-3.6; 2.3)	4	0.400
**LVDd % change**	21	-	0	-3.1 ± 12	6	-
	23	-4.8 ± 13	6	2.1 ± 8	9	0.237
	25	-11 ± 7.6	5	-2.6 ± 13	7	0.212
	27	-10.5	1	-15 ± 16	4	-
**LVSd % change**	21	-	0	-11.4	5	-
	23	-4.7 ± 19.9	6	5.6 ± 24	9	0.394
	25	-12 ± 8	4	-3.8 ± 33	7	0.378
	27	-19.6	1	-25.1	3	-
**IVSd % change**	21	-	1	-5.8 ± 19	6	-
	23	-8.9 ± 19	6	-12 ± 12	9	0.687
	25	-4.1 ± 22	5	-11 ± 22	7	0.606
	27	-24.6	1	0.59 ± 9.2	4	-
**LVPWd % change**	21	-	0	-8.6 ± 28	6	
	23	-18 ± 22	6	-12.1 ± 19	9	0.613
	25	17 ± 2	4	-4.8 ± 18	7	0.017*
	27	-15.5	1	-12.0 ± 12	4	

Values are mean ± SD or median (LQ to UQ), and % (n). * *P* <0.05

PG: peak gradient, MG: mean gradient, iEOA: indexed effective orifice area, Vmax: maximum velocity across aortic valve, LVDd: left ventricular diastolic dimension, LVSd: left ventricular systolic dimension, IVSd: interventricular septal diastolic dimension, LVOT: left ventricular outflow tract dimension, LVPWd: left ventricular posterior wall dimension

There were no clinical differences between the two groups at discharge (Table 4), including in subgroup analyses by isolated AVR and concomitant CABG.

The Perceval patients had a higher MG and PG in the 21-, 23-, and 25-mm sizes, higher Vmax in the 21- and 23-mm sizes, and a lower iEOA in the 25-mm size at discharge (Table 5). However, discharge % LVEF change, paravalvular regurgitation, and composite regurgitation were comparable (Table 5).

**Fig. 1:**
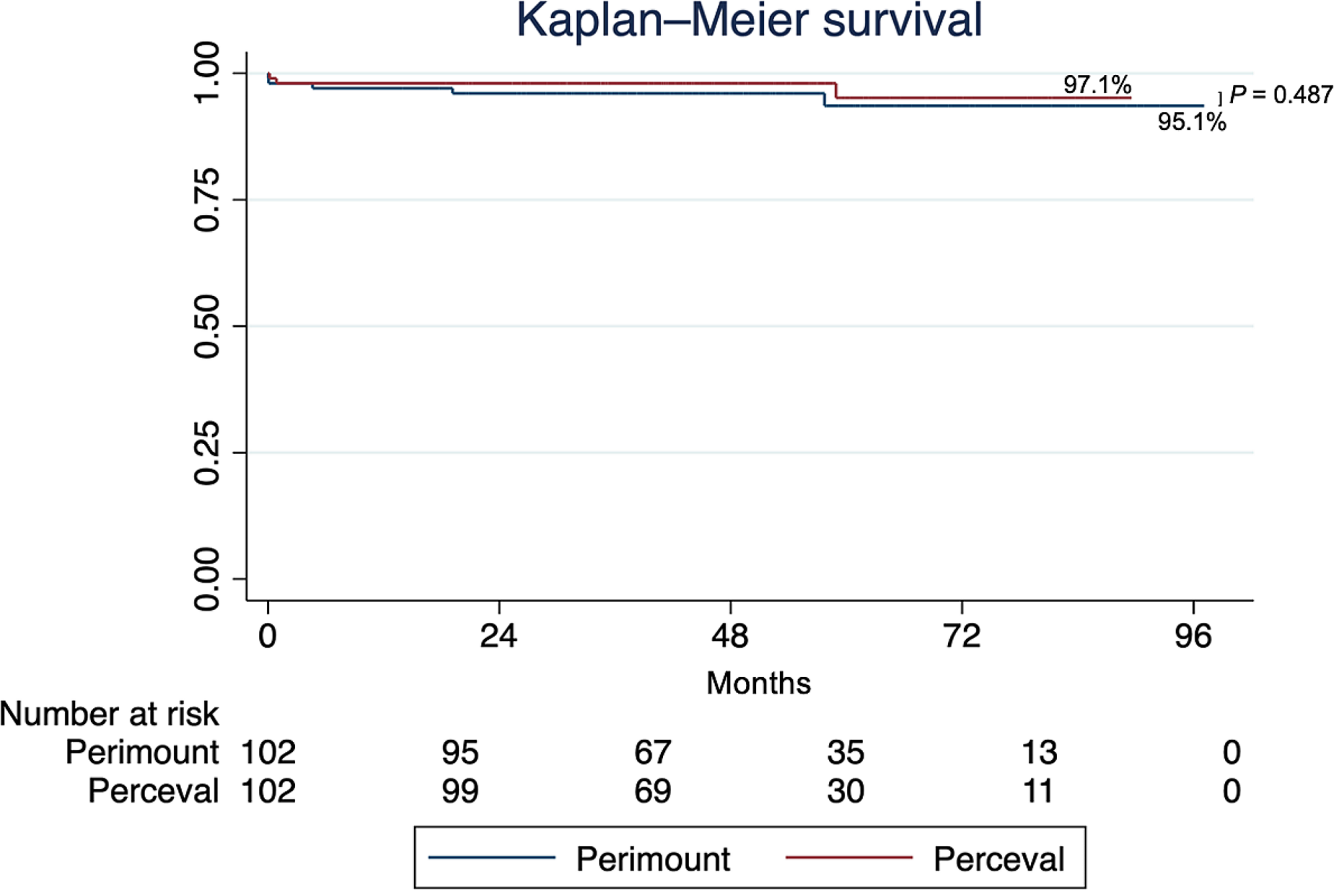
Kaplan-Meier survival estimate. Log-rank *P*=0.487. Median follow-up: 49.3 months (Perimount), 48.4 months (Perceval). Event rates: 5 (Perimount), 3 (Perceval)

At 6–12 months (median follow-up: 266 days [Perceval], 283 days [Perimount]), the Perimount group had a more negative LVDd % change in the 25-mm size, and the LVPWd % change was more negative with in the Perceval group in the 25-mm size; there were no other differences. At 1–2 years (median follow-up: 506 days [Perceval], 595 days [Perimount]), there were no differences. At 2 + years (median follow-up: 959 days [Perceval], 1363 days [Perimount]), the only difference was a more negative LVPWd % change in the Perceval group in the 25-mm size (as seen at 6–12 months). Mid-term mortality did not vary by valve choice (Fig. 1).

## Discussion

### Intra- and post-operative parameters

Cross-clamp and CPB times were shorter in the Perceval group. CPB time may independently influence post-operative morbidity and mortality, likely because CPB places the patient in a non-physiologic state where the blood withstands non-physiologic surfaces and atypical shear forces, causing systemic inflammation [16]. Although the differences in cross-clamp and CPB time were significant, one may argue that clinically this may not produce a large benefit for the cohort of Perceval patients. However, there might be a benefit for the individual, given that older patients with higher comorbidities also receive surgical aortic valve replacement. In combination with the possibility of carrying out the procedure via minimal invasive access without sewing in the valve, this may help the frail patient to get through the procedure with a cumulatively lower risk. Although this study found no clinical differences at discharge parameters, (in-line with some work such as PERSIST-AVR [8]), clinical benefits have been shown elsewhere including in a meta-analysis [6, 9–11]. We studied a matched population, thereby controlling for confounding factors and strengthening the comparison; nevertheless, it is possible that shorter operation times may translate into clinical benefits in larger populations or into benefits not measured in this study. The Perceval may be helpful for higher-risk patients benefitting from shorter operations, such as elderly patients with comorbidities, those with calcified aortas requiring minimal manipulation, and those undergoing concomitant procedures, particularly as operative mortality was comparable between groups.

MI-AVR was more frequent in the Perceval group, likely reflecting facilitation of MI-AVR by sutureless valves. This is consistent with the literature, although MI-AVR frequency varies depending on surgeon preferences [6, 9]. Converging sutureless valves with MI-AVR may further benefit recovery, as is supported by recent meta-analyses [3, 17].

The pacemaker rate was similar between groups, but the rate in both groups was relatively high compared to other studies [9]. Pacing of non-complete heart block arrhythmias may have contributed to this; moreover, our department has a low threshold for ensuring safe long-term outcome from new-onset dysrhythmias. Furthermore, among the patients receiving a permanent pacemaker, 1 Perceval patient had an ablation, and 1 Perimount patient underwent septal myotomy, increasing the pacing risk.

### Echocardiographic parameters

MG, PG, and Vmax were higher for most valve sizes in the Perceval group at discharge, and iEOA was lower for the two smaller valve sizes. This seemingly contrasts with previous work, including a recent meta-analysis [3], the ongoing PERSIST-AVR trial [18], and a recent propensity-matched study [19], which all suggest comparable or superior early hemodynamic performance with the Perceval. Although higher discharge MG with the Perceval has been previously reported [4], we found that haemodynamics equalised between groups beyond 12 months, suggesting limited clinical impact. The reduced early iEOA with the Perceval was unexpected, given its lack of a sewing ring [9]. Another technical explanation for the higher gradients in the two smaller valve sizes may be the effect of a planned slight oversizing leading to impaired leaflet motion due to incompletely deployed valves. In particular, during the early days of Perceval implantation, there was a tendency to oversize with an M-size valve rather than opting for the S-size valve, in an attempt to avoid paravalvular leak. In this study, paravalvular leakage was similar between groups; this contrasts with the 2022 meta-analysis, which showed higher rates of PVL with the Perceval [3], although a different meta-analysis [20] and other studies [4, 13] found no difference.

Overall, a key finding of this study is that haemodynamic performance was comparable using propensity- and size-matched comparisons between the valves at mid-term follow-up, suggesting the Perceval reduces operative times and improves post-operative recovery without a haemodynamic cost. There was a more negative LVPWd % change in the Perceval group, possibly suggesting greater regression of left ventricular hypertrophy. Indeed, at the early stages of Perceval implantation in 2014, beneficial LV remodelling had been noticed in the first year after implantation [21], and this positive effect seems to be confirmed across all sizes after a mean follow up of 5 years reporting continuously low gradients [22]. The main cause of the beneficial remodelling of the LV is unclear. One could speculate that patients benefit from the fact that the sutureless valve has no sewing ring, providing a larger orifice area than the corresponding stented valve; another explanation could be lower long-term gradients with the Perceval; unfortunately, because the cohorts became smaller with time in the present study, potentially beneficial haemodynamics in the long-term may have gone undetected due to low power.

### Limitations

This study faces the standard drawbacks of a retrospective, observational, single-centre design. Another limitation is the declining sample size with time (due to a combination of propensity matching and loss to follow-up). Although the resulting comparisons were fair and less likely to be confounded, they are likely underpowered. Echocardiograms taken / read from 2 different sites were included in this study, possibly introducing inconsistency. Functional outcomes such as symptoms or quality of life were not assessed.

Five staff surgeons perform conventional aortic valve replacement in our department. Out of the five operators, both Perceval implanters are the surgeons with the highest number of isolated AVRs performed and prefer the minimal-invasive access through upper mini-sternotomy. This may explain why, in the cohort of classic Perimount Magna Ease valves, the number of full sternotomies is higher.

## Conclusions

This study supports the operative advantage of the Perceval valve in reducing operative times when compared to conventional sutured valves in a propensity-matched cohort. The Perceval could function as a ‘bridge’ between cAVR and TAVR in intermediate-high-risk, elderly patients with comorbidities or calcified aortic roots undergoing concomitant procedures, who may benefit from shorter operations. Valve haemodynamics, compared between propensity-matched and size-matched groups, were similar at mid-term follow-up, but the Perceval may better facilitate regression of left ventricular hypertrophy. More real-world follow-up data is required to ascertain whether the Perceval delivers superior iEOA and gradients in the long-term.

### Electronic supplementary material

Below is the link to the electronic supplementary material.

Supplementary Material 1

## Data Availability

The datasets used and/or analysed during the current study are available from the corresponding author on reasonable request.

## Abbreviations

AVR: Aortic valve replacement
RDAVR: Rapid-deployment aortic valve replacement
cAVR: Conventional aortic valve replacement
PG: Peak gradient
MG: Mean gradient
iEOA: Indexed effective orifice area
Vmax: Maximum velocity across aortic valve
LVDd: Left ventricular diastolic dimension
LVSd: Left ventricular systolic dimension
IVSd: Interventricular septal diastolic dimension
LVOT: Left ventricular outflow tract dimension
LVPWd: Left ventricular posterior wall dimension
