# Supplementary material for: Perceval sutureless bioprosthesis versus Perimount sutured bioprosthesis for aortic valve replacement in patients with aortic stenosis: a retrospective, propensity-matched study
Source: J Cardiothorac Surg. 2024 Feb 14;19:95. doi: 10.1186/s13019-024-02575-4 (PMC10865583; doi:10.1186/s13019-024-02575-4)
Supplement: Supplementary file 1 — Supplementary Material 1 [file 13019_2024_2575_MOESM1_ESM.docx]

!!help!-> 1276 <-!help!! !!redo!-> "LogisticRegression" AAEAAAD/////AQAAAAAAAAAMAgAAAEJTdGF0c0RpcmVjdCwgVmVyc2lvbj0zLjMuNS4wLCBDdWx0dXJlPW5ldXRyYWwsIFB1YmxpY0tleVRva2VuPW51bGwFAQAAACJTdGF0c0RpcmVjdC5UZW1wbGF0ZXMuUGFyYW1ldGVyQmFnAQAAABBmaWxsZWRQYXJhbWV0ZXJzA/EBU3lzdGVtLkNvbGxlY3Rpb25zLkdlbmVyaWMuRGljdGlvbmFyeWAyW1tTeXN0ZW0uU3RyaW5nLCBtc2NvcmxpYiwgVmVyc2lvbj00LjAuMC4wLCBDdWx0dXJlPW5ldXRyYWwsIFB1YmxpY0tleVRva2VuPWI3N2E1YzU2MTkzNGUwODldLFtTdGF0c0RpcmVjdC5UZW1wbGF0ZXMuRmlsbGVkUGFyYW1ldGVyLCBTdGF0c0RpcmVjdCwgVmVyc2lvbj0zLjMuNS4wLCBDdWx0dXJlPW5ldXRyYWwsIFB1YmxpY0tleVRva2VuPW51bGxdXQIAAAAJAwAAAAQDAAAA8QFTeXN0ZW0uQ29sbGVjdGlvbnMuR2VuZXJpYy5EaWN0aW9uYXJ5YDJbW1N5c3RlbS5TdHJpbmcsIG1zY29ybGliLCBWZXJzaW9uPTQuMC4wLjAsIEN1bHR1cmU9bmV1dHJhbCwgUHVibGljS2V5VG9rZW49Yjc3YTVjNTYxOTM0ZTA4OV0sW1N0YXRzRGlyZWN0LlRlbXBsYXRlcy5GaWxsZWRQYXJhbWV0ZXIsIFN0YXRzRGlyZWN0LCBWZXJzaW9uPTMuMy41LjAsIEN1bHR1cmU9bmV1dHJhbCwgUHVibGljS2V5VG9rZW49bnVsbF1dBAAAAAdWZXJzaW9uCENvbXBhcmVyCEhhc2hTaXplDUtleVZhbHVlUGFpcnMAAwADCJIBU3lzdGVtLkNvbGxlY3Rpb25zLkdlbmVyaWMuR2VuZXJpY0VxdWFsaXR5Q29tcGFyZXJgMVtbU3lzdGVtLlN0cmluZywgbXNjb3JsaWIsIFZlcnNpb249NC4wLjAuMCwgQ3VsdHVyZT1uZXV0cmFsLCBQdWJsaWNLZXlUb2tlbj1iNzdhNWM1NjE5MzRlMDg5XV0I9QFTeXN0ZW0uQ29sbGVjdGlvbnMuR2VuZXJpYy5LZXlWYWx1ZVBhaXJgMltbU3lzdGVtLlN0cmluZywgbXNjb3JsaWIsIFZlcnNpb249NC4wLjAuMCwgQ3VsdHVyZT1uZXV0cmFsLCBQdWJsaWNLZXlUb2tlbj1iNzdhNWM1NjE5MzRlMDg5XSxbU3RhdHNEaXJlY3QuVGVtcGxhdGVzLkZpbGxlZFBhcmFtZXRlciwgU3RhdHNEaXJlY3QsIFZlcnNpb249My4zLjUuMCwgQ3VsdHVyZT1uZXV0cmFsLCBQdWJsaWNLZXlUb2tlbj1udWxsXV1bXQkAAAAJBAAAABEAAAAJBQAAAAQEAAAAkgFTeXN0ZW0uQ29sbGVjdGlvbnMuR2VuZXJpYy5HZW5lcmljRXF1YWxpdHlDb21wYXJlcmAxW1tTeXN0ZW0uU3RyaW5nLCBtc2NvcmxpYiwgVmVyc2lvbj00LjAuMC4wLCBDdWx0dXJlPW5ldXRyYWwsIFB1YmxpY0tleVRva2VuPWI3N2E1YzU2MTkzNGUwODldXQAAAAAHBQAAAAABAAAACQAAAAPzAVN5c3RlbS5Db2xsZWN0aW9ucy5HZW5lcmljLktleVZhbHVlUGFpcmAyW1tTeXN0ZW0uU3RyaW5nLCBtc2NvcmxpYiwgVmVyc2lvbj00LjAuMC4wLCBDdWx0dXJlPW5ldXRyYWwsIFB1YmxpY0tleVRva2VuPWI3N2E1YzU2MTkzNGUwODldLFtTdGF0c0RpcmVjdC5UZW1wbGF0ZXMuRmlsbGVkUGFyYW1ldGVyLCBTdGF0c0RpcmVjdCwgVmVyc2lvbj0zLjMuNS4wLCBDdWx0dXJlPW5ldXRyYWwsIFB1YmxpY0tleVRva2VuPW51bGxdXQT6////8wFTeXN0ZW0uQ29sbGVjdGlvbnMuR2VuZXJpYy5LZXlWYWx1ZVBhaXJgMltbU3lzdGVtLlN0cmluZywgbXNjb3JsaWIsIFZlcnNpb249NC4wLjAuMCwgQ3VsdHVyZT1uZXV0cmFsLCBQdWJsaWNLZXlUb2tlbj1iNzdhNWM1NjE5MzRlMDg5XSxbU3RhdHNEaXJlY3QuVGVtcGxhdGVzLkZpbGxlZFBhcmFtZXRlciwgU3RhdHNEaXJlY3QsIFZlcnNpb249My4zLjUuMCwgQ3VsdHVyZT1uZXV0cmFsLCBQdWJsaWNLZXlUb2tlbj1udWxsXV0CAAAAA2tleQV2YWx1ZQEEK1N0YXRzRGlyZWN0LlRlbXBsYXRlcy5GaWxsZWRPYmplY3RQYXJhbWV0ZXICAAAABgcAAAAHY29udGV4dAkIAAAAAff////6////BgoAAAATY2FuZGlkYXRlUHJlZGljdG9ycwkLAAAAAfT////6////Bg0AAAAKcHJlZGljdG9ycwkOAAAAAfH////6////BhAAAAAIcmVzcG9uc2UJEQAAAAHu////+v///wYTAAAACGdyb3VwaW5nCRQAAAAB6/////r///8GFgAAAAhhY2N1cmFjeQkXAAAAAej////6////BhkAAAAJaW50ZXJjZXB0CRoAAAAB5f////r///8GHAAAAAd3ZWlnaHRzCR0AAAAB4v////r///8GHwAAAAVnYW1tYQkgAAAABQgAAAArU3RhdHNEaXJlY3QuVGVtcGxhdGVzLkZpbGxlZE9iamVjdFBhcmFtZXRlcgIAAAAVPERhdGE+a19fQmFja2luZ0ZpZWxkKkZpbGxlZFBhcmFtZXRlcis8RGlyZWN0aW9uPmtfX0JhY2tpbmdGaWVsZAIELlN0YXRzRGlyZWN0LlRlbXBsYXRlcy5GaWxsZWRQYXJhbWV0ZXJEaXJlY3Rpb24CAAAAAgAAAAkhAAAABd7///8uU3RhdHNEaXJlY3QuVGVtcGxhdGVzLkZpbGxlZFBhcmFtZXRlckRpcmVjdGlvbgEAAAAHdmFsdWVfXwAIAgAAAAEAAAAFCwAAAC5TdGF0c0RpcmVjdC5UZW1wbGF0ZXMuRmlsbGVkRGF0YUZyYW1lUGFyYW1ldGVyAgAAABU8RGF0YT5rX19CYWNraW5nRmllbGQqRmlsbGVkUGFyYW1ldGVyKzxEaXJlY3Rpb24+a19fQmFja2luZ0ZpZWxkBAQaU3RhdHNEaXJlY3QuRGF0YS5EYXRhRnJhbWUCAAAALlN0YXRzRGlyZWN0LlRlbXBsYXRlcy5GaWxsZWRQYXJhbWV0ZXJEaXJlY3Rpb24CAAAAAgAAAAkjAAAAAdz////e////AQAAAAEOAAAACwAAAAklAAAAAdr////e////AQAAAAERAAAACwAAAAknAAAAAdj////e////AQAAAAUUAAAAK1N0YXRzRGlyZWN0LlRlbXBsYXRlcy5GaWxsZWRTdHJpbmdQYXJhbWV0ZXICAAAAFTxEYXRhPmtfX0JhY2tpbmdGaWVsZCpGaWxsZWRQYXJhbWV0ZXIrPERpcmVjdGlvbj5rX19CYWNraW5nRmllbGQBBC5TdGF0c0RpcmVjdC5UZW1wbGF0ZXMuRmlsbGVkUGFyYW1ldGVyRGlyZWN0aW9uAgAAAAIAAAAGKQAAAAppbmRpdmlkdWFsAdb////e////AQAAAAEXAAAAFAAAAAYrAAAACTAuMDAwMDAwMQHU////3v///wEAAAAFGgAAACxTdGF0c0RpcmVjdC5UZW1wbGF0ZXMuRmlsbGVkQm9vbGVhblBhcmFtZXRlcgIAAAAVPERhdGE+a19fQmFja2luZ0ZpZWxkKkZpbGxlZFBhcmFtZXRlcis8RGlyZWN0aW9uPmtfX0JhY2tpbmdGaWVsZAAEAS5TdGF0c0RpcmVjdC5UZW1wbGF0ZXMuRmlsbGVkUGFyYW1ldGVyRGlyZWN0aW9uAgAAAAIAAAABAdP////e////AQAAAAEdAAAAGgAAAAAB0v///97///8BAAAABSAAAAArU3RhdHNEaXJlY3QuVGVtcGxhdGVzLkZpbGxlZERvdWJsZVBhcmFtZXRlcgIAAAAVPERhdGE+a19fQmFja2luZ0ZpZWxkKkZpbGxlZFBhcmFtZXRlcis8RGlyZWN0aW9uPmtfX0JhY2tpbmdGaWVsZAAEBi5TdGF0c0RpcmVjdC5UZW1wbGF0ZXMuRmlsbGVkUGFyYW1ldGVyRGlyZWN0aW9uAgAAAAIAAABmZmZmZmbuPwHR////3v///wEAAAAFIQAAADxTdGF0c0RpcmVjdC5CdWlsdGlucy5SZWdyZXNzK011bHRpcGxlTGluZWFyUmVncmVzc2lvbkNvbnRleHQoAAAAFDxBcmc+a19fQmFja2luZ0ZpZWxkEjxCPmtfX0JhY2tpbmdGaWVsZBs8Q292YXJpYW5jZT5rX19CYWNraW5nRmllbGQUPERFVj5rX19CYWNraW5nRmllbGQVPERFVlg+a19fQmFja2luZ0ZpZWxkEzxERj5rX19CYWNraW5nRmllbGQUPERGWD5rX19CYWNraW5nRmllbGQUPERvQz5rX19CYWNraW5nRmllbGQTPERWPmtfX0JhY2tpbmdGaWVsZBI8TT5rX19CYWNraW5nRmllbGQTPEZWPmtfX0JhY2tpbmdGaWVsZBM8SDE+a19fQmFja2luZ0ZpZWxkEjxIPmtfX0JhY2tpbmdGaWVsZBc8TGFiZWxzPmtfX0JhY2tpbmdGaWVsZBQ8TExYPmtfX0JhY2tpbmdGaWVsZBI8Tj5rX19CYWNraW5nRmllbGQdPE91dGNvbWVUaXRsZT5rX19CYWNraW5nRmllbGQSPFA+a19fQmFja2luZ0ZpZWxkEjxSPmtfX0JhY2tpbmdGaWVsZBM8UjI+a19fQmFja2luZ0ZpZWxkFTxSQU5LPmtfX0JhY2tpbmdGaWVsZBM8UlY+a19fQmFja2luZ0ZpZWxkFDxSWEk+a19fQmFja2luZ0ZpZWxkEjxTPmtfX0JhY2tpbmdGaWVsZBM8U2U+a19fQmFja2luZ0ZpZWxkFjxTU1JFRz5rX19CYWNraW5nRmllbGQUPFNTWT5rX19CYWNraW5nRmllbGQTPFNWPmtfX0JhY2tpbmdGaWVsZBI8VD5rX19CYWNraW5nRmllbGQXPFRpdGxlcz5rX19CYWNraW5nRmllbGQUPFRPTD5rX19CYWNraW5nRmllbGQSPFY+a19fQmFja2luZ0ZpZWxkFDxWSUY+a19fQmFja2luZ0ZpZWxkFTx3YXJuPmtfX0JhY2tpbmdGaWVsZBc8V0VJR0hUPmtfX0JhY2tpbmdGaWVsZBw8d2VpZ2h0VGl0bGU+a19fQmFja2luZ0ZpZWxkEzxXVD5rX19CYWNraW5nRmllbGQSPFg+a19fQmFja2luZ0ZpZWxkEzxYMT5rX19CYWNraW5nRmllbGQSPFk+a19fQmFja2luZ0ZpZWxkBwcHAAAAAAAHAAcHAwYAAAEABwMABwcHBwAABwcGAAMHAQABBwMHBwYGBgYGCAgBBggGBhBTeXN0ZW0uRG91YmxlWyxdBggIBhBTeXN0ZW0uRG91YmxlWyxdCAYIBgYGBgYGBhBTeXN0ZW0uRG91YmxlWyxdBgEGEFN5c3RlbS5Eb3VibGVbLF0GBgIAAAAKCTAAAAAJMQAAAAAAAAAAAAAAKYKFF6g9T0D2////IQAAAAEKCQAAAAkyAAAACTMAAAAKCTQAAAB4LbhwetFwwCIAAAAKCgAAAAk1AAAACgoAAAAKCTYAAAAKCTcAAAAAAAAAAAAAAAAAAAAAAAAACgk4AAAACnsUrkfheoQ/CgoKAQoJOQAAAAk6AAAACgk7AAAABSMAAAAaU3RhdHNEaXJlY3QuRGF0YS5EYXRhRnJhbWUCAAAAFTxOYW1lPmtfX0JhY2tpbmdGaWVsZBo8VmFyaWFibGVzPmtfX0JhY2tpbmdGaWVsZAEDgwFTeXN0ZW0uQ29sbGVjdGlvbnMuR2VuZXJpYy5MaXN0YDFbW1N0YXRzRGlyZWN0LkRhdGEuSVZhcmlhYmxlLCBTdGF0c0RpcmVjdCwgVmVyc2lvbj0zLjMuNS4wLCBDdWx0dXJlPW5ldXRyYWwsIFB1YmxpY0tleVRva2VuPW51bGxdXQIAAAAKCTwAAAABJQAAACMAAAAGPQAAAAZEYXRhIDEJPgAAAAEnAAAAIwAAAAY/AAAABkRhdGEgMQlAAAAADzAAAAALAAAABgAAAAAAAAAAAAAAAAAAAAAAAAAAAAAAAAAAAAAAAAAAAAAAAAAAAAAAAAAAAAAAAAAAAAAAAAAAAAAAAAAAAAAAAAAAAAAAAAAAAAAAAAAAAAAAAAAAAAAPMQAAADgAAAAGAAAAAAAAAAAAAAAAAADwPwAAAAAAAAAAAAAAAAAA8D8AAAAAAAAAAAAAAAAAAAAAAAAAAAAA8D8AAAAAAAAAAAAAAAAAAAAAAAAAAAAAAAAAAAAAAADwPwAAAAAAAAAAAAAAAAAAAAAAAAAAAAAAAAAAAAAAAAAAAAAAAAAA8D8AAAAAAAAAAAAAAAAAAAAAAAAAAAAAAAAAAAAAAAAAAAAAAAAAAAAAAAAAAAAA8D8AAAAAAAAAAAAAAAAAAAAAAAAAAAAAAAAAAAAAAAAAAAAAAAAAAAAAAAAAAAAAAAAAAAAAAADwPwAAAAAAAAAAAAAAAAAAAAAAAAAAAAAAAAAAAAAAAAAAAAAAAAAAAAAAAAAAAAAAAAAAAAAAAAAAAAAAAAAA8D8AAAAAAAAAAAAAAAAAAAAAAAAAAAAAAAAAAAAAAAAAAAAAAAAAAAAAAAAAAAAAAAAAAAAAAAAAAAAAAAAAAAAAAAAAAAAA8D8AAAAAAAAAAAAAAAAAAAAAAAAAAAAAAAAAAAAAAAAAAAAAAAAAAAAAAAAAAAAAAAAAAAAAAAAAAAAAAAAAAAAAAAAAAAAAAAAAAAAAAADwPw8yAAAAIwAAAAYAAAAAAAAAAAAAAAAAAAAAAAAAAAAAAAAAAAAAAAAAAAAAAAAAAAAAAAAAAAAAAAAAAAAAAAAAAAAAAAAAAAAAAAAAAAAAAAAAAAAAAAAAAAAAAAAAAAAAAAAAAAAAAAAAAAAAAAAAAAAAAAAAAAAAAAAAAAAAAAAAAAAAAAAAAAAAAAAAAAAAAAAAAAAAAAAAAAAAAAAAAAAAAAAAAAAAAAAAAAAAAAAAAAAAAAAAAAAAAAAAAAAAAAAAAAAAAAAAAAAAAAAAAAAAAAAAAAAAAAAAAAAAAAAAAAAAAAAAAAAAAAAAAAAAAAAAAAAAAAAAAAAAAAAAAAAAAAAAAAAAAAAAAAAAAAAAAAAAAAAAAAAAAAAAAAAADzMAAAAjAAAABgAAAAAAAAAAAAAAAAAAAAAAAAAAAAAAAAAAAAAAAAAAAAAAAAAAAAAAAAAAAAAAAAAAAAAAAAAAAAAAAAAAAAAAAAAAAAAAAAAAAAAAAAAAAAAAAAAAAAAAAAAAAAAAAAAAAAAAAAAAAAAAAAAAAAAAAAAAAAAAAAAAAAAAAAAAAAAAAAAAAAAAAAAAAAAAAAAAAAAAAAAAAAAAAAAAAAAAAAAAAAAAAAAAAAAAAAAAAAAAAAAAAAAAAAAAAAAAAAAAAAAAAAAAAAAAAAAAAAAAAAAAAAAAAAAAAAAAAAAAAAAAAAAAAAAAAAAAAAAAAAAAAAAAAAAAAAAAAAAAAAAAAAAAAAAAAAAAAAAAAAAAAAAAAAAAAAARNAAAAAsAAAAGQQAAAApWYWx2ZSB0eXBlBkIAAAAWVmFsdmUgb3IgcmluZyBzaXplKDIxKQZDAAAAFlZhbHZlIG9yIHJpbmcgc2l6ZSgyMykGRAAAABZWYWx2ZSBvciByaW5nIHNpemUoMjUpBkUAAAAWVmFsdmUgb3IgcmluZyBzaXplKDI3KQZGAAAAEkNhcmRpYWMgcHJvY2VkdXJlcwZHAAAABUVGKDEpBkgAAAAFRUYoMikGSQAAAAVFRigzKQZKAAAABkdlbmRlcgoPNQAAACMAAAAGAAAAAAAAAAAAAAAAAAAAAAAAAAAAAAAAAAAAAAAAAAAAAAAAAAAAAAAAAAAAAAAAAAAAAAAAAAAAAAAAAAAAAAAAAAAAAAAAAAAAAAAAAAAAAAAAAAAAAAAAAAAAAAAAAAAAAAAAAAAAAAAAAAAAAAAAAAAAAAAAAAAAAAAAAAAAAAAAAAAAAAAAAAAAAAAAAAAAAAAAAAAAAAAAAAAAAAAAAAAAAAAAAAAAAAAAAAAAAAAAAAAAAAAAAAAAAAAAAAAAAAAAAAAAAAAAAAAAAAAAAAAAAAAAAAAAAAAAAAAAAAAAAAAAAAAAAAAAAAAAAAAAAAAAAAAAAAAAAAAAAAAAAAAAAAAAAAAAAAAAAAAAAAAAAAAAAA82AAAAAgAAAAgAAAAAAAAAAA83AAAAIwAAAAYAAAAAAAAAAAAAAAAAAPA/AAAAAAAA8D8AAAAAAADwPwAAAAAAAPA/AAAAAAAA8D8AAAAAAADwPwAAAAAAAPA/AAAAAAAA8D8AAAAAAADwPwAAAAAAAPA/AAAAAAAAAAAAAAAAAAAAAAAAAAAAAAAAAAAAAAAAAAAAAAAAAAAAAAAAAAAAAAAAAAAAAAAAAAAAAAAAAAAAAAAAAAAAAAAAAAAAAAAAAAAAAAAAAAAAAAAAAAAAAAAAAAAAAAAAAAAAAAAAAAAAAAAAAAAAAAAAAAAAAAAAAAAAAAAAAAAAAAAAAAAAAAAAAAAAAAAAAAAAAAAAAAAAAAAAAAAAAAAAAAAAAAAAAAAAAAAAAAAAAAAAAAAAAAAADzgAAAAjAAAABgAAAAAAAAAAAAAAAAAARkAAAAAAAIBFQAAAAAAAAElAAAAAAAAAJEAAAAAAAABHQAAAAAAAADBAAAAAAAAAAEAAAAAAAAAuQAAAAAAAACJAAAAAAAAAEEAAAAAAAAAoQAAAAAAAAERAAAAAAAAACEAAAAAAAIBLQAAAAAAAAABAAAAAAAAALEAAAAAAAAAAQAAAAAAAACJAAAAAAAAA8D8AAAAAAADwPwAAAAAAABxAAAAAAAAANUAAAAAAAADwPwAAAAAAAABAAAAAAAAACEAAAAAAAAAAQAAAAAAAAABAAAAAAAAAGEAAAAAAAAAIQAAAAAAAAABAAAAAAAAA8D8AAAAAAAAAQAAAAAAAAPA/AAAAAAAA8D8POQAAACMAAAAGAAAAAAAAAAAAAAAAAAAAAAAAAAAAAAAAAAAAAAAAAAAAAAAAAAAAAAAAAAAAAAAAAAAAAAAAAAAAAAAAAAAAAAAAAAAAAAAAAAAAAAAAAAAAAAAAAAAAAAAAAAAAAAAAAAAAAAAAAAAAAAAAAAAAAAAAAAAAAAAAAAAAAAAAAAAAAAAAAAAAAAAAAAAAAAAAAAAAAAAAAAAAAAAAAAAAAAAAAAAAAAAAAAAAAAAAAAAAAAAAAAAAAAAAAAAAAAAAAAAAAAAAAAAAAAAAAAAAAAAAAAAAAAAAAAAAAAAAAAAAAAAAAAAAAAAAAAAAAAAAAAAAAAAAAAAAAAAAAAAAAAAAAAAAAAAAAAAAAAAAAAAAAAAAAAAAAAc6AAAAAgIAAAAjAAAACwAAAAAGAAAAAAAAAAAAAAAAAAAAAAAAAAAAAAAAAAAAAAAAAAAAAAAAAAAAAAAAAAAAAAAAAAAAAAAAAAAAAAAAAAAAAAAAAAAAAAAAAAAAAAAAAAAAAAAAAAAAAAAAAAAAAAAAAAAAAAAA8D8AAAAAAAAAAAAAAAAAAAAAAAAAAAAAAAAAAAAAAAAAAAAAAAAAAPA/AAAAAAAAAAAAAAAAAAAAAAAAAAAAAPA/AAAAAAAAAAAAAAAAAAAAAAAAAAAAAAAAAAAAAAAAAAAAAAAAAADwPwAAAAAAAAAAAAAAAAAA8D8AAAAAAADwPwAAAAAAAAAAAAAAAAAAAAAAAAAAAAAAAAAAAAAAAAAAAAAAAAAAAAAAAAAAAAAAAAAAAAAAAPA/AAAAAAAAAAAAAAAAAAAAAAAAAAAAAPA/AAAAAAAA8D8AAAAAAAAAAAAAAAAAAAAAAAAAAAAAAAAAAAAAAAAAAAAAAAAAAAAAAAAAAAAAAAAAAAAAAADwPwAAAAAAAAAAAAAAAAAAAAAAAAAAAADwPwAAAAAAAAAAAAAAAAAA8D8AAAAAAAAAAAAAAAAAAAAAAAAAAAAAAAAAAAAAAAAAAAAAAAAAAAAAAAAAAAAA8D8AAAAAAAAAAAAAAAAAAAAAAAAAAAAAAAAAAAAAAADwPwAAAAAAAAAAAAAAAAAAAAAAAAAAAADwPwAAAAAAAAAAAAAAAAAAAAAAAAAAAAAAAAAAAAAAAPA/AAAAAAAAAAAAAAAAAAAAAAAAAAAAAPA/AAAAAAAA8D8AAAAAAAAAAAAAAAAAAAAAAAAAAAAA8D8AAAAAAAAAAAAAAAAAAAAAAAAAAAAAAAAAAAAAAADwPwAAAAAAAAAAAAAAAAAAAAAAAAAAAADwPwAAAAAAAAAAAAAAAAAA8D8AAAAAAAAAAAAAAAAAAPA/AAAAAAAAAAAAAAAAAAAAAAAAAAAAAPA/AAAAAAAAAAAAAAAAAAAAAAAAAAAAAAAAAAAAAAAA8D8AAAAAAADwPwAAAAAAAAAAAAAAAAAAAAAAAAAAAADwPwAAAAAAAAAAAAAAAAAAAAAAAAAAAAAAAAAAAAAAAPA/AAAAAAAAAAAAAAAAAAAAAAAAAAAAAAAAAAAAAAAAAAAAAAAAAADwPwAAAAAAAAAAAAAAAAAAAAAAAAAAAAAAAAAAAAAAAAAAAAAAAAAA8D8AAAAAAAAAAAAAAAAAAAAAAAAAAAAAAAAAAAAAAAAAAAAAAAAAAPA/AAAAAAAAAAAAAAAAAAAAAAAAAAAAAAAAAAAAAAAAAAAAAAAAAAAAAAAAAAAAAAAAAAAAAAAAAAAAAAAAAADwPwAAAAAAAAAAAAAAAAAA8D8AAAAAAAAAAAAAAAAAAPA/AAAAAAAAAAAAAAAAAAAAAAAAAAAAAAAAAAAAAAAAAAAAAAAAAAAAAAAAAAAAAPA/AAAAAAAAAAAAAAAAAAAAAAAAAAAAAAAAAAAAAAAA8D8AAAAAAAAAAAAAAAAAAAAAAAAAAAAAAAAAAAAAAAAAAAAAAAAAAAAAAAAAAAAAAAAAAAAAAAAAAAAAAAAAAPA/AAAAAAAAAAAAAAAAAADwPwAAAAAAAAAAAAAAAAAAAAAAAAAAAADwPwAAAAAAAAAAAAAAAAAAAAAAAAAAAAAAAAAAAAAAAAAAAAAAAAAAAAAAAAAAAADwPwAAAAAAAAAAAAAAAAAAAAAAAAAAAADwPwAAAAAAAAAAAAAAAAAAAAAAAAAAAAAAAAAAAAAAAAAAAAAAAAAAAAAAAAAAAADwPwAAAAAAAAAAAAAAAAAAAAAAAAAAAAAAAAAAAAAAAPA/AAAAAAAA8D8AAAAAAAAAAAAAAAAAAAAAAAAAAAAAAAAAAAAAAAAAAAAAAAAAAAAAAAAAAAAAAAAAAAAAAAAAAAAAAAAAAPA/AAAAAAAAAAAAAAAAAAAAAAAAAAAAAPA/AAAAAAAAAAAAAAAAAAAAAAAAAAAAAPA/AAAAAAAAAAAAAAAAAAAAAAAAAAAAAAAAAAAAAAAA8D8AAAAAAAAAAAAAAAAAAAAAAAAAAAAAAAAAAAAAAAAAAAAAAAAAAPA/AAAAAAAAAAAAAAAAAADwPwAAAAAAAAAAAAAAAAAAAAAAAAAAAAAAAAAAAAAAAAAAAAAAAAAA8D8AAAAAAAAAAAAAAAAAAAAAAAAAAAAAAAAAAAAAAADwPwAAAAAAAAAAAAAAAAAAAAAAAAAAAAAAAAAAAAAAAAAAAAAAAAAA8D8AAAAAAAAAAAAAAAAAAAAAAAAAAAAAAAAAAAAAAAAAAAAAAAAAAAAAAAAAAAAAAAAAAAAAAADwPwAAAAAAAPA/AAAAAAAAAAAAAAAAAAAAAAAAAAAAAPA/AAAAAAAAAAAAAAAAAAAAAAAAAAAAAAAAAAAAAAAAAAAAAAAAAAAAAAAAAAAAAAAAAAAAAAAA8D8AAAAAAAAAAAAAAAAAAAAAAAAAAAAAAAAAAAAAAAAAAAAAAAAAAAAAAAAAAAAAAAAAAAAAAADwPwAAAAAAAAAAAAAAAAAAAAAAAAAAAADwPwAAAAAAAAAAAAAAAAAAAAAAAAAAAAAAAAAAAAAAAAAAAAAAAAAAAAAAAAAAAAAAAAAAAAAAAAAAAAAAAAAA8D8AAAAAAAAAAAAAAAAAAPA/AAAAAAAAAAAAAAAAAAAAAAAAAAAAAAAAAAAAAAAAAAAAAAAAAAAAAAAAAAAAAAAAAAAAAAAA8D8AAAAAAAAAAAAAAAAAAAAAAAAAAAAAAAAAAAAAAAAAAAAAAAAAAAAAAAAAAAAA8D8AAAAAAAAAAAAAAAAAAAAAAAAAAAAAAAAAAAAAAADwPwAAAAAAAAAAAAAAAAAAAAAAAAAAAAAAAAAAAAAAAPA/AAAAAAAAAAAAAAAAAADwPwAAAAAAAAAAAAAAAAAA8D8AAAAAAAAAAAAAAAAAAAAAAAAAAAAA8D8AAAAAAAAAAAAAAAAAAAAAAAAAAAAAAAAAAAAAAAAAAAAAAAAAAAAAAAAAAAAA8D8AAAAAAAAAAAAAAAAAAPA/AAAAAAAAAAAAAAAAAAAAAAAAAAAAAAAAAAAAAAAAAAAAAAAAAADwPwAAAAAAAAAAAAAAAAAAAAAAAAAAAAAAAAAAAAAAAAAAAAAAAAAA8D8AAAAAAAAAAAAAAAAAAAAAAAAAAAAAAAAAAAAAAAAAAAAAAAAAAAAAAAAAAAAAAAAAAAAAAADwPwAAAAAAAPA/AAAAAAAAAAAAAAAAAAAAAAAAAAAAAPA/AAAAAAAAAAAAAAAAAAAAAAAAAAAAAAAAAAAAAAAAAAAAAAAAAAAAAAAAAAAAAAAAAAAAAAAA8D8AAAAAAADwPwAAAAAAAAAAAAAAAAAA8D8AAAAAAAAAAAAAAAAAAAAAAAAAAAAAAAAAAAAAAAAAAAAAAAAAAAAAAAAAAAAAAAAAAAAAAAAAAAAAAAAAAPA/AAAAAAAAAAAAAAAAAAAAAAAAAAAAAAAAAAAAAAAA8D8AAAAAAAAAAAAAAAAAAAAAAAAAAAAAAAAAAAAAAAAAAAAAAAAAAAAAAAAAAAAA8D8AAAAAAAAAAAAAAAAAAPA/AAAAAAAA8D8AAAAAAAAAAAAAAAAAAAAAAAAAAAAA8D8AAAAAAAAAAAAAAAAAAAAAAAAAAAAA8D8AAAAAAAAAAAAAAAAAAAAAAAAAAAAAAAAAAAAAAAAAAAAAAAAAAAAAAAAAAAAA8D8AAAAAAAAAAAAAAAAAAAAAAAAAAAAAAAAAAAAAAAAAAAAAAAAAAAAAAAAAAAAAAAAAAAAAAAAAAAAAAAAAAPA/AAAAAAAAAAAAAAAAAADwPwAAAAAAAAAAAAAAAAAAAAAAAAAAAADwPwAAAAAAAAAAAAAAAAAAAAAAAAAAAAAAAAAAAAAAAPA/AAAAAAAAAAAAAAAAAAAAAAAAAAAAAPA/AAAAAAAAAAAAAAAAAAAAAAAAAAAAAPA/AAAAAAAA8D8AAAAAAAAAAAAAAAAAAAAAAAAAAAAA8D8AAAAAAAAAAAAAAAAAAAAAAAAAAAAAAAAAAAAAAADwPwAAAAAAAAAAAAAAAAAA8D8AAAAAAAAAAAAAAAAAAAAAAAAAAAAAAAAPOwAAACMAAAAGAAAAAAAAAAAAAAAAAAAoQAAAAAAAAC5AAAAAAAAAIkAAAAAAAAAAQAAAAAAAAC5AAAAAAAAAIEAAAAAAAACgPAAAAAAAABRAAAAAAAAAoDwAAAAAAAAIQAAAAAAAABBAAAAAAAAAIkAAAAAAAADwPwAAAAAAAChAAAAAAAAAoDwAAAAAAAAkQAAAAAAAAKA8AAAAAAAAEEAAAAAAAACgPAAAAAAAAKA8AAAAAAAAAEAAAAAAAAAkQAAAAAAAAKA8AAAAAAAAoDwAAAAAAADwPwAAAAAAAPA/AAAAAAAA8D8AAAAAAAAIQAAAAAAAAABAAAAAAAAAAED////////vPwAAAAAAAABA////////7z/////////vPwQ8AAAAgwFTeXN0ZW0uQ29sbGVjdGlvbnMuR2VuZXJpYy5MaXN0YDFbW1N0YXRzRGlyZWN0LkRhdGEuSVZhcmlhYmxlLCBTdGF0c0RpcmVjdCwgVmVyc2lvbj0zLjMuNS4wLCBDdWx0dXJlPW5ldXRyYWwsIFB1YmxpY0tleVRva2VuPW51bGxdXQMAAAAGX2l0ZW1zBV9zaXplCF92ZXJzaW9uBAAAHFN0YXRzRGlyZWN0LkRhdGEuSVZhcmlhYmxlW10CAAAACAgJSwAAAAMAAAADAAAAAT4AAAA8AAAACUwAAAAJAAAACQAAAAFAAAAAPAAAAAlNAAAAAQAAAAEAAAAHSwAAAAABAAAABAAAAAQaU3RhdHNEaXJlY3QuRGF0YS5JVmFyaWFibGUCAAAACU4AAAAJTwAAAAlQAAAACgdMAAAAAAEAAAAQAAAABBpTdGF0c0RpcmVjdC5EYXRhLklWYXJpYWJsZQIAAAAJUQAAAAlSAAAACVMAAAAJVAAAAAlVAAAACVYAAAAJVwAAAAlYAAAACVkAAAANBwdNAAAAAAEAAAAEAAAABBpTdGF0c0RpcmVjdC5EYXRhLklWYXJpYWJsZQIAAAAJWgAAAA0DBU4AAAAfU3RhdHNEaXJlY3QuRGF0YS5TdHJpbmdWYXJpYWJsZQMAAAAWR2VuZXJpY1ZhcmlhYmxlYDErZGF0YShHZW5lcmljVmFyaWFibGVgMSs8VGl0bGU+a19fQmFja2luZ0ZpZWxkKUdlbmVyaWNWYXJpYWJsZWAxKzxPcmlnaW4+a19fQmFja2luZ0ZpZWxkBgEEGFN0YXRzRGlyZWN0LkRhdGEuSU9yaWdpbgIAAAACAAAACVsAAAAGXAAAAAROYW1lCgFPAAAATgAAAAldAAAABl4AAAAFVmFsdWUKBVAAAAAfU3RhdHNEaXJlY3QuRGF0YS5Eb3VibGVWYXJpYWJsZQcAAAADc3VtA21pbgNtYXgMaGFzU3VtbWFyaWVzFkdlbmVyaWNWYXJpYWJsZWAxK2RhdGEoR2VuZXJpY1ZhcmlhYmxlYDErPFRpdGxlPmtfX0JhY2tpbmdGaWVsZClHZW5lcmljVmFyaWFibGVgMSs8T3JpZ2luPmtfX0JhY2tpbmdGaWVsZAAAAAAHAQQGBgYBBhhTdGF0c0RpcmVjdC5EYXRhLklPcmlnaW4CAAAAAgAAAAAAAAAAAAAAAAAAAAAAAAAAAAAAAAAAAAAJXwAAAAZgAAAACU9sZCB2YWx1ZQoBUQAAAFAAAAAAAAAAAAAAAAAAAAAAAAAAAAAAAAAAAAAACWEAAAAJQgAAAAoBUgAAAFAAAAAAAAAAAAAAAAAAAAAAAAAAAAAAAAAAAAAACWMAAAAJQwAAAAoBUwAAAFAAAAAAAAAAAAAAAAAAAAAAAAAAAAAAAAAAAAAACWUAAAAJRAAAAAoBVAAAAFAAAAAAAAAAAAAAAAAAAAAAAAAAAAAAAAAAAAAACWcAAAAJRQAAAAoBVQAAAFAAAAAAAAAAAAAAAAAAAAAAAAAAAAAAAAAAAAAACglGAAAACWoAAAABVgAAAFAAAAAAAAAAAAAAAAAAAAAAAAAAAAAAAAAAAAAACWsAAAAJRwAAAAoBVwAAAFAAAAAAAAAAAAAAAAAAAAAAAAAAAAAAAAAAAAAACW0AAAAJSAAAAAoBWAAAAFAAAAAAAAAAAAAAAAAAAAAAAAAAAAAAAAAAAAAACW8AAAAJSQAAAAoBWQAAAFAAAAAAAAAAAAAAAAAAAAAAAAAAAAAAAAAAAAAACglKAAAACXIAAAABWgAAAFAAAAAAAAAAAAAAAAAAAAAAAAAAAAAAAAAAAAAACglBAAAACXQAAAARWwAAAAkAAAAJQgAAAAlDAAAACUQAAAAJRQAAAAlGAAAACUcAAAAJSAAAAAlJAAAACUoAAAARXQAAAAkAAAAGfgAAAAMwLDIGfwAAAAMwLDIGgAAAAAMwLDIGgQAAAAMwLDIGggAAAAMwLDUGgwAAAAQwLDI1BoQAAAAEMCwyNQaFAAAABDAsMjUGhgAAAAMwLDUPXwAAAAkAAAAGmpmZmZmZyT+amZmZmZnJP5qZmZmZmck/mpmZmZmZyT8AAAAAAADgPwAAAAAAANA/AAAAAAAA0D8AAAAAAADQPwAAAAAAAOA/D2EAAACwAQAABgAAAAAAAPA/AAAAAAAA8D8AAAAAAAAAAAAAAAAAAAAAAAAAAAAAAAAAAAAAAAAAAAAAAAAAAAAAAAAAAAAAAAAAAAAAAAAAAAAAAAAAAAAAAAAAAAAAAAAAAAAAAADwPwAAAAAAAAAAAAAAAAAAAAAAAAAAAADwPwAAAAAAAAAAAAAAAAAAAAAAAAAAAAAAAAAAAAAAAAAAAAAAAAAAAAAAAAAAAADwPwAAAAAAAPA/AAAAAAAAAAAAAAAAAAAAAAAAAAAAAAAAAAAAAAAAAAAAAAAAAADwPwAAAAAAAAAAAAAAAAAAAAAAAAAAAAAAAAAAAAAAAAAAAAAAAAAAAAAAAAAAAAAAAAAAAAAAAAAAAAAAAAAAAAAAAAAAAADwPwAAAAAAAAAAAAAAAAAA8D8AAAAAAAAAAAAAAAAAAPA/AAAAAAAAAAAAAAAAAAAAAAAAAAAAAAAAAAAAAAAAAAAAAAAAAAAAAAAAAAAAAAAAAAAAAAAAAAAAAAAAAAAAAAAAAAAAAAAAAAAAAAAAAAAAAAAAAADwPwAAAAAAAAAAAAAAAAAA8D8AAAAAAAAAAAAAAAAAAAAAAAAAAAAAAAAAAAAAAADwPwAAAAAAAAAAAAAAAAAA8D8AAAAAAAAAAAAAAAAAAAAAAAAAAAAAAAAAAAAAAAAAAAAAAAAAAPA/AAAAAAAAAAAAAAAAAAAAAAAAAAAAAAAAAAAAAAAAAAAAAAAAAAAAAAAAAAAAAAAAAAAAAAAA8D8AAAAAAAAAAAAAAAAAAPA/AAAAAAAAAAAAAAAAAAAAAAAAAAAAAAAAAAAAAAAAAAAAAAAAAAAAAAAAAAAAAPA/AAAAAAAAAAAAAAAAAAAAAAAAAAAAAPA/AAAAAAAAAAAAAAAAAAAAAAAAAAAAAAAAAAAAAAAAAAAAAAAAAAAAAAAAAAAAAAAAAAAAAAAAAAAAAAAAAADwPwAAAAAAAAAAAAAAAAAAAAAAAAAAAAAAAAAAAAAAAPA/AAAAAAAAAAAAAAAAAAAAAAAAAAAAAAAAAAAAAAAAAAAAAAAAAAAAAAAAAAAAAAAAAAAAAAAAAAAAAAAAAAAAAAAAAAAAAAAAAAAAAAAAAAAAAAAAAAAAAAAAAAAAAAAAAAAAAAAAAAAAAAAAAAAAAAAAAAAAAPA/AAAAAAAAAAAAAAAAAAAAAAAAAAAAAAAAAAAAAAAAAAAAAAAAAAAAAAAAAAAAAAAAAAAAAAAAAAAAAAAAAAAAAAAAAAAAAAAAAAAAAAAAAAAAAAAAAAAAAAAAAAAAAAAAAAAAAAAAAAAAAAAAAAAAAAAAAAAAAPA/AAAAAAAAAAAAAAAAAAAAAAAAAAAAAAAAAAAAAAAAAAAAAAAAAAAAAAAAAAAAAAAAAAAAAAAAAAAAAAAAAADwPwAAAAAAAAAAAAAAAAAAAAAAAAAAAAAAAAAAAAAAAAAAAAAAAAAAAAAAAAAAAAAAAAAAAAAAAAAAAAAAAAAAAAAAAAAAAAAAAAAAAAAAAAAAAAAAAAAAAAAAAAAAAAAAAAAAAAAAAAAAAAAAAAAA8D8AAAAAAAAAAAAAAAAAAPA/AAAAAAAAAAAAAAAAAADwPwAAAAAAAPA/AAAAAAAAAAAAAAAAAADwPwAAAAAAAPA/AAAAAAAAAAAAAAAAAAAAAAAAAAAAAAAAAAAAAAAAAAAAAAAAAAAAAAAAAAAAAAAAAAAAAAAA8D8AAAAAAAAAAAAAAAAAAAAAAAAAAAAAAAAAAAAAAADwPwAAAAAAAPA/AAAAAAAAAAAAAAAAAAAAAAAAAAAAAAAAAAAAAAAAAAAAAAAAAAAAAAAAAAAAAPA/AAAAAAAAAAAAAAAAAAAAAAAAAAAAAAAAAAAAAAAAAAAAAAAAAADwPwAAAAAAAAAAAAAAAAAAAAAAAAAAAAAAAAAAAAAAAAAAAAAAAAAAAAAAAAAAAADwPwAAAAAAAAAAAAAAAAAAAAAAAAAAAAAAAAAAAAAAAAAAAAAAAAAAAAAAAAAAAAAAAAAAAAAAAAAAAAAAAAAAAAAAAAAAAAAAAAAAAAAAAAAAAAAAAAAAAAAAAAAAAAAAAAAAAAAAAAAAAAAAAAAAAAAAAAAAAAAAAAAAAAAAAAAAAAAAAAAAAAAAAAAAAAAAAAAAAAAAAAAAAAAAAAAAAAAAAAAAAAAAAAAAAAAAAAAAAAAAAAAAAAAAAAAAAAAAAAAAAAAAAAAAAAAAAAAA8D8AAAAAAAAAAAAAAAAAAPA/AAAAAAAAAAAAAAAAAAAAAAAAAAAAAAAAAAAAAAAAAAAAAAAAAAAAAAAAAAAAAAAAAAAAAAAAAAAAAAAAAAAAAAAAAAAAAPA/AAAAAAAAAAAAAAAAAAAAAAAAAAAAAPA/AAAAAAAAAAAAAAAAAAAAAAAAAAAAAAAAAAAAAAAAAAAAAAAAAAAAAAAAAAAAAPA/AAAAAAAAAAAAAAAAAAAAAAAAAAAAAAAAAAAAAAAAAAAAAAAAAAAAAAAAAAAAAAAAAAAAAAAAAAAAAAAAAAAAAAAAAAAAAAAAAAAAAAAAAAAAAAAAAAAAAAAAAAAAAAAAAAAAAAAAAAAAAAAAAADwPwAAAAAAAAAAAAAAAAAAAAAAAAAAAAAAAAAAAAAAAPA/AAAAAAAAAAAAAAAAAADwPwAAAAAAAAAAAAAAAAAAAAAAAAAAAADwPwAAAAAAAAAAAAAAAAAAAAAAAAAAAAAAAAAAAAAAAAAAAAAAAAAAAAAAAAAAAAAAAAAAAAAAAPA/AAAAAAAAAAAAAAAAAADwPwAAAAAAAAAAAAAAAAAAAAAAAAAAAAAAAAAAAAAAAAAAAAAAAAAAAAAAAAAAAAAAAAAAAAAAAAAAAAAAAAAAAAAAAAAAAADwPwAAAAAAAAAAAAAAAAAAAAAAAAAAAAAAAAAAAAAAAAAAAAAAAAAAAAAAAAAAAAAAAAAAAAAAAAAAAAAAAAAA8D8AAAAAAADwPwAAAAAAAAAAAAAAAAAAAAAAAAAAAAAAAAAAAAAAAAAAAAAAAAAAAAAAAAAAAAAAAAAAAAAAAAAAAAAAAAAAAAAAAAAAAADwPwAAAAAAAAAAAAAAAAAAAAAAAAAAAAAAAAAAAAAAAAAAAAAAAAAAAAAAAAAAAAAAAAAAAAAAAAAAAAAAAAAAAAAAAAAAAADwPwAAAAAAAAAAAAAAAAAA8D8AAAAAAAAAAAAAAAAAAAAAAAAAAAAAAAAAAAAAAAAAAAAAAAAAAAAAAAAAAAAAAAAAAAAAAAAAAAAAAAAAAAAAAAAAAAAAAAAAAAAAAAAAAAAAAAAAAAAAAAAAAAAAAAAAAAAAAADwPwAAAAAAAAAAAAAAAAAAAAAAAAAAAAAAAAAAAAAAAAAAAAAAAAAA8D8AAAAAAAAAAAAAAAAAAAAAAAAAAAAAAAAAAAAAAAAAAAAAAAAAAAAAAAAAAAAAAAAAAAAAAADwPwAAAAAAAAAAAAAAAAAAAAAAAAAAAAAAAAAAAAAAAAAAAAAAAAAA8D8AAAAAAAAAAAAAAAAAAAAAAAAAAAAAAAAAAAAAAAAAAAAAAAAAAAAAAAAAAAAAAAAAAAAAAAAAAAAAAAAAAAAAAAAAAAAAAAAAAAAAAAAAAAAAAAAAAAAAAAAAAAAAAAAAAAAAAAAAAAAAAAAAAAAAAAAAAAAAAAAAAAAAAAAAAAAAAAAAAAAAAAAAAAAAAAAAAAAAAAAAAAAAAAAAAAAAAAAAAAAAAAAAAAAAAAAAAAAAAAAAAAAAAAAAAAAAAAAAAAAAAAAAAAAAAAAAAAAAAAAAAAAAAAAAAAAAAAAAAAAAAAAAAAAAAAAAAAAAAAAAAAAAAAAAAAAAAAAAAAAAAAAAAAAAAAAAAAAAAAAAAAAAAAAAAAAAAAAAAAAAAAAAAAAAAADwPwAAAAAAAAAAAAAAAAAA8D8AAAAAAAAAAAAAAAAAAAAAAAAAAAAAAAAAAAAAAAAAAAAAAAAAAAAAAAAAAAAAAAAAAAAAAAAAAAAAAAAAAAAAAAAAAAAAAAAAAAAAAAAAAAAAAAAAAAAAAAAAAAAAAAAAAAAAAAAAAAAAAAAAAAAAAAAAAAAAAAAAAAAAAADwPwAAAAAAAAAAAAAAAAAAAAAAAAAAAAAAAAAAAAAAAAAAAAAAAAAAAAAAAAAAAAAAAAAAAAAAAAAAAAAAAAAA8D8AAAAAAAAAAAAAAAAAAAAAAAAAAAAAAAAAAAAAAAAAAAAAAAAAAPA/AAAAAAAAAAAAAAAAAAAAAAAAAAAAAAAAAAAAAAAAAAAAAAAAAADwPwAAAAAAAPA/AAAAAAAAAAAAAAAAAADwPwAAAAAAAAAAAAAAAAAAAAAAAAAAAADwPwAAAAAAAAAAAAAAAAAAAAAAAAAAAAAAAAAAAAAAAAAAAAAAAAAAAAAAAAAAAADwPwAAAAAAAAAAAAAAAAAAAAAAAAAAAADwPwAAAAAAAAAAAAAAAAAAAAAAAAAAAAAAAAAAAAAAAPA/AAAAAAAAAAAAAAAAAAAAAAAAAAAAAPA/AAAAAAAA8D8AAAAAAAAAAAAAAAAAAPA/AAAAAAAA8D8AAAAAAAAAAAAAAAAAAAAAAAAAAAAAAAAAAAAAAADwPw9jAAAAsAEAAAYAAAAAAAAAAAAAAAAAAAAAAAAAAAAAAAAAAAAAAAAAAAAAAAAAAPA/AAAAAAAA8D8AAAAAAADwPwAAAAAAAAAAAAAAAAAA8D8AAAAAAADwPwAAAAAAAPA/AAAAAAAAAAAAAAAAAADwPwAAAAAAAPA/AAAAAAAAAAAAAAAAAADwPwAAAAAAAAAAAAAAAAAA8D8AAAAAAADwPwAAAAAAAPA/AAAAAAAAAAAAAAAAAAAAAAAAAAAAAPA/AAAAAAAA8D8AAAAAAADwPwAAAAAAAPA/AAAAAAAAAAAAAAAAAADwPwAAAAAAAPA/AAAAAAAA8D8AAAAAAADwPwAAAAAAAPA/AAAAAAAAAAAAAAAAAADwPwAAAAAAAPA/AAAAAAAAAAAAAAAAAADwPwAAAAAAAAAAAAAAAAAA8D8AAAAAAAAAAAAAAAAAAPA/AAAAAAAAAAAAAAAAAADwPwAAAAAAAPA/AAAAAAAA8D8AAAAAAADwPwAAAAAAAPA/AAAAAAAA8D8AAAAAAAAAAAAAAAAAAPA/AAAAAAAAAAAAAAAAAAAAAAAAAAAAAAAAAAAAAAAAAAAAAAAAAADwPwAAAAAAAPA/AAAAAAAAAAAAAAAAAADwPwAAAAAAAAAAAAAAAAAAAAAAAAAAAAAAAAAAAAAAAPA/AAAAAAAA8D8AAAAAAAAAAAAAAAAAAPA/AAAAAAAAAAAAAAAAAADwPwAAAAAAAAAAAAAAAAAAAAAAAAAAAADwPwAAAAAAAAAAAAAAAAAAAAAAAAAAAAAAAAAAAAAAAPA/AAAAAAAAAAAAAAAAAAAAAAAAAAAAAAAAAAAAAAAA8D8AAAAAAAAAAAAAAAAAAAAAAAAAAAAAAAAAAAAAAAAAAAAAAAAAAPA/AAAAAAAA8D8AAAAAAAAAAAAAAAAAAAAAAAAAAAAAAAAAAAAAAADwPwAAAAAAAPA/AAAAAAAAAAAAAAAAAADwPwAAAAAAAPA/AAAAAAAA8D8AAAAAAAAAAAAAAAAAAAAAAAAAAAAA8D8AAAAAAAAAAAAAAAAAAPA/AAAAAAAAAAAAAAAAAADwPwAAAAAAAAAAAAAAAAAAAAAAAAAAAAAAAAAAAAAAAAAAAAAAAAAA8D8AAAAAAADwPwAAAAAAAPA/AAAAAAAAAAAAAAAAAAAAAAAAAAAAAAAAAAAAAAAAAAAAAAAAAAAAAAAAAAAAAPA/AAAAAAAA8D8AAAAAAADwPwAAAAAAAAAAAAAAAAAAAAAAAAAAAAAAAAAAAAAAAAAAAAAAAAAA8D8AAAAAAAAAAAAAAAAAAPA/AAAAAAAA8D8AAAAAAAAAAAAAAAAAAAAAAAAAAAAA8D8AAAAAAAAAAAAAAAAAAPA/AAAAAAAA8D8AAAAAAAAAAAAAAAAAAAAAAAAAAAAAAAAAAAAAAAAAAAAAAAAAAAAAAAAAAAAA8D8AAAAAAAAAAAAAAAAAAAAAAAAAAAAA8D8AAAAAAAAAAAAAAAAAAAAAAAAAAAAAAAAAAAAAAADwPwAAAAAAAAAAAAAAAAAAAAAAAAAAAAAAAAAAAAAAAAAAAAAAAAAA8D8AAAAAAAAAAAAAAAAAAAAAAAAAAAAAAAAAAAAAAAAAAAAAAAAAAPA/AAAAAAAAAAAAAAAAAAAAAAAAAAAAAAAAAAAAAAAA8D8AAAAAAADwPwAAAAAAAPA/AAAAAAAAAAAAAAAAAADwPwAAAAAAAAAAAAAAAAAA8D8AAAAAAAAAAAAAAAAAAAAAAAAAAAAAAAAAAAAAAAAAAAAAAAAAAAAAAAAAAAAA8D8AAAAAAAAAAAAAAAAAAAAAAAAAAAAAAAAAAAAAAAAAAAAAAAAAAAAAAAAAAAAA8D8AAAAAAAAAAAAAAAAAAAAAAAAAAAAAAAAAAAAAAAAAAAAAAAAAAPA/AAAAAAAAAAAAAAAAAAAAAAAAAAAAAPA/AAAAAAAAAAAAAAAAAAAAAAAAAAAAAPA/AAAAAAAA8D8AAAAAAADwPwAAAAAAAPA/AAAAAAAAAAAAAAAAAADwPwAAAAAAAAAAAAAAAAAA8D8AAAAAAAAAAAAAAAAAAAAAAAAAAAAA8D8AAAAAAADwPwAAAAAAAAAAAAAAAAAA8D8AAAAAAAAAAAAAAAAAAPA/AAAAAAAAAAAAAAAAAAAAAAAAAAAAAPA/AAAAAAAAAAAAAAAAAADwPwAAAAAAAAAAAAAAAAAAAAAAAAAAAADwPwAAAAAAAAAAAAAAAAAA8D8AAAAAAAAAAAAAAAAAAAAAAAAAAAAAAAAAAAAAAADwPwAAAAAAAPA/AAAAAAAA8D8AAAAAAADwPwAAAAAAAPA/AAAAAAAA8D8AAAAAAAAAAAAAAAAAAAAAAAAAAAAA8D8AAAAAAAAAAAAAAAAAAAAAAAAAAAAAAAAAAAAAAAAAAAAAAAAAAAAAAAAAAAAA8D8AAAAAAAAAAAAAAAAAAPA/AAAAAAAAAAAAAAAAAAAAAAAAAAAAAAAAAAAAAAAA8D8AAAAAAADwPwAAAAAAAPA/AAAAAAAAAAAAAAAAAAAAAAAAAAAAAPA/AAAAAAAA8D8AAAAAAADwPwAAAAAAAAAAAAAAAAAAAAAAAAAAAAAAAAAAAAAAAAAAAAAAAAAAAAAAAAAAAAAAAAAAAAAAAPA/AAAAAAAAAAAAAAAAAADwPwAAAAAAAAAAAAAAAAAAAAAAAAAAAAAAAAAAAAAAAAAAAAAAAAAAAAAAAAAAAADwPwAAAAAAAPA/AAAAAAAAAAAAAAAAAAAAAAAAAAAAAAAAAAAAAAAAAAAAAAAAAADwPwAAAAAAAAAAAAAAAAAA8D8AAAAAAADwPwAAAAAAAAAAAAAAAAAAAAAAAAAAAADwPwAAAAAAAPA/AAAAAAAAAAAAAAAAAADwPwAAAAAAAPA/AAAAAAAAAAAAAAAAAADwPwAAAAAAAPA/AAAAAAAA8D8AAAAAAAAAAAAAAAAAAAAAAAAAAAAAAAAAAAAAAAAAAAAAAAAAAPA/AAAAAAAA8D8AAAAAAADwPwAAAAAAAPA/AAAAAAAA8D8AAAAAAADwPwAAAAAAAPA/AAAAAAAAAAAAAAAAAAAAAAAAAAAAAPA/AAAAAAAA8D8AAAAAAAAAAAAAAAAAAPA/AAAAAAAAAAAAAAAAAAAAAAAAAAAAAPA/AAAAAAAAAAAAAAAAAAAAAAAAAAAAAAAAAAAAAAAAAAAAAAAAAADwPwAAAAAAAPA/AAAAAAAA8D8AAAAAAAAAAAAAAAAAAPA/AAAAAAAAAAAAAAAAAAAAAAAAAAAAAAAAAAAAAAAAAAAAAAAAAAAAAAAAAAAAAPA/AAAAAAAAAAAAAAAAAAAAAAAAAAAAAPA/AAAAAAAA8D8AAAAAAAAAAAAAAAAAAAAAAAAAAAAA8D8AAAAAAAAAAAAAAAAAAAAAAAAAAAAAAAAAAAAAAADwPwAAAAAAAAAAAAAAAAAAAAAAAAAAAAAAAAAAAAAAAAAAAAAAAAAAAAAAAAAAAADwPwAAAAAAAAAAAAAAAAAA8D8AAAAAAAAAAAAAAAAAAPA/AAAAAAAAAAAAAAAAAADwPwAAAAAAAAAAAAAAAAAAAAAAAAAAAAAAAAAAAAAAAPA/AAAAAAAA8D8AAAAAAAAAAAAAAAAAAAAAAAAAAAAA8D8AAAAAAAAAAAAAAAAAAAAAAAAAAAAAAAAAAAAAAAAAAAAAAAAAAPA/AAAAAAAA8D8AAAAAAAAAAAAAAAAAAPA/AAAAAAAAAAAAAAAAAAAAAAAAAAAAAPA/AAAAAAAAAAAAAAAAAADwPwAAAAAAAAAAAAAAAAAAAAAAAAAAAAAAAAAAAAAAAPA/AAAAAAAAAAAAAAAAAAAAAAAAAAAAAAAAAAAAAAAAAAAAAAAAAADwPwAAAAAAAPA/AAAAAAAAAAAAAAAAAAAAAAAAAAAAAAAAAAAAAAAAAAAAAAAAAAAAAAAAAAAAAAAAAAAAAAAAAAAAAAAAAADwPwAAAAAAAAAAAAAAAAAA8D8AAAAAAAAAAAAAAAAAAPA/AAAAAAAAAAAAAAAAAAAAAAAAAAAAAPA/AAAAAAAA8D8AAAAAAAAAAAAAAAAAAAAAAAAAAAAAAAAAAAAAAADwPwAAAAAAAAAAAAAAAAAAAAAAAAAAAAAAAAAAAAAAAAAAAAAAAAAA8D8AAAAAAAAAAAAAAAAAAAAAAAAAAAAAAAAAAAAAAADwPwAAAAAAAAAAAAAAAAAAAAAAAAAAAAAAAAAAAAAAAPA/AAAAAAAA8D8AAAAAAADwPwAAAAAAAPA/AAAAAAAAAAAAAAAAAAAAAAAAAAAAAPA/AAAAAAAAAAAAAAAAAAAAAAAAAAAAAPA/AAAAAAAAAAAAAAAAAADwPwAAAAAAAPA/AAAAAAAAAAAAAAAAAAAAAAAAAAAAAAAAAAAAAAAAAAAAAAAAAADwPwAAAAAAAAAAAAAAAAAAAAAAAAAAAAAAAAAAAAAAAPA/AAAAAAAA8D8AAAAAAAAAAAAAAAAAAPA/AAAAAAAAAAAAAAAAAAAAAAAAAAAAAAAAAAAAAAAAAAAAAAAAAAAAAAAAAAAAAAAAAAAAAAAAAAAAAAAAAAAAAAAAAAAAAAAAAAAAAAAAAAAPZQAAALABAAAGAAAAAAAAAAAAAAAAAAAAAAAAAAAAAPA/AAAAAAAA8D8AAAAAAAAAAAAAAAAAAAAAAAAAAAAAAAAAAAAAAADwPwAAAAAAAAAAAAAAAAAAAAAAAAAAAAAAAAAAAAAAAAAAAAAAAAAAAAAAAAAAAAAAAAAAAAAAAAAAAAAAAAAAAAAAAAAAAADwPwAAAAAAAAAAAAAAAAAAAAAAAAAAAAAAAAAAAAAAAAAAAAAAAAAAAAAAAAAAAAAAAAAAAAAAAAAAAAAAAAAAAAAAAAAAAAAAAAAAAAAAAAAAAAAAAAAAAAAAAAAAAAAAAAAAAAAAAAAAAAAAAAAAAAAAAAAAAAAAAAAAAAAAAPA/AAAAAAAAAAAAAAAAAAAAAAAAAAAAAAAAAAAAAAAAAAAAAAAAAAAAAAAAAAAAAAAAAAAAAAAAAAAAAAAAAAAAAAAAAAAAAPA/AAAAAAAAAAAAAAAAAAAAAAAAAAAAAAAAAAAAAAAAAAAAAAAAAAAAAAAAAAAAAAAAAAAAAAAA8D8AAAAAAAAAAAAAAAAAAAAAAAAAAAAA8D8AAAAAAAAAAAAAAAAAAPA/AAAAAAAAAAAAAAAAAAAAAAAAAAAAAAAAAAAAAAAAAAAAAAAAAAAAAAAAAAAAAPA/AAAAAAAA8D8AAAAAAAAAAAAAAAAAAAAAAAAAAAAAAAAAAAAAAAAAAAAAAAAAAPA/AAAAAAAAAAAAAAAAAADwPwAAAAAAAPA/AAAAAAAAAAAAAAAAAAAAAAAAAAAAAPA/AAAAAAAAAAAAAAAAAAAAAAAAAAAAAPA/AAAAAAAA8D8AAAAAAADwPwAAAAAAAAAAAAAAAAAAAAAAAAAAAAAAAAAAAAAAAPA/AAAAAAAAAAAAAAAAAAAAAAAAAAAAAAAAAAAAAAAA8D8AAAAAAADwPwAAAAAAAAAAAAAAAAAAAAAAAAAAAAAAAAAAAAAAAAAAAAAAAAAAAAAAAAAAAAAAAAAAAAAAAAAAAAAAAAAAAAAAAAAAAADwPwAAAAAAAAAAAAAAAAAA8D8AAAAAAAAAAAAAAAAAAPA/AAAAAAAAAAAAAAAAAADwPwAAAAAAAPA/AAAAAAAA8D8AAAAAAADwPwAAAAAAAAAAAAAAAAAAAAAAAAAAAAAAAAAAAAAAAAAAAAAAAAAAAAAAAAAAAADwPwAAAAAAAAAAAAAAAAAA8D8AAAAAAAAAAAAAAAAAAAAAAAAAAAAAAAAAAAAAAADwPwAAAAAAAPA/AAAAAAAA8D8AAAAAAADwPwAAAAAAAAAAAAAAAAAA8D8AAAAAAAAAAAAAAAAAAAAAAAAAAAAAAAAAAAAAAADwPwAAAAAAAAAAAAAAAAAA8D8AAAAAAAAAAAAAAAAAAAAAAAAAAAAAAAAAAAAAAADwPwAAAAAAAAAAAAAAAAAAAAAAAAAAAAAAAAAAAAAAAAAAAAAAAAAA8D8AAAAAAADwPwAAAAAAAAAAAAAAAAAA8D8AAAAAAADwPwAAAAAAAAAAAAAAAAAAAAAAAAAAAADwPwAAAAAAAPA/AAAAAAAA8D8AAAAAAAAAAAAAAAAAAAAAAAAAAAAAAAAAAAAAAADwPwAAAAAAAAAAAAAAAAAAAAAAAAAAAAAAAAAAAAAAAAAAAAAAAAAAAAAAAAAAAADwPwAAAAAAAAAAAAAAAAAAAAAAAAAAAAAAAAAAAAAAAPA/AAAAAAAAAAAAAAAAAAAAAAAAAAAAAAAAAAAAAAAA8D8AAAAAAADwPwAAAAAAAAAAAAAAAAAAAAAAAAAAAADwPwAAAAAAAAAAAAAAAAAAAAAAAAAAAADwPwAAAAAAAAAAAAAAAAAAAAAAAAAAAADwPwAAAAAAAAAAAAAAAAAA8D8AAAAAAADwPwAAAAAAAAAAAAAAAAAA8D8AAAAAAAAAAAAAAAAAAPA/AAAAAAAA8D8AAAAAAAAAAAAAAAAAAAAAAAAAAAAA8D8AAAAAAAAAAAAAAAAAAAAAAAAAAAAAAAAAAAAAAAAAAAAAAAAAAAAAAAAAAAAAAAAAAAAAAAAAAAAAAAAAAAAAAAAAAAAA8D8AAAAAAADwPwAAAAAAAAAAAAAAAAAAAAAAAAAAAADwPwAAAAAAAAAAAAAAAAAA8D8AAAAAAAAAAAAAAAAAAPA/AAAAAAAA8D8AAAAAAAAAAAAAAAAAAPA/AAAAAAAAAAAAAAAAAADwPwAAAAAAAPA/AAAAAAAAAAAAAAAAAAAAAAAAAAAAAAAAAAAAAAAAAAAAAAAAAADwPwAAAAAAAPA/AAAAAAAAAAAAAAAAAAAAAAAAAAAAAAAAAAAAAAAAAAAAAAAAAAAAAAAAAAAAAAAAAAAAAAAAAAAAAAAAAAAAAAAAAAAAAAAAAAAAAAAAAAAAAAAAAADwPwAAAAAAAAAAAAAAAAAA8D8AAAAAAADwPwAAAAAAAAAAAAAAAAAAAAAAAAAAAAAAAAAAAAAAAAAAAAAAAAAA8D8AAAAAAAAAAAAAAAAAAAAAAAAAAAAAAAAAAAAAAAAAAAAAAAAAAAAAAAAAAAAA8D8AAAAAAAAAAAAAAAAAAAAAAAAAAAAAAAAAAAAAAAAAAAAAAAAAAAAAAAAAAAAA8D8AAAAAAADwPwAAAAAAAPA/AAAAAAAAAAAAAAAAAAAAAAAAAAAAAAAAAAAAAAAAAAAAAAAAAADwPwAAAAAAAAAAAAAAAAAA8D8AAAAAAADwPwAAAAAAAPA/AAAAAAAAAAAAAAAAAAAAAAAAAAAAAPA/AAAAAAAAAAAAAAAAAAAAAAAAAAAAAAAAAAAAAAAAAAAAAAAAAADwPwAAAAAAAAAAAAAAAAAAAAAAAAAAAADwPwAAAAAAAPA/AAAAAAAAAAAAAAAAAAAAAAAAAAAAAAAAAAAAAAAAAAAAAAAAAAAAAAAAAAAAAAAAAAAAAAAAAAAAAAAAAAAAAAAAAAAAAAAAAAAAAAAA8D8AAAAAAAAAAAAAAAAAAAAAAAAAAAAAAAAAAAAAAAAAAAAAAAAAAAAAAAAAAAAAAAAAAAAAAAAAAAAAAAAAAAAAAAAAAAAAAAAAAAAAAAAAAAAAAAAAAAAAAAAAAAAA8D8AAAAAAAAAAAAAAAAAAAAAAAAAAAAA8D8AAAAAAAAAAAAAAAAAAPA/AAAAAAAA8D8AAAAAAAAAAAAAAAAAAAAAAAAAAAAA8D8AAAAAAAAAAAAAAAAAAPA/AAAAAAAAAAAAAAAAAAAAAAAAAAAAAAAAAAAAAAAA8D8AAAAAAAAAAAAAAAAAAPA/AAAAAAAA8D8AAAAAAADwPwAAAAAAAPA/AAAAAAAA8D8AAAAAAAAAAAAAAAAAAAAAAAAAAAAA8D8AAAAAAAAAAAAAAAAAAAAAAAAAAAAA8D8AAAAAAAAAAAAAAAAAAAAAAAAAAAAAAAAAAAAAAADwPwAAAAAAAAAAAAAAAAAAAAAAAAAAAADwPwAAAAAAAAAAAAAAAAAA8D8AAAAAAADwPwAAAAAAAPA/AAAAAAAAAAAAAAAAAAAAAAAAAAAAAAAAAAAAAAAAAAAAAAAAAAAAAAAAAAAAAAAAAAAAAAAAAAAAAAAAAADwPwAAAAAAAPA/AAAAAAAA8D8AAAAAAAAAAAAAAAAAAAAAAAAAAAAAAAAAAAAAAADwPwAAAAAAAAAAAAAAAAAAAAAAAAAAAAAAAAAAAAAAAPA/AAAAAAAA8D8AAAAAAAAAAAAAAAAAAAAAAAAAAAAA8D8AAAAAAAAAAAAAAAAAAPA/AAAAAAAA8D8AAAAAAAAAAAAAAAAAAPA/AAAAAAAAAAAAAAAAAAAAAAAAAAAAAAAAAAAAAAAA8D8AAAAAAAAAAAAAAAAAAAAAAAAAAAAA8D8AAAAAAAAAAAAAAAAAAPA/AAAAAAAAAAAAAAAAAAAAAAAAAAAAAAAAAAAAAAAA8D8AAAAAAAAAAAAAAAAAAPA/AAAAAAAA8D8AAAAAAAAAAAAAAAAAAPA/AAAAAAAAAAAAAAAAAAAAAAAAAAAAAAAAAAAAAAAA8D8AAAAAAAAAAAAAAAAAAAAAAAAAAAAA8D8AAAAAAAAAAAAAAAAAAAAAAAAAAAAA8D8AAAAAAADwPwAAAAAAAAAAAAAAAAAAAAAAAAAAAADwPwAAAAAAAPA/AAAAAAAA8D8AAAAAAAAAAAAAAAAAAAAAAAAAAAAAAAAAAAAAAAAAAAAAAAAAAPA/AAAAAAAAAAAAAAAAAADwPwAAAAAAAPA/AAAAAAAAAAAAAAAAAAAAAAAAAAAAAAAAAAAAAAAAAAAAAAAAAAAAAAAAAAAAAAAAAAAAAAAAAAAAAAAAAAAAAAAAAAAAAAAAAAAAAAAA8D8AAAAAAAAAAAAAAAAAAAAAAAAAAAAAAAAAAAAAAAAAAAAAAAAAAAAAAAAAAAAA8D8AAAAAAAAAAAAAAAAAAAAAAAAAAAAAAAAAAAAAAADwPwAAAAAAAAAAAAAAAAAAAAAAAAAAAAAAAAAAAAAAAAAAAAAAAAAAAAAAAAAAAAAAAAAAAAAAAPA/AAAAAAAAAAAAAAAAAAAAAAAAAAAAAPA/AAAAAAAAAAAAAAAAAAAAAAAAAAAAAPA/AAAAAAAA8D8AAAAAAAAAAAAAAAAAAAAAD2cAAACwAQAABgAAAAAAAAAAAAAAAAAAAAAAAAAAAAAAAAAAAAAAAAAAAAAAAAAAAAAAAAAAAAAAAAAAAAAAAAAAAAAAAAAAAAAAAAAAAAAAAAAAAAAAAAAAAAAAAAAAAAAAAAAAAAAAAAAAAAAAAAAAAAAAAAAAAAAAAAAAAAAAAAAAAAAAAAAAAAAAAAAAAAAAAAAAAAAAAAAAAAAAAAAAAAAAAAAAAAAAAAAAAAAAAAAAAAAAAAAAAAAAAAAAAAAAAAAAAAAAAAAAAAAAAAAAAAAAAAAAAAAAAAAAAAAAAAAAAAAAAAAAAAAAAAAAAAAAAAAAAAAAAAAAAAAAAAAAAAAAAAAAAAAAAAAAAAAAAAAAAAAAAAAAAAAAAAAAAAAAAAAAAAAAAAAAAAAAAAAAAAAAAAAAAAAAAAAAAAAAAAAAAAAAAAAAAAAAAAAAAAAAAAAAAAAAAAAAAAAAAAAAAAAAAAAAAAAAAAAAAAAAAAAAAAAAAAAAAAAAAAAAAAAAAAAAAAAAAAAAAAAAAAAAAAAAAAAAAAAAAAAAAAAAAAAAAAAAAAAAAAAAAAAAAAAAAAAAAAAAAAAAAAAAAAAAAAAAAAAAAAAAAAAAAAAAAAAAAAAAAAAAAAAAAAAAAAAAAAAAAAAAAAAAAAAAAAAAAAAAAAAAAAAAAAAAAAAAAAAAAAAAAAAAAAAAAAAAAAAAAAAAAAAAAAAAAAAAAAAAAAAAAAAAAAAAAAAAAAAAAAAAAAAAAAAAAAAAAAAAAAAAAAAAAAAAAAAAAAAAAAAAAAAAAAAAAAAAAAAAAAAAAAAAAAAAAAAAAAAAAAAAAAAAAAAAAAAAAAAAAAAAAAAAAAAAAAAA8D8AAAAAAAAAAAAAAAAAAAAAAAAAAAAAAAAAAAAAAAAAAAAAAAAAAAAAAAAAAAAAAAAAAAAAAADwPwAAAAAAAAAAAAAAAAAAAAAAAAAAAAAAAAAAAAAAAAAAAAAAAAAAAAAAAAAAAAAAAAAAAAAAAAAAAAAAAAAAAAAAAAAAAAAAAAAAAAAAAAAAAAAAAAAAAAAAAAAAAAAAAAAAAAAAAAAAAAAAAAAAAAAAAAAAAAAAAAAAAAAAAAAAAAAAAAAAAAAAAAAAAAAAAAAAAAAAAAAAAAAAAAAAAAAAAAAAAADwPwAAAAAAAAAAAAAAAAAAAAAAAAAAAADwPwAAAAAAAAAAAAAAAAAAAAAAAAAAAAAAAAAAAAAAAAAAAAAAAAAAAAAAAAAAAAAAAAAAAAAAAAAAAAAAAAAAAAAAAAAAAAAAAAAAAAAAAAAAAAAAAAAAAAAAAAAAAAAAAAAAAAAAAAAAAAAAAAAAAAAAAAAAAAAAAAAAAAAAAAAAAAAAAAAAAAAAAAAAAAAAAAAAAAAAAPA/AAAAAAAAAAAAAAAAAAAAAAAAAAAAAPA/AAAAAAAA8D8AAAAAAAAAAAAAAAAAAAAAAAAAAAAAAAAAAAAAAAAAAAAAAAAAAAAAAAAAAAAAAAAAAAAAAADwPwAAAAAAAAAAAAAAAAAAAAAAAAAAAAAAAAAAAAAAAAAAAAAAAAAAAAAAAAAAAAAAAAAAAAAAAAAAAAAAAAAAAAAAAAAAAAAAAAAAAAAAAAAAAAAAAAAAAAAAAAAAAAAAAAAAAAAAAAAAAAAAAAAAAAAAAAAAAAAAAAAAAAAAAAAAAAAAAAAAAAAAAAAAAAAAAAAAAAAAAAAAAAAAAAAAAAAAAAAAAAAAAAAAAAAAAAAAAAAAAAAAAAAAAAAAAAAAAAAAAAAAAAAAAAAAAAAAAAAAAAAAAAAAAAAAAAAAAPA/AAAAAAAAAAAAAAAAAADwPwAAAAAAAAAAAAAAAAAAAAAAAAAAAAAAAAAAAAAAAAAAAAAAAAAAAAAAAAAAAAAAAAAAAAAAAAAAAAAAAAAAAAAAAAAAAAAAAAAAAAAAAAAAAAAAAAAAAAAAAAAAAAAAAAAAAAAAAAAAAAAAAAAAAAAAAAAAAAAAAAAAAAAAAAAAAAAAAAAAAAAAAAAAAADwPwAAAAAAAAAAAAAAAAAA8D8AAAAAAAAAAAAAAAAAAAAAAAAAAAAAAAAAAAAAAAAAAAAAAAAAAAAAAAAAAAAAAAAAAAAAAAAAAAAAAAAAAAAAAAAAAAAAAAAAAAAAAAAAAAAAAAAAAAAAAAAAAAAAAAAAAAAAAAAAAAAAAAAAAAAAAAAAAAAAAAAAAAAAAAAAAAAAAAAAAAAAAAAAAAAAAAAAAAAAAAAAAAAAAAAAAAAAAAAAAAAAAAAAAAAAAAAAAAAAAAAAAAAAAAAAAAAAAAAAAAAAAAAAAAAAAAAAAAAAAAAAAAAAAAAAAAAAAAAAAAAAAAAAAAAAAAAAAAAA8D8AAAAAAAAAAAAAAAAAAAAAAAAAAAAAAAAAAAAAAADwPwAAAAAAAAAAAAAAAAAAAAAAAAAAAAAAAAAAAAAAAAAAAAAAAAAAAAAAAAAAAADwPwAAAAAAAAAAAAAAAAAA8D8AAAAAAAAAAAAAAAAAAAAAAAAAAAAAAAAAAAAAAADwPwAAAAAAAAAAAAAAAAAAAAAAAAAAAAAAAAAAAAAAAAAAAAAAAAAA8D8AAAAAAAAAAAAAAAAAAAAAAAAAAAAAAAAAAAAAAAAAAAAAAAAAAAAAAAAAAAAAAAAAAAAAAAAAAAAAAAAAAAAAAAAAAAAAAAAAAAAAAAAAAAAAAAAAAAAAAAAAAAAAAAAAAAAAAAAAAAAAAAAAAAAAAAAAAAAAAAAAAAAAAAAAAAAAAAAAAAAAAAAAAAAA8D8AAAAAAAAAAAAAAAAAAAAAAAAAAAAAAAAAAAAAAAAAAAAAAAAAAAAAAAAAAAAAAAAAAAAAAAAAAAAAAAAAAAAAAAAAAAAAAAAAAAAAAAAAAAAAAAAAAAAAAAAAAAAAAAAAAAAAAADwPwAAAAAAAAAAAAAAAAAAAAAAAAAAAAAAAAAAAAAAAAAAAAAAAAAAAAAAAAAAAAAAAAAAAAAAAPA/AAAAAAAAAAAAAAAAAAAAAAAAAAAAAAAAAAAAAAAAAAAAAAAAAAAAAAAAAAAAAAAAAAAAAAAAAAAAAAAAAAAAAAAAAAAAAAAAAAAAAAAAAAAAAAAAAAAAAAAAAAAAAAAAAAAAAAAAAAAAAAAAAAAAAAAAAAAAAAAAAAAAAAAAAAAAAAAAAAAAAAAAAAAAAAAAAAAAAAAAAAAAAAAAAAAAAAAAAAAAAAAAAAAAAAAAAAAAAAAAAAAAAAAAAAAAAAAAAAAAAAAAAAAAAAAAAAAAAAAAAAAAAAAAAAAAAAAAAAAAAAAAAAAAAAAAAAAAAAAAAAAAAAAAAAAAAAAAAAAAAAAAAAAAAAAAAAAAAAAAAAAAAAAAAAAAAAAAAAAAAAAAAAAAAAAAAAAAAAAAAAAAAAAAAAAAAPA/AAAAAAAAAAAAAAAAAADwPwAAAAAAAAAAAAAAAAAAAAAAAAAAAAAAAAAAAAAAAAAAAAAAAAAAAAAAAAAAAAAAAAAAAAAAAAAAAAAAAAAAAAAAAAAAAAAAAAAAAAAAAPA/AAAAAAAAAAAAAAAAAADwPwAAAAAAAAAAAAAAAAAAAAAAAAAAAAAAAAAAAAAAAAAAAAAAAAAAAAAAAAAAAAAAAAAAAAAAAPA/AAAAAAAAAAAAAAAAAAAAAAAAAAAAAPA/AAAAAAAA8D8AAAAAAAAAAAAAAAAAAAAAAAAAAAAAAAAAAAAAAAAAAAAAAAAAAAAAAAAAAAAAAAAAAAAAAAAAAAAAAAAAAAAAAAAAAAAAAAAAAAAAAAAAAAAAAAAAAAAAAAAAAAAA8D8AAAAAAADwPwAAAAAAAAAAAAAAAAAAAAAAAAAAAADwPwAAAAAAAAAAAAAAAAAA8D8AAAAAAAAAAAAAAAAAAAAAAAAAAAAAAAAAAAAAAAAAAAAAAAAAAAAAAAAAAAAAAAAAAAAAAAAAAAAAAAAAAAAAAAAAAAAA8D8AAAAAAAAAAAAAAAAAAAAAAAAAAAAA8D8AAAAAAAAAAAAAAAAAAAAAAAAAAAAAAAAAAAAAAADwPwAAAAAAAAAAAAAAAAAAAAAAAAAAAAAAAAAAAAAAAAAAAAAAAAAAAAAAAAAAAAAAAAAAAAAAAAAAAAAAAAAAAAAAAAAAAAAAAAAAAAAAAAAAAAAAAAAA8D8AAAAAAAAAAAAAAAAAAPA/AAAAAAAAAAAAAAAAAAAAAAAAAAAAAAAAAAAAAAAAAAAAAAAAAAAAAAAAAAAAAAAAAAAAAAAAAAAAAAAAAAAAAAAAAAAAAAAAAAAAAAAAAAAAAAAAAAAAAAAAAAAAAAAAAAAAAAAAAAAAAAAAAAAAAAAAAAAAAAAAAAAAAAAAAAAAAAAAAAAAAAAAAAAAAAAAAAAAAAAAAAAAAAAAAADwPwAAAAAAAAAAAAAAAAAA8D8AAAAAAAAAAAAAAAAAAAAAAAAAAAAAAAAAAAAAAAAAAAAAAAAAAPA/AAAAAAAAAAAAAAAAAAAAAAAAAAAAAAAAAAAAAAAAAAAAAAAAAAAAAAAAAAAAAAAAAAAAAAAAAAAAAAAAAAAAAAAAAAAAAAAAAAAAAAAAAAAAAAAAAAAAAAAAAAAAAAAAAAAAAAAA8D8AAAAAAAAAAAVqAAAAIFN0YXRzRGlyZWN0LkRhdGEuV29ya3NoZWV0T3JpZ2luCQAAABc8Q29sdW1uPmtfX0JhY2tpbmdGaWVsZBU8TW9kZT5rX19CYWNraW5nRmllbGQXPFRvcFJvdz5rX19CYWNraW5nRmllbGQVPFJvd3M+a19fQmFja2luZ0ZpZWxkHTxXb3JrYm9va1BhdGg+a19fQmFja2luZ0ZpZWxkHjxXb3Jrc2hlZXROYW1lPmtfX0JhY2tpbmdGaWVsZBk8SGFzVGl0bGU+a19fQmFja2luZ0ZpZWxkHDxXYXNGaWx0ZXJlZD5rX19CYWNraW5nRmllbGQcPE9yaWdpbkdyb3VwPmtfX0JhY2tpbmdGaWVsZAAEAAABAQAAAAgpU3RhdHNEaXJlY3QuVXRpbGl0aWVzLkRhdGFBY3F1aXNpdGlvbk1vZGUCAAAACAgBAQgCAAAAHAAAAAV5////KVN0YXRzRGlyZWN0LlV0aWxpdGllcy5EYXRhQWNxdWlzaXRpb25Nb2RlAQAAAAd2YWx1ZV9fAAgCAAAAagAAAAAAAACxAQAABogAAAAsQzpcVXNlcnNcaGFuc1xBcHBEYXRhXExvY2FsXFRlbXBcfkV4Y2VsLnhsc3gGiQAAAAhNYXRjaGluZwAAAQAAAA9rAAAAsAEAAAYAAAAAAADwPwAAAAAAAPA/AAAAAAAA8D8AAAAAAADwPwAAAAAAAPA/AAAAAAAAAAAAAAAAAADwPwAAAAAAAPA/AAAAAAAA8D8AAAAAAADwPwAAAAAAAAAAAAAAAAAA8D8AAAAAAAAAAAAAAAAAAPA/AAAAAAAA8D8AAAAAAADwPwAAAAAAAAAAAAAAAAAAAAAAAAAAAADwPwAAAAAAAAAAAAAAAAAA8D8AAAAAAADwPwAAAAAAAPA/AAAAAAAA8D8AAAAAAADwPwAAAAAAAAAAAAAAAAAA8D8AAAAAAAAAAAAAAAAAAPA/AAAAAAAAAAAAAAAAAADwPwAAAAAAAPA/AAAAAAAAAAAAAAAAAADwPwAAAAAAAPA/AAAAAAAA8D8AAAAAAADwPwAAAAAAAPA/AAAAAAAA8D8AAAAAAADwPwAAAAAAAPA/AAAAAAAA8D8AAAAAAADwPwAAAAAAAPA/AAAAAAAA8D8AAAAAAADwPwAAAAAAAAAAAAAAAAAA8D8AAAAAAADwPwAAAAAAAPA/AAAAAAAA8D8AAAAAAADwPwAAAAAAAPA/AAAAAAAA8D8AAAAAAADwPwAAAAAAAPA/AAAAAAAA8D8AAAAAAADwPwAAAAAAAPA/AAAAAAAA8D8AAAAAAADwPwAAAAAAAAAAAAAAAAAA8D8AAAAAAADwPwAAAAAAAPA/AAAAAAAAAAAAAAAAAADwPwAAAAAAAPA/AAAAAAAAAAAAAAAAAADwPwAAAAAAAAAAAAAAAAAA8D8AAAAAAADwPwAAAAAAAPA/AAAAAAAA8D8AAAAAAADwPwAAAAAAAPA/AAAAAAAA8D8AAAAAAAAAAAAAAAAAAAAAAAAAAAAA8D8AAAAAAADwPwAAAAAAAPA/AAAAAAAA8D8AAAAAAAAAAAAAAAAAAPA/AAAAAAAA8D8AAAAAAADwPwAAAAAAAPA/AAAAAAAA8D8AAAAAAADwPwAAAAAAAPA/AAAAAAAA8D8AAAAAAADwPwAAAAAAAPA/AAAAAAAAAAAAAAAAAADwPwAAAAAAAPA/AAAAAAAA8D8AAAAAAADwPwAAAAAAAPA/AAAAAAAA8D8AAAAAAADwPwAAAAAAAPA/AAAAAAAA8D8AAAAAAADwPwAAAAAAAPA/AAAAAAAA8D8AAAAAAADwPwAAAAAAAAAAAAAAAAAAAAAAAAAAAADwPwAAAAAAAAAAAAAAAAAA8D8AAAAAAAAAAAAAAAAAAPA/AAAAAAAAAAAAAAAAAADwPwAAAAAAAPA/AAAAAAAA8D8AAAAAAADwPwAAAAAAAPA/AAAAAAAA8D8AAAAAAADwPwAAAAAAAPA/AAAAAAAAAAAAAAAAAADwPwAAAAAAAPA/AAAAAAAAAAAAAAAAAADwPwAAAAAAAPA/AAAAAAAA8D8AAAAAAAAAAAAAAAAAAAAAAAAAAAAA8D8AAAAAAAAAAAAAAAAAAPA/AAAAAAAA8D8AAAAAAADwPwAAAAAAAAAAAAAAAAAA8D8AAAAAAAAAAAAAAAAAAPA/AAAAAAAA8D8AAAAAAADwPwAAAAAAAPA/AAAAAAAA8D8AAAAAAADwPwAAAAAAAPA/AAAAAAAAAAAAAAAAAADwPwAAAAAAAPA/AAAAAAAA8D8AAAAAAADwPwAAAAAAAAAAAAAAAAAA8D8AAAAAAADwPwAAAAAAAPA/AAAAAAAA8D8AAAAAAADwPwAAAAAAAPA/AAAAAAAA8D8AAAAAAADwPwAAAAAAAPA/AAAAAAAA8D8AAAAAAADwPwAAAAAAAPA/AAAAAAAA8D8AAAAAAADwPwAAAAAAAPA/AAAAAAAA8D8AAAAAAADwPwAAAAAAAPA/AAAAAAAA8D8AAAAAAADwPwAAAAAAAPA/AAAAAAAAAAAAAAAAAADwPwAAAAAAAPA/AAAAAAAA8D8AAAAAAADwPwAAAAAAAPA/AAAAAAAA8D8AAAAAAADwPwAAAAAAAPA/AAAAAAAA8D8AAAAAAADwPwAAAAAAAPA/AAAAAAAA8D8AAAAAAADwPwAAAAAAAPA/AAAAAAAA8D8AAAAAAADwPwAAAAAAAPA/AAAAAAAA8D8AAAAAAADwPwAAAAAAAPA/AAAAAAAA8D8AAAAAAADwPwAAAAAAAPA/AAAAAAAA8D8AAAAAAADwPwAAAAAAAPA/AAAAAAAA8D8AAAAAAADwPwAAAAAAAPA/AAAAAAAA8D8AAAAAAADwPwAAAAAAAPA/AAAAAAAA8D8AAAAAAADwPwAAAAAAAPA/AAAAAAAAAAAAAAAAAADwPwAAAAAAAPA/AAAAAAAA8D8AAAAAAADwPwAAAAAAAPA/AAAAAAAAAAAAAAAAAADwPwAAAAAAAAAAAAAAAAAA8D8AAAAAAADwPwAAAAAAAPA/AAAAAAAAAAAAAAAAAADwPwAAAAAAAAAAAAAAAAAA8D8AAAAAAADwPwAAAAAAAPA/AAAAAAAAAAAAAAAAAADwPwAAAAAAAPA/AAAAAAAAAAAAAAAAAADwPwAAAAAAAAAAAAAAAAAAAAAAAAAAAADwPwAAAAAAAPA/AAAAAAAA8D8AAAAAAADwPwAAAAAAAAAAAAAAAAAA8D8AAAAAAAAAAAAAAAAAAAAAAAAAAAAA8D8AAAAAAAAAAAAAAAAAAPA/AAAAAAAA8D8AAAAAAADwPwAAAAAAAPA/AAAAAAAA8D8AAAAAAADwPwAAAAAAAPA/AAAAAAAA8D8AAAAAAADwPwAAAAAAAPA/AAAAAAAA8D8AAAAAAAAAAAAAAAAAAPA/AAAAAAAA8D8AAAAAAADwPwAAAAAAAAAAAAAAAAAA8D8AAAAAAADwPwAAAAAAAPA/AAAAAAAA8D8AAAAAAADwPwAAAAAAAAAAAAAAAAAA8D8AAAAAAADwPwAAAAAAAPA/AAAAAAAAAAAAAAAAAADwPwAAAAAAAPA/AAAAAAAAAAAAAAAAAAAAAAAAAAAAAPA/AAAAAAAA8D8AAAAAAADwPwAAAAAAAPA/AAAAAAAA8D8AAAAAAADwPwAAAAAAAPA/AAAAAAAA8D8AAAAAAADwPwAAAAAAAPA/AAAAAAAA8D8AAAAAAADwPwAAAAAAAAAAAAAAAAAA8D8AAAAAAADwPwAAAAAAAPA/AAAAAAAA8D8AAAAAAADwPwAAAAAAAPA/AAAAAAAA8D8AAAAAAAAAAAAAAAAAAPA/AAAAAAAA8D8AAAAAAADwPwAAAAAAAPA/AAAAAAAA8D8AAAAAAADwPwAAAAAAAPA/AAAAAAAAAAAAAAAAAADwPwAAAAAAAPA/AAAAAAAA8D8AAAAAAADwPwAAAAAAAPA/AAAAAAAAAAAAAAAAAAAAAAAAAAAAAPA/AAAAAAAA8D8AAAAAAADwPwAAAAAAAPA/AAAAAAAAAAAAAAAAAADwPwAAAAAAAPA/AAAAAAAAAAAAAAAAAADwPwAAAAAAAPA/AAAAAAAA8D8AAAAAAAAAAAAAAAAAAPA/AAAAAAAA8D8AAAAAAADwPwAAAAAAAPA/AAAAAAAA8D8AAAAAAADwPwAAAAAAAPA/AAAAAAAA8D8AAAAAAADwPwAAAAAAAPA/AAAAAAAA8D8AAAAAAAAAAAAAAAAAAAAAAAAAAAAA8D8AAAAAAADwPwAAAAAAAAAAAAAAAAAA8D8AAAAAAAAAAAAAAAAAAPA/AAAAAAAA8D8AAAAAAADwPwAAAAAAAPA/AAAAAAAA8D8AAAAAAADwPwAAAAAAAPA/AAAAAAAA8D8AAAAAAADwPwAAAAAAAPA/AAAAAAAA8D8AAAAAAADwPwAAAAAAAPA/AAAAAAAAAAAAAAAAAADwPwAAAAAAAPA/AAAAAAAA8D8AAAAAAADwPwAAAAAAAAAAAAAAAAAA8D8AAAAAAADwPwAAAAAAAPA/AAAAAAAA8D8AAAAAAADwPwAAAAAAAAAAAAAAAAAA8D8AAAAAAADwPwAAAAAAAAAAAAAAAAAA8D8AAAAAAADwPwAAAAAAAPA/AAAAAAAA8D8AAAAAAADwPwAAAAAAAPA/AAAAAAAA8D8AAAAAAADwPwAAAAAAAPA/AAAAAAAA8D8AAAAAAADwPwAAAAAAAPA/AAAAAAAA8D8AAAAAAADwPwAAAAAAAPA/AAAAAAAAAAAAAAAAAADwPwAAAAAAAPA/AAAAAAAA8D8AAAAAAAAAAAAAAAAAAPA/AAAAAAAA8D8AAAAAAADwPwAAAAAAAAAAAAAAAAAA8D8AAAAAAADwPwAAAAAAAPA/AAAAAAAA8D8AAAAAAADwPwAAAAAAAPA/AAAAAAAA8D8AAAAAAADwPwAAAAAAAPA/AAAAAAAA8D8AAAAAAAAAAAAAAAAAAPA/AAAAAAAAAAAAAAAAAADwPwAAAAAAAPA/AAAAAAAA8D8AAAAAAADwPwAAAAAAAAAAAAAAAAAA8D8AAAAAAADwPwAAAAAAAPA/AAAAAAAA8D8AAAAAAAAAAAAAAAAAAAAAAAAAAAAA8D8AAAAAAADwPwAAAAAAAPA/AAAAAAAA8D8AAAAAAADwPwAAAAAAAPA/AAAAAAAA8D8AAAAAAADwPwAAAAAAAPA/AAAAAAAA8D8AAAAAAAAAAAAAAAAAAPA/AAAAAAAA8D8PbQAAALABAAAGAAAAAAAAAAAAAAAAAAAAAAAAAAAAAAAAAAAAAAAAAAAAAAAAAAAAAAAAAAAAAPA/AAAAAAAAAAAAAAAAAAAAAAAAAAAAAAAAAAAAAAAAAAAAAAAAAADwPwAAAAAAAAAAAAAAAAAA8D8AAAAAAAAAAAAAAAAAAAAAAAAAAAAAAAAAAAAAAADwPwAAAAAAAPA/AAAAAAAAAAAAAAAAAADwPwAAAAAAAAAAAAAAAAAAAAAAAAAAAAAAAAAAAAAAAAAAAAAAAAAAAAAAAAAAAADwPwAAAAAAAAAAAAAAAAAA8D8AAAAAAAAAAAAAAAAAAPA/AAAAAAAAAAAAAAAAAAAAAAAAAAAAAAAAAAAAAAAAAAAAAAAAAAAAAAAAAAAAAAAAAAAAAAAAAAAAAAAAAAAAAAAAAAAAAAAAAAAAAAAAAAAAAAAAAAAAAAAAAAAAAAAAAAAAAAAAAAAAAAAAAAAAAAAAAAAAAAAAAAAAAAAAAAAAAAAAAADwPwAAAAAAAAAAAAAAAAAAAAAAAAAAAAAAAAAAAAAAAAAAAAAAAAAAAAAAAAAAAAAAAAAAAAAAAAAAAAAAAAAAAAAAAAAAAAAAAAAAAAAAAAAAAAAAAAAAAAAAAAAAAAAAAAAAAAAAAAAAAAAAAAAAAAAAAAAAAADwPwAAAAAAAAAAAAAAAAAAAAAAAAAAAAAAAAAAAAAAAPA/AAAAAAAAAAAAAAAAAAAAAAAAAAAAAPA/AAAAAAAAAAAAAAAAAAAAAAAAAAAAAAAAAAAAAAAAAAAAAAAAAAAAAAAAAAAAAAAAAAAAAAAAAAAAAAAAAAAAAAAAAAAAAAAAAAAAAAAAAAAAAAAAAADwPwAAAAAAAAAAAAAAAAAAAAAAAAAAAAAAAAAAAAAAAAAAAAAAAAAA8D8AAAAAAAAAAAAAAAAAAAAAAAAAAAAAAAAAAAAAAAAAAAAAAAAAAAAAAAAAAAAAAAAAAAAAAAAAAAAAAAAAAAAAAAAAAAAAAAAAAAAAAAAAAAAAAAAAAPA/AAAAAAAAAAAAAAAAAAAAAAAAAAAAAAAAAAAAAAAAAAAAAAAAAAAAAAAAAAAAAAAAAAAAAAAAAAAAAAAAAAAAAAAAAAAAAAAAAAAAAAAAAAAAAAAAAAAAAAAAAAAAAAAAAAAAAAAAAAAAAAAAAAAAAAAAAAAAAPA/AAAAAAAAAAAAAAAAAADwPwAAAAAAAAAAAAAAAAAA8D8AAAAAAAAAAAAAAAAAAPA/AAAAAAAAAAAAAAAAAAAAAAAAAAAAAAAAAAAAAAAAAAAAAAAAAAAAAAAAAAAAAAAAAAAAAAAAAAAAAAAAAAAAAAAAAAAAAAAAAAAAAAAAAAAAAAAAAAAAAAAAAAAAAPA/AAAAAAAAAAAAAAAAAAAAAAAAAAAAAAAAAAAAAAAA8D8AAAAAAADwPwAAAAAAAAAAAAAAAAAA8D8AAAAAAAAAAAAAAAAAAAAAAAAAAAAAAAAAAAAAAADwPwAAAAAAAAAAAAAAAAAA8D8AAAAAAAAAAAAAAAAAAAAAAAAAAAAAAAAAAAAAAAAAAAAAAAAAAAAAAAAAAAAAAAAAAAAAAAAAAAAAAAAAAPA/AAAAAAAAAAAAAAAAAAAAAAAAAAAAAAAAAAAAAAAAAAAAAAAAAADwPwAAAAAAAAAAAAAAAAAAAAAAAAAAAAAAAAAAAAAAAAAAAAAAAAAAAAAAAAAAAAAAAAAAAAAAAAAAAAAAAAAAAAAAAAAAAAAAAAAAAAAAAAAAAAAAAAAAAAAAAAAAAAAAAAAAAAAAAAAAAAAAAAAAAAAAAAAAAAAAAAAAAAAAAAAAAAAAAAAAAAAAAAAAAAAAAAAAAAAAAAAAAAAAAAAAAAAAAAAAAAAAAAAAAAAAAPA/AAAAAAAAAAAAAAAAAAAAAAAAAAAAAAAAAAAAAAAAAAAAAAAAAAAAAAAAAAAAAAAAAAAAAAAAAAAAAAAAAAAAAAAAAAAAAAAAAAAAAAAAAAAAAAAAAAAAAAAAAAAAAAAAAAAAAAAAAAAAAAAAAAAAAAAAAAAAAAAAAAAAAAAAAAAAAAAAAAAAAAAAAAAAAAAAAAAAAAAAAAAAAAAAAAAAAAAAAAAAAAAAAAAAAAAAAAAAAAAAAAAAAAAAAAAAAAAAAAAAAAAAAAAAAAAAAAAAAAAAAAAAAAAAAAAAAAAAAAAAAAAAAAAAAAAAAAAAAAAAAAAAAAAAAAAAAAAAAAAAAAAAAAAAAAAAAAAAAAAAAAAAAAAAAAAAAAAAAAAAAAAAAAAAAAAAAAAAAAAAAAAAAAAAAAAAAAAAAAAAAAAAAAAAAAAAAAAAAAAAAAAAAPA/AAAAAAAAAAAAAAAAAAAAAAAAAAAAAAAAAAAAAAAAAAAAAAAAAAAAAAAAAAAAAPA/AAAAAAAAAAAAAAAAAADwPwAAAAAAAAAAAAAAAAAAAAAAAAAAAAAAAAAAAAAAAAAAAAAAAAAAAAAAAAAAAAAAAAAAAAAAAPA/AAAAAAAAAAAAAAAAAADwPwAAAAAAAPA/AAAAAAAAAAAAAAAAAAAAAAAAAAAAAAAAAAAAAAAAAAAAAAAAAADwPwAAAAAAAAAAAAAAAAAA8D8AAAAAAADwPwAAAAAAAAAAAAAAAAAA8D8AAAAAAAAAAAAAAAAAAAAAAAAAAAAAAAAAAAAAAAAAAAAAAAAAAAAAAAAAAAAAAAAAAAAAAAAAAAAAAAAAAAAAAAAAAAAAAAAAAAAAAAAAAAAAAAAAAAAAAAAAAAAA8D8AAAAAAAAAAAAAAAAAAAAAAAAAAAAAAAAAAAAAAADwPwAAAAAAAAAAAAAAAAAAAAAAAAAAAAAAAAAAAAAAAAAAAAAAAAAAAAAAAAAAAADwPwAAAAAAAAAAAAAAAAAAAAAAAAAAAAAAAAAAAAAAAPA/AAAAAAAAAAAAAAAAAAAAAAAAAAAAAPA/AAAAAAAA8D8AAAAAAAAAAAAAAAAAAAAAAAAAAAAAAAAAAAAAAAAAAAAAAAAAAAAAAAAAAAAAAAAAAAAAAAAAAAAAAAAAAAAAAAAAAAAAAAAAAAAAAAAAAAAAAAAAAAAAAAAAAAAAAAAAAAAAAADwPwAAAAAAAAAAAAAAAAAAAAAAAAAAAAAAAAAAAAAAAAAAAAAAAAAAAAAAAAAAAAAAAAAAAAAAAAAAAAAAAAAA8D8AAAAAAAAAAAAAAAAAAAAAAAAAAAAAAAAAAAAAAAAAAAAAAAAAAAAAAAAAAAAAAAAAAAAAAAAAAAAAAAAAAAAAAAAAAAAAAAAAAAAAAAAAAAAAAAAAAAAAAAAAAAAAAAAAAAAAAAAAAAAAAAAAAPA/AAAAAAAA8D8AAAAAAAAAAAAAAAAAAAAAAAAAAAAAAAAAAAAAAAAAAAAAAAAAAPA/AAAAAAAAAAAAAAAAAAAAAAAAAAAAAPA/AAAAAAAAAAAAAAAAAAAAAAAAAAAAAAAAAAAAAAAA8D8AAAAAAAAAAAAAAAAAAAAAAAAAAAAAAAAAAAAAAAAAAAAAAAAAAAAAAAAAAAAAAAAAAAAAAAAAAAAAAAAAAAAAAAAAAAAAAAAAAAAAAAAAAAAAAAAAAAAAAAAAAAAA8D8AAAAAAAAAAAAAAAAAAAAAAAAAAAAAAAAAAAAAAADwPwAAAAAAAAAAAAAAAAAAAAAAAAAAAAAAAAAAAAAAAAAAAAAAAAAAAAAAAAAAAAAAAAAAAAAAAAAAAAAAAAAAAAAAAAAAAAAAAAAAAAAAAAAAAAAAAAAAAAAAAAAAAAAAAAAAAAAAAAAAAAAAAAAAAAAAAAAAAAAAAAAAAAAAAPA/AAAAAAAAAAAAAAAAAAAAAAAAAAAAAAAAAAAAAAAAAAAAAAAAAADwPwAAAAAAAAAAAAAAAAAAAAAAAAAAAAAAAAAAAAAAAAAAAAAAAAAAAAAAAAAAAADwPwAAAAAAAAAAAAAAAAAAAAAAAAAAAAAAAAAAAAAAAAAAAAAAAAAAAAAAAAAAAAAAAAAAAAAAAAAAAAAAAAAAAAAAAAAAAAAAAAAAAAAAAAAAAAAAAAAAAAAAAAAAAAAAAAAAAAAAAAAAAAAAAAAAAAAAAAAAAAAAAAAAAAAAAAAAAAAAAAAAAAAAAAAAAAAAAAAAAAAAAPA/AAAAAAAAAAAAAAAAAAAAAAAAAAAAAAAAAAAAAAAA8D8AAAAAAAAAAAAAAAAAAAAAAAAAAAAAAAAAAAAAAADwPwAAAAAAAAAAAAAAAAAAAAAAAAAAAAAAAAAAAAAAAAAAAAAAAAAAAAAAAAAAAAAAAAAAAAAAAAAAAAAAAAAAAAAAAAAAAAAAAAAAAAAAAAAAAAAAAAAAAAAAAAAAAAAAAAAAAAAAAPA/AAAAAAAAAAAAAAAAAAAAAAAAAAAAAAAAAAAAAAAAAAAAAAAAAADwPwAAAAAAAAAAAAAAAAAAAAAAAAAAAAAAAAAAAAAAAAAAAAAAAAAAAAAAAAAAAADwPwAAAAAAAAAAAAAAAAAAAAAAAAAAAAAAAAAAAAAAAAAAAAAAAAAAAAAAAAAAAAAAAAAAAAAAAAAAAAAAAAAAAAAAAAAAAAAAAAAAAAAAAAAAAAAAAAAA8D8AAAAAAAAAAAAAAAAAAAAAD28AAACwAQAABgAAAAAAAAAAAAAAAAAAAAAAAAAAAAAAAAAAAAAAAAAAAAAAAAAAAAAAAAAAAAAAAAAAAAAAAAAAAAAAAAAAAAAAAAAAAAAAAAAAAAAAAAAAAAAAAAAAAAAAAAAAAAAAAAAAAAAAAAAAAAAAAAAAAAAAAAAAAAAAAAAAAAAAAAAAAAAAAAAAAAAAAAAAAAAAAAAAAAAAAAAAAAAAAAAAAAAAAAAAAAAAAAAAAAAAAAAAAAAAAAAAAAAAAAAAAAAAAAAAAAAAAAAAAAAAAAAAAAAAAAAAAAAAAAAAAAAAAAAAAAAAAAAAAAAAAAAAAAAAAAAAAAAAAAAAAAAAAAAAAAAAAAAAAADwPwAAAAAAAAAAAAAAAAAAAAAAAAAAAAAAAAAAAAAAAAAAAAAAAAAAAAAAAAAAAAAAAAAAAAAAAAAAAAAAAAAAAAAAAAAAAAAAAAAAAAAAAAAAAAAAAAAAAAAAAAAAAAAAAAAAAAAAAAAAAAAAAAAAAAAAAAAAAAAAAAAAAAAAAAAAAAAAAAAAAAAAAAAAAAAAAAAAAAAAAAAAAAAAAAAAAAAAAAAAAAAAAAAAAAAAAAAAAAAAAAAAAAAAAAAAAAAAAAAAAAAAAAAAAAAAAAAAAAAAAAAAAAAAAAAAAAAAAAAAAAAAAAAAAAAAAAAAAAAAAAAAAAAAAAAAAAAAAAAAAAAAAAAAAAAAAAAAAAAAAAAAAAAAAAAAAAAAAAAAAAAAAAAAAAAAAAAAAAAAAAAA8D8AAAAAAAAAAAAAAAAAAAAAAAAAAAAAAAAAAAAAAAAAAAAAAAAAAAAAAAAAAAAAAAAAAAAAAAAAAAAAAAAAAPA/AAAAAAAAAAAAAAAAAAAAAAAAAAAAAAAAAAAAAAAAAAAAAAAAAAAAAAAAAAAAAAAAAAAAAAAAAAAAAAAAAAAAAAAAAAAAAAAAAAAAAAAAAAAAAAAAAAAAAAAAAAAAAAAAAAAAAAAAAAAAAAAAAAAAAAAAAAAAAAAAAAAAAAAAAAAAAAAAAAAAAAAAAAAAAAAAAAAAAAAAAAAAAAAAAAAAAAAAAAAAAAAAAAAAAAAAAAAAAAAAAAAAAAAAAAAAAAAAAAAAAAAAAAAAAAAAAAAAAAAAAAAAAAAAAAAAAAAAAAAAAAAAAAAAAAAAAAAAAAAAAAAAAAAA8D8AAAAAAAAAAAAAAAAAAAAAAAAAAAAAAAAAAAAAAAAAAAAAAAAAAAAAAAAAAAAAAAAAAAAAAAAAAAAAAAAAAAAAAAAAAAAAAAAAAAAAAAAAAAAAAAAAAAAAAAAAAAAAAAAAAAAAAAAAAAAAAAAAAAAAAAAAAAAAAAAAAAAAAADwPwAAAAAAAAAAAAAAAAAAAAAAAAAAAAAAAAAAAAAAAAAAAAAAAAAAAAAAAAAAAAAAAAAAAAAAAAAAAAAAAAAAAAAAAAAAAAAAAAAAAAAAAAAAAAAAAAAAAAAAAAAAAAAAAAAAAAAAAAAAAAAAAAAAAAAAAAAAAAAAAAAAAAAAAAAAAAAAAAAAAAAAAAAAAAAAAAAAAAAAAAAAAAAAAAAAAAAAAAAAAAAAAAAAAAAAAAAAAAAAAAAAAAAAAAAAAAAAAAAAAAAAAAAAAAAAAAAAAAAAAAAAAAAAAAAAAAAAAAAAAAAAAAAAAAAAAAAAAAAAAAAAAAAAAAAAAAAAAAAAAAAAAAAAAAAAAAAAAAAAAAAAAAAAAAAAAAAAAAAAAAAAAAAAAAAAAAAAAAAAAAAAAAAAAAAAAAAAAAAAAAAAAAAAAAAAAAAAAAAAAAAAAAAAAAAAAAAAAAAAAAAAAAAAAAAAAAAAAAAAAAAAAAAAAAAAAAAAAAAAAAAAAAAAAAAAAAAAAAAAAAAAAAAAAAAAAAAAAAAAAAAAAAAAAAAAAAAAAAAAAAAAAAAAAAAAAAAAAAAAAAAAAAAAAAAAAAAAAAAAAAAAAAAAAAAAAAAAAAAAAAAAAAAAAAAAAAAAAAAAAAAAAAAAAAAAAAAAAAAAAAAAAAAAAAAAAAAAAAAAAAAAAAAAAAAAAAAAAAAAAAAAAAAAAAAAAAAAAAAAAAAAAAAAAAAAAAAAAAAAAAAAAAAAAAAAAAAAAAAAAAAAAAAAAAAAAAAAAAAAAAAAAAAAAAAAAAAAAAAAAAAAAAAAAAAAAAAAAAAAAAAAAAAAAAAAAAAAAAAAAAAAAAAAAAAAAAAAAAAAAAAAAAAAAAAAAAAAAAAAAAAAAAAAAAAAAAAAAAAAAAAAAAAAAAAAAAAAAAAAAAAAAADwPwAAAAAAAAAAAAAAAAAAAAAAAAAAAAAAAAAAAAAAAAAAAAAAAAAAAAAAAAAAAAAAAAAAAAAAAAAAAAAAAAAA8D8AAAAAAAAAAAAAAAAAAAAAAAAAAAAAAAAAAAAAAAAAAAAAAAAAAAAAAAAAAAAAAAAAAAAAAAAAAAAAAAAAAAAAAAAAAAAAAAAAAAAAAADwPwAAAAAAAAAAAAAAAAAAAAAAAAAAAAAAAAAAAAAAAAAAAAAAAAAAAAAAAAAAAAAAAAAAAAAAAAAAAAAAAAAAAAAAAAAAAAAAAAAAAAAAAAAAAAAAAAAAAAAAAAAAAAAAAAAAAAAAAAAAAAAAAAAAAAAAAAAAAAAAAAAAAAAAAAAAAAAAAAAAAAAAAAAAAAAAAAAAAAAAAAAAAAAAAAAAAAAAAAAAAAAAAAAAAAAAAAAAAAAAAAAAAAAAAAAAAAAAAAAAAAAAAAAAAAAAAAAAAAAAAAAAAAAAAAAAAAAAAAAAAAAAAAAAAAAAAAAAAAAAAAAAAAAAAAAAAAAAAAAAAAAAAAAAAAAAAAAAAAAAAAAAAAAAAAAAAAAAAAAAAAAAAAAAAAAAAAAAAAAAAAAAAAAAAAAAAAAAAAAAAAAAAAAAAAAAAAAAAAAAAAAAAAAAAAAAAAAAAAAAAAAAAAAAAAAAAAAAAAAAAAAAAAAAAAAAAAAAAAAAAAAAAAAAAAAAAAAAAAAAAAAAAAAAAAAAAAAAAAAAAAAAAAAAAAAAAAAAAAAAAAAAAAAAAAAAAAAAAAAAAAAAAAAAAAAAAAAAAAAAAAAAAAAAAAAAAAAAAAAAAAAAAAAAAAAAAAAAAAAAAAAAAAAAAAAAAAAAAAAAAAAAAAAAAAAAAAAAAAAAAAAAAAAAAAAAAAAAAAAAAAAAAAAAAAAAAAAAAAAAAAAAAAAAAAAAAAAAAAAAAAAAAAAAAAAAAAAAAAAAAAAAAAAAAAAAAAAAAAAAAAAAAAAAAAAAAAAAAADwPwAAAAAAAAAAAAAAAAAAAAAAAAAAAAAAAAAAAAAAAAAAAAAAAAAAAAAAAAAAAAAAAAAAAAAAAAAAAAAAAAAAAAAAAAAAAAAAAAAAAAAAAAAAAAAAAAAAAAAAAAAAAAAAAAAAAAAAAAAAAAAAAAAAAAAAAAAAAAAAAAAAAAAAAAAAAAAAAAAAAAAAAAAAAAAAAAAAAAAAAAAAAAAAAAAAAAAAAAAAAAAAAAAAAAAAAAAAAAAAAAAAAAAAAAAAAAAAAAAAAAAAAAAAAAAAAAAAAAAAAAAAAAAAAAAAAAAAAAAAAAAAAAAAAAAAAAAAAAAAAAAAAAAAAAAAAAAAAAAA8D8AAAAAAAAAAAAAAAAAAAAAAAAAAAAAAAAAAAAAAAAAAAAAAAAAAPA/AAAAAAAAAAAAAAAAAAAAAAAAAAAAAAAAAAAAAAAAAAAAAAAAAAAAAAAAAAAAAAAAAAAAAAAAAAAAAAAAAAAAAAAAAAAAAAAAAAAAAAAAAAAAAAAAAAAAAAAAAAAAAAAAAAAAAAAAAAAAAAAAAAAAAAAAAAAAAAAAAAAAAAAAAAAAAAAAAAAAAAAAAAAAAAAAAAAAAAAAAAAAAAAAAAAAAAAAAAAAAAAAAAAAAAAAAAAAAAAAAAAAAAAAAAAAAAAAAAAAAAAAAAAAAAAAAAAAAAAAAAAAAAAAAAAAAAAA8D8AAAAAAAAAAAAAAAAAAAAAAAAAAAAAAAAAAAAAAAAAAAAAAAAAAAAAAAAAAAAAAAAAAAAAAAAAAAAAAAAAAAAAAAAAAAAAAAAAAAAAAAAAAAAAAAAAAAAAAAAAAAAAAAAAAAAAAAAAAAAAAAAAAAAAAAAAAAAAAAAAAAAAAAAAAAAAAAAAAAAAAAAAAAAAAAAAAAAAAAAAAAAAAAAAAAAAAAAAAAAAAAAAAAAAAAAAAAAAAAAAAAAAAAAAAAAAAAAAAAAAAAAAAAAAAAAAAAAAAAAAAAAAAAAAAAAAAAAAAAAAAAAAAAAAAAAAAAAAAAAAAAAAAAAAAAAAAAAAAAAAAAAAAAAAAAAAAAAAAAAAAAAAAAAAAPA/AAAAAAAAAAAAAAAAAAAAAAAAAAAAAAAAAAAAAAAAAAAAAAAAAAAAAAAAAAAAAAAAAAAAAAAAAAAAAAAAAAAAAAAAAAAAAAAAAAAAAAAAAAAAAAAAAAAAAAAAAAAAAPA/AAAAAAAAAAAAAAAAAAAAAAAAAAAAAAAAAAAAAAAAAAAAAAAAAAAAAAAAAAAAAAAAAAAAAAAAAAAAAAAAAAAAAAAAAAAAAAAAAAAAAAAAAAAAAAAAAAAAAAAAAAAAAAAAAAAAAAAAAAAAAAAAAAAAAAFyAAAAagAAAAQAAAABdv///3n///9qAAAAAAAAALEBAAAJiAAAAAaMAAAACE1hdGNoaW5nAAABAAAAAXQAAABqAAAAGgAAAAFz////ef///wIAAAAAAAAAsQEAAAmIAAAABo8AAAAITWF0Y2hpbmcAAAAAAAAL <-!redo!!

**Logistic regression**

Warning: Rank changed, consider dropping predictors

Warning: saturated model (all degrees of freedom used, can't assess goodness of fit)

The following observations were dropped due to complete determination of the outcome: 1, 2, 3, 4, 5, 6, 7, 8, 9, 10, 11, 12, 13, 14, 15, 16, 17, 18, 19, 20, 21, 22, 23, 24, 25, 26, 27, 28, 29, 30, 31, 32, 33, 34

| Deviance goodness of fit chi-square = | 0 | df = -10 | P = * |  |
| --- | --- | --- | --- | --- |
| Deviance (likelihood ratio) chi-square = | 62,481692 | df = 43 | P = 0,0276 |  |

| Parameter | Odds Ratio | 95% Conf. Int. | Z Value | P (>\|Z\|) |
| --- | --- | --- | --- | --- |
| (intercept) | n/a |  | 0 | P > 0,9999 |
| Valve or ring size(21) | 1 | (0,140863 to 7,099071) | 0 | P > 0,9999 |
| Valve or ring size(23) | 1 | (0,140863 to 7,099071) | 0 | P > 0,9999 |
| Valve or ring size(25) | 1 | (0,140863 to 7,099071) | 0 | P > 0,9999 |
| Valve or ring size(27) | 1 | (0,140863 to 7,099071) | 0 | P > 0,9999 |
| Cardiac procedures | 1 | (0,140863 to 7,099071) | 0 | P > 0,9999 |
| EF(1) | 1 | (0,140863 to 7,099071) | 0 | P > 0,9999 |
| EF(2) | 1 | (0,140863 to 7,099071) | 0 | P > 0,9999 |
| EF(3) | 1 | (0,140863 to 7,099071) | 0 | P > 0,9999 |
| Gender | 1 | (0,140863 to 7,099071) | 0 | P > 0,9999 |

logit Valve type = 0 +0 Valve or ring size(21) +0 Valve or ring size(23) +0 Valve or ring size(25) +0 Valve or ring size(27) +0 Cardiac procedures +0 EF(1) +0 EF(2) +0 EF(3) +0 Gender

!!help!-> 0 <-!help!! !!redo!-> "LogisticRegressionModel" AAEAAAD/////AQAAAAAAAAAMAgAAAEJTdGF0c0RpcmVjdCwgVmVyc2lvbj0zLjMuNS4wLCBDdWx0dXJlPW5ldXRyYWwsIFB1YmxpY0tleVRva2VuPW51bGwFAQAAACJTdGF0c0RpcmVjdC5UZW1wbGF0ZXMuUGFyYW1ldGVyQmFnAQAAABBmaWxsZWRQYXJhbWV0ZXJzA/EBU3lzdGVtLkNvbGxlY3Rpb25zLkdlbmVyaWMuRGljdGlvbmFyeWAyW1tTeXN0ZW0uU3RyaW5nLCBtc2NvcmxpYiwgVmVyc2lvbj00LjAuMC4wLCBDdWx0dXJlPW5ldXRyYWwsIFB1YmxpY0tleVRva2VuPWI3N2E1YzU2MTkzNGUwODldLFtTdGF0c0RpcmVjdC5UZW1wbGF0ZXMuRmlsbGVkUGFyYW1ldGVyLCBTdGF0c0RpcmVjdCwgVmVyc2lvbj0zLjMuNS4wLCBDdWx0dXJlPW5ldXRyYWwsIFB1YmxpY0tleVRva2VuPW51bGxdXQIAAAAJAwAAAAQDAAAA8QFTeXN0ZW0uQ29sbGVjdGlvbnMuR2VuZXJpYy5EaWN0aW9uYXJ5YDJbW1N5c3RlbS5TdHJpbmcsIG1zY29ybGliLCBWZXJzaW9uPTQuMC4wLjAsIEN1bHR1cmU9bmV1dHJhbCwgUHVibGljS2V5VG9rZW49Yjc3YTVjNTYxOTM0ZTA4OV0sW1N0YXRzRGlyZWN0LlRlbXBsYXRlcy5GaWxsZWRQYXJhbWV0ZXIsIFN0YXRzRGlyZWN0LCBWZXJzaW9uPTMuMy41LjAsIEN1bHR1cmU9bmV1dHJhbCwgUHVibGljS2V5VG9rZW49bnVsbF1dBAAAAAdWZXJzaW9uCENvbXBhcmVyCEhhc2hTaXplDUtleVZhbHVlUGFpcnMAAwADCJIBU3lzdGVtLkNvbGxlY3Rpb25zLkdlbmVyaWMuR2VuZXJpY0VxdWFsaXR5Q29tcGFyZXJgMVtbU3lzdGVtLlN0cmluZywgbXNjb3JsaWIsIFZlcnNpb249NC4wLjAuMCwgQ3VsdHVyZT1uZXV0cmFsLCBQdWJsaWNLZXlUb2tlbj1iNzdhNWM1NjE5MzRlMDg5XV0I9QFTeXN0ZW0uQ29sbGVjdGlvbnMuR2VuZXJpYy5LZXlWYWx1ZVBhaXJgMltbU3lzdGVtLlN0cmluZywgbXNjb3JsaWIsIFZlcnNpb249NC4wLjAuMCwgQ3VsdHVyZT1uZXV0cmFsLCBQdWJsaWNLZXlUb2tlbj1iNzdhNWM1NjE5MzRlMDg5XSxbU3RhdHNEaXJlY3QuVGVtcGxhdGVzLkZpbGxlZFBhcmFtZXRlciwgU3RhdHNEaXJlY3QsIFZlcnNpb249My4zLjUuMCwgQ3VsdHVyZT1uZXV0cmFsLCBQdWJsaWNLZXlUb2tlbj1udWxsXV1bXQoAAAAJBAAAABEAAAAJBQAAAAQEAAAAkgFTeXN0ZW0uQ29sbGVjdGlvbnMuR2VuZXJpYy5HZW5lcmljRXF1YWxpdHlDb21wYXJlcmAxW1tTeXN0ZW0uU3RyaW5nLCBtc2NvcmxpYiwgVmVyc2lvbj00LjAuMC4wLCBDdWx0dXJlPW5ldXRyYWwsIFB1YmxpY0tleVRva2VuPWI3N2E1YzU2MTkzNGUwODldXQAAAAAHBQAAAAABAAAACgAAAAPzAVN5c3RlbS5Db2xsZWN0aW9ucy5HZW5lcmljLktleVZhbHVlUGFpcmAyW1tTeXN0ZW0uU3RyaW5nLCBtc2NvcmxpYiwgVmVyc2lvbj00LjAuMC4wLCBDdWx0dXJlPW5ldXRyYWwsIFB1YmxpY0tleVRva2VuPWI3N2E1YzU2MTkzNGUwODldLFtTdGF0c0RpcmVjdC5UZW1wbGF0ZXMuRmlsbGVkUGFyYW1ldGVyLCBTdGF0c0RpcmVjdCwgVmVyc2lvbj0zLjMuNS4wLCBDdWx0dXJlPW5ldXRyYWwsIFB1YmxpY0tleVRva2VuPW51bGxdXQT6////8wFTeXN0ZW0uQ29sbGVjdGlvbnMuR2VuZXJpYy5LZXlWYWx1ZVBhaXJgMltbU3lzdGVtLlN0cmluZywgbXNjb3JsaWIsIFZlcnNpb249NC4wLjAuMCwgQ3VsdHVyZT1uZXV0cmFsLCBQdWJsaWNLZXlUb2tlbj1iNzdhNWM1NjE5MzRlMDg5XSxbU3RhdHNEaXJlY3QuVGVtcGxhdGVzLkZpbGxlZFBhcmFtZXRlciwgU3RhdHNEaXJlY3QsIFZlcnNpb249My4zLjUuMCwgQ3VsdHVyZT1uZXV0cmFsLCBQdWJsaWNLZXlUb2tlbj1udWxsXV0CAAAAA2tleQV2YWx1ZQEEK1N0YXRzRGlyZWN0LlRlbXBsYXRlcy5GaWxsZWRPYmplY3RQYXJhbWV0ZXICAAAABgcAAAAHY29udGV4dAkIAAAAAff////6////BgoAAAATY2FuZGlkYXRlUHJlZGljdG9ycwkLAAAAAfT////6////Bg0AAAAKcHJlZGljdG9ycwkOAAAAAfH////6////BhAAAAAIcmVzcG9uc2UJEQAAAAHu////+v///wYTAAAACGdyb3VwaW5nCRQAAAAB6/////r///8GFgAAAAhhY2N1cmFjeQkXAAAAAej////6////BhkAAAAJaW50ZXJjZXB0CRoAAAAB5f////r///8GHAAAAAd3ZWlnaHRzCR0AAAAB4v////r///8GHwAAAAVnYW1tYQkgAAAAAd/////6////BiIAAAAac3RhdHNkaXJlY3Qtb3BlcmF0aW9uLWxpc3QJIwAAAAUIAAAAK1N0YXRzRGlyZWN0LlRlbXBsYXRlcy5GaWxsZWRPYmplY3RQYXJhbWV0ZXICAAAAFTxEYXRhPmtfX0JhY2tpbmdGaWVsZCpGaWxsZWRQYXJhbWV0ZXIrPERpcmVjdGlvbj5rX19CYWNraW5nRmllbGQCBC5TdGF0c0RpcmVjdC5UZW1wbGF0ZXMuRmlsbGVkUGFyYW1ldGVyRGlyZWN0aW9uAgAAAAIAAAAJJAAAAAXb////LlN0YXRzRGlyZWN0LlRlbXBsYXRlcy5GaWxsZWRQYXJhbWV0ZXJEaXJlY3Rpb24BAAAAB3ZhbHVlX18ACAIAAAABAAAABQsAAAAuU3RhdHNEaXJlY3QuVGVtcGxhdGVzLkZpbGxlZERhdGFGcmFtZVBhcmFtZXRlcgIAAAAVPERhdGE+a19fQmFja2luZ0ZpZWxkKkZpbGxlZFBhcmFtZXRlcis8RGlyZWN0aW9uPmtfX0JhY2tpbmdGaWVsZAQEGlN0YXRzRGlyZWN0LkRhdGEuRGF0YUZyYW1lAgAAAC5TdGF0c0RpcmVjdC5UZW1wbGF0ZXMuRmlsbGVkUGFyYW1ldGVyRGlyZWN0aW9uAgAAAAIAAAAJJgAAAAHZ////2////wEAAAABDgAAAAsAAAAJKAAAAAHX////2////wEAAAABEQAAAAsAAAAJKgAAAAHV////2////wEAAAAFFAAAACtTdGF0c0RpcmVjdC5UZW1wbGF0ZXMuRmlsbGVkU3RyaW5nUGFyYW1ldGVyAgAAABU8RGF0YT5rX19CYWNraW5nRmllbGQqRmlsbGVkUGFyYW1ldGVyKzxEaXJlY3Rpb24+a19fQmFja2luZ0ZpZWxkAQQuU3RhdHNEaXJlY3QuVGVtcGxhdGVzLkZpbGxlZFBhcmFtZXRlckRpcmVjdGlvbgIAAAACAAAABiwAAAAKaW5kaXZpZHVhbAHT////2////wEAAAABFwAAABQAAAAGLgAAAAkwLjAwMDAwMDEB0f///9v///8BAAAABRoAAAAsU3RhdHNEaXJlY3QuVGVtcGxhdGVzLkZpbGxlZEJvb2xlYW5QYXJhbWV0ZXICAAAAFTxEYXRhPmtfX0JhY2tpbmdGaWVsZCpGaWxsZWRQYXJhbWV0ZXIrPERpcmVjdGlvbj5rX19CYWNraW5nRmllbGQABAEuU3RhdHNEaXJlY3QuVGVtcGxhdGVzLkZpbGxlZFBhcmFtZXRlckRpcmVjdGlvbgIAAAACAAAAAQHQ////2////wEAAAABHQAAABoAAAAAAc/////b////AQAAAAUgAAAAK1N0YXRzRGlyZWN0LlRlbXBsYXRlcy5GaWxsZWREb3VibGVQYXJhbWV0ZXICAAAAFTxEYXRhPmtfX0JhY2tpbmdGaWVsZCpGaWxsZWRQYXJhbWV0ZXIrPERpcmVjdGlvbj5rX19CYWNraW5nRmllbGQABAYuU3RhdHNEaXJlY3QuVGVtcGxhdGVzLkZpbGxlZFBhcmFtZXRlckRpcmVjdGlvbgIAAAACAAAAZmZmZmZm7j8Bzv///9v///8BAAAABSMAAAAvU3RhdHNEaXJlY3QuVGVtcGxhdGVzLkZpbGxlZFN0cmluZ0xpc3RQYXJhbWV0ZXICAAAAFTxEYXRhPmtfX0JhY2tpbmdGaWVsZCpGaWxsZWRQYXJhbWV0ZXIrPERpcmVjdGlvbj5rX19CYWNraW5nRmllbGQDBH9TeXN0ZW0uQ29sbGVjdGlvbnMuR2VuZXJpYy5MaXN0YDFbW1N5c3RlbS5TdHJpbmcsIG1zY29ybGliLCBWZXJzaW9uPTQuMC4wLjAsIEN1bHR1cmU9bmV1dHJhbCwgUHVibGljS2V5VG9rZW49Yjc3YTVjNTYxOTM0ZTA4OV1dLlN0YXRzRGlyZWN0LlRlbXBsYXRlcy5GaWxsZWRQYXJhbWV0ZXJEaXJlY3Rpb24CAAAAAgAAAAkzAAAAAcz////b////AQAAAAUkAAAAPFN0YXRzRGlyZWN0LkJ1aWx0aW5zLlJlZ3Jlc3MrTXVsdGlwbGVMaW5lYXJSZWdyZXNzaW9uQ29udGV4dCgAAAAUPEFyZz5rX19CYWNraW5nRmllbGQSPEI+a19fQmFja2luZ0ZpZWxkGzxDb3ZhcmlhbmNlPmtfX0JhY2tpbmdGaWVsZBQ8REVWPmtfX0JhY2tpbmdGaWVsZBU8REVWWD5rX19CYWNraW5nRmllbGQTPERGPmtfX0JhY2tpbmdGaWVsZBQ8REZYPmtfX0JhY2tpbmdGaWVsZBQ8RG9DPmtfX0JhY2tpbmdGaWVsZBM8RFY+a19fQmFja2luZ0ZpZWxkEjxNPmtfX0JhY2tpbmdGaWVsZBM8RlY+a19fQmFja2luZ0ZpZWxkEzxIMT5rX19CYWNraW5nRmllbGQSPEg+a19fQmFja2luZ0ZpZWxkFzxMYWJlbHM+a19fQmFja2luZ0ZpZWxkFDxMTFg+a19fQmFja2luZ0ZpZWxkEjxOPmtfX0JhY2tpbmdGaWVsZB08T3V0Y29tZVRpdGxlPmtfX0JhY2tpbmdGaWVsZBI8UD5rX19CYWNraW5nRmllbGQSPFI+a19fQmFja2luZ0ZpZWxkEzxSMj5rX19CYWNraW5nRmllbGQVPFJBTks+a19fQmFja2luZ0ZpZWxkEzxSVj5rX19CYWNraW5nRmllbGQUPFJYST5rX19CYWNraW5nRmllbGQSPFM+a19fQmFja2luZ0ZpZWxkEzxTZT5rX19CYWNraW5nRmllbGQWPFNTUkVHPmtfX0JhY2tpbmdGaWVsZBQ8U1NZPmtfX0JhY2tpbmdGaWVsZBM8U1Y+a19fQmFja2luZ0ZpZWxkEjxUPmtfX0JhY2tpbmdGaWVsZBc8VGl0bGVzPmtfX0JhY2tpbmdGaWVsZBQ8VE9MPmtfX0JhY2tpbmdGaWVsZBI8Vj5rX19CYWNraW5nRmllbGQUPFZJRj5rX19CYWNraW5nRmllbGQVPHdhcm4+a19fQmFja2luZ0ZpZWxkFzxXRUlHSFQ+a19fQmFja2luZ0ZpZWxkHDx3ZWlnaHRUaXRsZT5rX19CYWNraW5nRmllbGQTPFdUPmtfX0JhY2tpbmdGaWVsZBI8WD5rX19CYWNraW5nRmllbGQTPFgxPmtfX0JhY2tpbmdGaWVsZBI8WT5rX19CYWNraW5nRmllbGQHBwcAAAAAAAcABwcDBgAAAQAHAwAHBwcHAAAHBwYAAwcBAAEHAwcHBgYGBgYICAEGCAYGEFN5c3RlbS5Eb3VibGVbLF0GCAgGEFN5c3RlbS5Eb3VibGVbLF0IBggGBgYGBgYGEFN5c3RlbS5Eb3VibGVbLF0GAQYQU3lzdGVtLkRvdWJsZVssXQYGAgAAAAoJNQAAAAk2AAAAAAAAAAAAAAApgoUXqD1PQPb///8hAAAAAQoJAAAACTcAAAAJOAAAAAoJOQAAAHgtuHB60XDAIgAAAAoKAAAACToAAAAKCgAAAAoJOwAAAAoJPAAAAAAAAAAAAAAAAAAAAAAAAAAKCT0AAAAKexSuR+F6hD8KCgoBCgk+AAAACT8AAAAKCUAAAAAFJgAAABpTdGF0c0RpcmVjdC5EYXRhLkRhdGFGcmFtZQIAAAAVPE5hbWU+a19fQmFja2luZ0ZpZWxkGjxWYXJpYWJsZXM+a19fQmFja2luZ0ZpZWxkAQODAVN5c3RlbS5Db2xsZWN0aW9ucy5HZW5lcmljLkxpc3RgMVtbU3RhdHNEaXJlY3QuRGF0YS5JVmFyaWFibGUsIFN0YXRzRGlyZWN0LCBWZXJzaW9uPTMuMy41LjAsIEN1bHR1cmU9bmV1dHJhbCwgUHVibGljS2V5VG9rZW49bnVsbF1dAgAAAAoJQQAAAAEoAAAAJgAAAAZCAAAABkRhdGEgMQlDAAAAASoAAAAmAAAABkQAAAAGRGF0YSAxCUUAAAAEMwAAAH9TeXN0ZW0uQ29sbGVjdGlvbnMuR2VuZXJpYy5MaXN0YDFbW1N5c3RlbS5TdHJpbmcsIG1zY29ybGliLCBWZXJzaW9uPTQuMC4wLjAsIEN1bHR1cmU9bmV1dHJhbCwgUHVibGljS2V5VG9rZW49Yjc3YTVjNTYxOTM0ZTA4OV1dAwAAAAZfaXRlbXMFX3NpemUIX3ZlcnNpb24GAAAICAlGAAAAAQAAAAEAAAAPNQAAAAsAAAAGAAAAAAAAAAAAAAAAAAAAAAAAAAAAAAAAAAAAAAAAAAAAAAAAAAAAAAAAAAAAAAAAAAAAAAAAAAAAAAAAAAAAAAAAAAAAAAAAAAAAAAAAAAAAAAAAAAAAAA82AAAAOAAAAAYAAAAAAAAAAAAAAAAAAPA/AAAAAAAAAAAAAAAAAADwPwAAAAAAAAAAAAAAAAAAAAAAAAAAAADwPwAAAAAAAAAAAAAAAAAAAAAAAAAAAAAAAAAAAAAAAPA/AAAAAAAAAAAAAAAAAAAAAAAAAAAAAAAAAAAAAAAAAAAAAAAAAADwPwAAAAAAAAAAAAAAAAAAAAAAAAAAAAAAAAAAAAAAAAAAAAAAAAAAAAAAAAAAAADwPwAAAAAAAAAAAAAAAAAAAAAAAAAAAAAAAAAAAAAAAAAAAAAAAAAAAAAAAAAAAAAAAAAAAAAAAPA/AAAAAAAAAAAAAAAAAAAAAAAAAAAAAAAAAAAAAAAAAAAAAAAAAAAAAAAAAAAAAAAAAAAAAAAAAAAAAAAAAADwPwAAAAAAAAAAAAAAAAAAAAAAAAAAAAAAAAAAAAAAAAAAAAAAAAAAAAAAAAAAAAAAAAAAAAAAAAAAAAAAAAAAAAAAAAAAAADwPwAAAAAAAAAAAAAAAAAAAAAAAAAAAAAAAAAAAAAAAAAAAAAAAAAAAAAAAAAAAAAAAAAAAAAAAAAAAAAAAAAAAAAAAAAAAAAAAAAAAAAAAPA/DzcAAAAjAAAABgAAAAAAAAAAAAAAAAAAAAAAAAAAAAAAAAAAAAAAAAAAAAAAAAAAAAAAAAAAAAAAAAAAAAAAAAAAAAAAAAAAAAAAAAAAAAAAAAAAAAAAAAAAAAAAAAAAAAAAAAAAAAAAAAAAAAAAAAAAAAAAAAAAAAAAAAAAAAAAAAAAAAAAAAAAAAAAAAAAAAAAAAAAAAAAAAAAAAAAAAAAAAAAAAAAAAAAAAAAAAAAAAAAAAAAAAAAAAAAAAAAAAAAAAAAAAAAAAAAAAAAAAAAAAAAAAAAAAAAAAAAAAAAAAAAAAAAAAAAAAAAAAAAAAAAAAAAAAAAAAAAAAAAAAAAAAAAAAAAAAAAAAAAAAAAAAAAAAAAAAAAAAAAAAAAAAAPOAAAACMAAAAGAAAAAAAAAAAAAAAAAAAAAAAAAAAAAAAAAAAAAAAAAAAAAAAAAAAAAAAAAAAAAAAAAAAAAAAAAAAAAAAAAAAAAAAAAAAAAAAAAAAAAAAAAAAAAAAAAAAAAAAAAAAAAAAAAAAAAAAAAAAAAAAAAAAAAAAAAAAAAAAAAAAAAAAAAAAAAAAAAAAAAAAAAAAAAAAAAAAAAAAAAAAAAAAAAAAAAAAAAAAAAAAAAAAAAAAAAAAAAAAAAAAAAAAAAAAAAAAAAAAAAAAAAAAAAAAAAAAAAAAAAAAAAAAAAAAAAAAAAAAAAAAAAAAAAAAAAAAAAAAAAAAAAAAAAAAAAAAAAAAAAAAAAAAAAAAAAAAAAAAAAAAAAAAAAAAAABE5AAAACwAAAAZHAAAAClZhbHZlIHR5cGUGSAAAABZWYWx2ZSBvciByaW5nIHNpemUoMjEpBkkAAAAWVmFsdmUgb3IgcmluZyBzaXplKDIzKQZKAAAAFlZhbHZlIG9yIHJpbmcgc2l6ZSgyNSkGSwAAABZWYWx2ZSBvciByaW5nIHNpemUoMjcpBkwAAAASQ2FyZGlhYyBwcm9jZWR1cmVzBk0AAAAFRUYoMSkGTgAAAAVFRigyKQZPAAAABUVGKDMpBlAAAAAGR2VuZGVyCg86AAAAIwAAAAYAAAAAAAAAAAAAAAAAAAAAAAAAAAAAAAAAAAAAAAAAAAAAAAAAAAAAAAAAAAAAAAAAAAAAAAAAAAAAAAAAAAAAAAAAAAAAAAAAAAAAAAAAAAAAAAAAAAAAAAAAAAAAAAAAAAAAAAAAAAAAAAAAAAAAAAAAAAAAAAAAAAAAAAAAAAAAAAAAAAAAAAAAAAAAAAAAAAAAAAAAAAAAAAAAAAAAAAAAAAAAAAAAAAAAAAAAAAAAAAAAAAAAAAAAAAAAAAAAAAAAAAAAAAAAAAAAAAAAAAAAAAAAAAAAAAAAAAAAAAAAAAAAAAAAAAAAAAAAAAAAAAAAAAAAAAAAAAAAAAAAAAAAAAAAAAAAAAAAAAAAAAAAAAAAAAAADzsAAAACAAAACAAAAAAAAAAADzwAAAAjAAAABgAAAAAAAAAAAAAAAAAA8D8AAAAAAADwPwAAAAAAAPA/AAAAAAAA8D8AAAAAAADwPwAAAAAAAPA/AAAAAAAA8D8AAAAAAADwPwAAAAAAAPA/AAAAAAAA8D8AAAAAAAAAAAAAAAAAAAAAAAAAAAAAAAAAAAAAAAAAAAAAAAAAAAAAAAAAAAAAAAAAAAAAAAAAAAAAAAAAAAAAAAAAAAAAAAAAAAAAAAAAAAAAAAAAAAAAAAAAAAAAAAAAAAAAAAAAAAAAAAAAAAAAAAAAAAAAAAAAAAAAAAAAAAAAAAAAAAAAAAAAAAAAAAAAAAAAAAAAAAAAAAAAAAAAAAAAAAAAAAAAAAAAAAAAAAAAAAAAAAAAAAAAAAAAAAAPPQAAACMAAAAGAAAAAAAAAAAAAAAAAABGQAAAAAAAgEVAAAAAAAAASUAAAAAAAAAkQAAAAAAAAEdAAAAAAAAAMEAAAAAAAAAAQAAAAAAAAC5AAAAAAAAAIkAAAAAAAAAQQAAAAAAAAChAAAAAAAAAREAAAAAAAAAIQAAAAAAAgEtAAAAAAAAAAEAAAAAAAAAsQAAAAAAAAABAAAAAAAAAIkAAAAAAAADwPwAAAAAAAPA/AAAAAAAAHEAAAAAAAAA1QAAAAAAAAPA/AAAAAAAAAEAAAAAAAAAIQAAAAAAAAABAAAAAAAAAAEAAAAAAAAAYQAAAAAAAAAhAAAAAAAAAAEAAAAAAAADwPwAAAAAAAABAAAAAAAAA8D8AAAAAAADwPw8+AAAAIwAAAAYAAAAAAAAAAAAAAAAAAAAAAAAAAAAAAAAAAAAAAAAAAAAAAAAAAAAAAAAAAAAAAAAAAAAAAAAAAAAAAAAAAAAAAAAAAAAAAAAAAAAAAAAAAAAAAAAAAAAAAAAAAAAAAAAAAAAAAAAAAAAAAAAAAAAAAAAAAAAAAAAAAAAAAAAAAAAAAAAAAAAAAAAAAAAAAAAAAAAAAAAAAAAAAAAAAAAAAAAAAAAAAAAAAAAAAAAAAAAAAAAAAAAAAAAAAAAAAAAAAAAAAAAAAAAAAAAAAAAAAAAAAAAAAAAAAAAAAAAAAAAAAAAAAAAAAAAAAAAAAAAAAAAAAAAAAAAAAAAAAAAAAAAAAAAAAAAAAAAAAAAAAAAAAAAAAAAABz8AAAACAgAAACMAAAALAAAAAAYAAAAAAAAAAAAAAAAAAAAAAAAAAAAAAAAAAAAAAAAAAAAAAAAAAAAAAAAAAAAAAAAAAAAAAAAAAAAAAAAAAAAAAAAAAAAAAAAAAAAAAAAAAAAAAAAAAAAAAAAAAAAAAAAAAAAAAADwPwAAAAAAAAAAAAAAAAAAAAAAAAAAAAAAAAAAAAAAAAAAAAAAAAAA8D8AAAAAAAAAAAAAAAAAAAAAAAAAAAAA8D8AAAAAAAAAAAAAAAAAAAAAAAAAAAAAAAAAAAAAAAAAAAAAAAAAAPA/AAAAAAAAAAAAAAAAAADwPwAAAAAAAPA/AAAAAAAAAAAAAAAAAAAAAAAAAAAAAAAAAAAAAAAAAAAAAAAAAAAAAAAAAAAAAAAAAAAAAAAA8D8AAAAAAAAAAAAAAAAAAAAAAAAAAAAA8D8AAAAAAADwPwAAAAAAAAAAAAAAAAAAAAAAAAAAAAAAAAAAAAAAAAAAAAAAAAAAAAAAAAAAAAAAAAAAAAAAAPA/AAAAAAAAAAAAAAAAAAAAAAAAAAAAAPA/AAAAAAAAAAAAAAAAAADwPwAAAAAAAAAAAAAAAAAAAAAAAAAAAAAAAAAAAAAAAAAAAAAAAAAAAAAAAAAAAADwPwAAAAAAAAAAAAAAAAAAAAAAAAAAAAAAAAAAAAAAAPA/AAAAAAAAAAAAAAAAAAAAAAAAAAAAAPA/AAAAAAAAAAAAAAAAAAAAAAAAAAAAAAAAAAAAAAAA8D8AAAAAAAAAAAAAAAAAAAAAAAAAAAAA8D8AAAAAAADwPwAAAAAAAAAAAAAAAAAAAAAAAAAAAADwPwAAAAAAAAAAAAAAAAAAAAAAAAAAAAAAAAAAAAAAAPA/AAAAAAAAAAAAAAAAAAAAAAAAAAAAAPA/AAAAAAAAAAAAAAAAAADwPwAAAAAAAAAAAAAAAAAA8D8AAAAAAAAAAAAAAAAAAAAAAAAAAAAA8D8AAAAAAAAAAAAAAAAAAAAAAAAAAAAAAAAAAAAAAADwPwAAAAAAAPA/AAAAAAAAAAAAAAAAAAAAAAAAAAAAAPA/AAAAAAAAAAAAAAAAAAAAAAAAAAAAAAAAAAAAAAAA8D8AAAAAAAAAAAAAAAAAAAAAAAAAAAAAAAAAAAAAAAAAAAAAAAAAAPA/AAAAAAAAAAAAAAAAAAAAAAAAAAAAAAAAAAAAAAAAAAAAAAAAAADwPwAAAAAAAAAAAAAAAAAAAAAAAAAAAAAAAAAAAAAAAAAAAAAAAAAA8D8AAAAAAAAAAAAAAAAAAAAAAAAAAAAAAAAAAAAAAAAAAAAAAAAAAAAAAAAAAAAAAAAAAAAAAAAAAAAAAAAAAPA/AAAAAAAAAAAAAAAAAADwPwAAAAAAAAAAAAAAAAAA8D8AAAAAAAAAAAAAAAAAAAAAAAAAAAAAAAAAAAAAAAAAAAAAAAAAAAAAAAAAAAAA8D8AAAAAAAAAAAAAAAAAAAAAAAAAAAAAAAAAAAAAAADwPwAAAAAAAAAAAAAAAAAAAAAAAAAAAAAAAAAAAAAAAAAAAAAAAAAAAAAAAAAAAAAAAAAAAAAAAAAAAAAAAAAA8D8AAAAAAAAAAAAAAAAAAPA/AAAAAAAAAAAAAAAAAAAAAAAAAAAAAPA/AAAAAAAAAAAAAAAAAAAAAAAAAAAAAAAAAAAAAAAAAAAAAAAAAAAAAAAAAAAAAPA/AAAAAAAAAAAAAAAAAAAAAAAAAAAAAPA/AAAAAAAAAAAAAAAAAAAAAAAAAAAAAAAAAAAAAAAAAAAAAAAAAAAAAAAAAAAAAPA/AAAAAAAAAAAAAAAAAAAAAAAAAAAAAAAAAAAAAAAA8D8AAAAAAADwPwAAAAAAAAAAAAAAAAAAAAAAAAAAAAAAAAAAAAAAAAAAAAAAAAAAAAAAAAAAAAAAAAAAAAAAAAAAAAAAAAAA8D8AAAAAAAAAAAAAAAAAAAAAAAAAAAAA8D8AAAAAAAAAAAAAAAAAAAAAAAAAAAAA8D8AAAAAAAAAAAAAAAAAAAAAAAAAAAAAAAAAAAAAAADwPwAAAAAAAAAAAAAAAAAAAAAAAAAAAAAAAAAAAAAAAAAAAAAAAAAA8D8AAAAAAAAAAAAAAAAAAPA/AAAAAAAAAAAAAAAAAAAAAAAAAAAAAAAAAAAAAAAAAAAAAAAAAADwPwAAAAAAAAAAAAAAAAAAAAAAAAAAAAAAAAAAAAAAAPA/AAAAAAAAAAAAAAAAAAAAAAAAAAAAAAAAAAAAAAAAAAAAAAAAAADwPwAAAAAAAAAAAAAAAAAAAAAAAAAAAAAAAAAAAAAAAAAAAAAAAAAAAAAAAAAAAAAAAAAAAAAAAPA/AAAAAAAA8D8AAAAAAAAAAAAAAAAAAAAAAAAAAAAA8D8AAAAAAAAAAAAAAAAAAAAAAAAAAAAAAAAAAAAAAAAAAAAAAAAAAAAAAAAAAAAAAAAAAAAAAADwPwAAAAAAAAAAAAAAAAAAAAAAAAAAAAAAAAAAAAAAAAAAAAAAAAAAAAAAAAAAAAAAAAAAAAAAAPA/AAAAAAAAAAAAAAAAAAAAAAAAAAAAAPA/AAAAAAAAAAAAAAAAAAAAAAAAAAAAAAAAAAAAAAAAAAAAAAAAAAAAAAAAAAAAAAAAAAAAAAAAAAAAAAAAAADwPwAAAAAAAAAAAAAAAAAA8D8AAAAAAAAAAAAAAAAAAAAAAAAAAAAAAAAAAAAAAAAAAAAAAAAAAAAAAAAAAAAAAAAAAAAAAADwPwAAAAAAAAAAAAAAAAAAAAAAAAAAAAAAAAAAAAAAAAAAAAAAAAAAAAAAAAAAAADwPwAAAAAAAAAAAAAAAAAAAAAAAAAAAAAAAAAAAAAAAPA/AAAAAAAAAAAAAAAAAAAAAAAAAAAAAAAAAAAAAAAA8D8AAAAAAAAAAAAAAAAAAPA/AAAAAAAAAAAAAAAAAADwPwAAAAAAAAAAAAAAAAAAAAAAAAAAAADwPwAAAAAAAAAAAAAAAAAAAAAAAAAAAAAAAAAAAAAAAAAAAAAAAAAAAAAAAAAAAADwPwAAAAAAAAAAAAAAAAAA8D8AAAAAAAAAAAAAAAAAAAAAAAAAAAAAAAAAAAAAAAAAAAAAAAAAAPA/AAAAAAAAAAAAAAAAAAAAAAAAAAAAAAAAAAAAAAAAAAAAAAAAAADwPwAAAAAAAAAAAAAAAAAAAAAAAAAAAAAAAAAAAAAAAAAAAAAAAAAAAAAAAAAAAAAAAAAAAAAAAPA/AAAAAAAA8D8AAAAAAAAAAAAAAAAAAAAAAAAAAAAA8D8AAAAAAAAAAAAAAAAAAAAAAAAAAAAAAAAAAAAAAAAAAAAAAAAAAAAAAAAAAAAAAAAAAAAAAADwPwAAAAAAAPA/AAAAAAAAAAAAAAAAAADwPwAAAAAAAAAAAAAAAAAAAAAAAAAAAAAAAAAAAAAAAAAAAAAAAAAAAAAAAAAAAAAAAAAAAAAAAAAAAAAAAAAA8D8AAAAAAAAAAAAAAAAAAAAAAAAAAAAAAAAAAAAAAADwPwAAAAAAAAAAAAAAAAAAAAAAAAAAAAAAAAAAAAAAAAAAAAAAAAAAAAAAAAAAAADwPwAAAAAAAAAAAAAAAAAA8D8AAAAAAADwPwAAAAAAAAAAAAAAAAAAAAAAAAAAAADwPwAAAAAAAAAAAAAAAAAAAAAAAAAAAADwPwAAAAAAAAAAAAAAAAAAAAAAAAAAAAAAAAAAAAAAAAAAAAAAAAAAAAAAAAAAAADwPwAAAAAAAAAAAAAAAAAAAAAAAAAAAAAAAAAAAAAAAAAAAAAAAAAAAAAAAAAAAAAAAAAAAAAAAAAAAAAAAAAA8D8AAAAAAAAAAAAAAAAAAPA/AAAAAAAAAAAAAAAAAAAAAAAAAAAAAPA/AAAAAAAAAAAAAAAAAAAAAAAAAAAAAAAAAAAAAAAA8D8AAAAAAAAAAAAAAAAAAAAAAAAAAAAA8D8AAAAAAAAAAAAAAAAAAAAAAAAAAAAA8D8AAAAAAADwPwAAAAAAAAAAAAAAAAAAAAAAAAAAAADwPwAAAAAAAAAAAAAAAAAAAAAAAAAAAAAAAAAAAAAAAPA/AAAAAAAAAAAAAAAAAADwPwAAAAAAAAAAAAAAAAAAAAAAAAAAAAAAAA9AAAAAIwAAAAYAAAAAAAAAAAAAAAAAAChAAAAAAAAALkAAAAAAAAAiQAAAAAAAAABAAAAAAAAALkAAAAAAAAAgQAAAAAAAAKA8AAAAAAAAFEAAAAAAAACgPAAAAAAAAAhAAAAAAAAAEEAAAAAAAAAiQAAAAAAAAPA/AAAAAAAAKEAAAAAAAACgPAAAAAAAACRAAAAAAAAAoDwAAAAAAAAQQAAAAAAAAKA8AAAAAAAAoDwAAAAAAAAAQAAAAAAAACRAAAAAAAAAoDwAAAAAAACgPAAAAAAAAPA/AAAAAAAA8D8AAAAAAADwPwAAAAAAAAhAAAAAAAAAAEAAAAAAAAAAQP///////+8/AAAAAAAAAED////////vP////////+8/BEEAAACDAVN5c3RlbS5Db2xsZWN0aW9ucy5HZW5lcmljLkxpc3RgMVtbU3RhdHNEaXJlY3QuRGF0YS5JVmFyaWFibGUsIFN0YXRzRGlyZWN0LCBWZXJzaW9uPTMuMy41LjAsIEN1bHR1cmU9bmV1dHJhbCwgUHVibGljS2V5VG9rZW49bnVsbF1dAwAAAAZfaXRlbXMFX3NpemUIX3ZlcnNpb24EAAAcU3RhdHNEaXJlY3QuRGF0YS5JVmFyaWFibGVbXQIAAAAICAlRAAAAAwAAAAMAAAABQwAAAEEAAAAJUgAAAAkAAAAJAAAAAUUAAABBAAAACVMAAAABAAAAAQAAABFGAAAABAAAAAZUAAAAEkxvZ2lzdGljUmVncmVzc2lvbg0DB1EAAAAAAQAAAAQAAAAEGlN0YXRzRGlyZWN0LkRhdGEuSVZhcmlhYmxlAgAAAAlVAAAACVYAAAAJVwAAAAoHUgAAAAABAAAAEAAAAAQaU3RhdHNEaXJlY3QuRGF0YS5JVmFyaWFibGUCAAAACVgAAAAJWQAAAAlaAAAACVsAAAAJXAAAAAldAAAACV4AAAAJXwAAAAlgAAAADQcHUwAAAAABAAAABAAAAAQaU3RhdHNEaXJlY3QuRGF0YS5JVmFyaWFibGUCAAAACWEAAAANAwVVAAAAH1N0YXRzRGlyZWN0LkRhdGEuU3RyaW5nVmFyaWFibGUDAAAAFkdlbmVyaWNWYXJpYWJsZWAxK2RhdGEoR2VuZXJpY1ZhcmlhYmxlYDErPFRpdGxlPmtfX0JhY2tpbmdGaWVsZClHZW5lcmljVmFyaWFibGVgMSs8T3JpZ2luPmtfX0JhY2tpbmdGaWVsZAYBBBhTdGF0c0RpcmVjdC5EYXRhLklPcmlnaW4CAAAAAgAAAAliAAAABmMAAAAETmFtZQoBVgAAAFUAAAAJZAAAAAZlAAAABVZhbHVlCgVXAAAAH1N0YXRzRGlyZWN0LkRhdGEuRG91YmxlVmFyaWFibGUHAAAAA3N1bQNtaW4DbWF4DGhhc1N1bW1hcmllcxZHZW5lcmljVmFyaWFibGVgMStkYXRhKEdlbmVyaWNWYXJpYWJsZWAxKzxUaXRsZT5rX19CYWNraW5nRmllbGQpR2VuZXJpY1ZhcmlhYmxlYDErPE9yaWdpbj5rX19CYWNraW5nRmllbGQAAAAABwEEBgYGAQYYU3RhdHNEaXJlY3QuRGF0YS5JT3JpZ2luAgAAAAIAAAAAAAAAAAAAAAAAAAAAAAAAAAAAAAAAAAAACWYAAAAGZwAAAAlPbGQgdmFsdWUKAVgAAABXAAAAAAAAAAAAAAAAAAAAAAAAAAAAAAAAAAAAAAloAAAACUgAAAAKAVkAAABXAAAAAAAAAAAAAAAAAAAAAAAAAAAAAAAAAAAAAAlqAAAACUkAAAAKAVoAAABXAAAAAAAAAAAAAAAAAAAAAAAAAAAAAAAAAAAAAAlsAAAACUoAAAAKAVsAAABXAAAAAAAAAAAAAAAAAAAAAAAAAAAAAAAAAAAAAAluAAAACUsAAAAKAVwAAABXAAAAAAAAAAAAAAAAAAAAAAAAAAAAAAAAAAAAAAoJTAAAAAlxAAAAAV0AAABXAAAAAAAAAAAAAAAAAAAAAAAAAAAAAAAAAAAAAAlyAAAACU0AAAAKAV4AAABXAAAAAAAAAAAAAAAAAAAAAAAAAAAAAAAAAAAAAAl0AAAACU4AAAAKAV8AAABXAAAAAAAAAAAAAAAAAAAAAAAAAAAAAAAAAAAAAAl2AAAACU8AAAAKAWAAAABXAAAAAAAAAAAAAAAAAAAAAAAAAAAAAAAAAAAAAAoJUAAAAAl5AAAAAWEAAABXAAAAAAAAAAAAAAAAAAAAAAAAAAAAAAAAAAAAAAoJRwAAAAl7AAAAEWIAAAAJAAAACUgAAAAJSQAAAAlKAAAACUsAAAAJTAAAAAlNAAAACU4AAAAJTwAAAAlQAAAAEWQAAAAJAAAABoUAAAADMCwyBoYAAAADMCwyBocAAAADMCwyBogAAAADMCwyBokAAAADMCw1BooAAAAEMCwyNQaLAAAABDAsMjUGjAAAAAQwLDI1Bo0AAAADMCw1D2YAAAAJAAAABpqZmZmZmck/mpmZmZmZyT+amZmZmZnJP5qZmZmZmck/AAAAAAAA4D8AAAAAAADQPwAAAAAAANA/AAAAAAAA0D8AAAAAAADgPw9oAAAAsAEAAAYAAAAAAADwPwAAAAAAAPA/AAAAAAAAAAAAAAAAAAAAAAAAAAAAAAAAAAAAAAAAAAAAAAAAAAAAAAAAAAAAAAAAAAAAAAAAAAAAAAAAAAAAAAAAAAAAAAAAAAAAAAAA8D8AAAAAAAAAAAAAAAAAAAAAAAAAAAAA8D8AAAAAAAAAAAAAAAAAAAAAAAAAAAAAAAAAAAAAAAAAAAAAAAAAAAAAAAAAAAAA8D8AAAAAAADwPwAAAAAAAAAAAAAAAAAAAAAAAAAAAAAAAAAAAAAAAAAAAAAAAAAA8D8AAAAAAAAAAAAAAAAAAAAAAAAAAAAAAAAAAAAAAAAAAAAAAAAAAAAAAAAAAAAAAAAAAAAAAAAAAAAAAAAAAAAAAAAAAAAA8D8AAAAAAAAAAAAAAAAAAPA/AAAAAAAAAAAAAAAAAADwPwAAAAAAAAAAAAAAAAAAAAAAAAAAAAAAAAAAAAAAAAAAAAAAAAAAAAAAAAAAAAAAAAAAAAAAAAAAAAAAAAAAAAAAAAAAAAAAAAAAAAAAAAAAAAAAAAAA8D8AAAAAAAAAAAAAAAAAAPA/AAAAAAAAAAAAAAAAAAAAAAAAAAAAAAAAAAAAAAAA8D8AAAAAAAAAAAAAAAAAAPA/AAAAAAAAAAAAAAAAAAAAAAAAAAAAAAAAAAAAAAAAAAAAAAAAAADwPwAAAAAAAAAAAAAAAAAAAAAAAAAAAAAAAAAAAAAAAAAAAAAAAAAAAAAAAAAAAAAAAAAAAAAAAPA/AAAAAAAAAAAAAAAAAADwPwAAAAAAAAAAAAAAAAAAAAAAAAAAAAAAAAAAAAAAAAAAAAAAAAAAAAAAAAAAAADwPwAAAAAAAAAAAAAAAAAAAAAAAAAAAADwPwAAAAAAAAAAAAAAAAAAAAAAAAAAAAAAAAAAAAAAAAAAAAAAAAAAAAAAAAAAAAAAAAAAAAAAAAAAAAAAAAAA8D8AAAAAAAAAAAAAAAAAAAAAAAAAAAAAAAAAAAAAAADwPwAAAAAAAAAAAAAAAAAAAAAAAAAAAAAAAAAAAAAAAAAAAAAAAAAAAAAAAAAAAAAAAAAAAAAAAAAAAAAAAAAAAAAAAAAAAAAAAAAAAAAAAAAAAAAAAAAAAAAAAAAAAAAAAAAAAAAAAAAAAAAAAAAAAAAAAAAAAADwPwAAAAAAAAAAAAAAAAAAAAAAAAAAAAAAAAAAAAAAAAAAAAAAAAAAAAAAAAAAAAAAAAAAAAAAAAAAAAAAAAAAAAAAAAAAAAAAAAAAAAAAAAAAAAAAAAAAAAAAAAAAAAAAAAAAAAAAAAAAAAAAAAAAAAAAAAAAAADwPwAAAAAAAAAAAAAAAAAAAAAAAAAAAAAAAAAAAAAAAAAAAAAAAAAAAAAAAAAAAAAAAAAAAAAAAAAAAAAAAAAA8D8AAAAAAAAAAAAAAAAAAAAAAAAAAAAAAAAAAAAAAAAAAAAAAAAAAAAAAAAAAAAAAAAAAAAAAAAAAAAAAAAAAAAAAAAAAAAAAAAAAAAAAAAAAAAAAAAAAAAAAAAAAAAAAAAAAAAAAAAAAAAAAAAAAPA/AAAAAAAAAAAAAAAAAADwPwAAAAAAAAAAAAAAAAAA8D8AAAAAAADwPwAAAAAAAAAAAAAAAAAA8D8AAAAAAADwPwAAAAAAAAAAAAAAAAAAAAAAAAAAAAAAAAAAAAAAAAAAAAAAAAAAAAAAAAAAAAAAAAAAAAAAAPA/AAAAAAAAAAAAAAAAAAAAAAAAAAAAAAAAAAAAAAAA8D8AAAAAAADwPwAAAAAAAAAAAAAAAAAAAAAAAAAAAAAAAAAAAAAAAAAAAAAAAAAAAAAAAAAAAADwPwAAAAAAAAAAAAAAAAAAAAAAAAAAAAAAAAAAAAAAAAAAAAAAAAAA8D8AAAAAAAAAAAAAAAAAAAAAAAAAAAAAAAAAAAAAAAAAAAAAAAAAAAAAAAAAAAAA8D8AAAAAAAAAAAAAAAAAAAAAAAAAAAAAAAAAAAAAAAAAAAAAAAAAAAAAAAAAAAAAAAAAAAAAAAAAAAAAAAAAAAAAAAAAAAAAAAAAAAAAAAAAAAAAAAAAAAAAAAAAAAAAAAAAAAAAAAAAAAAAAAAAAAAAAAAAAAAAAAAAAAAAAAAAAAAAAAAAAAAAAAAAAAAAAAAAAAAAAAAAAAAAAAAAAAAAAAAAAAAAAAAAAAAAAAAAAAAAAAAAAAAAAAAAAAAAAAAAAAAAAAAAAAAAAAAAAPA/AAAAAAAAAAAAAAAAAADwPwAAAAAAAAAAAAAAAAAAAAAAAAAAAAAAAAAAAAAAAAAAAAAAAAAAAAAAAAAAAAAAAAAAAAAAAAAAAAAAAAAAAAAAAAAAAADwPwAAAAAAAAAAAAAAAAAAAAAAAAAAAADwPwAAAAAAAAAAAAAAAAAAAAAAAAAAAAAAAAAAAAAAAAAAAAAAAAAAAAAAAAAAAADwPwAAAAAAAAAAAAAAAAAAAAAAAAAAAAAAAAAAAAAAAAAAAAAAAAAAAAAAAAAAAAAAAAAAAAAAAAAAAAAAAAAAAAAAAAAAAAAAAAAAAAAAAAAAAAAAAAAAAAAAAAAAAAAAAAAAAAAAAAAAAAAAAAAA8D8AAAAAAAAAAAAAAAAAAAAAAAAAAAAAAAAAAAAAAADwPwAAAAAAAAAAAAAAAAAA8D8AAAAAAAAAAAAAAAAAAAAAAAAAAAAA8D8AAAAAAAAAAAAAAAAAAAAAAAAAAAAAAAAAAAAAAAAAAAAAAAAAAAAAAAAAAAAAAAAAAAAAAADwPwAAAAAAAAAAAAAAAAAA8D8AAAAAAAAAAAAAAAAAAAAAAAAAAAAAAAAAAAAAAAAAAAAAAAAAAAAAAAAAAAAAAAAAAAAAAAAAAAAAAAAAAAAAAAAAAAAA8D8AAAAAAAAAAAAAAAAAAAAAAAAAAAAAAAAAAAAAAAAAAAAAAAAAAAAAAAAAAAAAAAAAAAAAAAAAAAAAAAAAAPA/AAAAAAAA8D8AAAAAAAAAAAAAAAAAAAAAAAAAAAAAAAAAAAAAAAAAAAAAAAAAAAAAAAAAAAAAAAAAAAAAAAAAAAAAAAAAAAAAAAAAAAAA8D8AAAAAAAAAAAAAAAAAAAAAAAAAAAAAAAAAAAAAAAAAAAAAAAAAAAAAAAAAAAAAAAAAAAAAAAAAAAAAAAAAAAAAAAAAAAAA8D8AAAAAAAAAAAAAAAAAAPA/AAAAAAAAAAAAAAAAAAAAAAAAAAAAAAAAAAAAAAAAAAAAAAAAAAAAAAAAAAAAAAAAAAAAAAAAAAAAAAAAAAAAAAAAAAAAAAAAAAAAAAAAAAAAAAAAAAAAAAAAAAAAAAAAAAAAAAAA8D8AAAAAAAAAAAAAAAAAAAAAAAAAAAAAAAAAAAAAAAAAAAAAAAAAAPA/AAAAAAAAAAAAAAAAAAAAAAAAAAAAAAAAAAAAAAAAAAAAAAAAAAAAAAAAAAAAAAAAAAAAAAAA8D8AAAAAAAAAAAAAAAAAAAAAAAAAAAAAAAAAAAAAAAAAAAAAAAAAAPA/AAAAAAAAAAAAAAAAAAAAAAAAAAAAAAAAAAAAAAAAAAAAAAAAAAAAAAAAAAAAAAAAAAAAAAAAAAAAAAAAAAAAAAAAAAAAAAAAAAAAAAAAAAAAAAAAAAAAAAAAAAAAAAAAAAAAAAAAAAAAAAAAAAAAAAAAAAAAAAAAAAAAAAAAAAAAAAAAAAAAAAAAAAAAAAAAAAAAAAAAAAAAAAAAAAAAAAAAAAAAAAAAAAAAAAAAAAAAAAAAAAAAAAAAAAAAAAAAAAAAAAAAAAAAAAAAAAAAAAAAAAAAAAAAAAAAAAAAAAAAAAAAAAAAAAAAAAAAAAAAAAAAAAAAAAAAAAAAAAAAAAAAAAAAAAAAAAAAAAAAAAAAAAAAAAAAAAAAAAAAAAAAAAAAAAAA8D8AAAAAAAAAAAAAAAAAAPA/AAAAAAAAAAAAAAAAAAAAAAAAAAAAAAAAAAAAAAAAAAAAAAAAAAAAAAAAAAAAAAAAAAAAAAAAAAAAAAAAAAAAAAAAAAAAAAAAAAAAAAAAAAAAAAAAAAAAAAAAAAAAAAAAAAAAAAAAAAAAAAAAAAAAAAAAAAAAAAAAAAAAAAAA8D8AAAAAAAAAAAAAAAAAAAAAAAAAAAAAAAAAAAAAAAAAAAAAAAAAAAAAAAAAAAAAAAAAAAAAAAAAAAAAAAAAAPA/AAAAAAAAAAAAAAAAAAAAAAAAAAAAAAAAAAAAAAAAAAAAAAAAAADwPwAAAAAAAAAAAAAAAAAAAAAAAAAAAAAAAAAAAAAAAAAAAAAAAAAA8D8AAAAAAADwPwAAAAAAAAAAAAAAAAAA8D8AAAAAAAAAAAAAAAAAAAAAAAAAAAAA8D8AAAAAAAAAAAAAAAAAAAAAAAAAAAAAAAAAAAAAAAAAAAAAAAAAAAAAAAAAAAAA8D8AAAAAAAAAAAAAAAAAAAAAAAAAAAAA8D8AAAAAAAAAAAAAAAAAAAAAAAAAAAAAAAAAAAAAAADwPwAAAAAAAAAAAAAAAAAAAAAAAAAAAADwPwAAAAAAAPA/AAAAAAAAAAAAAAAAAADwPwAAAAAAAPA/AAAAAAAAAAAAAAAAAAAAAAAAAAAAAAAAAAAAAAAA8D8PagAAALABAAAGAAAAAAAAAAAAAAAAAAAAAAAAAAAAAAAAAAAAAAAAAAAAAAAAAADwPwAAAAAAAPA/AAAAAAAA8D8AAAAAAAAAAAAAAAAAAPA/AAAAAAAA8D8AAAAAAADwPwAAAAAAAAAAAAAAAAAA8D8AAAAAAADwPwAAAAAAAAAAAAAAAAAA8D8AAAAAAAAAAAAAAAAAAPA/AAAAAAAA8D8AAAAAAADwPwAAAAAAAAAAAAAAAAAAAAAAAAAAAADwPwAAAAAAAPA/AAAAAAAA8D8AAAAAAADwPwAAAAAAAAAAAAAAAAAA8D8AAAAAAADwPwAAAAAAAPA/AAAAAAAA8D8AAAAAAADwPwAAAAAAAAAAAAAAAAAA8D8AAAAAAADwPwAAAAAAAAAAAAAAAAAA8D8AAAAAAAAAAAAAAAAAAPA/AAAAAAAAAAAAAAAAAADwPwAAAAAAAAAAAAAAAAAA8D8AAAAAAADwPwAAAAAAAPA/AAAAAAAA8D8AAAAAAADwPwAAAAAAAPA/AAAAAAAAAAAAAAAAAADwPwAAAAAAAAAAAAAAAAAAAAAAAAAAAAAAAAAAAAAAAAAAAAAAAAAA8D8AAAAAAADwPwAAAAAAAAAAAAAAAAAA8D8AAAAAAAAAAAAAAAAAAAAAAAAAAAAAAAAAAAAAAADwPwAAAAAAAPA/AAAAAAAAAAAAAAAAAADwPwAAAAAAAAAAAAAAAAAA8D8AAAAAAAAAAAAAAAAAAAAAAAAAAAAA8D8AAAAAAAAAAAAAAAAAAAAAAAAAAAAAAAAAAAAAAADwPwAAAAAAAAAAAAAAAAAAAAAAAAAAAAAAAAAAAAAAAPA/AAAAAAAAAAAAAAAAAAAAAAAAAAAAAAAAAAAAAAAAAAAAAAAAAADwPwAAAAAAAPA/AAAAAAAAAAAAAAAAAAAAAAAAAAAAAAAAAAAAAAAA8D8AAAAAAADwPwAAAAAAAAAAAAAAAAAA8D8AAAAAAADwPwAAAAAAAPA/AAAAAAAAAAAAAAAAAAAAAAAAAAAAAPA/AAAAAAAAAAAAAAAAAADwPwAAAAAAAAAAAAAAAAAA8D8AAAAAAAAAAAAAAAAAAAAAAAAAAAAAAAAAAAAAAAAAAAAAAAAAAPA/AAAAAAAA8D8AAAAAAADwPwAAAAAAAAAAAAAAAAAAAAAAAAAAAAAAAAAAAAAAAAAAAAAAAAAAAAAAAAAAAADwPwAAAAAAAPA/AAAAAAAA8D8AAAAAAAAAAAAAAAAAAAAAAAAAAAAAAAAAAAAAAAAAAAAAAAAAAPA/AAAAAAAAAAAAAAAAAADwPwAAAAAAAPA/AAAAAAAAAAAAAAAAAAAAAAAAAAAAAPA/AAAAAAAAAAAAAAAAAADwPwAAAAAAAPA/AAAAAAAAAAAAAAAAAAAAAAAAAAAAAAAAAAAAAAAAAAAAAAAAAAAAAAAAAAAAAPA/AAAAAAAAAAAAAAAAAAAAAAAAAAAAAPA/AAAAAAAAAAAAAAAAAAAAAAAAAAAAAAAAAAAAAAAA8D8AAAAAAAAAAAAAAAAAAAAAAAAAAAAAAAAAAAAAAAAAAAAAAAAAAPA/AAAAAAAAAAAAAAAAAAAAAAAAAAAAAAAAAAAAAAAAAAAAAAAAAADwPwAAAAAAAAAAAAAAAAAAAAAAAAAAAAAAAAAAAAAAAPA/AAAAAAAA8D8AAAAAAADwPwAAAAAAAAAAAAAAAAAA8D8AAAAAAAAAAAAAAAAAAPA/AAAAAAAAAAAAAAAAAAAAAAAAAAAAAAAAAAAAAAAAAAAAAAAAAAAAAAAAAAAAAPA/AAAAAAAAAAAAAAAAAAAAAAAAAAAAAAAAAAAAAAAAAAAAAAAAAAAAAAAAAAAAAPA/AAAAAAAAAAAAAAAAAAAAAAAAAAAAAAAAAAAAAAAAAAAAAAAAAADwPwAAAAAAAAAAAAAAAAAAAAAAAAAAAADwPwAAAAAAAAAAAAAAAAAAAAAAAAAAAADwPwAAAAAAAPA/AAAAAAAA8D8AAAAAAADwPwAAAAAAAAAAAAAAAAAA8D8AAAAAAAAAAAAAAAAAAPA/AAAAAAAAAAAAAAAAAAAAAAAAAAAAAPA/AAAAAAAA8D8AAAAAAAAAAAAAAAAAAPA/AAAAAAAAAAAAAAAAAADwPwAAAAAAAAAAAAAAAAAAAAAAAAAAAADwPwAAAAAAAAAAAAAAAAAA8D8AAAAAAAAAAAAAAAAAAAAAAAAAAAAA8D8AAAAAAAAAAAAAAAAAAPA/AAAAAAAAAAAAAAAAAAAAAAAAAAAAAAAAAAAAAAAA8D8AAAAAAADwPwAAAAAAAPA/AAAAAAAA8D8AAAAAAADwPwAAAAAAAPA/AAAAAAAAAAAAAAAAAAAAAAAAAAAAAPA/AAAAAAAAAAAAAAAAAAAAAAAAAAAAAAAAAAAAAAAAAAAAAAAAAAAAAAAAAAAAAPA/AAAAAAAAAAAAAAAAAADwPwAAAAAAAAAAAAAAAAAAAAAAAAAAAAAAAAAAAAAAAPA/AAAAAAAA8D8AAAAAAADwPwAAAAAAAAAAAAAAAAAAAAAAAAAAAADwPwAAAAAAAPA/AAAAAAAA8D8AAAAAAAAAAAAAAAAAAAAAAAAAAAAAAAAAAAAAAAAAAAAAAAAAAAAAAAAAAAAAAAAAAAAAAADwPwAAAAAAAAAAAAAAAAAA8D8AAAAAAAAAAAAAAAAAAAAAAAAAAAAAAAAAAAAAAAAAAAAAAAAAAAAAAAAAAAAA8D8AAAAAAADwPwAAAAAAAAAAAAAAAAAAAAAAAAAAAAAAAAAAAAAAAAAAAAAAAAAA8D8AAAAAAAAAAAAAAAAAAPA/AAAAAAAA8D8AAAAAAAAAAAAAAAAAAAAAAAAAAAAA8D8AAAAAAADwPwAAAAAAAAAAAAAAAAAA8D8AAAAAAADwPwAAAAAAAAAAAAAAAAAA8D8AAAAAAADwPwAAAAAAAPA/AAAAAAAAAAAAAAAAAAAAAAAAAAAAAAAAAAAAAAAAAAAAAAAAAADwPwAAAAAAAPA/AAAAAAAA8D8AAAAAAADwPwAAAAAAAPA/AAAAAAAA8D8AAAAAAADwPwAAAAAAAAAAAAAAAAAAAAAAAAAAAADwPwAAAAAAAPA/AAAAAAAAAAAAAAAAAADwPwAAAAAAAAAAAAAAAAAAAAAAAAAAAADwPwAAAAAAAAAAAAAAAAAAAAAAAAAAAAAAAAAAAAAAAAAAAAAAAAAA8D8AAAAAAADwPwAAAAAAAPA/AAAAAAAAAAAAAAAAAADwPwAAAAAAAAAAAAAAAAAAAAAAAAAAAAAAAAAAAAAAAAAAAAAAAAAAAAAAAAAAAADwPwAAAAAAAAAAAAAAAAAAAAAAAAAAAADwPwAAAAAAAPA/AAAAAAAAAAAAAAAAAAAAAAAAAAAAAPA/AAAAAAAAAAAAAAAAAAAAAAAAAAAAAAAAAAAAAAAA8D8AAAAAAAAAAAAAAAAAAAAAAAAAAAAAAAAAAAAAAAAAAAAAAAAAAAAAAAAAAAAA8D8AAAAAAAAAAAAAAAAAAPA/AAAAAAAAAAAAAAAAAADwPwAAAAAAAAAAAAAAAAAA8D8AAAAAAAAAAAAAAAAAAAAAAAAAAAAAAAAAAAAAAADwPwAAAAAAAPA/AAAAAAAAAAAAAAAAAAAAAAAAAAAAAPA/AAAAAAAAAAAAAAAAAAAAAAAAAAAAAAAAAAAAAAAAAAAAAAAAAADwPwAAAAAAAPA/AAAAAAAAAAAAAAAAAADwPwAAAAAAAAAAAAAAAAAAAAAAAAAAAADwPwAAAAAAAAAAAAAAAAAA8D8AAAAAAAAAAAAAAAAAAAAAAAAAAAAAAAAAAAAAAADwPwAAAAAAAAAAAAAAAAAAAAAAAAAAAAAAAAAAAAAAAAAAAAAAAAAA8D8AAAAAAADwPwAAAAAAAAAAAAAAAAAAAAAAAAAAAAAAAAAAAAAAAAAAAAAAAAAAAAAAAAAAAAAAAAAAAAAAAAAAAAAAAAAA8D8AAAAAAAAAAAAAAAAAAPA/AAAAAAAAAAAAAAAAAADwPwAAAAAAAAAAAAAAAAAAAAAAAAAAAADwPwAAAAAAAPA/AAAAAAAAAAAAAAAAAAAAAAAAAAAAAAAAAAAAAAAA8D8AAAAAAAAAAAAAAAAAAAAAAAAAAAAAAAAAAAAAAAAAAAAAAAAAAPA/AAAAAAAAAAAAAAAAAAAAAAAAAAAAAAAAAAAAAAAA8D8AAAAAAAAAAAAAAAAAAAAAAAAAAAAAAAAAAAAAAADwPwAAAAAAAPA/AAAAAAAA8D8AAAAAAADwPwAAAAAAAAAAAAAAAAAAAAAAAAAAAADwPwAAAAAAAAAAAAAAAAAAAAAAAAAAAADwPwAAAAAAAAAAAAAAAAAA8D8AAAAAAADwPwAAAAAAAAAAAAAAAAAAAAAAAAAAAAAAAAAAAAAAAAAAAAAAAAAA8D8AAAAAAAAAAAAAAAAAAAAAAAAAAAAAAAAAAAAAAADwPwAAAAAAAPA/AAAAAAAAAAAAAAAAAADwPwAAAAAAAAAAAAAAAAAAAAAAAAAAAAAAAAAAAAAAAAAAAAAAAAAAAAAAAAAAAAAAAAAAAAAAAAAAAAAAAAAAAAAAAAAAAAAAAAAAAAAAAAAAD2wAAACwAQAABgAAAAAAAAAAAAAAAAAAAAAAAAAAAADwPwAAAAAAAPA/AAAAAAAAAAAAAAAAAAAAAAAAAAAAAAAAAAAAAAAA8D8AAAAAAAAAAAAAAAAAAAAAAAAAAAAAAAAAAAAAAAAAAAAAAAAAAAAAAAAAAAAAAAAAAAAAAAAAAAAAAAAAAAAAAAAAAAAA8D8AAAAAAAAAAAAAAAAAAAAAAAAAAAAAAAAAAAAAAAAAAAAAAAAAAAAAAAAAAAAAAAAAAAAAAAAAAAAAAAAAAAAAAAAAAAAAAAAAAAAAAAAAAAAAAAAAAAAAAAAAAAAAAAAAAAAAAAAAAAAAAAAAAAAAAAAAAAAAAAAAAAAAAADwPwAAAAAAAAAAAAAAAAAAAAAAAAAAAAAAAAAAAAAAAAAAAAAAAAAAAAAAAAAAAAAAAAAAAAAAAAAAAAAAAAAAAAAAAAAAAADwPwAAAAAAAAAAAAAAAAAAAAAAAAAAAAAAAAAAAAAAAAAAAAAAAAAAAAAAAAAAAAAAAAAAAAAAAPA/AAAAAAAAAAAAAAAAAAAAAAAAAAAAAPA/AAAAAAAAAAAAAAAAAADwPwAAAAAAAAAAAAAAAAAAAAAAAAAAAAAAAAAAAAAAAAAAAAAAAAAAAAAAAAAAAADwPwAAAAAAAPA/AAAAAAAAAAAAAAAAAAAAAAAAAAAAAAAAAAAAAAAAAAAAAAAAAADwPwAAAAAAAAAAAAAAAAAA8D8AAAAAAADwPwAAAAAAAAAAAAAAAAAAAAAAAAAAAADwPwAAAAAAAAAAAAAAAAAAAAAAAAAAAADwPwAAAAAAAPA/AAAAAAAA8D8AAAAAAAAAAAAAAAAAAAAAAAAAAAAAAAAAAAAAAADwPwAAAAAAAAAAAAAAAAAAAAAAAAAAAAAAAAAAAAAAAPA/AAAAAAAA8D8AAAAAAAAAAAAAAAAAAAAAAAAAAAAAAAAAAAAAAAAAAAAAAAAAAAAAAAAAAAAAAAAAAAAAAAAAAAAAAAAAAAAAAAAAAAAA8D8AAAAAAAAAAAAAAAAAAPA/AAAAAAAAAAAAAAAAAADwPwAAAAAAAAAAAAAAAAAA8D8AAAAAAADwPwAAAAAAAPA/AAAAAAAA8D8AAAAAAAAAAAAAAAAAAAAAAAAAAAAAAAAAAAAAAAAAAAAAAAAAAAAAAAAAAAAA8D8AAAAAAAAAAAAAAAAAAPA/AAAAAAAAAAAAAAAAAAAAAAAAAAAAAAAAAAAAAAAA8D8AAAAAAADwPwAAAAAAAPA/AAAAAAAA8D8AAAAAAAAAAAAAAAAAAPA/AAAAAAAAAAAAAAAAAAAAAAAAAAAAAAAAAAAAAAAA8D8AAAAAAAAAAAAAAAAAAPA/AAAAAAAAAAAAAAAAAAAAAAAAAAAAAAAAAAAAAAAA8D8AAAAAAAAAAAAAAAAAAAAAAAAAAAAAAAAAAAAAAAAAAAAAAAAAAPA/AAAAAAAA8D8AAAAAAAAAAAAAAAAAAPA/AAAAAAAA8D8AAAAAAAAAAAAAAAAAAAAAAAAAAAAA8D8AAAAAAADwPwAAAAAAAPA/AAAAAAAAAAAAAAAAAAAAAAAAAAAAAAAAAAAAAAAA8D8AAAAAAAAAAAAAAAAAAAAAAAAAAAAAAAAAAAAAAAAAAAAAAAAAAAAAAAAAAAAA8D8AAAAAAAAAAAAAAAAAAAAAAAAAAAAAAAAAAAAAAADwPwAAAAAAAAAAAAAAAAAAAAAAAAAAAAAAAAAAAAAAAPA/AAAAAAAA8D8AAAAAAAAAAAAAAAAAAAAAAAAAAAAA8D8AAAAAAAAAAAAAAAAAAAAAAAAAAAAA8D8AAAAAAAAAAAAAAAAAAAAAAAAAAAAA8D8AAAAAAAAAAAAAAAAAAPA/AAAAAAAA8D8AAAAAAAAAAAAAAAAAAPA/AAAAAAAAAAAAAAAAAADwPwAAAAAAAPA/AAAAAAAAAAAAAAAAAAAAAAAAAAAAAPA/AAAAAAAAAAAAAAAAAAAAAAAAAAAAAAAAAAAAAAAAAAAAAAAAAAAAAAAAAAAAAAAAAAAAAAAAAAAAAAAAAAAAAAAAAAAAAPA/AAAAAAAA8D8AAAAAAAAAAAAAAAAAAAAAAAAAAAAA8D8AAAAAAAAAAAAAAAAAAPA/AAAAAAAAAAAAAAAAAADwPwAAAAAAAPA/AAAAAAAAAAAAAAAAAADwPwAAAAAAAAAAAAAAAAAA8D8AAAAAAADwPwAAAAAAAAAAAAAAAAAAAAAAAAAAAAAAAAAAAAAAAAAAAAAAAAAA8D8AAAAAAADwPwAAAAAAAAAAAAAAAAAAAAAAAAAAAAAAAAAAAAAAAAAAAAAAAAAAAAAAAAAAAAAAAAAAAAAAAAAAAAAAAAAAAAAAAAAAAAAAAAAAAAAAAAAAAAAAAAAA8D8AAAAAAAAAAAAAAAAAAPA/AAAAAAAA8D8AAAAAAAAAAAAAAAAAAAAAAAAAAAAAAAAAAAAAAAAAAAAAAAAAAPA/AAAAAAAAAAAAAAAAAAAAAAAAAAAAAAAAAAAAAAAAAAAAAAAAAAAAAAAAAAAAAPA/AAAAAAAAAAAAAAAAAAAAAAAAAAAAAAAAAAAAAAAAAAAAAAAAAAAAAAAAAAAAAPA/AAAAAAAA8D8AAAAAAADwPwAAAAAAAAAAAAAAAAAAAAAAAAAAAAAAAAAAAAAAAAAAAAAAAAAA8D8AAAAAAAAAAAAAAAAAAPA/AAAAAAAA8D8AAAAAAADwPwAAAAAAAAAAAAAAAAAAAAAAAAAAAADwPwAAAAAAAAAAAAAAAAAAAAAAAAAAAAAAAAAAAAAAAAAAAAAAAAAA8D8AAAAAAAAAAAAAAAAAAAAAAAAAAAAA8D8AAAAAAADwPwAAAAAAAAAAAAAAAAAAAAAAAAAAAAAAAAAAAAAAAAAAAAAAAAAAAAAAAAAAAAAAAAAAAAAAAAAAAAAAAAAAAAAAAAAAAAAAAAAAAAAAAPA/AAAAAAAAAAAAAAAAAAAAAAAAAAAAAAAAAAAAAAAAAAAAAAAAAAAAAAAAAAAAAAAAAAAAAAAAAAAAAAAAAAAAAAAAAAAAAAAAAAAAAAAAAAAAAAAAAAAAAAAAAAAAAPA/AAAAAAAAAAAAAAAAAAAAAAAAAAAAAPA/AAAAAAAAAAAAAAAAAADwPwAAAAAAAPA/AAAAAAAAAAAAAAAAAAAAAAAAAAAAAPA/AAAAAAAAAAAAAAAAAADwPwAAAAAAAAAAAAAAAAAAAAAAAAAAAAAAAAAAAAAAAPA/AAAAAAAAAAAAAAAAAADwPwAAAAAAAPA/AAAAAAAA8D8AAAAAAADwPwAAAAAAAPA/AAAAAAAAAAAAAAAAAAAAAAAAAAAAAPA/AAAAAAAAAAAAAAAAAAAAAAAAAAAAAPA/AAAAAAAAAAAAAAAAAAAAAAAAAAAAAAAAAAAAAAAA8D8AAAAAAAAAAAAAAAAAAAAAAAAAAAAA8D8AAAAAAAAAAAAAAAAAAPA/AAAAAAAA8D8AAAAAAADwPwAAAAAAAAAAAAAAAAAAAAAAAAAAAAAAAAAAAAAAAAAAAAAAAAAAAAAAAAAAAAAAAAAAAAAAAAAAAAAAAAAA8D8AAAAAAADwPwAAAAAAAPA/AAAAAAAAAAAAAAAAAAAAAAAAAAAAAAAAAAAAAAAA8D8AAAAAAAAAAAAAAAAAAAAAAAAAAAAAAAAAAAAAAADwPwAAAAAAAPA/AAAAAAAAAAAAAAAAAAAAAAAAAAAAAPA/AAAAAAAAAAAAAAAAAADwPwAAAAAAAPA/AAAAAAAAAAAAAAAAAADwPwAAAAAAAAAAAAAAAAAAAAAAAAAAAAAAAAAAAAAAAPA/AAAAAAAAAAAAAAAAAAAAAAAAAAAAAPA/AAAAAAAAAAAAAAAAAADwPwAAAAAAAAAAAAAAAAAAAAAAAAAAAAAAAAAAAAAAAPA/AAAAAAAAAAAAAAAAAADwPwAAAAAAAPA/AAAAAAAAAAAAAAAAAADwPwAAAAAAAAAAAAAAAAAAAAAAAAAAAAAAAAAAAAAAAPA/AAAAAAAAAAAAAAAAAAAAAAAAAAAAAPA/AAAAAAAAAAAAAAAAAAAAAAAAAAAAAPA/AAAAAAAA8D8AAAAAAAAAAAAAAAAAAAAAAAAAAAAA8D8AAAAAAADwPwAAAAAAAPA/AAAAAAAAAAAAAAAAAAAAAAAAAAAAAAAAAAAAAAAAAAAAAAAAAADwPwAAAAAAAAAAAAAAAAAA8D8AAAAAAADwPwAAAAAAAAAAAAAAAAAAAAAAAAAAAAAAAAAAAAAAAAAAAAAAAAAAAAAAAAAAAAAAAAAAAAAAAAAAAAAAAAAAAAAAAAAAAAAAAAAAAAAAAPA/AAAAAAAAAAAAAAAAAAAAAAAAAAAAAAAAAAAAAAAAAAAAAAAAAAAAAAAAAAAAAPA/AAAAAAAAAAAAAAAAAAAAAAAAAAAAAAAAAAAAAAAA8D8AAAAAAAAAAAAAAAAAAAAAAAAAAAAAAAAAAAAAAAAAAAAAAAAAAAAAAAAAAAAAAAAAAAAAAADwPwAAAAAAAAAAAAAAAAAAAAAAAAAAAADwPwAAAAAAAAAAAAAAAAAAAAAAAAAAAADwPwAAAAAAAPA/AAAAAAAAAAAAAAAAAAAAAA9uAAAAsAEAAAYAAAAAAAAAAAAAAAAAAAAAAAAAAAAAAAAAAAAAAAAAAAAAAAAAAAAAAAAAAAAAAAAAAAAAAAAAAAAAAAAAAAAAAAAAAAAAAAAAAAAAAAAAAAAAAAAAAAAAAAAAAAAAAAAAAAAAAAAAAAAAAAAAAAAAAAAAAAAAAAAAAAAAAAAAAAAAAAAAAAAAAAAAAAAAAAAAAAAAAAAAAAAAAAAAAAAAAAAAAAAAAAAAAAAAAAAAAAAAAAAAAAAAAAAAAAAAAAAAAAAAAAAAAAAAAAAAAAAAAAAAAAAAAAAAAAAAAAAAAAAAAAAAAAAAAAAAAAAAAAAAAAAAAAAAAAAAAAAAAAAAAAAAAAAAAAAAAAAAAAAAAAAAAAAAAAAAAAAAAAAAAAAAAAAAAAAAAAAAAAAAAAAAAAAAAAAAAAAAAAAAAAAAAAAAAAAAAAAAAAAAAAAAAAAAAAAAAAAAAAAAAAAAAAAAAAAAAAAAAAAAAAAAAAAAAAAAAAAAAAAAAAAAAAAAAAAAAAAAAAAAAAAAAAAAAAAAAAAAAAAAAAAAAAAAAAAAAAAAAAAAAAAAAAAAAAAAAAAAAAAAAAAAAAAAAAAAAAAAAAAAAAAAAAAAAAAAAAAAAAAAAAAAAAAAAAAAAAAAAAAAAAAAAAAAAAAAAAAAAAAAAAAAAAAAAAAAAAAAAAAAAAAAAAAAAAAAAAAAAAAAAAAAAAAAAAAAAAAAAAAAAAAAAAAAAAAAAAAAAAAAAAAAAAAAAAAAAAAAAAAAAAAAAAAAAAAAAAAAAAAAAAAAAAAAAAAAAAAAAAAAAAAAAAAAAAAAAAAAAAAAAAAAAAAAAAAAAAAAAAAAAAAAAAAAAPA/AAAAAAAAAAAAAAAAAAAAAAAAAAAAAAAAAAAAAAAAAAAAAAAAAAAAAAAAAAAAAAAAAAAAAAAA8D8AAAAAAAAAAAAAAAAAAAAAAAAAAAAAAAAAAAAAAAAAAAAAAAAAAAAAAAAAAAAAAAAAAAAAAAAAAAAAAAAAAAAAAAAAAAAAAAAAAAAAAAAAAAAAAAAAAAAAAAAAAAAAAAAAAAAAAAAAAAAAAAAAAAAAAAAAAAAAAAAAAAAAAAAAAAAAAAAAAAAAAAAAAAAAAAAAAAAAAAAAAAAAAAAAAAAAAAAAAAAA8D8AAAAAAAAAAAAAAAAAAAAAAAAAAAAA8D8AAAAAAAAAAAAAAAAAAAAAAAAAAAAAAAAAAAAAAAAAAAAAAAAAAAAAAAAAAAAAAAAAAAAAAAAAAAAAAAAAAAAAAAAAAAAAAAAAAAAAAAAAAAAAAAAAAAAAAAAAAAAAAAAAAAAAAAAAAAAAAAAAAAAAAAAAAAAAAAAAAAAAAAAAAAAAAAAAAAAAAAAAAAAAAAAAAAAAAADwPwAAAAAAAAAAAAAAAAAAAAAAAAAAAADwPwAAAAAAAPA/AAAAAAAAAAAAAAAAAAAAAAAAAAAAAAAAAAAAAAAAAAAAAAAAAAAAAAAAAAAAAAAAAAAAAAAA8D8AAAAAAAAAAAAAAAAAAAAAAAAAAAAAAAAAAAAAAAAAAAAAAAAAAAAAAAAAAAAAAAAAAAAAAAAAAAAAAAAAAAAAAAAAAAAAAAAAAAAAAAAAAAAAAAAAAAAAAAAAAAAAAAAAAAAAAAAAAAAAAAAAAAAAAAAAAAAAAAAAAAAAAAAAAAAAAAAAAAAAAAAAAAAAAAAAAAAAAAAAAAAAAAAAAAAAAAAAAAAAAAAAAAAAAAAAAAAAAAAAAAAAAAAAAAAAAAAAAAAAAAAAAAAAAAAAAAAAAAAAAAAAAAAAAAAAAADwPwAAAAAAAAAAAAAAAAAA8D8AAAAAAAAAAAAAAAAAAAAAAAAAAAAAAAAAAAAAAAAAAAAAAAAAAAAAAAAAAAAAAAAAAAAAAAAAAAAAAAAAAAAAAAAAAAAAAAAAAAAAAAAAAAAAAAAAAAAAAAAAAAAAAAAAAAAAAAAAAAAAAAAAAAAAAAAAAAAAAAAAAAAAAAAAAAAAAAAAAAAAAAAAAAAA8D8AAAAAAAAAAAAAAAAAAPA/AAAAAAAAAAAAAAAAAAAAAAAAAAAAAAAAAAAAAAAAAAAAAAAAAAAAAAAAAAAAAAAAAAAAAAAAAAAAAAAAAAAAAAAAAAAAAAAAAAAAAAAAAAAAAAAAAAAAAAAAAAAAAAAAAAAAAAAAAAAAAAAAAAAAAAAAAAAAAAAAAAAAAAAAAAAAAAAAAAAAAAAAAAAAAAAAAAAAAAAAAAAAAAAAAAAAAAAAAAAAAAAAAAAAAAAAAAAAAAAAAAAAAAAAAAAAAAAAAAAAAAAAAAAAAAAAAAAAAAAAAAAAAAAAAAAAAAAAAAAAAAAAAAAAAAAAAAAAAPA/AAAAAAAAAAAAAAAAAAAAAAAAAAAAAAAAAAAAAAAA8D8AAAAAAAAAAAAAAAAAAAAAAAAAAAAAAAAAAAAAAAAAAAAAAAAAAAAAAAAAAAAA8D8AAAAAAAAAAAAAAAAAAPA/AAAAAAAAAAAAAAAAAAAAAAAAAAAAAAAAAAAAAAAA8D8AAAAAAAAAAAAAAAAAAAAAAAAAAAAAAAAAAAAAAAAAAAAAAAAAAPA/AAAAAAAAAAAAAAAAAAAAAAAAAAAAAAAAAAAAAAAAAAAAAAAAAAAAAAAAAAAAAAAAAAAAAAAAAAAAAAAAAAAAAAAAAAAAAAAAAAAAAAAAAAAAAAAAAAAAAAAAAAAAAAAAAAAAAAAAAAAAAAAAAAAAAAAAAAAAAAAAAAAAAAAAAAAAAAAAAAAAAAAAAAAAAPA/AAAAAAAAAAAAAAAAAAAAAAAAAAAAAAAAAAAAAAAAAAAAAAAAAAAAAAAAAAAAAAAAAAAAAAAAAAAAAAAAAAAAAAAAAAAAAAAAAAAAAAAAAAAAAAAAAAAAAAAAAAAAAAAAAAAAAAAA8D8AAAAAAAAAAAAAAAAAAAAAAAAAAAAAAAAAAAAAAAAAAAAAAAAAAAAAAAAAAAAAAAAAAAAAAADwPwAAAAAAAAAAAAAAAAAAAAAAAAAAAAAAAAAAAAAAAAAAAAAAAAAAAAAAAAAAAAAAAAAAAAAAAAAAAAAAAAAAAAAAAAAAAAAAAAAAAAAAAAAAAAAAAAAAAAAAAAAAAAAAAAAAAAAAAAAAAAAAAAAAAAAAAAAAAAAAAAAAAAAAAAAAAAAAAAAAAAAAAAAAAAAAAAAAAAAAAAAAAAAAAAAAAAAAAAAAAAAAAAAAAAAAAAAAAAAAAAAAAAAAAAAAAAAAAAAAAAAAAAAAAAAAAAAAAAAAAAAAAAAAAAAAAAAAAAAAAAAAAAAAAAAAAAAAAAAAAAAAAAAAAAAAAAAAAAAAAAAAAAAAAAAAAAAAAAAAAAAAAAAAAAAAAAAAAAAAAAAAAAAAAAAAAAAAAAAAAAAAAAAAAAAAAADwPwAAAAAAAAAAAAAAAAAA8D8AAAAAAAAAAAAAAAAAAAAAAAAAAAAAAAAAAAAAAAAAAAAAAAAAAAAAAAAAAAAAAAAAAAAAAAAAAAAAAAAAAAAAAAAAAAAAAAAAAAAAAADwPwAAAAAAAAAAAAAAAAAA8D8AAAAAAAAAAAAAAAAAAAAAAAAAAAAAAAAAAAAAAAAAAAAAAAAAAAAAAAAAAAAAAAAAAAAAAADwPwAAAAAAAAAAAAAAAAAAAAAAAAAAAADwPwAAAAAAAPA/AAAAAAAAAAAAAAAAAAAAAAAAAAAAAAAAAAAAAAAAAAAAAAAAAAAAAAAAAAAAAAAAAAAAAAAAAAAAAAAAAAAAAAAAAAAAAAAAAAAAAAAAAAAAAAAAAAAAAAAAAAAAAPA/AAAAAAAA8D8AAAAAAAAAAAAAAAAAAAAAAAAAAAAA8D8AAAAAAAAAAAAAAAAAAPA/AAAAAAAAAAAAAAAAAAAAAAAAAAAAAAAAAAAAAAAAAAAAAAAAAAAAAAAAAAAAAAAAAAAAAAAAAAAAAAAAAAAAAAAAAAAAAPA/AAAAAAAAAAAAAAAAAAAAAAAAAAAAAPA/AAAAAAAAAAAAAAAAAAAAAAAAAAAAAAAAAAAAAAAA8D8AAAAAAAAAAAAAAAAAAAAAAAAAAAAAAAAAAAAAAAAAAAAAAAAAAAAAAAAAAAAAAAAAAAAAAAAAAAAAAAAAAAAAAAAAAAAAAAAAAAAAAAAAAAAAAAAAAPA/AAAAAAAAAAAAAAAAAADwPwAAAAAAAAAAAAAAAAAAAAAAAAAAAAAAAAAAAAAAAAAAAAAAAAAAAAAAAAAAAAAAAAAAAAAAAAAAAAAAAAAAAAAAAAAAAAAAAAAAAAAAAAAAAAAAAAAAAAAAAAAAAAAAAAAAAAAAAAAAAAAAAAAAAAAAAAAAAAAAAAAAAAAAAAAAAAAAAAAAAAAAAAAAAAAAAAAAAAAAAAAAAAAAAAAA8D8AAAAAAAAAAAAAAAAAAPA/AAAAAAAAAAAAAAAAAAAAAAAAAAAAAAAAAAAAAAAAAAAAAAAAAADwPwAAAAAAAAAAAAAAAAAAAAAAAAAAAAAAAAAAAAAAAAAAAAAAAAAAAAAAAAAAAAAAAAAAAAAAAAAAAAAAAAAAAAAAAAAAAAAAAAAAAAAAAAAAAAAAAAAAAAAAAAAAAAAAAAAAAAAAAPA/AAAAAAAAAAAFcQAAACBTdGF0c0RpcmVjdC5EYXRhLldvcmtzaGVldE9yaWdpbgkAAAAXPENvbHVtbj5rX19CYWNraW5nRmllbGQVPE1vZGU+a19fQmFja2luZ0ZpZWxkFzxUb3BSb3c+a19fQmFja2luZ0ZpZWxkFTxSb3dzPmtfX0JhY2tpbmdGaWVsZB08V29ya2Jvb2tQYXRoPmtfX0JhY2tpbmdGaWVsZB48V29ya3NoZWV0TmFtZT5rX19CYWNraW5nRmllbGQZPEhhc1RpdGxlPmtfX0JhY2tpbmdGaWVsZBw8V2FzRmlsdGVyZWQ+a19fQmFja2luZ0ZpZWxkHDxPcmlnaW5Hcm91cD5rX19CYWNraW5nRmllbGQABAAAAQEAAAAIKVN0YXRzRGlyZWN0LlV0aWxpdGllcy5EYXRhQWNxdWlzaXRpb25Nb2RlAgAAAAgIAQEIAgAAABwAAAAFcv///ylTdGF0c0RpcmVjdC5VdGlsaXRpZXMuRGF0YUFjcXVpc2l0aW9uTW9kZQEAAAAHdmFsdWVfXwAIAgAAAGoAAAAAAAAAsQEAAAaPAAAALEM6XFVzZXJzXGhhbnNcQXBwRGF0YVxMb2NhbFxUZW1wXH5FeGNlbC54bHN4BpAAAAAITWF0Y2hpbmcAAAEAAAAPcgAAALABAAAGAAAAAAAA8D8AAAAAAADwPwAAAAAAAPA/AAAAAAAA8D8AAAAAAADwPwAAAAAAAAAAAAAAAAAA8D8AAAAAAADwPwAAAAAAAPA/AAAAAAAA8D8AAAAAAAAAAAAAAAAAAPA/AAAAAAAAAAAAAAAAAADwPwAAAAAAAPA/AAAAAAAA8D8AAAAAAAAAAAAAAAAAAAAAAAAAAAAA8D8AAAAAAAAAAAAAAAAAAPA/AAAAAAAA8D8AAAAAAADwPwAAAAAAAPA/AAAAAAAA8D8AAAAAAAAAAAAAAAAAAPA/AAAAAAAAAAAAAAAAAADwPwAAAAAAAAAAAAAAAAAA8D8AAAAAAADwPwAAAAAAAAAAAAAAAAAA8D8AAAAAAADwPwAAAAAAAPA/AAAAAAAA8D8AAAAAAADwPwAAAAAAAPA/AAAAAAAA8D8AAAAAAADwPwAAAAAAAPA/AAAAAAAA8D8AAAAAAADwPwAAAAAAAPA/AAAAAAAA8D8AAAAAAAAAAAAAAAAAAPA/AAAAAAAA8D8AAAAAAADwPwAAAAAAAPA/AAAAAAAA8D8AAAAAAADwPwAAAAAAAPA/AAAAAAAA8D8AAAAAAADwPwAAAAAAAPA/AAAAAAAA8D8AAAAAAADwPwAAAAAAAPA/AAAAAAAA8D8AAAAAAAAAAAAAAAAAAPA/AAAAAAAA8D8AAAAAAADwPwAAAAAAAAAAAAAAAAAA8D8AAAAAAADwPwAAAAAAAAAAAAAAAAAA8D8AAAAAAAAAAAAAAAAAAPA/AAAAAAAA8D8AAAAAAADwPwAAAAAAAPA/AAAAAAAA8D8AAAAAAADwPwAAAAAAAPA/AAAAAAAAAAAAAAAAAAAAAAAAAAAAAPA/AAAAAAAA8D8AAAAAAADwPwAAAAAAAPA/AAAAAAAAAAAAAAAAAADwPwAAAAAAAPA/AAAAAAAA8D8AAAAAAADwPwAAAAAAAPA/AAAAAAAA8D8AAAAAAADwPwAAAAAAAPA/AAAAAAAA8D8AAAAAAADwPwAAAAAAAAAAAAAAAAAA8D8AAAAAAADwPwAAAAAAAPA/AAAAAAAA8D8AAAAAAADwPwAAAAAAAPA/AAAAAAAA8D8AAAAAAADwPwAAAAAAAPA/AAAAAAAA8D8AAAAAAADwPwAAAAAAAPA/AAAAAAAA8D8AAAAAAAAAAAAAAAAAAAAAAAAAAAAA8D8AAAAAAAAAAAAAAAAAAPA/AAAAAAAAAAAAAAAAAADwPwAAAAAAAAAAAAAAAAAA8D8AAAAAAADwPwAAAAAAAPA/AAAAAAAA8D8AAAAAAADwPwAAAAAAAPA/AAAAAAAA8D8AAAAAAADwPwAAAAAAAAAAAAAAAAAA8D8AAAAAAADwPwAAAAAAAAAAAAAAAAAA8D8AAAAAAADwPwAAAAAAAPA/AAAAAAAAAAAAAAAAAAAAAAAAAAAAAPA/AAAAAAAAAAAAAAAAAADwPwAAAAAAAPA/AAAAAAAA8D8AAAAAAAAAAAAAAAAAAPA/AAAAAAAAAAAAAAAAAADwPwAAAAAAAPA/AAAAAAAA8D8AAAAAAADwPwAAAAAAAPA/AAAAAAAA8D8AAAAAAADwPwAAAAAAAAAAAAAAAAAA8D8AAAAAAADwPwAAAAAAAPA/AAAAAAAA8D8AAAAAAAAAAAAAAAAAAPA/AAAAAAAA8D8AAAAAAADwPwAAAAAAAPA/AAAAAAAA8D8AAAAAAADwPwAAAAAAAPA/AAAAAAAA8D8AAAAAAADwPwAAAAAAAPA/AAAAAAAA8D8AAAAAAADwPwAAAAAAAPA/AAAAAAAA8D8AAAAAAADwPwAAAAAAAPA/AAAAAAAA8D8AAAAAAADwPwAAAAAAAPA/AAAAAAAA8D8AAAAAAADwPwAAAAAAAAAAAAAAAAAA8D8AAAAAAADwPwAAAAAAAPA/AAAAAAAA8D8AAAAAAADwPwAAAAAAAPA/AAAAAAAA8D8AAAAAAADwPwAAAAAAAPA/AAAAAAAA8D8AAAAAAADwPwAAAAAAAPA/AAAAAAAA8D8AAAAAAADwPwAAAAAAAPA/AAAAAAAA8D8AAAAAAADwPwAAAAAAAPA/AAAAAAAA8D8AAAAAAADwPwAAAAAAAPA/AAAAAAAA8D8AAAAAAADwPwAAAAAAAPA/AAAAAAAA8D8AAAAAAADwPwAAAAAAAPA/AAAAAAAA8D8AAAAAAADwPwAAAAAAAPA/AAAAAAAA8D8AAAAAAADwPwAAAAAAAPA/AAAAAAAA8D8AAAAAAADwPwAAAAAAAAAAAAAAAAAA8D8AAAAAAADwPwAAAAAAAPA/AAAAAAAA8D8AAAAAAADwPwAAAAAAAAAAAAAAAAAA8D8AAAAAAAAAAAAAAAAAAPA/AAAAAAAA8D8AAAAAAADwPwAAAAAAAAAAAAAAAAAA8D8AAAAAAAAAAAAAAAAAAPA/AAAAAAAA8D8AAAAAAADwPwAAAAAAAAAAAAAAAAAA8D8AAAAAAADwPwAAAAAAAAAAAAAAAAAA8D8AAAAAAAAAAAAAAAAAAAAAAAAAAAAA8D8AAAAAAADwPwAAAAAAAPA/AAAAAAAA8D8AAAAAAAAAAAAAAAAAAPA/AAAAAAAAAAAAAAAAAAAAAAAAAAAAAPA/AAAAAAAAAAAAAAAAAADwPwAAAAAAAPA/AAAAAAAA8D8AAAAAAADwPwAAAAAAAPA/AAAAAAAA8D8AAAAAAADwPwAAAAAAAPA/AAAAAAAA8D8AAAAAAADwPwAAAAAAAPA/AAAAAAAAAAAAAAAAAADwPwAAAAAAAPA/AAAAAAAA8D8AAAAAAAAAAAAAAAAAAPA/AAAAAAAA8D8AAAAAAADwPwAAAAAAAPA/AAAAAAAA8D8AAAAAAAAAAAAAAAAAAPA/AAAAAAAA8D8AAAAAAADwPwAAAAAAAAAAAAAAAAAA8D8AAAAAAADwPwAAAAAAAAAAAAAAAAAAAAAAAAAAAADwPwAAAAAAAPA/AAAAAAAA8D8AAAAAAADwPwAAAAAAAPA/AAAAAAAA8D8AAAAAAADwPwAAAAAAAPA/AAAAAAAA8D8AAAAAAADwPwAAAAAAAPA/AAAAAAAA8D8AAAAAAAAAAAAAAAAAAPA/AAAAAAAA8D8AAAAAAADwPwAAAAAAAPA/AAAAAAAA8D8AAAAAAADwPwAAAAAAAPA/AAAAAAAAAAAAAAAAAADwPwAAAAAAAPA/AAAAAAAA8D8AAAAAAADwPwAAAAAAAPA/AAAAAAAA8D8AAAAAAADwPwAAAAAAAAAAAAAAAAAA8D8AAAAAAADwPwAAAAAAAPA/AAAAAAAA8D8AAAAAAADwPwAAAAAAAAAAAAAAAAAAAAAAAAAAAADwPwAAAAAAAPA/AAAAAAAA8D8AAAAAAADwPwAAAAAAAAAAAAAAAAAA8D8AAAAAAADwPwAAAAAAAAAAAAAAAAAA8D8AAAAAAADwPwAAAAAAAPA/AAAAAAAAAAAAAAAAAADwPwAAAAAAAPA/AAAAAAAA8D8AAAAAAADwPwAAAAAAAPA/AAAAAAAA8D8AAAAAAADwPwAAAAAAAPA/AAAAAAAA8D8AAAAAAADwPwAAAAAAAPA/AAAAAAAAAAAAAAAAAAAAAAAAAAAAAPA/AAAAAAAA8D8AAAAAAAAAAAAAAAAAAPA/AAAAAAAAAAAAAAAAAADwPwAAAAAAAPA/AAAAAAAA8D8AAAAAAADwPwAAAAAAAPA/AAAAAAAA8D8AAAAAAADwPwAAAAAAAPA/AAAAAAAA8D8AAAAAAADwPwAAAAAAAPA/AAAAAAAA8D8AAAAAAADwPwAAAAAAAAAAAAAAAAAA8D8AAAAAAADwPwAAAAAAAPA/AAAAAAAA8D8AAAAAAAAAAAAAAAAAAPA/AAAAAAAA8D8AAAAAAADwPwAAAAAAAPA/AAAAAAAA8D8AAAAAAAAAAAAAAAAAAPA/AAAAAAAA8D8AAAAAAAAAAAAAAAAAAPA/AAAAAAAA8D8AAAAAAADwPwAAAAAAAPA/AAAAAAAA8D8AAAAAAADwPwAAAAAAAPA/AAAAAAAA8D8AAAAAAADwPwAAAAAAAPA/AAAAAAAA8D8AAAAAAADwPwAAAAAAAPA/AAAAAAAA8D8AAAAAAADwPwAAAAAAAAAAAAAAAAAA8D8AAAAAAADwPwAAAAAAAPA/AAAAAAAAAAAAAAAAAADwPwAAAAAAAPA/AAAAAAAA8D8AAAAAAAAAAAAAAAAAAPA/AAAAAAAA8D8AAAAAAADwPwAAAAAAAPA/AAAAAAAA8D8AAAAAAADwPwAAAAAAAPA/AAAAAAAA8D8AAAAAAADwPwAAAAAAAPA/AAAAAAAAAAAAAAAAAADwPwAAAAAAAAAAAAAAAAAA8D8AAAAAAADwPwAAAAAAAPA/AAAAAAAA8D8AAAAAAAAAAAAAAAAAAPA/AAAAAAAA8D8AAAAAAADwPwAAAAAAAPA/AAAAAAAAAAAAAAAAAAAAAAAAAAAAAPA/AAAAAAAA8D8AAAAAAADwPwAAAAAAAPA/AAAAAAAA8D8AAAAAAADwPwAAAAAAAPA/AAAAAAAA8D8AAAAAAADwPwAAAAAAAPA/AAAAAAAAAAAAAAAAAADwPwAAAAAAAPA/D3QAAACwAQAABgAAAAAAAAAAAAAAAAAAAAAAAAAAAAAAAAAAAAAAAAAAAAAAAAAAAAAAAAAAAADwPwAAAAAAAAAAAAAAAAAAAAAAAAAAAAAAAAAAAAAAAAAAAAAAAAAA8D8AAAAAAAAAAAAAAAAAAPA/AAAAAAAAAAAAAAAAAAAAAAAAAAAAAAAAAAAAAAAA8D8AAAAAAADwPwAAAAAAAAAAAAAAAAAA8D8AAAAAAAAAAAAAAAAAAAAAAAAAAAAAAAAAAAAAAAAAAAAAAAAAAAAAAAAAAAAA8D8AAAAAAAAAAAAAAAAAAPA/AAAAAAAAAAAAAAAAAADwPwAAAAAAAAAAAAAAAAAAAAAAAAAAAAAAAAAAAAAAAAAAAAAAAAAAAAAAAAAAAAAAAAAAAAAAAAAAAAAAAAAAAAAAAAAAAAAAAAAAAAAAAAAAAAAAAAAAAAAAAAAAAAAAAAAAAAAAAAAAAAAAAAAAAAAAAAAAAAAAAAAAAAAAAAAAAAAAAAAA8D8AAAAAAAAAAAAAAAAAAAAAAAAAAAAAAAAAAAAAAAAAAAAAAAAAAAAAAAAAAAAAAAAAAAAAAAAAAAAAAAAAAAAAAAAAAAAAAAAAAAAAAAAAAAAAAAAAAAAAAAAAAAAAAAAAAAAAAAAAAAAAAAAAAAAAAAAAAAAA8D8AAAAAAAAAAAAAAAAAAAAAAAAAAAAAAAAAAAAAAADwPwAAAAAAAAAAAAAAAAAAAAAAAAAAAADwPwAAAAAAAAAAAAAAAAAAAAAAAAAAAAAAAAAAAAAAAAAAAAAAAAAAAAAAAAAAAAAAAAAAAAAAAAAAAAAAAAAAAAAAAAAAAAAAAAAAAAAAAAAAAAAAAAAA8D8AAAAAAAAAAAAAAAAAAAAAAAAAAAAAAAAAAAAAAAAAAAAAAAAAAPA/AAAAAAAAAAAAAAAAAAAAAAAAAAAAAAAAAAAAAAAAAAAAAAAAAAAAAAAAAAAAAAAAAAAAAAAAAAAAAAAAAAAAAAAAAAAAAAAAAAAAAAAAAAAAAAAAAADwPwAAAAAAAAAAAAAAAAAAAAAAAAAAAAAAAAAAAAAAAAAAAAAAAAAAAAAAAAAAAAAAAAAAAAAAAAAAAAAAAAAAAAAAAAAAAAAAAAAAAAAAAAAAAAAAAAAAAAAAAAAAAAAAAAAAAAAAAAAAAAAAAAAAAAAAAAAAAADwPwAAAAAAAAAAAAAAAAAA8D8AAAAAAAAAAAAAAAAAAPA/AAAAAAAAAAAAAAAAAADwPwAAAAAAAAAAAAAAAAAAAAAAAAAAAAAAAAAAAAAAAAAAAAAAAAAAAAAAAAAAAAAAAAAAAAAAAAAAAAAAAAAAAAAAAAAAAAAAAAAAAAAAAAAAAAAAAAAAAAAAAAAAAADwPwAAAAAAAAAAAAAAAAAAAAAAAAAAAAAAAAAAAAAAAPA/AAAAAAAA8D8AAAAAAAAAAAAAAAAAAPA/AAAAAAAAAAAAAAAAAAAAAAAAAAAAAAAAAAAAAAAA8D8AAAAAAAAAAAAAAAAAAPA/AAAAAAAAAAAAAAAAAAAAAAAAAAAAAAAAAAAAAAAAAAAAAAAAAAAAAAAAAAAAAAAAAAAAAAAAAAAAAAAAAADwPwAAAAAAAAAAAAAAAAAAAAAAAAAAAAAAAAAAAAAAAAAAAAAAAAAA8D8AAAAAAAAAAAAAAAAAAAAAAAAAAAAAAAAAAAAAAAAAAAAAAAAAAAAAAAAAAAAAAAAAAAAAAAAAAAAAAAAAAAAAAAAAAAAAAAAAAAAAAAAAAAAAAAAAAAAAAAAAAAAAAAAAAAAAAAAAAAAAAAAAAAAAAAAAAAAAAAAAAAAAAAAAAAAAAAAAAAAAAAAAAAAAAAAAAAAAAAAAAAAAAAAAAAAAAAAAAAAAAAAAAAAAAADwPwAAAAAAAAAAAAAAAAAAAAAAAAAAAAAAAAAAAAAAAAAAAAAAAAAAAAAAAAAAAAAAAAAAAAAAAAAAAAAAAAAAAAAAAAAAAAAAAAAAAAAAAAAAAAAAAAAAAAAAAAAAAAAAAAAAAAAAAAAAAAAAAAAAAAAAAAAAAAAAAAAAAAAAAAAAAAAAAAAAAAAAAAAAAAAAAAAAAAAAAAAAAAAAAAAAAAAAAAAAAAAAAAAAAAAAAAAAAAAAAAAAAAAAAAAAAAAAAAAAAAAAAAAAAAAAAAAAAAAAAAAAAAAAAAAAAAAAAAAAAAAAAAAAAAAAAAAAAAAAAAAAAAAAAAAAAAAAAAAAAAAAAAAAAAAAAAAAAAAAAAAAAAAAAAAAAAAAAAAAAAAAAAAAAAAAAAAAAAAAAAAAAAAAAAAAAAAAAAAAAAAAAAAAAAAAAAAAAAAAAAAAAADwPwAAAAAAAAAAAAAAAAAAAAAAAAAAAAAAAAAAAAAAAAAAAAAAAAAAAAAAAAAAAADwPwAAAAAAAAAAAAAAAAAA8D8AAAAAAAAAAAAAAAAAAAAAAAAAAAAAAAAAAAAAAAAAAAAAAAAAAAAAAAAAAAAAAAAAAAAAAADwPwAAAAAAAAAAAAAAAAAA8D8AAAAAAADwPwAAAAAAAAAAAAAAAAAAAAAAAAAAAAAAAAAAAAAAAAAAAAAAAAAA8D8AAAAAAAAAAAAAAAAAAPA/AAAAAAAA8D8AAAAAAAAAAAAAAAAAAPA/AAAAAAAAAAAAAAAAAAAAAAAAAAAAAAAAAAAAAAAAAAAAAAAAAAAAAAAAAAAAAAAAAAAAAAAAAAAAAAAAAAAAAAAAAAAAAAAAAAAAAAAAAAAAAAAAAAAAAAAAAAAAAPA/AAAAAAAAAAAAAAAAAAAAAAAAAAAAAAAAAAAAAAAA8D8AAAAAAAAAAAAAAAAAAAAAAAAAAAAAAAAAAAAAAAAAAAAAAAAAAAAAAAAAAAAA8D8AAAAAAAAAAAAAAAAAAAAAAAAAAAAAAAAAAAAAAADwPwAAAAAAAAAAAAAAAAAAAAAAAAAAAADwPwAAAAAAAPA/AAAAAAAAAAAAAAAAAAAAAAAAAAAAAAAAAAAAAAAAAAAAAAAAAAAAAAAAAAAAAAAAAAAAAAAAAAAAAAAAAAAAAAAAAAAAAAAAAAAAAAAAAAAAAAAAAAAAAAAAAAAAAAAAAAAAAAAA8D8AAAAAAAAAAAAAAAAAAAAAAAAAAAAAAAAAAAAAAAAAAAAAAAAAAAAAAAAAAAAAAAAAAAAAAAAAAAAAAAAAAPA/AAAAAAAAAAAAAAAAAAAAAAAAAAAAAAAAAAAAAAAAAAAAAAAAAAAAAAAAAAAAAAAAAAAAAAAAAAAAAAAAAAAAAAAAAAAAAAAAAAAAAAAAAAAAAAAAAAAAAAAAAAAAAAAAAAAAAAAAAAAAAAAAAADwPwAAAAAAAPA/AAAAAAAAAAAAAAAAAAAAAAAAAAAAAAAAAAAAAAAAAAAAAAAAAADwPwAAAAAAAAAAAAAAAAAAAAAAAAAAAADwPwAAAAAAAAAAAAAAAAAAAAAAAAAAAAAAAAAAAAAAAPA/AAAAAAAAAAAAAAAAAAAAAAAAAAAAAAAAAAAAAAAAAAAAAAAAAAAAAAAAAAAAAAAAAAAAAAAAAAAAAAAAAAAAAAAAAAAAAAAAAAAAAAAAAAAAAAAAAAAAAAAAAAAAAPA/AAAAAAAAAAAAAAAAAAAAAAAAAAAAAAAAAAAAAAAA8D8AAAAAAAAAAAAAAAAAAAAAAAAAAAAAAAAAAAAAAAAAAAAAAAAAAAAAAAAAAAAAAAAAAAAAAAAAAAAAAAAAAAAAAAAAAAAAAAAAAAAAAAAAAAAAAAAAAAAAAAAAAAAAAAAAAAAAAAAAAAAAAAAAAAAAAAAAAAAAAAAAAAAAAADwPwAAAAAAAAAAAAAAAAAAAAAAAAAAAAAAAAAAAAAAAAAAAAAAAAAA8D8AAAAAAAAAAAAAAAAAAAAAAAAAAAAAAAAAAAAAAAAAAAAAAAAAAAAAAAAAAAAA8D8AAAAAAAAAAAAAAAAAAAAAAAAAAAAAAAAAAAAAAAAAAAAAAAAAAAAAAAAAAAAAAAAAAAAAAAAAAAAAAAAAAAAAAAAAAAAAAAAAAAAAAAAAAAAAAAAAAAAAAAAAAAAAAAAAAAAAAAAAAAAAAAAAAAAAAAAAAAAAAAAAAAAAAAAAAAAAAAAAAAAAAAAAAAAAAAAAAAAAAADwPwAAAAAAAAAAAAAAAAAAAAAAAAAAAAAAAAAAAAAAAPA/AAAAAAAAAAAAAAAAAAAAAAAAAAAAAAAAAAAAAAAA8D8AAAAAAAAAAAAAAAAAAAAAAAAAAAAAAAAAAAAAAAAAAAAAAAAAAAAAAAAAAAAAAAAAAAAAAAAAAAAAAAAAAAAAAAAAAAAAAAAAAAAAAAAAAAAAAAAAAAAAAAAAAAAAAAAAAAAAAADwPwAAAAAAAAAAAAAAAAAAAAAAAAAAAAAAAAAAAAAAAAAAAAAAAAAA8D8AAAAAAAAAAAAAAAAAAAAAAAAAAAAAAAAAAAAAAAAAAAAAAAAAAAAAAAAAAAAA8D8AAAAAAAAAAAAAAAAAAAAAAAAAAAAAAAAAAAAAAAAAAAAAAAAAAAAAAAAAAAAAAAAAAAAAAAAAAAAAAAAAAAAAAAAAAAAAAAAAAAAAAAAAAAAAAAAAAPA/AAAAAAAAAAAAAAAAAAAAAA92AAAAsAEAAAYAAAAAAAAAAAAAAAAAAAAAAAAAAAAAAAAAAAAAAAAAAAAAAAAAAAAAAAAAAAAAAAAAAAAAAAAAAAAAAAAAAAAAAAAAAAAAAAAAAAAAAAAAAAAAAAAAAAAAAAAAAAAAAAAAAAAAAAAAAAAAAAAAAAAAAAAAAAAAAAAAAAAAAAAAAAAAAAAAAAAAAAAAAAAAAAAAAAAAAAAAAAAAAAAAAAAAAAAAAAAAAAAAAAAAAAAAAAAAAAAAAAAAAAAAAAAAAAAAAAAAAAAAAAAAAAAAAAAAAAAAAAAAAAAAAAAAAAAAAAAAAAAAAAAAAAAAAAAAAAAAAAAAAAAAAAAAAAAAAAAAAAAAAAAA8D8AAAAAAAAAAAAAAAAAAAAAAAAAAAAAAAAAAAAAAAAAAAAAAAAAAAAAAAAAAAAAAAAAAAAAAAAAAAAAAAAAAAAAAAAAAAAAAAAAAAAAAAAAAAAAAAAAAAAAAAAAAAAAAAAAAAAAAAAAAAAAAAAAAAAAAAAAAAAAAAAAAAAAAAAAAAAAAAAAAAAAAAAAAAAAAAAAAAAAAAAAAAAAAAAAAAAAAAAAAAAAAAAAAAAAAAAAAAAAAAAAAAAAAAAAAAAAAAAAAAAAAAAAAAAAAAAAAAAAAAAAAAAAAAAAAAAAAAAAAAAAAAAAAAAAAAAAAAAAAAAAAAAAAAAAAAAAAAAAAAAAAAAAAAAAAAAAAAAAAAAAAAAAAAAAAAAAAAAAAAAAAAAAAAAAAAAAAAAAAAAAAPA/AAAAAAAAAAAAAAAAAAAAAAAAAAAAAAAAAAAAAAAAAAAAAAAAAAAAAAAAAAAAAAAAAAAAAAAAAAAAAAAAAADwPwAAAAAAAAAAAAAAAAAAAAAAAAAAAAAAAAAAAAAAAAAAAAAAAAAAAAAAAAAAAAAAAAAAAAAAAAAAAAAAAAAAAAAAAAAAAAAAAAAAAAAAAAAAAAAAAAAAAAAAAAAAAAAAAAAAAAAAAAAAAAAAAAAAAAAAAAAAAAAAAAAAAAAAAAAAAAAAAAAAAAAAAAAAAAAAAAAAAAAAAAAAAAAAAAAAAAAAAAAAAAAAAAAAAAAAAAAAAAAAAAAAAAAAAAAAAAAAAAAAAAAAAAAAAAAAAAAAAAAAAAAAAAAAAAAAAAAAAAAAAAAAAAAAAAAAAAAAAAAAAAAAAAAAAPA/AAAAAAAAAAAAAAAAAAAAAAAAAAAAAAAAAAAAAAAAAAAAAAAAAAAAAAAAAAAAAAAAAAAAAAAAAAAAAAAAAAAAAAAAAAAAAAAAAAAAAAAAAAAAAAAAAAAAAAAAAAAAAAAAAAAAAAAAAAAAAAAAAAAAAAAAAAAAAAAAAAAAAAAA8D8AAAAAAAAAAAAAAAAAAAAAAAAAAAAAAAAAAAAAAAAAAAAAAAAAAAAAAAAAAAAAAAAAAAAAAAAAAAAAAAAAAAAAAAAAAAAAAAAAAAAAAAAAAAAAAAAAAAAAAAAAAAAAAAAAAAAAAAAAAAAAAAAAAAAAAAAAAAAAAAAAAAAAAAAAAAAAAAAAAAAAAAAAAAAAAAAAAAAAAAAAAAAAAAAAAAAAAAAAAAAAAAAAAAAAAAAAAAAAAAAAAAAAAAAAAAAAAAAAAAAAAAAAAAAAAAAAAAAAAAAAAAAAAAAAAAAAAAAAAAAAAAAAAAAAAAAAAAAAAAAAAAAAAAAAAAAAAAAAAAAAAAAAAAAAAAAAAAAAAAAAAAAAAAAAAAAAAAAAAAAAAAAAAAAAAAAAAAAAAAAAAAAAAAAAAAAAAAAAAAAAAAAAAAAAAAAAAAAAAAAAAAAAAAAAAAAAAAAAAAAAAAAAAAAAAAAAAAAAAAAAAAAAAAAAAAAAAAAAAAAAAAAAAAAAAAAAAAAAAAAAAAAAAAAAAAAAAAAAAAAAAAAAAAAAAAAAAAAAAAAAAAAAAAAAAAAAAAAAAAAAAAAAAAAAAAAAAAAAAAAAAAAAAAAAAAAAAAAAAAAAAAAAAAAAAAAAAAAAAAAAAAAAAAAAAAAAAAAAAAAAAAAAAAAAAAAAAAAAAAAAAAAAAAAAAAAAAAAAAAAAAAAAAAAAAAAAAAAAAAAAAAAAAAAAAAAAAAAAAAAAAAAAAAAAAAAAAAAAAAAAAAAAAAAAAAAAAAAAAAAAAAAAAAAAAAAAAAAAAAAAAAAAAAAAAAAAAAAAAAAAAAAAAAAAAAAAAAAAAAAAAAAAAAAAAAAAAAAAAAAAAAAAAAAAAAAAAAAAAAAAAAAAAAAAAAAAAAAAAAAAAAAAAAAAAAAAAAAAAAAAAAAA8D8AAAAAAAAAAAAAAAAAAAAAAAAAAAAAAAAAAAAAAAAAAAAAAAAAAAAAAAAAAAAAAAAAAAAAAAAAAAAAAAAAAPA/AAAAAAAAAAAAAAAAAAAAAAAAAAAAAAAAAAAAAAAAAAAAAAAAAAAAAAAAAAAAAAAAAAAAAAAAAAAAAAAAAAAAAAAAAAAAAAAAAAAAAAAA8D8AAAAAAAAAAAAAAAAAAAAAAAAAAAAAAAAAAAAAAAAAAAAAAAAAAAAAAAAAAAAAAAAAAAAAAAAAAAAAAAAAAAAAAAAAAAAAAAAAAAAAAAAAAAAAAAAAAAAAAAAAAAAAAAAAAAAAAAAAAAAAAAAAAAAAAAAAAAAAAAAAAAAAAAAAAAAAAAAAAAAAAAAAAAAAAAAAAAAAAAAAAAAAAAAAAAAAAAAAAAAAAAAAAAAAAAAAAAAAAAAAAAAAAAAAAAAAAAAAAAAAAAAAAAAAAAAAAAAAAAAAAAAAAAAAAAAAAAAAAAAAAAAAAAAAAAAAAAAAAAAAAAAAAAAAAAAAAAAAAAAAAAAAAAAAAAAAAAAAAAAAAAAAAAAAAAAAAAAAAAAAAAAAAAAAAAAAAAAAAAAAAAAAAAAAAAAAAAAAAAAAAAAAAAAAAAAAAAAAAAAAAAAAAAAAAAAAAAAAAAAAAAAAAAAAAAAAAAAAAAAAAAAAAAAAAAAAAAAAAAAAAAAAAAAAAAAAAAAAAAAAAAAAAAAAAAAAAAAAAAAAAAAAAAAAAAAAAAAAAAAAAAAAAAAAAAAAAAAAAAAAAAAAAAAAAAAAAAAAAAAAAAAAAAAAAAAAAAAAAAAAAAAAAAAAAAAAAAAAAAAAAAAAAAAAAAAAAAAAAAAAAAAAAAAAAAAAAAAAAAAAAAAAAAAAAAAAAAAAAAAAAAAAAAAAAAAAAAAAAAAAAAAAAAAAAAAAAAAAAAAAAAAAAAAAAAAAAAAAAAAAAAAAAAAAAAAAAAAAAAAAAAAAAAAAAAAAAAAA8D8AAAAAAAAAAAAAAAAAAAAAAAAAAAAAAAAAAAAAAAAAAAAAAAAAAAAAAAAAAAAAAAAAAAAAAAAAAAAAAAAAAAAAAAAAAAAAAAAAAAAAAAAAAAAAAAAAAAAAAAAAAAAAAAAAAAAAAAAAAAAAAAAAAAAAAAAAAAAAAAAAAAAAAAAAAAAAAAAAAAAAAAAAAAAAAAAAAAAAAAAAAAAAAAAAAAAAAAAAAAAAAAAAAAAAAAAAAAAAAAAAAAAAAAAAAAAAAAAAAAAAAAAAAAAAAAAAAAAAAAAAAAAAAAAAAAAAAAAAAAAAAAAAAAAAAAAAAAAAAAAAAAAAAAAAAAAAAAAAAPA/AAAAAAAAAAAAAAAAAAAAAAAAAAAAAAAAAAAAAAAAAAAAAAAAAADwPwAAAAAAAAAAAAAAAAAAAAAAAAAAAAAAAAAAAAAAAAAAAAAAAAAAAAAAAAAAAAAAAAAAAAAAAAAAAAAAAAAAAAAAAAAAAAAAAAAAAAAAAAAAAAAAAAAAAAAAAAAAAAAAAAAAAAAAAAAAAAAAAAAAAAAAAAAAAAAAAAAAAAAAAAAAAAAAAAAAAAAAAAAAAAAAAAAAAAAAAAAAAAAAAAAAAAAAAAAAAAAAAAAAAAAAAAAAAAAAAAAAAAAAAAAAAAAAAAAAAAAAAAAAAAAAAAAAAAAAAAAAAAAAAAAAAAAAAPA/AAAAAAAAAAAAAAAAAAAAAAAAAAAAAAAAAAAAAAAAAAAAAAAAAAAAAAAAAAAAAAAAAAAAAAAAAAAAAAAAAAAAAAAAAAAAAAAAAAAAAAAAAAAAAAAAAAAAAAAAAAAAAAAAAAAAAAAAAAAAAAAAAAAAAAAAAAAAAAAAAAAAAAAAAAAAAAAAAAAAAAAAAAAAAAAAAAAAAAAAAAAAAAAAAAAAAAAAAAAAAAAAAAAAAAAAAAAAAAAAAAAAAAAAAAAAAAAAAAAAAAAAAAAAAAAAAAAAAAAAAAAAAAAAAAAAAAAAAAAAAAAAAAAAAAAAAAAAAAAAAAAAAAAAAAAAAAAAAAAAAAAAAAAAAAAAAAAAAAAAAAAAAAAAAADwPwAAAAAAAAAAAAAAAAAAAAAAAAAAAAAAAAAAAAAAAAAAAAAAAAAAAAAAAAAAAAAAAAAAAAAAAAAAAAAAAAAAAAAAAAAAAAAAAAAAAAAAAAAAAAAAAAAAAAAAAAAAAADwPwAAAAAAAAAAAAAAAAAAAAAAAAAAAAAAAAAAAAAAAAAAAAAAAAAAAAAAAAAAAAAAAAAAAAAAAAAAAAAAAAAAAAAAAAAAAAAAAAAAAAAAAAAAAAAAAAAAAAAAAAAAAAAAAAAAAAAAAAAAAAAAAAAAAAABeQAAAHEAAAAEAAAAAW////9y////agAAAAAAAACxAQAACY8AAAAGkwAAAAhNYXRjaGluZwAAAQAAAAF7AAAAcQAAABoAAAABbP///3L///8CAAAAAAAAALEBAAAJjwAAAAaWAAAACE1hdGNoaW5nAAAAAAAACw== <-!redo!!

**Logistic regression - model analysis**

Accuracy = 0,01

Log likelihood with all covariates = 0

Deviance with all covariates = 0 df = -10 rank = 10

Akaike information criterion = 20

Schwartz information criterion = 38,789966

Deviance with no covariates = 62,481692

Deviance (likelihood ratio) chi-square = 62,481692 df = 43 P = 0,0276

Pseudo (McFadden) R-square = 1

Pseudo (likelihood ratio index) R-square = 1

Pearson chi-square goodness of fit = 0 df = -10 * saturated model: can't assess goodness of fit

Deviance goodness of fit = * df = -10 P = *

Hosmer-Lemeshow test = * df = 0 P = *

| Parameter | Coefficient | Standard Error | Z Value | P(>\|Z\|) |
| --- | --- | --- | --- | --- |
| (intercept) | 0 | 1 | 0 | P > 0,9999 |
| Valve or ring size(21) | 0 | 1 | 0 | P > 0,9999 |
| Valve or ring size(23) | 0 | 1 | 0 | P > 0,9999 |
| Valve or ring size(25) | 0 | 1 | 0 | P > 0,9999 |
| Valve or ring size(27) | 0 | 1 | 0 | P > 0,9999 |
| Cardiac procedures | 0 | 1 | 0 | P > 0,9999 |
| EF(1) | 0 | 1 | 0 | P > 0,9999 |
| EF(2) | 0 | 1 | 0 | P > 0,9999 |
| EF(3) | 0 | 1 | 0 | P > 0,9999 |
| Gender | 0 | 1 | 0 | P > 0,9999 |

!!help!-> 1276 <-!help!! !!redo!-> "LogisticRegression" AAEAAAD/////AQAAAAAAAAAMAgAAAEJTdGF0c0RpcmVjdCwgVmVyc2lvbj0zLjMuNS4wLCBDdWx0dXJlPW5ldXRyYWwsIFB1YmxpY0tleVRva2VuPW51bGwFAQAAACJTdGF0c0RpcmVjdC5UZW1wbGF0ZXMuUGFyYW1ldGVyQmFnAQAAABBmaWxsZWRQYXJhbWV0ZXJzA/EBU3lzdGVtLkNvbGxlY3Rpb25zLkdlbmVyaWMuRGljdGlvbmFyeWAyW1tTeXN0ZW0uU3RyaW5nLCBtc2NvcmxpYiwgVmVyc2lvbj00LjAuMC4wLCBDdWx0dXJlPW5ldXRyYWwsIFB1YmxpY0tleVRva2VuPWI3N2E1YzU2MTkzNGUwODldLFtTdGF0c0RpcmVjdC5UZW1wbGF0ZXMuRmlsbGVkUGFyYW1ldGVyLCBTdGF0c0RpcmVjdCwgVmVyc2lvbj0zLjMuNS4wLCBDdWx0dXJlPW5ldXRyYWwsIFB1YmxpY0tleVRva2VuPW51bGxdXQIAAAAJAwAAAAQDAAAA8QFTeXN0ZW0uQ29sbGVjdGlvbnMuR2VuZXJpYy5EaWN0aW9uYXJ5YDJbW1N5c3RlbS5TdHJpbmcsIG1zY29ybGliLCBWZXJzaW9uPTQuMC4wLjAsIEN1bHR1cmU9bmV1dHJhbCwgUHVibGljS2V5VG9rZW49Yjc3YTVjNTYxOTM0ZTA4OV0sW1N0YXRzRGlyZWN0LlRlbXBsYXRlcy5GaWxsZWRQYXJhbWV0ZXIsIFN0YXRzRGlyZWN0LCBWZXJzaW9uPTMuMy41LjAsIEN1bHR1cmU9bmV1dHJhbCwgUHVibGljS2V5VG9rZW49bnVsbF1dBAAAAAdWZXJzaW9uCENvbXBhcmVyCEhhc2hTaXplDUtleVZhbHVlUGFpcnMAAwADCJIBU3lzdGVtLkNvbGxlY3Rpb25zLkdlbmVyaWMuR2VuZXJpY0VxdWFsaXR5Q29tcGFyZXJgMVtbU3lzdGVtLlN0cmluZywgbXNjb3JsaWIsIFZlcnNpb249NC4wLjAuMCwgQ3VsdHVyZT1uZXV0cmFsLCBQdWJsaWNLZXlUb2tlbj1iNzdhNWM1NjE5MzRlMDg5XV0I9QFTeXN0ZW0uQ29sbGVjdGlvbnMuR2VuZXJpYy5LZXlWYWx1ZVBhaXJgMltbU3lzdGVtLlN0cmluZywgbXNjb3JsaWIsIFZlcnNpb249NC4wLjAuMCwgQ3VsdHVyZT1uZXV0cmFsLCBQdWJsaWNLZXlUb2tlbj1iNzdhNWM1NjE5MzRlMDg5XSxbU3RhdHNEaXJlY3QuVGVtcGxhdGVzLkZpbGxlZFBhcmFtZXRlciwgU3RhdHNEaXJlY3QsIFZlcnNpb249My4zLjUuMCwgQ3VsdHVyZT1uZXV0cmFsLCBQdWJsaWNLZXlUb2tlbj1udWxsXV1bXQkAAAAJBAAAABEAAAAJBQAAAAQEAAAAkgFTeXN0ZW0uQ29sbGVjdGlvbnMuR2VuZXJpYy5HZW5lcmljRXF1YWxpdHlDb21wYXJlcmAxW1tTeXN0ZW0uU3RyaW5nLCBtc2NvcmxpYiwgVmVyc2lvbj00LjAuMC4wLCBDdWx0dXJlPW5ldXRyYWwsIFB1YmxpY0tleVRva2VuPWI3N2E1YzU2MTkzNGUwODldXQAAAAAHBQAAAAABAAAACQAAAAPzAVN5c3RlbS5Db2xsZWN0aW9ucy5HZW5lcmljLktleVZhbHVlUGFpcmAyW1tTeXN0ZW0uU3RyaW5nLCBtc2NvcmxpYiwgVmVyc2lvbj00LjAuMC4wLCBDdWx0dXJlPW5ldXRyYWwsIFB1YmxpY0tleVRva2VuPWI3N2E1YzU2MTkzNGUwODldLFtTdGF0c0RpcmVjdC5UZW1wbGF0ZXMuRmlsbGVkUGFyYW1ldGVyLCBTdGF0c0RpcmVjdCwgVmVyc2lvbj0zLjMuNS4wLCBDdWx0dXJlPW5ldXRyYWwsIFB1YmxpY0tleVRva2VuPW51bGxdXQT6////8wFTeXN0ZW0uQ29sbGVjdGlvbnMuR2VuZXJpYy5LZXlWYWx1ZVBhaXJgMltbU3lzdGVtLlN0cmluZywgbXNjb3JsaWIsIFZlcnNpb249NC4wLjAuMCwgQ3VsdHVyZT1uZXV0cmFsLCBQdWJsaWNLZXlUb2tlbj1iNzdhNWM1NjE5MzRlMDg5XSxbU3RhdHNEaXJlY3QuVGVtcGxhdGVzLkZpbGxlZFBhcmFtZXRlciwgU3RhdHNEaXJlY3QsIFZlcnNpb249My4zLjUuMCwgQ3VsdHVyZT1uZXV0cmFsLCBQdWJsaWNLZXlUb2tlbj1udWxsXV0CAAAAA2tleQV2YWx1ZQEEK1N0YXRzRGlyZWN0LlRlbXBsYXRlcy5GaWxsZWRPYmplY3RQYXJhbWV0ZXICAAAABgcAAAAHY29udGV4dAkIAAAAAff////6////BgoAAAATY2FuZGlkYXRlUHJlZGljdG9ycwkLAAAAAfT////6////Bg0AAAAKcHJlZGljdG9ycwkOAAAAAfH////6////BhAAAAAIcmVzcG9uc2UJEQAAAAHu////+v///wYTAAAACGdyb3VwaW5nCRQAAAAB6/////r///8GFgAAAAhhY2N1cmFjeQkXAAAAAej////6////BhkAAAAJaW50ZXJjZXB0CRoAAAAB5f////r///8GHAAAAAd3ZWlnaHRzCR0AAAAB4v////r///8GHwAAAAVnYW1tYQkgAAAABQgAAAArU3RhdHNEaXJlY3QuVGVtcGxhdGVzLkZpbGxlZE9iamVjdFBhcmFtZXRlcgIAAAAVPERhdGE+a19fQmFja2luZ0ZpZWxkKkZpbGxlZFBhcmFtZXRlcis8RGlyZWN0aW9uPmtfX0JhY2tpbmdGaWVsZAIELlN0YXRzRGlyZWN0LlRlbXBsYXRlcy5GaWxsZWRQYXJhbWV0ZXJEaXJlY3Rpb24CAAAAAgAAAAkhAAAABd7///8uU3RhdHNEaXJlY3QuVGVtcGxhdGVzLkZpbGxlZFBhcmFtZXRlckRpcmVjdGlvbgEAAAAHdmFsdWVfXwAIAgAAAAEAAAAFCwAAAC5TdGF0c0RpcmVjdC5UZW1wbGF0ZXMuRmlsbGVkRGF0YUZyYW1lUGFyYW1ldGVyAgAAABU8RGF0YT5rX19CYWNraW5nRmllbGQqRmlsbGVkUGFyYW1ldGVyKzxEaXJlY3Rpb24+a19fQmFja2luZ0ZpZWxkBAQaU3RhdHNEaXJlY3QuRGF0YS5EYXRhRnJhbWUCAAAALlN0YXRzRGlyZWN0LlRlbXBsYXRlcy5GaWxsZWRQYXJhbWV0ZXJEaXJlY3Rpb24CAAAAAgAAAAkjAAAAAdz////e////AQAAAAEOAAAACwAAAAklAAAAAdr////e////AQAAAAERAAAACwAAAAknAAAAAdj////e////AQAAAAUUAAAAK1N0YXRzRGlyZWN0LlRlbXBsYXRlcy5GaWxsZWRTdHJpbmdQYXJhbWV0ZXICAAAAFTxEYXRhPmtfX0JhY2tpbmdGaWVsZCpGaWxsZWRQYXJhbWV0ZXIrPERpcmVjdGlvbj5rX19CYWNraW5nRmllbGQBBC5TdGF0c0RpcmVjdC5UZW1wbGF0ZXMuRmlsbGVkUGFyYW1ldGVyRGlyZWN0aW9uAgAAAAIAAAAGKQAAAAppbmRpdmlkdWFsAdb////e////AQAAAAEXAAAAFAAAAAYrAAAACTAuMDAwMDAwMQHU////3v///wEAAAAFGgAAACxTdGF0c0RpcmVjdC5UZW1wbGF0ZXMuRmlsbGVkQm9vbGVhblBhcmFtZXRlcgIAAAAVPERhdGE+a19fQmFja2luZ0ZpZWxkKkZpbGxlZFBhcmFtZXRlcis8RGlyZWN0aW9uPmtfX0JhY2tpbmdGaWVsZAAEAS5TdGF0c0RpcmVjdC5UZW1wbGF0ZXMuRmlsbGVkUGFyYW1ldGVyRGlyZWN0aW9uAgAAAAIAAAABAdP////e////AQAAAAEdAAAAGgAAAAAB0v///97///8BAAAABSAAAAArU3RhdHNEaXJlY3QuVGVtcGxhdGVzLkZpbGxlZERvdWJsZVBhcmFtZXRlcgIAAAAVPERhdGE+a19fQmFja2luZ0ZpZWxkKkZpbGxlZFBhcmFtZXRlcis8RGlyZWN0aW9uPmtfX0JhY2tpbmdGaWVsZAAEBi5TdGF0c0RpcmVjdC5UZW1wbGF0ZXMuRmlsbGVkUGFyYW1ldGVyRGlyZWN0aW9uAgAAAAIAAABmZmZmZmbuPwHR////3v///wEAAAAFIQAAADxTdGF0c0RpcmVjdC5CdWlsdGlucy5SZWdyZXNzK011bHRpcGxlTGluZWFyUmVncmVzc2lvbkNvbnRleHQoAAAAFDxBcmc+a19fQmFja2luZ0ZpZWxkEjxCPmtfX0JhY2tpbmdGaWVsZBs8Q292YXJpYW5jZT5rX19CYWNraW5nRmllbGQUPERFVj5rX19CYWNraW5nRmllbGQVPERFVlg+a19fQmFja2luZ0ZpZWxkEzxERj5rX19CYWNraW5nRmllbGQUPERGWD5rX19CYWNraW5nRmllbGQUPERvQz5rX19CYWNraW5nRmllbGQTPERWPmtfX0JhY2tpbmdGaWVsZBI8TT5rX19CYWNraW5nRmllbGQTPEZWPmtfX0JhY2tpbmdGaWVsZBM8SDE+a19fQmFja2luZ0ZpZWxkEjxIPmtfX0JhY2tpbmdGaWVsZBc8TGFiZWxzPmtfX0JhY2tpbmdGaWVsZBQ8TExYPmtfX0JhY2tpbmdGaWVsZBI8Tj5rX19CYWNraW5nRmllbGQdPE91dGNvbWVUaXRsZT5rX19CYWNraW5nRmllbGQSPFA+a19fQmFja2luZ0ZpZWxkEjxSPmtfX0JhY2tpbmdGaWVsZBM8UjI+a19fQmFja2luZ0ZpZWxkFTxSQU5LPmtfX0JhY2tpbmdGaWVsZBM8UlY+a19fQmFja2luZ0ZpZWxkFDxSWEk+a19fQmFja2luZ0ZpZWxkEjxTPmtfX0JhY2tpbmdGaWVsZBM8U2U+a19fQmFja2luZ0ZpZWxkFjxTU1JFRz5rX19CYWNraW5nRmllbGQUPFNTWT5rX19CYWNraW5nRmllbGQTPFNWPmtfX0JhY2tpbmdGaWVsZBI8VD5rX19CYWNraW5nRmllbGQXPFRpdGxlcz5rX19CYWNraW5nRmllbGQUPFRPTD5rX19CYWNraW5nRmllbGQSPFY+a19fQmFja2luZ0ZpZWxkFDxWSUY+a19fQmFja2luZ0ZpZWxkFTx3YXJuPmtfX0JhY2tpbmdGaWVsZBc8V0VJR0hUPmtfX0JhY2tpbmdGaWVsZBw8d2VpZ2h0VGl0bGU+a19fQmFja2luZ0ZpZWxkEzxXVD5rX19CYWNraW5nRmllbGQSPFg+a19fQmFja2luZ0ZpZWxkEzxYMT5rX19CYWNraW5nRmllbGQSPFk+a19fQmFja2luZ0ZpZWxkBwcHAAAAAAAHAAcHAwYAAAEABwMABwcHBwAABwcGAAMHAQABBwMHBwYGBgYGCAgBBggGBhBTeXN0ZW0uRG91YmxlWyxdBggIBhBTeXN0ZW0uRG91YmxlWyxdCAYIBgYGBgYGBhBTeXN0ZW0uRG91YmxlWyxdBgEGEFN5c3RlbS5Eb3VibGVbLF0GBgIAAAAKCTAAAAAJMQAAAP0zPM4CJ3RA7al6pfYRd0AFAQAADQEAAAEKCgAAAAkyAAAACTMAAAAKCTQAAACtRNtvetFwwA4BAAAKCwAAAAk1AAAACgkAAAAKCTYAAAAKCTcAAAAAAAAAAAAAAAAAAAAAAAAACgk4AAAACnsUrkfheoQ/CgoKAAoJOQAAAAk6AAAACgk7AAAABSMAAAAaU3RhdHNEaXJlY3QuRGF0YS5EYXRhRnJhbWUCAAAAFTxOYW1lPmtfX0JhY2tpbmdGaWVsZBo8VmFyaWFibGVzPmtfX0JhY2tpbmdGaWVsZAEDgwFTeXN0ZW0uQ29sbGVjdGlvbnMuR2VuZXJpYy5MaXN0YDFbW1N0YXRzRGlyZWN0LkRhdGEuSVZhcmlhYmxlLCBTdGF0c0RpcmVjdCwgVmVyc2lvbj0zLjMuNS4wLCBDdWx0dXJlPW5ldXRyYWwsIFB1YmxpY0tleVRva2VuPW51bGxdXQIAAAAKCTwAAAABJQAAACMAAAAGPQAAAAZEYXRhIDEJPgAAAAEnAAAAIwAAAAY/AAAABkRhdGEgMQlAAAAADzAAAAAMAAAABgAAAAAAAAAAklaJg9LjDMCRDe5aXJf6v+31a6VQjfe/EMq5YbZf5L+U2iFRGWfEPxgwPPVcn7Q/XCszcBxR9L/wetzigm34v7kNBmgLEuq/cpzHjFIe8D/WFAXm+yKxPw8xAAAAQwAAAAYAAAAAAAAAADzwFe9r9t4/BnjYuHjawz/4KnGRMgm/P0vcnIA+ssA/+8DD2BKzoj+LPm5HMbaxP9bqFKanLLw/Wg0c2e5KjT8MgljG2rmbP99MVss2PLA/bi1Yo5mTtj9rdBadoVyRvzjfkIWMNmO/nLxP4MSJdT+3VcUg2iu6P3iWrbftnjO/F2WHx6+Kdr//NkF/nkOBv2ROf7hIQVu/q2ZXDChUjz9JM237/FutP5TNwDAnvcE/xAw82mOApT9ESj9yr16fP5rCAazzq5s/FqOmWefuoz+XCrjmu7N6P7WEq3iYNrk/sqeilttHxT8zC7/hJeeqP8ydEYwWN6c/myBm2G93pD9SqqcohBOdP7Xy5VCNb2q/wo6ahyC7rz9HuTxL+53BPzxryBbV58Y/LchmJ1kCrz9ILkK9i+KrP9LTwp3lC6Y/RcVrsRNdlT9X1X8zyGttv6LD3GtpZ5a/BtUCWp8Tob/0uMQaqvnNP3GS/v+MvqG/f+2RSdnNq78gLT86dIeRv+HX2OC5I4w/Ynf53C+Ulz9iX0R7BQKQPygNhCNfNJK/8KSpRkXFer/8XJ0V0y6Fv8E5Jp5BuLY/ICzzqOG1hL+ymzc1JhJovykU+KDu6GW/gfg992UgZL8fCF/WC7xgv2kGsqvqUDu/rznbumHGbL9shnvXXNxuv3XwdRHINGe/qPl+lUfpMD+oeyHYPZItPw8yAAAADwEAAAYAAAAAAAAAALNUvmzCE+Q/GFXkJ3hwwz9BD3u7dLDuPyIz4CT/+NA/ueSXSGN69j/+LaBy/IXCPzO1wv4pFvE/Mxysk2c39j999aXwMXbsP38AOuhuOgJATYoGacwj2D9wvPlBuDnbP8SA7yBlga0/9UG49JPsxj/EoQpG1YjPP9NGhpQ+984/MREn1lM+xz/xJE5utSPSP9YkgNSWAdY/03s+Nxrx9j+90YpNP4+wP4erti+zrOg/hlGGoTmV0D8ySe8XwvLKP/mAXjFCTuA/LZOrPdAntD/A36CPWwXjP82/uD73Aes/+17ar8o42T8j78FQ6zvMP4JrrgF0/8U/1QfZU/U04z9mHin5C0fsPyvPZFwja+E/KH/7sPsJ5D/vPUPeMeryP5huicQibtM/j7gDcuIy4j/gMIhIDk3mPwzuyf5yGOI/tv4Qvceyqz9RqP0cEoXYP+X2CYIY39w/8RDhHPAGsD+ynTds37XyPw5XngFIZc4/m9lNshD64j9w9lz3V2nxP5MBz2A0+fk/F9ft0gR5yT/zlfLq4KW7Px+ZjsNdgds/J908lUmG3j+cwq2dGJXQP5FTkdJuQvQ/PwEtKMB14T9GEeWzmD7mP63LB2zUeOI/x4OCYIrLxz8OnAEhvRrSP2xlI1fE+80/1nP8nHZm4T9rH5GDIXXlP76Uvqkwj9Q/Ijm530vBnD8QAe/2zX7kP6yi5DLydeo/hAgkmYD8xj+IQ2uRVSncP6pv+50VNOM/TGRCOp6vlT8uOMbIwd2bP1GHdZqFfMg/aWawbefq+D+t439egSTnP0JpXlv0R+E/F5e6+F2H2z+CViSg+wTLP9d5an7CxeQ/0vs39cRn/T80AkQilfTDP+kRlrIsifI/goqjlaGx4j9VkdBEZBztPyNVq1iKjbQ/FhWySzlY3D/12aC2sjStP28UZpJMPdE/9HAzIoegsT9k1zf3dDnUPzaXb7Gt+OM/XY3zKnCowT/9oJAJhpfzP5LFKEG5s8w/DAEtGMZCwz+HBVqv5XzBP4A0Jta7nNU/vYdvw6GD4j/5H3aT+2bLPzVneXPGDMs/CiV4knx8/j/NEs813JvpPxk0CE4+St8/QMh3ASap5z/ME/6FWp/UP7IELQpU1+A/o1wn9AZP0j/dn7CdXk/TP24DO2xY9+U/wOVHbZyH3j8Idv1uU0LXP7NFRM42U8E/y91S06CU1j+lVZLQynjuPyie14PJT/c/uQJoUqjw6j8XFAOSq2jzPxTH2XiNVb8/tDHp2hD75z82u5G+JZbyP0QUKRcT4cQ/03dBnu8Q0D9ia51VKYTvP/dmu0rpurk/lVI+yv382T+hVarcT1PEP5QbWOcIO94/NCaUI6km0D/MY+JcoSrTP/T3cGShRsg/gNnKrM0e1j9tIKCQWRnXPw3aYNFOQv0/xTFZmS5F2T+DFR6iyba1P1WWhUJcpdE/J9OJjjsO2z9wlT/jbGfeP/oRdQzQiNU/GZAW3EbP5T/ESM7bWWr0P5r8HX6eyOA/+n5e7jQzwD9CZHfkxQjnP1T99oHfaOg/YnR7oE315T8PFGFe57W/P0cY1N4cWbg/YkN1+ILPwT/GjKlOCJbHP7C6EB/PEeQ/fmTdESOr8D+QgeqLMGzWP0QovQu+KdY/bUKsFvWzzD+GwDgnGYq9P5ymjjFjedE/fiQvWD629T8dCfnTuijjP1LqGWmV2+Q/f9SWM+HvxT9hPt4R0V/iPxBbvnbpQ9Q/LRhXs1I06D/mb0FkaZDyPyluT5R1g+Q/q+42+saS2D8y+W062dfaP8/lyAn2INM/fIYqxzNt7z96fiZwO86xP9h96bNGNs8/cnxpTKAC1T8g7tPRZ5DUP95RYfi+Sus/dEiHYFDO6T+yWgs3NnXiP264+Ai5RfA/r2W2Hh1Gxj8FwRN0TYjHP5/qERV08sU/1I9jexIQ3D+v9hb0snPTPx7rXZRsTvA/VeM6y15q1D/X8Y2Ep53DPwmeq7KsR/I/vYf2SC+o0j/pvKeFg2qrPyahyYutT+I/B4O9Mb5UyT+jC5821ja4P81kC8o7wMU/ukkETzLr9D/OaOAulQTRPwGg7kBgYsM/hn9bV4tf4j9F8wN5z2jQP6T6237TRdI/wLFbuWe4xj/9590mQR/BP2zLda62bb0/0fBPFDY9yz+PiEZxqc3QP0SS7bBtI9I/ApgAe6Vd5z8jivBPZzvhP7v0cCzdGOQ/mfVEdHgv4D/gNtS2wv7jP3yQLSF4ANg/JSCQC5tt2T9ugBEv+bLEPxxWIC3ya80/F2FGlTb96D/ZQ+UpdO31P/L1cD+etdA/tVJZGfsU5T91X/v7Uy3NP4+eLM93l9E/XLUq7Mzu3z/tZFSn2K77P8rsrpu8deU/S6XLraXO4D/GIJFm72XJP2ff919z0Og/5ztP7ZUT4D8QMAx+mhjYPy9qi3HdpNI/lCUhAt1q2j9Ds/YT++3TP5zSa0B6St0/SdQVRQyl3j/MROtOzFDkPwpItUrZ2OE/tTbJe+g/2j/licl9n3nmP8YPsydzRtk/2lU3fgHG5z9+l/BYIDvsP/1dlRcfZ+E/cJnrMohP1T8sukjWXg7KPxAeP4QpSuU/C8b7pOvw4z/7VQ3LXlveP6Lj9ZMxfuQ/ZEGNr3TC7z9DGdMjNpjfP3IrlMy3wb4/4OTCQ8jUvj9vfuGTphvhP2+LXdmFQ9U/I8VZ1ujk4j9gS2j7N8jGPy2guuZUb7U/KN2J0Xb71j8K7X56Hx/dP6V87Z1SaN0/YIEb3r8PvT/7L5uyOBnYP/bXpc3GANU/vjJF6caC4T/oNix9sE7AP1Mwxc/0I9g/GgSQFYVq6D/FTakvj4zRP1rLB2cRBNo/R4hnq6Lv1z8rC4Zkzy/uPw8zAAAADwEAAAYAAAAAAAAAAAmhRU5VyZ8/r0U6sadmkD/ZCAYBD0ahP5uDQ+LKTIs/SlJGMBMpsj9PrbayQi2RP1dSQWnoEKU//FIWmYS2qD9oD+jRwx+nP9hnw/AHU7s/YvbM5bPhpD+gfzQOlq+ZPzfbRbNhhog/2iY642cAmD+a7lqh0XuJP3z5hoaEQJg/ZrKsBz2ZoD+qdgr3pT6TP10BZcoO4KY/d+7s7Y0MsT/ql0UfNmKBP8yOU7204qQ/LUgavTiqjj+Xr5vjtXKcP/puN0Kt150/Q5hkGx4bjD98orMQ6jabP0FzkghrM6Y/+MpAz+Rytz+vn+Q91iuNP7scxB0KOpc//be/gdG8oz/rL91F50yqP/HliLzWxJ4/aDAjlFgAoz8TxGQriG2kPxjhO4cfYZM/KKHUoLUynz+LYScw+oGlP20LSnjQyaI/Quui6jfohz/lD0MJE6ucP5GXmHGxaJY/ZrpNZD1XjT9DOonOoFmrPz8qYeUpGIY/I56rmIk3pD+IqxJMhpysP0BD+Myk9LI/kLZeoPbGkT/Ls4rLVbORP1Zx+t0ZRo4/tlxuZAwomj8pb+tIzROhP0a3dJaSpa0/1M+UznLzqD/OOMXvCmilP4SnbWTIyaE/QNynlkQTlz/Yxz32YUe3PyZYY/Bxu44/gZ/Dt/MHmT/n6RhiiXSaP3kSm8lmmJM/moZi5fbglD9dPCumRaGnPxTubpHMM50/MZxWnwnljz9klbD6ttaaP/ez/rPfZqQ/9UPRuR/Chz8i5tkwW52IPwsKGdfimKE/CZO8X0dprz9Mx1FS5uGnP9AUfsZjips/EHwQtBranD8tCC3l5BOPP4BMkZt2G6Y/h1unS6k/sT/eQXkngoORP1+qVciPiac/esfjKiegmj9U9ul1+ZCgP+XfBvlgeI0/1T3n4AgzkD/exaIk1ESBP/wCtHyYe5E/zMOf0PZtgT/LDJa1b9+gP4G43hf947g/ucckbc63oT9iDZVeXnGqP4NDvBBl/5c/oPr5QCF4kD9mFdISm6CQP+WNGYHBlJs/sKsRNx/VlT99lc6/NMuGPz/omHvI5ZE/dgW24vTfsD8L677FMG6lP3iS/REjO5w//Y9dDyz+pj9g0gsSi/64PxZitD7kQJY/DuWVO0YjpD/O4dE4sPqgP6E8FPaClaA/Yx35alpSpT+0HUMQwCykP0dLCM41JoM/1gi75iFKnD9TOBmxt2KiP8RIv/7JWaw/1bVOQfcFsz9BrledqjGxPwOzFqiXvIs/oOTEfQZmoD8YRPo4NhmmP44aWelpS4k/AOvn3MNMoj9DLmrk1EyoP2vSSRL5G5E/eoahegw8lz9fs4QMT1WEP6MNpcavX5Y/U7N/yDLDiz/PiWzoQVGPP8/0UYA0mY8/VZop3AWroD+JTYvcR5OgP1N/M2Rb6LA/k1JzpJq3nD+RfIGNGbqWP/3gpjceuIU/kosTkU8LlT+2IpIJM7ibP3/E00MPFZs/J9fLIRKmmz+v+hB3fI2xP/uY2yc2g6A/ruLSLrEXkT//QUbhezelP6H+ZRVNmJs/TQqNWgQjpD/fse6SY4WRP/DyhwbZ7pY/ijIGDEcckT8tNlfyo2SPPz2bNvHKdaE/AbBSuB7goT9Y4QPA3KyKP6hzSq3dlpc/uobMnrEHnD/1B5lt5iCMP3torQ6poLQ/6z5ElXG/qT9WEh8huEugP6UbruMsXZs/ymNbBggskz+XZZyIzJe6P4fewvHwK5c/JzxqZIfomT+836/2jy64P71Wnha4YZo/s1YdQoTnnT+G5vAyrYqPPzmF/+IjXrg/j4r/CBhesD/mS8segI6KP31OFrn4dI4/3qv23gAtoz8p3QaWMpqnP3gJCTopfJ4/56yCIvtpnj/WssNTubKmP6pR4w6iXq0/VZpfT6u7lj+OdhrbKR2XPwyvGop9rJw/rDJZsoyolD89doJbyEWgPzhn4HBp5aQ/njUoyA40pD/4KVvisXOhP0uyS35w+64/rysbuvJ0nD/BLeMaDDKBP+9NMYoMwpo/Nn/SN5YEiD/jGWzGAteOPxxnRxOlGZA/+8g2T9CiqD+eUqIXIl6nP+x2uUpOD5U/5pk0bv7foD+MlW4oJ0yQP0dXXkKbfY4/olQz/RjjhT8qpUBQgteCP0XxQf1YD5c/7CGP0Dnblj/OgLJAGLyFP+jhfjgrU4s/Oo/bBNbLsD8bYkbavI2bP2xxdjIsf6A/2F3I+BSblz8mC5b4Pu6YP9DhfPXNpLc/1WuD5WJPjD/jihonM4egP0a0SCNN9pc/dbBq/V2DpD/HMxwtr6ypP+HRmQN6Bpw/SG3jMqdpqD8B8qFpbd2MPxclc0Ca5ZY/HZQMgDK7oj/7M4o6Vp6yPwq1THSucJk/Lr4rqdpEnj8Fq24VQECOP3tjGyNm46E/GNwE67Pikz+rB7nvF36gPyMfCtS7V5o/qYqZbzbBoj+Utkfu5125Pwlmh6OCTqA/Pw21kDXApT/CIRczjC64P/rmKuiVvKA/Iz1hj1zDjD/E/aVU1rSfP4oT1aSkcaQ/veeiceWwnD/hbuSGsk6wP1Zr0piJN6Y/B3fxA/tDnz8DMoOluO+hPwr/7D0Fkbc/q08q16kBqD9mYhztmVOgP48SYWP1D5k/DLzzBDAnoj85QyTCq8mZP4LDoqKZ6ZE/7jP2P/kcgz/sLBDExPKoP7ZR0gUIFIc/xFE3kzgHoT+Yt9j7uV6PP9VzpOmQlI0/afTj3khAlD/c+VV/ZaKVP5vHFV01YLg/RQoKPJckgz9HZgzpuJulP0+hDAqCvZI/64RWUu8SqD+P/2N/3ZWSP5aXIwiKPJg/1lMncuEVtT96J0ZdgpSdP3GGZ3djvpQ/Yz0APSGZkT9C0dWRK7CmPxE0AAAADAAAAAZBAAAAClZhbHZlIHR5cGUGQgAAABZWYWx2ZSBvciByaW5nIHNpemUoMjEpBkMAAAAWVmFsdmUgb3IgcmluZyBzaXplKDIzKQZEAAAAFlZhbHZlIG9yIHJpbmcgc2l6ZSgyNSkGRQAAABZWYWx2ZSBvciByaW5nIHNpemUoMjcpBkYAAAASQ2FyZGlhYyBwcm9jZWR1cmVzBkcAAAAFRUYoMSkGSAAAAAVFRigyKQZJAAAABUVGKDMpBkoAAAAGR2VuZGVyBksAAAAQQWdlIGF0IG9wZXJhdGlvbgoPNQAAAA8BAAAGAAAAAAAAAAB+fewuPaLzv3OTC4j3XeK/X4heTNjb+b/1JLsXJh/pv1NZDBGo9dm/pWUIf3vk4b/fjIrJSv/5v3irtRdYv+Y/HjE+TNSx9r+VDp8d1LLkP7Tv+jQBJO+/ma0l5NDX8L+eu8vM/gzWv9Q6jQ+bGuS/Nb2AS2UR6L/ztng7CdHnv6AGP2EkQuS/WxhuC9cE6b/wDy0qK1nrv3A4vx+yJv6/whF3jNRn17/JPOiSJtHSP6z7LztxyOi/Oes+V9QA5r8RhjP+y+jwv2HNUtc169m/j+O+EgMB87/JvUPKpWf0P7OXAT0zBPC/aUPV/GGX5r9qKlCz3Kbjv5A9QGtWP/K/KUS6JoJv+L8WoiKtQwnyv19ObZhDUvs/UO7OCmC9+79XNczz4/fpv42rCJHW9/G/AnVV8g9s2T/I6bCHdXLyvyZGkF5WWNW/W+qV/I5j7T8DlpDeuS3wv33N97V5A9e/l7nQO7Ptyb/hjNGGNJDnv9m3UCPfQeM/G/e3UECA+b9l5TGA+lLYP1Lv/XYsuOS/3Uo7jSmZ3r/Th7qAcPXwv3qh0ko5N/K/j/xSdlTI6L+ICifK5yrUvzOwySPHGPS/Lr899lSV2T/w9lwoLB7yv5TYAxYEhuS/sltr+4UY6r9GCKIpJGHnvyLlkRhlC/S/iObixhFz9L/+sx4Qtsfqv0oqS+rojM6/wRXwYM7i9r/SEOX6f1/3vxu9PE9SIuS/IxAqZ13C6z88KO4o+hzzv/7Qid/re8q/S6vaw2ARzr/EIGx1XNrkv43E/dRhweS/C5+iS6mN2T82XU0BzvPxvz11Lzb01e6/pHJxLDgJ5r8wYuEDR1rzv1yyMQFdvwHAPVhyAmBD+T+3aHkmGsb9v0M+ppOtt+M/LeVy29OO97/Qo0ixFS/av5PO/gdwTvG/Iv3fU2bv1b+TSjd4P1rpv9WXEyjGLNi/AEidb8/j6792DxQb3GH2vwu8Fiihb+G/FwcxbWrV6T9iPTvqr83mv6t0+891RuK/bi1KnmlY4b8YaRpTdAztv7ip5xrnAuQ/SbiZKzk25r988KOwglH2P8P7pfEEJrU/9Y0bPLNh9b9mrWjGOfHwv950Zyd/mdU/LqqYoyc57L9OtHuJA4/zv+/L5ZIPJem/4SJIQMce67/kMGgzm7/0v57rtIzHN/K/WPX7t9lp7r/5HZ0MG0Lhv83bsaMo2u2/UObseolMsT+N1/4pxDrkP4TY5fEbfva/U6+2ZgKC07+2Oae3KFrgv8eAeufaAvW/5a7DZl5t+7/xS2X50Rjjv+ydRVW8VOi/cE1Cehi5+L9JqDwHHB79P1tNzpwVfe6/8nCHfIXR4r8Qm+J5qRfyvyXVzT7MZ+i/zuVo27f/6r/OFG+ewsDkv0wrl2l+eO2/c2+swvpH7r+gWFGgAtD/PyJsSqROCfC/5nH7kzny2r8/hSIk6LPpv0UXz63YxfC/nkFzvkUq8r+puCRc7EHwP0wrkHWumN0/cQDQMUR52b9HW8g5UoLzv6uYl522peC/TzOLjN5Y1z+Z2tBkWC32v2FpdP5CJN0/kCictvN04L8AVlwhAp3cv26ic2lkhOG/SVyH41ts5L9fWaCR2oHhP2FG4U90Bqq/tcs6sqi47b8G6VOXkYHtv2kNO/nKzea/wUA0RDKx37/VzioIE47pvzgyH/Au2Nq/m/Xdli8W879Be4rV8UTgP1NLzfwzn+O/dEdmBGLo9L9bpDL2k+zrvyFS5QkuY9Y//stss84yzb833krmwM7gPxWMk66If++/gagXaGKv8L/c+Ch3iffqv+VQvELX85k/kP0H8i9N2L+IW4PN7+znvxQuPCUWjOy/Z7ROQ6Ys7L/pxAjXR8fKP4v+Eoy5/fa/y8o7J6L79L/yGIxHC6eYvykyIL+FyeO/GvGwcsNl5L9DFWsneKDjvxmafWGFMPG/eYKFGnQ967+2TSoey7qbv1h7X7XODOy/A9F+PiJ14r81zAVQPtjsP84MUm4akeq/pa8L4qI71b86G37p5Nn0v4P300dhQOW/65rhgc2H3L91nt6nuYfjvzYAshKzwek/QlkULjAp6b8mi6q2uVbiv4Jm7lsj6PS/UEH3MrGh6L8cz9oqVD3qv6rHE/hGAeS/vCWj1i8m4b877YUW/KDfv6v9sUsQI+a/KggVmIv56L+0BO+Y8x/qv7TDy/SNDwBA73xz9E3N8T9Fw2kxbt3uP8Y0WeRqrvI/0IcewYMI7z9GOIYvmmj2P2s9Dzc3vfU/XMMhFdWK/j8fO9gk5G/7P6ZypCFPCP8/+pf3+Ias8z8Rw2EXtzj6P7tpm64VPO0/JtBTy82D+z+IsoSl3bb5P7YraEH63fI/tsBxkVcT/T/tFfnzBJvsP3sb4u9KKPI/i1Z+78XG/D8/Wx42bNLmP5ctxkQMxvI/ATejSCBd9j81hL0CMTUGQPyS8mfwSPU/iOwk+sRw+D9U8Cb3KQH0Pyz+tbXwavM/qpwcLQ2B7j/7JkdnUkrxP5ko+9iFXPU/oti0M27m6j/VtnbNX8/1PzpeL/o52P8/+jsmKTjz/D+z5Efj2qjxP3d90thzuvc/H/KNfUuM/D/YDQ/FouPsPx1VczFaH+8/0oMTma+K8z9s4TVbATbuP+Ldi6BJyfo/LsSzadMC8z/I25YNKngAQAZkav7BdQBAF9frL8NgAkBjG9g1icD3Py0CcuKqbPA/AY2P7066/T/SXoDlQdEBQMKmirIs5vY/yPHGDyAU9D9F/EhNIvTzP/26W/YwsABAwxTvINVc9j9SIYtte+L3P81ddrDUkfE/l0Y88mY9AEBvE306vFf2P7qAxjtdiec/tIShOwu9+T9nZh6I5Xf1P+Je276ecPY/OuqoqHm9+z8PNgAAAAIAAAAIAAAAAAAAAAAPNwAAAA8BAAAGAAAAAAAAAACvlHLy8kHmP6nK4o+xSNY/SqzIyYfV0D8T9wVH/x3QP2vtbWSRdtQ/ulStrbemzj/+FWe3yhXUP46DbpxNvtc/cVAD+pj43j+GGVCs6xDTP3Dm5ab8wo4/AAAAAAAAAAAAAAAAAAAAAAAAAAAAAAAAAAAAAAAAAAAAAAAAAAAAAAAAAAAAAAAAAAAAAAAAAAAAAAAAAAAAAAAAAAAAAAAAAAAAAAAAAAAAAAAAAAAAAAAAAAAAAAAAAAAAAAAAAAAAAAAAAAAAAAAAAAAAAAAAAAAAAAAAAAAAAAAAAAAAAAAAAAAAAAAAAAAAAAAAAAAAAAAAAAAAAAAAAAAAAAAAAAAAAAAAAAAAAAAAAAAAAAAAAAAAAAAAAAAAAAAAAAAAAAAAAAAAAAAAAAAAAAAAAAAAAAAAAAAAAAAAAAAAAAAAAAAAAAAAAAAAAAAAAAAAAAAAAAAAAAAAAAAAAAAAAAAAAAAAAAAAAAAAAAAAAAAAAAAAAAAAAAAAAAAAAAAAAAAAAAAAAAAAAAAAAAAAAAAAAAAAAAAAAAAAAAAAAAAAAAAAAAAAAAAAAAAAAAAAAAAAAAAAAAAAAAAAAAAAAAAAAAAAAAAAAAAAAAAAAAAAAAAAAAAAAAAAAAAAAAAAAAAAAAAAAAAAAAAAAAAAAAAAAAAAAAAAAAAAAAAAAAAAAAAAAAAAAAAAAAAAAAAAAAAAAAAAAAAAAAAAAAAAAAAAAAAAAAAAAAAAAAAAAAAAAAAAAAAAAAAAAAAAAAAAAAAAAAAAAAAAAAAAAAAAAAAAAAAAAAAAAAAAAAAAAAAAAAAAAAAAAAAAAAAAAAAAAAAAAAAAAAAAAAAAAAAAAAAAAAAAAAAAAAAAAAAAAAAAAAAAAAAAAAAAAAAAAAAAAAAAAAAAAAAAAAAAAAAAAAAAAAAAAAAAAAAAAAAAAAAAAAAAAAAAAAAAAAAAAAAAAAAAAAAAAAAAAAAAAAAAAAAAAAAAAAAAAAAAAAAAAAAAAAAAAAAAAAAAAAAAAAAAAAAAAAAAAAAAAAAAAAAAAAAAAAAAAAAAAAAAAAAAAAAAAAAAAAAAAAAAAAAAAAAAAAAAAAAAAAAAAAAAAAAAAAAAAAAAAAAAAAAAAAAAAAAAAAAAAAAAAAAAAAAAAAAAAAAAAAAAAAAAAAAAAAAAAAAAAAAAAAAAAAAAAAAAAAAAAAAAAAAAAAAAAAAAAAAAAAAAAAAAAAAAAAAAAAAAAAAAAAAAAAAAAAAAAAAAAAAAAAAAAAAAAAAAAAAAAAAAAAAAAAAAAAAAAAAAAAAAAAAAAAAAAAAAAAAAAAAAAAAAAAAAAAAAAAAAAAAAAAAAAAAAAAAAAAAAAAAAAAAAAAAAAAAAAAAAAAAAAAAAAAAAAAAAAAAAAAAAAAAAAAAAAAAAAAAAAAAAAAAAAAAAAAAAAAAAAAAAAAAAAAAAAAAAAAAAAAAAAAAAAAAAAAAAAAAAAAAAAAAAAAAAAAAAAAAAAAAAAAAAAAAAAAAAAAAAAAAAAAAAAAAAAAAAAAAAAAAAAAAAAAAAAAAAAAAAAAAAAAAAAAAAAAAAAAAAAAAAAAAAAAAAAAAAAAAAAAAAAAAAAAAAAAAAAAAAAAAAAAAAAAAAAAAAAAAAAAAAAAAAAAAAAAAAAAAAAAAAAAAAAAAAAAAAAAAAAAAAAAAAAAAAAAAAAAAAAAAAAAAAAAAAAAAAAAAAAAAAAAAAAAAAAAAAAAAAAAAAAAAAAAAAAAAAAAAAAAAAAAAAAAAAAAAAAAAAAAAAAAAAAAAAAAAAAAAAAAAAAAAAAAAAAAAAAAAAAAAAAAAAAAAAAAAAAAAAAAAAAAAAAAAAAAAAAAAAAAAAAAAAAAAAAAAAAAAAAAAAAAAAAAAAAAAAAAAAAAAAAAAAAAAAAAAAAAAAAAAAAAAAAAAAAAAAAAAAAAAAAAAAAAAAAAAAAAAAAAAAAAAAAAAAAAAAAAAAAAAAAAAAAAAAAAAAAAAAAAAAAAAAAAAAAAAAAAAAAAAAAAAAAAAAAAAAAAAAAAAAAAAAAAAAAAAAAAAAAAAAAAAAAAAAAAAAAAAAAAAAAAAAAAAAAAAAAAAAAAAAAAAAAAAAAAAAAAAAAAAAAAAAAAAAAAAAAAAAAAAAAAAAAAAAAAAAAAAAAAAAAAAAAAAAAAAAAAAAAAAAAAAAAAAAAAAAAAAAAAAAAAAAAAAAAAAAAAAAAAAAAAAAAAAAAAAAAAAAAAAAAAAAAAAAAAAAAAAAAAAAAAAAAAAAAAAAAAAAAAAAAAAAAAAAAAAAAAAAAAAAAAAAAAAAAAAAAAAAAAAAAAAAAAAAAAAAAAAAAAAAAAAAAAAAAAAAAAAAAAAAAAAAAAAAAAAAAAAAAAAAAAAAAAAAAAAAAAAAAAAAAAAAAAAAAAAAAAAAAAAAAAAAAAAAAAAAAAAAAAAAAAAAAAAAAAAAAAAAAAAAAAAAAAAAAAAAAAAAAAAAAAAAAAAAAAAAAAAAAAAAAAAAAAAAAAAAAAAAAAAAAAAAAAAAAAAAAAAAAAAAAAAAAAAAAAAAAAAAAAAAAAAAAAAAAAAAAAAAAAAAAAAAAAAAAAAAAAAAAAAAAAAAAAAAAAAAAAAAAAAAAAAAAAAAAAAAAAAAAAAAAAAAAAAAAAAAAAAAAAAAAAAAAAAAAAAAAAAAAAAAAAAAAAAAAAAAAAAAAAAAAAAAAAAAAAAAAAAAAAAAAAAAAAAAAAAAAAAAAAAAAAAAAAAAAAAAAAAAAAAAAAAAAAAAAAAAAAAAAAAAAAAAAAAAAAAAAAAAAAAAAAAAAAAAAAAAAAAAAAAAAAAAAAAAAAAAAAAAAAAAAAAAAAAAAAAAAAAAAAAAAAAAAAAAAAAAAAAAAAAPOAAAAA8BAAAGAAAAAAAAAAAAAAAAAAAAQAAAAAAAAPA/AAAAAAAAAEAAAAAAAADwPwAAAAAAABhAAAAAAAAA8D8AAAAAAAAIQAAAAAAAAAhAAAAAAAAAEEAAAAAAAAAUQAAAAAAAAPA/AAAAAAAA8D8AAAAAAADwPwAAAAAAAPA/AAAAAAAA8D8AAAAAAADwPwAAAAAAAPA/AAAAAAAAAEAAAAAAAAAIQAAAAAAAABBAAAAAAAAA8D8AAAAAAAAIQAAAAAAAAPA/AAAAAAAA8D8AAAAAAAAIQAAAAAAAAPA/AAAAAAAAAEAAAAAAAAAQQAAAAAAAAPA/AAAAAAAA8D8AAAAAAADwPwAAAAAAABBAAAAAAAAAAEAAAAAAAAAAQAAAAAAAAAhAAAAAAAAACEAAAAAAAAAAQAAAAAAAAAhAAAAAAAAACEAAAAAAAAAAQAAAAAAAAPA/AAAAAAAACEAAAAAAAAAAQAAAAAAAAPA/AAAAAAAACEAAAAAAAADwPwAAAAAAAABAAAAAAAAAEEAAAAAAAAAQQAAAAAAAAABAAAAAAAAA8D8AAAAAAADwPwAAAAAAAPA/AAAAAAAA8D8AAAAAAAAIQAAAAAAAAPA/AAAAAAAACEAAAAAAAAAIQAAAAAAAAPA/AAAAAAAA8D8AAAAAAADwPwAAAAAAAPA/AAAAAAAAAEAAAAAAAAAAQAAAAAAAAPA/AAAAAAAA8D8AAAAAAAAAQAAAAAAAAPA/AAAAAAAAAEAAAAAAAAAAQAAAAAAAAPA/AAAAAAAA8D8AAAAAAADwPwAAAAAAAAhAAAAAAAAAAEAAAAAAAAAAQAAAAAAAAAhAAAAAAAAA8D8AAAAAAAAIQAAAAAAAABBAAAAAAAAAAEAAAAAAAAAAQAAAAAAAAABAAAAAAAAACEAAAAAAAADwPwAAAAAAAPA/AAAAAAAA8D8AAAAAAADwPwAAAAAAAPA/AAAAAAAA8D8AAAAAAADwPwAAAAAAAPA/AAAAAAAAEEAAAAAAAADwPwAAAAAAAPA/AAAAAAAA8D8AAAAAAADwPwAAAAAAAABAAAAAAAAA8D8AAAAAAAAAQAAAAAAAABhAAAAAAAAAEEAAAAAAAAAAQAAAAAAAAAhAAAAAAAAA8D8AAAAAAADwPwAAAAAAAABAAAAAAAAA8D8AAAAAAAAAQAAAAAAAAPA/AAAAAAAA8D8AAAAAAADwPwAAAAAAAPA/AAAAAAAAAEAAAAAAAAAIQAAAAAAAAAhAAAAAAAAAAEAAAAAAAADwPwAAAAAAAAhAAAAAAAAACEAAAAAAAADwPwAAAAAAAPA/AAAAAAAACEAAAAAAAAAAQAAAAAAAAABAAAAAAAAA8D8AAAAAAADwPwAAAAAAAPA/AAAAAAAA8D8AAAAAAADwPwAAAAAAAPA/AAAAAAAA8D8AAAAAAAAUQAAAAAAAAPA/AAAAAAAA8D8AAAAAAADwPwAAAAAAAPA/AAAAAAAA8D8AAAAAAAAIQAAAAAAAAABAAAAAAAAAAEAAAAAAAADwPwAAAAAAAPA/AAAAAAAACEAAAAAAAAAAQAAAAAAAAABAAAAAAAAA8D8AAAAAAADwPwAAAAAAAPA/AAAAAAAA8D8AAAAAAAAAQAAAAAAAAAhAAAAAAAAA8D8AAAAAAADwPwAAAAAAAPA/AAAAAAAA8D8AAAAAAADwPwAAAAAAAAhAAAAAAAAAAEAAAAAAAAAAQAAAAAAAAPA/AAAAAAAA8D8AAAAAAADwPwAAAAAAAABAAAAAAAAAAEAAAAAAAAAAQAAAAAAAAPA/AAAAAAAA8D8AAAAAAADwPwAAAAAAAABAAAAAAAAA8D8AAAAAAADwPwAAAAAAAPA/AAAAAAAA8D8AAAAAAAAAQAAAAAAAAABAAAAAAAAA8D8AAAAAAAAAQAAAAAAAAPA/AAAAAAAA8D8AAAAAAADwPwAAAAAAAPA/AAAAAAAA8D8AAAAAAAAAQAAAAAAAAPA/AAAAAAAA8D8AAAAAAAAQQAAAAAAAAPA/AAAAAAAA8D8AAAAAAADwPwAAAAAAAPA/AAAAAAAA8D8AAAAAAADwPwAAAAAAAAhAAAAAAAAA8D8AAAAAAADwPwAAAAAAAPA/AAAAAAAA8D8AAAAAAADwPwAAAAAAAPA/AAAAAAAA8D8AAAAAAADwPwAAAAAAAPA/AAAAAAAA8D8AAAAAAADwPwAAAAAAAABAAAAAAAAA8D8AAAAAAADwPwAAAAAAAPA/AAAAAAAA8D8AAAAAAADwPwAAAAAAAPA/AAAAAAAA8D8AAAAAAADwPwAAAAAAAABAAAAAAAAAAEAAAAAAAADwPwAAAAAAAPA/AAAAAAAA8D8AAAAAAADwPwAAAAAAAPA/AAAAAAAACEAAAAAAAADwPwAAAAAAAPA/AAAAAAAA8D8AAAAAAADwPwAAAAAAAPA/AAAAAAAA8D8AAAAAAAAAQAAAAAAAAPA/AAAAAAAA8D8AAAAAAADwPwAAAAAAAPA/AAAAAAAA8D8AAAAAAADwPwAAAAAAAPA/AAAAAAAA8D8AAAAAAADwPwAAAAAAAABAAAAAAAAAAEAAAAAAAADwPwAAAAAAAPA/AAAAAAAA8D8AAAAAAADwPwAAAAAAAPA/AAAAAAAA8D8AAAAAAADwPwAAAAAAAABAAAAAAAAA8D8AAAAAAADwPwAAAAAAAPA/AAAAAAAAAEAAAAAAAADwPwAAAAAAAPA/AAAAAAAA8D8AAAAAAADwPwAAAAAAAPA/AAAAAAAA8D8AAAAAAADwPwAAAAAAAPA/AAAAAAAA8D8AAAAAAADwPwAAAAAAAPA/AAAAAAAA8D8AAAAAAADwPwAAAAAAAPA/AAAAAAAA8D8AAAAAAADwPwAAAAAAAPA/AAAAAAAAAEAPOQAAAA8BAAAGAAAAAAAAAAAAAAAAAADwPwAAAAAAAPA/AAAAAAAA8D8AAAAAAADwPwAAAAAAAPA/AAAAAAAA8D8AAAAAAADwPwAAAAAAAPA/AAAAAAAA8D8AAAAAAADwPwAAAAAAAPA/AAAAAAAA8D8AAAAAAADwPwAAAAAAAPA/AAAAAAAA8D8AAAAAAADwPwAAAAAAAPA/AAAAAAAA8D8AAAAAAADwPwAAAAAAAPA/AAAAAAAA8D8AAAAAAADwPwAAAAAAAPA/AAAAAAAA8D8AAAAAAADwPwAAAAAAAPA/AAAAAAAA8D8AAAAAAADwPwAAAAAAAPA/AAAAAAAA8D8AAAAAAADwPwAAAAAAAPA/AAAAAAAA8D8AAAAAAADwPwAAAAAAAPA/AAAAAAAA8D8AAAAAAADwPwAAAAAAAPA/AAAAAAAA8D8AAAAAAADwPwAAAAAAAPA/AAAAAAAA8D8AAAAAAADwPwAAAAAAAPA/AAAAAAAA8D8AAAAAAADwPwAAAAAAAPA/AAAAAAAA8D8AAAAAAADwPwAAAAAAAPA/AAAAAAAA8D8AAAAAAADwPwAAAAAAAPA/AAAAAAAA8D8AAAAAAADwPwAAAAAAAPA/AAAAAAAA8D8AAAAAAADwPwAAAAAAAPA/AAAAAAAA8D8AAAAAAADwPwAAAAAAAPA/AAAAAAAA8D8AAAAAAADwPwAAAAAAAPA/AAAAAAAA8D8AAAAAAADwPwAAAAAAAPA/AAAAAAAA8D8AAAAAAADwPwAAAAAAAPA/AAAAAAAA8D8AAAAAAADwPwAAAAAAAPA/AAAAAAAA8D8AAAAAAADwPwAAAAAAAPA/AAAAAAAA8D8AAAAAAADwPwAAAAAAAPA/AAAAAAAA8D8AAAAAAADwPwAAAAAAAPA/AAAAAAAA8D8AAAAAAADwPwAAAAAAAPA/AAAAAAAA8D8AAAAAAADwPwAAAAAAAPA/AAAAAAAA8D8AAAAAAADwPwAAAAAAAPA/AAAAAAAA8D8AAAAAAADwPwAAAAAAAPA/AAAAAAAA8D8AAAAAAADwPwAAAAAAAPA/AAAAAAAA8D8AAAAAAADwPwAAAAAAAPA/AAAAAAAA8D8AAAAAAADwPwAAAAAAAPA/AAAAAAAA8D8AAAAAAADwPwAAAAAAAPA/AAAAAAAA8D8AAAAAAADwPwAAAAAAAPA/AAAAAAAA8D8AAAAAAADwPwAAAAAAAPA/AAAAAAAA8D8AAAAAAADwPwAAAAAAAPA/AAAAAAAA8D8AAAAAAADwPwAAAAAAAPA/AAAAAAAA8D8AAAAAAADwPwAAAAAAAPA/AAAAAAAA8D8AAAAAAADwPwAAAAAAAPA/AAAAAAAA8D8AAAAAAADwPwAAAAAAAPA/AAAAAAAA8D8AAAAAAADwPwAAAAAAAPA/AAAAAAAA8D8AAAAAAADwPwAAAAAAAPA/AAAAAAAA8D8AAAAAAADwPwAAAAAAAPA/AAAAAAAA8D8AAAAAAADwPwAAAAAAAPA/AAAAAAAA8D8AAAAAAADwPwAAAAAAAPA/AAAAAAAA8D8AAAAAAADwPwAAAAAAAPA/AAAAAAAA8D8AAAAAAADwPwAAAAAAAPA/AAAAAAAA8D8AAAAAAADwPwAAAAAAAPA/AAAAAAAA8D8AAAAAAADwPwAAAAAAAPA/AAAAAAAA8D8AAAAAAADwPwAAAAAAAPA/AAAAAAAA8D8AAAAAAADwPwAAAAAAAPA/AAAAAAAA8D8AAAAAAADwPwAAAAAAAPA/AAAAAAAA8D8AAAAAAADwPwAAAAAAAPA/AAAAAAAA8D8AAAAAAADwPwAAAAAAAPA/AAAAAAAA8D8AAAAAAADwPwAAAAAAAPA/AAAAAAAA8D8AAAAAAADwPwAAAAAAAPA/AAAAAAAA8D8AAAAAAADwPwAAAAAAAPA/AAAAAAAA8D8AAAAAAADwPwAAAAAAAPA/AAAAAAAA8D8AAAAAAADwPwAAAAAAAPA/AAAAAAAA8D8AAAAAAADwPwAAAAAAAPA/AAAAAAAA8D8AAAAAAADwPwAAAAAAAPA/AAAAAAAA8D8AAAAAAADwPwAAAAAAAPA/AAAAAAAA8D8AAAAAAADwPwAAAAAAAPA/AAAAAAAA8D8AAAAAAADwPwAAAAAAAPA/AAAAAAAA8D8AAAAAAADwPwAAAAAAAPA/AAAAAAAA8D8AAAAAAADwPwAAAAAAAPA/AAAAAAAA8D8AAAAAAADwPwAAAAAAAPA/AAAAAAAA8D8AAAAAAADwPwAAAAAAAPA/AAAAAAAA8D8AAAAAAADwPwAAAAAAAPA/AAAAAAAA8D8AAAAAAADwPwAAAAAAAPA/AAAAAAAA8D8AAAAAAADwPwAAAAAAAPA/AAAAAAAA8D8AAAAAAADwPwAAAAAAAPA/AAAAAAAA8D8AAAAAAADwPwAAAAAAAPA/AAAAAAAA8D8AAAAAAADwPwAAAAAAAPA/AAAAAAAA8D8AAAAAAADwPwAAAAAAAPA/AAAAAAAA8D8AAAAAAADwPwAAAAAAAPA/AAAAAAAA8D8AAAAAAADwPwAAAAAAAPA/AAAAAAAA8D8AAAAAAADwPwAAAAAAAPA/AAAAAAAA8D8AAAAAAADwPwAAAAAAAPA/AAAAAAAA8D8AAAAAAADwPwAAAAAAAPA/AAAAAAAA8D8AAAAAAADwPwAAAAAAAPA/AAAAAAAA8D8AAAAAAADwPwAAAAAAAPA/AAAAAAAA8D8AAAAAAADwPwAAAAAAAPA/AAAAAAAA8D8AAAAAAADwPwAAAAAAAPA/AAAAAAAA8D8AAAAAAADwPwAAAAAAAPA/AAAAAAAA8D8AAAAAAADwPwAAAAAAAPA/AAAAAAAA8D8AAAAAAADwPwAAAAAAAPA/AAAAAAAA8D8HOgAAAAICAAAADwEAAAwAAAAABgAAAAAAAAAAAAAAAAAAAAAAAAAAAAAAAAAAAAAAAAAAAAAAAAAAAAAAAAAAAAAAAAAAAAAAAAAAAAAAAAAAAAAAAAAAAAAAAAAAAAAAAAAAAAAAAAAAAAAAAAAAAAAAAAAAAAAAAAAAAAAAAAAA8D8AAAAAAAAAAAAAAAAAAAAAAAAAAAAAAAAAAAAAAAAAAAAAAAAAAPA/AAAAAAAAAAAAAAAAAAAAAAAAAAAAAPA/AAAAAADAUUAAAAAAAAAAAAAAAAAAAAAAAAAAAAAA8D8AAAAAAAAAAAAAAAAAAAAAAAAAAAAAAAAAAAAAAAAAAAAAAAAAAPA/AAAAAAAAAAAAAAAAAAAAAAAAAAAAAPA/AAAAAACATEAAAAAAAAAAAAAAAAAAAAAAAAAAAAAAAAAAAAAAAAAAAAAAAAAAAPA/AAAAAAAAAAAAAAAAAADwPwAAAAAAAPA/AAAAAAAAAAAAAAAAAAAAAAAAAAAAAAAAAAAAAAAAVEAAAAAAAAAAAAAAAAAAAAAAAAAAAAAAAAAAAAAAAAAAAAAAAAAAAPA/AAAAAAAAAAAAAAAAAADwPwAAAAAAAPA/AAAAAAAAAAAAAAAAAAAAAAAAAAAAAAAAAAAAAACAUEAAAAAAAAAAAAAAAAAAAAAAAAAAAAAAAAAAAAAAAADwPwAAAAAAAAAAAAAAAAAAAAAAAAAAAADwPwAAAAAAAPA/AAAAAAAAAAAAAAAAAAAAAAAAAAAAAAAAAAAAAAAAU0AAAAAAAAAAAAAAAAAAAAAAAAAAAAAAAAAAAAAAAADwPwAAAAAAAAAAAAAAAAAAAAAAAAAAAADwPwAAAAAAAAAAAAAAAAAA8D8AAAAAAAAAAAAAAAAAAAAAAAAAAADAUUAAAAAAAAAAAAAAAAAAAAAAAAAAAAAAAAAAAAAAAADwPwAAAAAAAAAAAAAAAAAAAAAAAAAAAAAAAAAAAAAAAPA/AAAAAAAAAAAAAAAAAAAAAAAAAAAAAPA/AAAAAADAUUAAAAAAAAAAAAAAAAAAAAAAAAAAAAAAAAAAAAAAAAAAAAAAAAAAAPA/AAAAAAAAAAAAAAAAAADwPwAAAAAAAPA/AAAAAAAAAAAAAAAAAAAAAAAAAAAAAAAAAAAAAADAU0AAAAAAAAAAAAAAAAAAAAAAAAAAAAAAAAAAAAAAAADwPwAAAAAAAAAAAAAAAAAAAAAAAAAAAADwPwAAAAAAAPA/AAAAAAAAAAAAAAAAAAAAAAAAAAAAAAAAAAAAAADAUkAAAAAAAAAAAAAAAAAAAAAAAAAAAAAAAAAAAAAAAADwPwAAAAAAAAAAAAAAAAAAAAAAAAAAAADwPwAAAAAAAPA/AAAAAAAAAAAAAAAAAAAAAAAAAAAAAPA/AAAAAAAAU0AAAAAAAAAAAAAAAAAAAAAAAAAAAAAAAAAAAAAAAADwPwAAAAAAAAAAAAAAAAAAAAAAAAAAAADwPwAAAAAAAAAAAAAAAAAA8D8AAAAAAAAAAAAAAAAAAPA/AAAAAADAUkAAAAAAAAAAAAAAAAAAAAAAAAAAAAAA8D8AAAAAAAAAAAAAAAAAAAAAAAAAAAAAAAAAAAAAAADwPwAAAAAAAPA/AAAAAAAAAAAAAAAAAAAAAAAAAAAAAPA/AAAAAABAU0AAAAAAAAAAAAAAAAAAAAAAAAAAAAAAAAAAAAAAAADwPwAAAAAAAAAAAAAAAAAAAAAAAAAAAAAAAAAAAAAAAAAAAAAAAAAA8D8AAAAAAAAAAAAAAAAAAAAAAAAAAACATEAAAAAAAAAAAAAAAAAAAAAAAAAAAAAA8D8AAAAAAAAAAAAAAAAAAAAAAAAAAAAAAAAAAAAAAAAAAAAAAAAAAPA/AAAAAAAAAAAAAAAAAAAAAAAAAAAAAAAAAAAAAADAUkAAAAAAAAAAAAAAAAAAAAAAAAAAAAAAAAAAAAAAAADwPwAAAAAAAAAAAAAAAAAAAAAAAAAAAADwPwAAAAAAAPA/AAAAAAAAAAAAAAAAAAAAAAAAAAAAAAAAAAAAAABAU0AAAAAAAAAAAAAAAAAAAAAAAAAAAAAAAAAAAAAAAAAAAAAAAAAAAPA/AAAAAAAAAAAAAAAAAADwPwAAAAAAAAAAAAAAAAAA8D8AAAAAAAAAAAAAAAAAAAAAAAAAAAAAUUAAAAAAAAAAAAAAAAAAAAAAAAAAAAAAAAAAAAAAAADwPwAAAAAAAAAAAAAAAAAAAAAAAAAAAADwPwAAAAAAAAAAAAAAAAAA8D8AAAAAAAAAAAAAAAAAAPA/AAAAAAAATkAAAAAAAAAAAAAAAAAAAAAAAAAAAAAAAAAAAAAAAADwPwAAAAAAAAAAAAAAAAAAAAAAAAAAAAAAAAAAAAAAAPA/AAAAAAAAAAAAAAAAAAAAAAAAAAAAAAAAAAAAAAAAUUAAAAAAAAAAAAAAAAAAAAAAAAAAAAAAAAAAAAAAAADwPwAAAAAAAAAAAAAAAAAAAAAAAAAAAADwPwAAAAAAAAAAAAAAAAAA8D8AAAAAAAAAAAAAAAAAAAAAAAAAAADAUEAAAAAAAAAAAAAAAAAAAAAAAAAAAAAA8D8AAAAAAAAAAAAAAAAAAAAAAAAAAAAAAAAAAAAAAAAAAAAAAAAAAPA/AAAAAAAAAAAAAAAAAAAAAAAAAAAAAPA/AAAAAACAUkAAAAAAAAAAAAAAAAAAAAAAAAAAAAAAAAAAAAAAAADwPwAAAAAAAAAAAAAAAAAAAAAAAAAAAAAAAAAAAAAAAPA/AAAAAAAAAAAAAAAAAAAAAAAAAAAAAAAAAAAAAACAS0AAAAAAAAAAAAAAAAAAAAAAAAAAAAAAAAAAAAAAAADwPwAAAAAAAAAAAAAAAAAAAAAAAAAAAAAAAAAAAAAAAPA/AAAAAAAAAAAAAAAAAAAAAAAAAAAAAPA/AAAAAAAAUEAAAAAAAAAAAAAAAAAAAAAAAAAAAAAA8D8AAAAAAAAAAAAAAAAAAAAAAAAAAAAAAAAAAAAAAAAAAAAAAAAAAPA/AAAAAAAAAAAAAAAAAAAAAAAAAAAAAPA/AAAAAADAUEAAAAAAAAAAAAAAAAAAAAAAAAAAAAAAAAAAAAAAAADwPwAAAAAAAAAAAAAAAAAAAAAAAAAAAAAAAAAAAAAAAAAAAAAAAAAA8D8AAAAAAAAAAAAAAAAAAAAAAAAAAADAU0AAAAAAAAAAAAAAAAAAAAAAAAAAAAAAAAAAAAAAAADwPwAAAAAAAAAAAAAAAAAAAAAAAAAAAADwPwAAAAAAAPA/AAAAAAAAAAAAAAAAAAAAAAAAAAAAAAAAAAAAAACAUUAAAAAAAAAAAAAAAAAAAAAAAAAAAAAAAAAAAAAAAADwPwAAAAAAAAAAAAAAAAAAAAAAAAAAAAAAAAAAAAAAAAAAAAAAAAAA8D8AAAAAAAAAAAAAAAAAAAAAAAAAAAAAT0AAAAAAAAAAAAAAAAAAAAAAAAAAAAAAAAAAAAAAAADwPwAAAAAAAAAAAAAAAAAAAAAAAAAAAAAAAAAAAAAAAPA/AAAAAAAAAAAAAAAAAAAAAAAAAAAAAPA/AAAAAADAUEAAAAAAAAAAAAAAAAAAAAAAAAAAAAAAAAAAAAAAAADwPwAAAAAAAAAAAAAAAAAAAAAAAAAAAADwPwAAAAAAAPA/AAAAAAAAAAAAAAAAAAAAAAAAAAAAAAAAAAAAAACAUkAAAAAAAAAAAAAAAAAAAAAAAAAAAAAAAAAAAAAAAAAAAAAAAAAAAPA/AAAAAAAAAAAAAAAAAADwPwAAAAAAAAAAAAAAAAAAAAAAAAAAAADwPwAAAAAAAAAAAAAAAAAAUUAAAAAAAAAAAAAAAAAAAAAAAAAAAAAAAAAAAAAAAADwPwAAAAAAAAAAAAAAAAAAAAAAAAAAAAAAAAAAAAAAAPA/AAAAAAAAAAAAAAAAAAAAAAAAAAAAAPA/AAAAAACATkAAAAAAAAAAAAAAAAAAAAAAAAAAAAAA8D8AAAAAAAAAAAAAAAAAAAAAAAAAAAAAAAAAAAAAAADwPwAAAAAAAPA/AAAAAAAAAAAAAAAAAAAAAAAAAAAAAPA/AAAAAAAATUAAAAAAAAAAAAAAAAAAAAAAAAAAAAAAAAAAAAAAAADwPwAAAAAAAAAAAAAAAAAAAAAAAAAAAAAAAAAAAAAAAPA/AAAAAAAAAAAAAAAAAAAAAAAAAAAAAAAAAAAAAABAUUAAAAAAAAAAAAAAAAAAAAAAAAAAAAAA8D8AAAAAAAAAAAAAAAAAAAAAAAAAAAAAAAAAAAAAAADwPwAAAAAAAPA/AAAAAAAAAAAAAAAAAAAAAAAAAAAAAPA/AAAAAACAU0AAAAAAAAAAAAAAAAAAAAAAAAAAAAAA8D8AAAAAAAAAAAAAAAAAAAAAAAAAAAAAAAAAAAAAAAAAAAAAAAAAAPA/AAAAAAAAAAAAAAAAAAAAAAAAAAAAAPA/AAAAAAAAUUAAAAAAAAAAAAAAAAAAAAAAAAAAAAAAAAAAAAAAAADwPwAAAAAAAAAAAAAAAAAAAAAAAAAAAAAAAAAAAAAAAPA/AAAAAAAAAAAAAAAAAAAAAAAAAAAAAAAAAAAAAADAUkAAAAAAAAAAAAAAAAAAAAAAAAAAAAAAAAAAAAAAAAAAAAAAAAAAAPA/AAAAAAAAAAAAAAAAAAAAAAAAAAAAAPA/AAAAAAAAAAAAAAAAAAAAAAAAAAAAAAAAAAAAAAAAU0AAAAAAAAAAAAAAAAAAAAAAAAAAAAAAAAAAAAAAAADwPwAAAAAAAAAAAAAAAAAAAAAAAAAAAADwPwAAAAAAAPA/AAAAAAAAAAAAAAAAAAAAAAAAAAAAAAAAAAAAAAAAUUAAAAAAAAAAAAAAAAAAAAAAAAAAAAAAAAAAAAAAAADwPwAAAAAAAAAAAAAAAAAAAAAAAAAAAADwPwAAAAAAAPA/AAAAAAAAAAAAAAAAAAAAAAAAAAAAAAAAAAAAAAAAUkAAAAAAAAAAAAAAAAAAAAAAAAAAAAAAAAAAAAAAAADwPwAAAAAAAAAAAAAAAAAAAAAAAAAAAAAAAAAAAAAAAPA/AAAAAAAAAAAAAAAAAAAAAAAAAAAAAPA/AAAAAAAAT0AAAAAAAAAAAAAAAAAAAAAAAAAAAAAAAAAAAAAAAADwPwAAAAAAAAAAAAAAAAAAAAAAAAAAAAAAAAAAAAAAAPA/AAAAAAAAAAAAAAAAAAAAAAAAAAAAAAAAAAAAAABAVEAAAAAAAAAAAAAAAAAAAAAAAAAAAAAAAAAAAAAAAADwPwAAAAAAAAAAAAAAAAAAAAAAAAAAAAAAAAAAAAAAAAAAAAAAAAAA8D8AAAAAAAAAAAAAAAAAAAAAAAAAAAAATEAAAAAAAAAAAAAAAAAAAAAAAAAAAAAAAAAAAAAAAADwPwAAAAAAAAAAAAAAAAAAAAAAAAAAAADwPwAAAAAAAPA/AAAAAAAAAAAAAAAAAAAAAAAAAAAAAAAAAAAAAABAUEAAAAAAAAAAAAAAAAAAAAAAAAAAAAAAAAAAAAAAAAAAAAAAAAAAAPA/AAAAAAAAAAAAAAAAAAAAAAAAAAAAAPA/AAAAAAAAAAAAAAAAAAAAAAAAAAAAAAAAAAAAAAAAUEAAAAAAAAAAAAAAAAAAAAAAAAAAAAAAAAAAAAAAAAAAAAAAAAAAAPA/AAAAAAAAAAAAAAAAAAAAAAAAAAAAAPA/AAAAAAAAAAAAAAAAAAAAAAAAAAAAAAAAAAAAAAAARUAAAAAAAAAAAAAAAAAAAAAAAAAAAAAA8D8AAAAAAAAAAAAAAAAAAAAAAAAAAAAAAAAAAAAAAAAAAAAAAAAAAPA/AAAAAAAAAAAAAAAAAAAAAAAAAAAAAPA/AAAAAAAAU0AAAAAAAAAAAAAAAAAAAAAAAAAAAAAAAAAAAAAAAAAAAAAAAAAAAPA/AAAAAAAAAAAAAAAAAAAAAAAAAAAAAPA/AAAAAAAAAAAAAAAAAAAAAAAAAAAAAAAAAAAAAABAUEAAAAAAAAAAAAAAAAAAAAAAAAAAAAAAAAAAAAAAAADwPwAAAAAAAAAAAAAAAAAAAAAAAAAAAAAAAAAAAAAAAPA/AAAAAAAAAAAAAAAAAAAAAAAAAAAAAAAAAAAAAACAVEAAAAAAAAAAAAAAAAAAAAAAAAAAAAAAAAAAAAAAAADwPwAAAAAAAAAAAAAAAAAAAAAAAAAAAADwPwAAAAAAAPA/AAAAAAAAAAAAAAAAAAAAAAAAAAAAAAAAAAAAAADAU0AAAAAAAAAAAAAAAAAAAAAAAAAAAAAA8D8AAAAAAAAAAAAAAAAAAAAAAAAAAAAAAAAAAAAAAAAAAAAAAAAAAPA/AAAAAAAAAAAAAAAAAAAAAAAAAAAAAPA/AAAAAABAU0AAAAAAAAAAAAAAAAAAAAAAAAAAAAAAAAAAAAAAAADwPwAAAAAAAAAAAAAAAAAAAAAAAAAAAAAAAAAAAAAAAPA/AAAAAAAAAAAAAAAAAAAAAAAAAAAAAAAAAAAAAAAAT0AAAAAAAAAAAAAAAAAAAAAAAAAAAAAA8D8AAAAAAAAAAAAAAAAAAAAAAAAAAAAAAAAAAAAAAADwPwAAAAAAAPA/AAAAAAAAAAAAAAAAAAAAAAAAAAAAAAAAAAAAAABAUEAAAAAAAAAAAAAAAAAAAAAAAAAAAAAAAAAAAAAAAAAAAAAAAAAAAPA/AAAAAAAAAAAAAAAAAADwPwAAAAAAAPA/AAAAAAAAAAAAAAAAAAAAAAAAAAAAAAAAAAAAAABAU0AAAAAAAAAAAAAAAAAAAAAAAAAAAAAAAAAAAAAAAAAAAAAAAAAAAPA/AAAAAAAAAAAAAAAAAAAAAAAAAAAAAPA/AAAAAAAAAAAAAAAAAAAAAAAAAAAAAPA/AAAAAACAUEAAAAAAAAAAAAAAAAAAAAAAAAAAAAAAAAAAAAAAAADwPwAAAAAAAAAAAAAAAAAAAAAAAAAAAAAAAAAAAAAAAAAAAAAAAAAA8D8AAAAAAAAAAAAAAAAAAPA/AAAAAAAAUUAAAAAAAAAAAAAAAAAAAAAAAAAAAAAA8D8AAAAAAAAAAAAAAAAAAAAAAAAAAAAAAAAAAAAAAAAAAAAAAAAAAPA/AAAAAAAAAAAAAAAAAAAAAAAAAAAAAPA/AAAAAACAU0AAAAAAAAAAAAAAAAAAAAAAAAAAAAAAAAAAAAAAAAAAAAAAAAAAAPA/AAAAAAAAAAAAAAAAAAAAAAAAAAAAAAAAAAAAAAAA8D8AAAAAAAAAAAAAAAAAAAAAAAAAAABAVkAAAAAAAAAAAAAAAAAAAAAAAAAAAAAAAAAAAAAAAADwPwAAAAAAAAAAAAAAAAAAAAAAAAAAAAAAAAAAAAAAAPA/AAAAAAAAAAAAAAAAAAAAAAAAAAAAAAAAAAAAAABAU0AAAAAAAAAAAAAAAAAAAAAAAAAAAAAAAAAAAAAAAAAAAAAAAAAAAPA/AAAAAAAAAAAAAAAAAAAAAAAAAAAAAPA/AAAAAAAAAAAAAAAAAAAAAAAAAAAAAAAAAAAAAACATkAAAAAAAAAAAAAAAAAAAAAAAAAAAAAAAAAAAAAAAAAAAAAAAAAAAPA/AAAAAAAAAAAAAAAAAADwPwAAAAAAAAAAAAAAAAAA8D8AAAAAAAAAAAAAAAAAAAAAAAAAAACAT0AAAAAAAAAAAAAAAAAAAAAAAAAAAAAA8D8AAAAAAAAAAAAAAAAAAAAAAAAAAAAAAAAAAAAAAAAAAAAAAAAAAAAAAAAAAAAAAAAAAAAAAADwPwAAAAAAAPA/AAAAAAAAT0AAAAAAAAAAAAAAAAAAAAAAAAAAAAAA8D8AAAAAAAAAAAAAAAAAAAAAAAAAAAAAAAAAAAAAAAAAAAAAAAAAAPA/AAAAAAAAAAAAAAAAAAAAAAAAAAAAAPA/AAAAAABAUEAAAAAAAAAAAAAAAAAAAAAAAAAAAAAAAAAAAAAAAAAAAAAAAAAAAPA/AAAAAAAAAAAAAAAAAAAAAAAAAAAAAPA/AAAAAAAAAAAAAAAAAAAAAAAAAAAAAPA/AAAAAACAUUAAAAAAAAAAAAAAAAAAAAAAAAAAAAAAAAAAAAAAAAAAAAAAAAAAAPA/AAAAAAAAAAAAAAAAAADwPwAAAAAAAPA/AAAAAAAAAAAAAAAAAAAAAAAAAAAAAAAAAAAAAADAUUAAAAAAAAAAAAAAAAAAAAAAAAAAAAAAAAAAAAAAAADwPwAAAAAAAAAAAAAAAAAAAAAAAAAAAADwPwAAAAAAAPA/AAAAAAAAAAAAAAAAAAAAAAAAAAAAAAAAAAAAAABAUUAAAAAAAAAAAAAAAAAAAAAAAAAAAAAA8D8AAAAAAAAAAAAAAAAAAAAAAAAAAAAAAAAAAAAAAAAAAAAAAAAAAAAAAAAAAAAAAAAAAAAAAADwPwAAAAAAAAAAAAAAAAAAQ0AAAAAAAAAAAAAAAAAAAAAAAAAAAAAAAAAAAAAAAAAAAAAAAAAAAAAAAAAAAAAA8D8AAAAAAAAAAAAAAAAAAAAAAAAAAAAA8D8AAAAAAAAAAAAAAAAAAAAAAAAAAADAVEAAAAAAAAAAAAAAAAAAAAAAAAAAAAAAAAAAAAAAAAAAAAAAAAAAAPA/AAAAAAAAAAAAAAAAAADwPwAAAAAAAPA/AAAAAAAAAAAAAAAAAAAAAAAAAAAAAAAAAAAAAAAAU0AAAAAAAAAAAAAAAAAAAAAAAAAAAAAA8D8AAAAAAAAAAAAAAAAAAAAAAAAAAAAAAAAAAAAAAAAAAAAAAAAAAPA/AAAAAAAAAAAAAAAAAAAAAAAAAAAAAPA/AAAAAAAATkAAAAAAAAAAAAAAAAAAAAAAAAAAAAAAAAAAAAAAAADwPwAAAAAAAAAAAAAAAAAAAAAAAAAAAAAAAAAAAAAAAPA/AAAAAAAAAAAAAAAAAAAAAAAAAAAAAAAAAAAAAAAAU0AAAAAAAAAAAAAAAAAAAAAAAAAAAAAAAAAAAAAAAADwPwAAAAAAAAAAAAAAAAAAAAAAAAAAAADwPwAAAAAAAPA/AAAAAAAAAAAAAAAAAAAAAAAAAAAAAPA/AAAAAACAUEAAAAAAAAAAAAAAAAAAAAAAAAAAAAAAAAAAAAAAAAAAAAAAAAAAAPA/AAAAAAAAAAAAAAAAAAAAAAAAAAAAAAAAAAAAAAAA8D8AAAAAAAAAAAAAAAAAAAAAAAAAAAAAPUAAAAAAAAAAAAAAAAAAAAAAAAAAAAAAAAAAAAAAAAAAAAAAAAAAAPA/AAAAAAAAAAAAAAAAAAAAAAAAAAAAAPA/AAAAAAAAAAAAAAAAAAAAAAAAAAAAAAAAAAAAAAAAPUAAAAAAAAAAAAAAAAAAAAAAAAAAAAAAAAAAAAAAAAAAAAAAAAAAAAAAAAAAAAAA8D8AAAAAAAAAAAAAAAAAAPA/AAAAAAAAAAAAAAAAAAAAAAAAAAAAAAAAAAAAAACASEAAAAAAAAAAAAAAAAAAAAAAAAAAAAAAAAAAAAAAAADwPwAAAAAAAAAAAAAAAAAAAAAAAAAAAAAAAAAAAAAAAPA/AAAAAAAAAAAAAAAAAAAAAAAAAAAAAPA/AAAAAABAVEAAAAAAAAAAAAAAAAAAAAAAAAAAAAAA8D8AAAAAAAAAAAAAAAAAAAAAAAAAAAAAAAAAAAAAAADwPwAAAAAAAPA/AAAAAAAAAAAAAAAAAAAAAAAAAAAAAPA/AAAAAABAUkAAAAAAAAAAAAAAAAAAAAAAAAAAAAAAAAAAAAAAAADwPwAAAAAAAAAAAAAAAAAAAAAAAAAAAAAAAAAAAAAAAPA/AAAAAAAAAAAAAAAAAAAAAAAAAAAAAPA/AAAAAABAUEAAAAAAAAAAAAAAAAAAAAAAAAAAAAAAAAAAAAAAAADwPwAAAAAAAAAAAAAAAAAAAAAAAAAAAADwPwAAAAAAAPA/AAAAAAAAAAAAAAAAAAAAAAAAAAAAAAAAAAAAAADAUEAAAAAAAAAAAAAAAAAAAAAAAAAAAAAA8D8AAAAAAAAAAAAAAAAAAAAAAAAAAAAAAAAAAAAAAAAAAAAAAAAAAPA/AAAAAAAAAAAAAAAAAAAAAAAAAAAAAPA/AAAAAACAT0AAAAAAAAAAAAAAAAAAAAAAAAAAAAAAAAAAAAAAAAAAAAAAAAAAAPA/AAAAAAAAAAAAAAAAAADwPwAAAAAAAPA/AAAAAAAAAAAAAAAAAAAAAAAAAAAAAAAAAAAAAAAAT0AAAAAAAAAAAAAAAAAAAAAAAAAAAAAAAAAAAAAAAAAAAAAAAAAAAPA/AAAAAAAAAAAAAAAAAAAAAAAAAAAAAPA/AAAAAAAAAAAAAAAAAAAAAAAAAAAAAAAAAAAAAAAAVEAAAAAAAAAAAAAAAAAAAAAAAAAAAAAAAAAAAAAAAADwPwAAAAAAAAAAAAAAAAAAAAAAAAAAAAAAAAAAAAAAAPA/AAAAAAAAAAAAAAAAAAAAAAAAAAAAAAAAAAAAAAAATUAAAAAAAAAAAAAAAAAAAAAAAAAAAAAAAAAAAAAAAAAAAAAAAAAAAPA/AAAAAAAAAAAAAAAAAADwPwAAAAAAAPA/AAAAAAAAAAAAAAAAAAAAAAAAAAAAAAAAAAAAAACAVUAAAAAAAAAAAAAAAAAAAAAAAAAAAAAAAAAAAAAAAAAAAAAAAAAAAPA/AAAAAAAAAAAAAAAAAADwPwAAAAAAAPA/AAAAAAAAAAAAAAAAAAAAAAAAAAAAAAAAAAAAAAAAUUAAAAAAAAAAAAAAAAAAAAAAAAAAAAAAAAAAAAAAAAAAAAAAAAAAAPA/AAAAAAAAAAAAAAAAAAAAAAAAAAAAAPA/AAAAAAAAAAAAAAAAAAAAAAAAAAAAAAAAAAAAAACAUUAAAAAAAAAAAAAAAAAAAAAAAAAAAAAAAAAAAAAAAAAAAAAAAAAAAPA/AAAAAAAAAAAAAAAAAAAAAAAAAAAAAPA/AAAAAAAAAAAAAAAAAAAAAAAAAAAAAAAAAAAAAAAAR0AAAAAAAAAAAAAAAAAAAAAAAAAAAAAAAAAAAAAAAAAAAAAAAAAAAPA/AAAAAAAAAAAAAAAAAAAAAAAAAAAAAPA/AAAAAAAAAAAAAAAAAAAAAAAAAAAAAAAAAAAAAADAU0AAAAAAAAAAAAAAAAAAAAAAAAAAAAAAAAAAAAAAAADwPwAAAAAAAAAAAAAAAAAAAAAAAAAAAAAAAAAAAAAAAPA/AAAAAAAAAAAAAAAAAAAAAAAAAAAAAAAAAAAAAACASkAAAAAAAAAAAAAAAAAAAAAAAAAAAAAAAAAAAAAAAADwPwAAAAAAAAAAAAAAAAAAAAAAAAAAAAAAAAAAAAAAAPA/AAAAAAAAAAAAAAAAAAAAAAAAAAAAAAAAAAAAAAAAVEAAAAAAAAAAAAAAAAAAAAAAAAAAAAAAAAAAAAAAAADwPwAAAAAAAAAAAAAAAAAAAAAAAAAAAAAAAAAAAAAAAPA/AAAAAAAAAAAAAAAAAAAAAAAAAAAAAAAAAAAAAAAATEAAAAAAAAAAAAAAAAAAAAAAAAAAAAAAAAAAAAAAAAAAAAAAAAAAAAAAAAAAAAAA8D8AAAAAAAAAAAAAAAAAAPA/AAAAAAAAAAAAAAAAAAAAAAAAAAAAAAAAAAAAAACATUAAAAAAAAAAAAAAAAAAAAAAAAAAAAAAAAAAAAAAAAAAAAAAAAAAAPA/AAAAAAAAAAAAAAAAAADwPwAAAAAAAAAAAAAAAAAAAAAAAAAAAADwPwAAAAAAAAAAAAAAAACAVEAAAAAAAAAAAAAAAAAAAAAAAAAAAAAAAAAAAAAAAAAAAAAAAAAAAAAAAAAAAAAA8D8AAAAAAAAAAAAAAAAAAAAAAAAAAAAA8D8AAAAAAAAAAAAAAAAAAAAAAAAAAACAR0AAAAAAAAAAAAAAAAAAAAAAAAAAAAAAAAAAAAAAAAAAAAAAAAAAAPA/AAAAAAAAAAAAAAAAAADwPwAAAAAAAPA/AAAAAAAAAAAAAAAAAAAAAAAAAAAAAAAAAAAAAABAUUAAAAAAAAAAAAAAAAAAAAAAAAAAAAAAAAAAAAAAAADwPwAAAAAAAAAAAAAAAAAAAAAAAAAAAADwPwAAAAAAAAAAAAAAAAAA8D8AAAAAAAAAAAAAAAAAAAAAAAAAAADAU0AAAAAAAAAAAAAAAAAAAAAAAAAAAAAAAAAAAAAAAADwPwAAAAAAAAAAAAAAAAAAAAAAAAAAAAAAAAAAAAAAAPA/AAAAAAAAAAAAAAAAAAAAAAAAAAAAAPA/AAAAAAAAS0AAAAAAAAAAAAAAAAAAAAAAAAAAAAAAAAAAAAAAAADwPwAAAAAAAAAAAAAAAAAAAAAAAAAAAADwPwAAAAAAAAAAAAAAAAAA8D8AAAAAAAAAAAAAAAAAAAAAAAAAAACAUUAAAAAAAAAAAAAAAAAAAAAAAAAAAAAAAAAAAAAAAAAAAAAAAAAAAPA/AAAAAAAAAAAAAAAAAADwPwAAAAAAAAAAAAAAAAAA8D8AAAAAAAAAAAAAAAAAAAAAAAAAAADAUkAAAAAAAAAAAAAAAAAAAAAAAAAAAAAAAAAAAAAAAAAAAAAAAAAAAPA/AAAAAAAAAAAAAAAAAAAAAAAAAAAAAPA/AAAAAAAAAAAAAAAAAAAAAAAAAAAAAAAAAAAAAABAUUAAAAAAAAAAAAAAAAAAAAAAAAAAAAAAAAAAAAAAAAAAAAAAAAAAAPA/AAAAAAAAAAAAAAAAAAAAAAAAAAAAAPA/AAAAAAAAAAAAAAAAAAAAAAAAAAAAAAAAAAAAAACAT0AAAAAAAAAAAAAAAAAAAAAAAAAAAAAAAAAAAAAAAADwPwAAAAAAAAAAAAAAAAAAAAAAAAAAAAAAAAAAAAAAAPA/AAAAAAAAAAAAAAAAAAAAAAAAAAAAAAAAAAAAAACAT0AAAAAAAAAAAAAAAAAAAAAAAAAAAAAAAAAAAAAAAAAAAAAAAAAAAPA/AAAAAAAAAAAAAAAAAAAAAAAAAAAAAPA/AAAAAAAAAAAAAAAAAAAAAAAAAAAAAAAAAAAAAADAUUAAAAAAAAAAAAAAAAAAAAAAAAAAAAAAAAAAAAAAAADwPwAAAAAAAAAAAAAAAAAAAAAAAAAAAADwPwAAAAAAAPA/AAAAAAAAAAAAAAAAAAAAAAAAAAAAAAAAAAAAAABAUkAAAAAAAAAAAAAAAAAAAAAAAAAAAAAAAAAAAAAAAADwPwAAAAAAAAAAAAAAAAAAAAAAAAAAAAAAAAAAAAAAAPA/AAAAAAAAAAAAAAAAAAAAAAAAAAAAAPA/AAAAAACAT0AAAAAAAAAAAAAAAAAAAAAAAAAAAAAA8D8AAAAAAAAAAAAAAAAAAAAAAAAAAAAAAAAAAAAAAAAAAAAAAAAAAPA/AAAAAAAAAAAAAAAAAAAAAAAAAAAAAPA/AAAAAACAUEAAAAAAAAAAAAAAAAAAAAAAAAAAAAAAAAAAAAAAAADwPwAAAAAAAAAAAAAAAAAAAAAAAAAAAAAAAAAAAAAAAAAAAAAAAAAAAAAAAAAAAADwPwAAAAAAAAAAAAAAAABAU0AAAAAAAAAAAAAAAAAAAAAAAAAAAAAAAAAAAAAAAAAAAAAAAAAAAPA/AAAAAAAAAAAAAAAAAAAAAAAAAAAAAPA/AAAAAAAAAAAAAAAAAAAAAAAAAAAAAAAAAAAAAAAAVUAAAAAAAAAAAAAAAAAAAAAAAAAAAAAAAAAAAAAAAADwPwAAAAAAAAAAAAAAAAAAAAAAAAAAAAAAAAAAAAAAAAAAAAAAAAAA8D8AAAAAAAAAAAAAAAAAAAAAAAAAAAAAUkAAAAAAAAAAAAAAAAAAAAAAAAAAAAAAAAAAAAAAAAAAAAAAAAAAAAAAAAAAAAAA8D8AAAAAAAAAAAAAAAAAAPA/AAAAAAAAAAAAAAAAAAAAAAAAAAAAAAAAAAAAAAAATUAAAAAAAAAAAAAAAAAAAAAAAAAAAAAA8D8AAAAAAAAAAAAAAAAAAAAAAAAAAAAAAAAAAAAAAAAAAAAAAAAAAPA/AAAAAAAAAAAAAAAAAAAAAAAAAAAAAPA/AAAAAABAUkAAAAAAAAAAAAAAAAAAAAAAAAAAAAAAAAAAAAAAAAAAAAAAAAAAAAAAAAAAAAAA8D8AAAAAAAAAAAAAAAAAAAAAAAAAAAAA8D8AAAAAAAAAAAAAAAAAAAAAAAAAAABAUkAAAAAAAAAAAAAAAAAAAAAAAAAAAAAAAAAAAAAAAAAAAAAAAAAAAAAAAAAAAAAA8D8AAAAAAAAAAAAAAAAAAAAAAAAAAAAA8D8AAAAAAAAAAAAAAAAAAAAAAAAAAACAUEAAAAAAAAAAAAAAAAAAAAAAAAAAAAAAAAAAAAAAAADwPwAAAAAAAAAAAAAAAAAAAAAAAAAAAADwPwAAAAAAAPA/AAAAAAAAAAAAAAAAAAAAAAAAAAAAAAAAAAAAAACAUEAAAAAAAAAAAAAAAAAAAAAAAAAAAAAAAAAAAAAAAAAAAAAAAAAAAPA/AAAAAAAAAAAAAAAAAADwPwAAAAAAAAAAAAAAAAAA8D8AAAAAAAAAAAAAAAAAAAAAAAAAAAAAU0AAAAAAAAAAAAAAAAAAAAAAAAAAAAAAAAAAAAAAAAAAAAAAAAAAAPA/AAAAAAAAAAAAAAAAAAAAAAAAAAAAAPA/AAAAAAAAAAAAAAAAAAAAAAAAAAAAAAAAAAAAAABAVEAAAAAAAAAAAAAAAAAAAAAAAAAAAAAAAAAAAAAAAADwPwAAAAAAAAAAAAAAAAAAAAAAAAAAAAAAAAAAAAAAAPA/AAAAAAAAAAAAAAAAAAAAAAAAAAAAAPA/AAAAAADAU0AAAAAAAAAAAAAAAAAAAAAAAAAAAAAAAAAAAAAAAAAAAAAAAAAAAPA/AAAAAAAAAAAAAAAAAADwPwAAAAAAAAAAAAAAAAAA8D8AAAAAAAAAAAAAAAAAAAAAAAAAAADAUUAAAAAAAAAAAAAAAAAAAAAAAAAAAAAAAAAAAAAAAAAAAAAAAAAAAAAAAAAAAAAA8D8AAAAAAAAAAAAAAAAAAPA/AAAAAAAAAAAAAAAAAAAAAAAAAAAAAAAAAAAAAABAU0AAAAAAAAAAAAAAAAAAAAAAAAAAAAAAAAAAAAAAAAAAAAAAAAAAAPA/AAAAAAAAAAAAAAAAAAAAAAAAAAAAAPA/AAAAAAAAAAAAAAAAAAAAAAAAAAAAAAAAAAAAAACASkAAAAAAAAAAAAAAAAAAAAAAAAAAAAAAAAAAAAAAAAAAAAAAAAAAAPA/AAAAAAAAAAAAAAAAAAAAAAAAAAAAAPA/AAAAAAAAAAAAAAAAAAAAAAAAAAAAAAAAAAAAAACAUEAAAAAAAAAAAAAAAAAAAAAAAAAAAAAAAAAAAAAAAADwPwAAAAAAAAAAAAAAAAAAAAAAAAAAAAAAAAAAAAAAAPA/AAAAAAAAAAAAAAAAAAAAAAAAAAAAAPA/AAAAAABAUkAAAAAAAAAAAAAAAAAAAAAAAAAAAAAAAAAAAAAAAAAAAAAAAAAAAPA/AAAAAAAAAAAAAAAAAAAAAAAAAAAAAPA/AAAAAAAAAAAAAAAAAAAAAAAAAAAAAAAAAAAAAAAATUAAAAAAAAAAAAAAAAAAAAAAAAAAAAAA8D8AAAAAAAAAAAAAAAAAAAAAAAAAAAAAAAAAAAAAAADwPwAAAAAAAAAAAAAAAAAA8D8AAAAAAAAAAAAAAAAAAPA/AAAAAABAUUAAAAAAAAAAAAAAAAAAAAAAAAAAAAAA8D8AAAAAAAAAAAAAAAAAAAAAAAAAAAAAAAAAAAAAAAAAAAAAAAAAAPA/AAAAAAAAAAAAAAAAAAAAAAAAAAAAAPA/AAAAAAAAUkAAAAAAAAAAAAAAAAAAAAAAAAAAAAAAAAAAAAAAAADwPwAAAAAAAAAAAAAAAAAAAAAAAAAAAAAAAAAAAAAAAPA/AAAAAAAAAAAAAAAAAAAAAAAAAAAAAAAAAAAAAACASUAAAAAAAAAAAAAAAAAAAAAAAAAAAAAAAAAAAAAAAAAAAAAAAAAAAPA/AAAAAAAAAAAAAAAAAAAAAAAAAAAAAPA/AAAAAAAAAAAAAAAAAAAAAAAAAAAAAAAAAAAAAAAAT0AAAAAAAAAAAAAAAAAAAAAAAAAAAAAAAAAAAAAAAADwPwAAAAAAAAAAAAAAAAAAAAAAAAAAAAAAAAAAAAAAAPA/AAAAAAAAAAAAAAAAAAAAAAAAAAAAAAAAAAAAAACAUUAAAAAAAAAAAAAAAAAAAAAAAAAAAAAAAAAAAAAAAADwPwAAAAAAAAAAAAAAAAAAAAAAAAAAAADwPwAAAAAAAPA/AAAAAAAAAAAAAAAAAAAAAAAAAAAAAPA/AAAAAABAU0AAAAAAAAAAAAAAAAAAAAAAAAAAAAAAAAAAAAAAAAAAAAAAAAAAAPA/AAAAAAAAAAAAAAAAAADwPwAAAAAAAPA/AAAAAAAAAAAAAAAAAAAAAAAAAAAAAAAAAAAAAABAUEAAAAAAAAAAAAAAAAAAAAAAAAAAAAAA8D8AAAAAAAAAAAAAAAAAAAAAAAAAAAAAAAAAAAAAAAAAAAAAAAAAAPA/AAAAAAAAAAAAAAAAAAAAAAAAAAAAAPA/AAAAAACAUUAAAAAAAAAAAAAAAAAAAAAAAAAAAAAA8D8AAAAAAAAAAAAAAAAAAAAAAAAAAAAAAAAAAAAAAAAAAAAAAAAAAPA/AAAAAAAAAAAAAAAAAAAAAAAAAAAAAPA/AAAAAACATkAAAAAAAAAAAAAAAAAAAAAAAAAAAAAAAAAAAAAAAAAAAAAAAAAAAAAAAAAAAAAA8D8AAAAAAAAAAAAAAAAAAPA/AAAAAAAAAAAAAAAAAAAAAAAAAAAAAAAAAAAAAACATkAAAAAAAAAAAAAAAAAAAAAAAAAAAAAAAAAAAAAAAAAAAAAAAAAAAAAAAAAAAAAA8D8AAAAAAAAAAAAAAAAAAPA/AAAAAAAAAAAAAAAAAAAAAAAAAAAAAAAAAAAAAAAAT0AAAAAAAAAAAAAAAAAAAAAAAAAAAAAAAAAAAAAAAAAAAAAAAAAAAPA/AAAAAAAAAAAAAAAAAADwPwAAAAAAAPA/AAAAAAAAAAAAAAAAAAAAAAAAAAAAAAAAAAAAAABAUkAAAAAAAAAAAAAAAAAAAAAAAAAAAAAAAAAAAAAAAAAAAAAAAAAAAPA/AAAAAAAAAAAAAAAAAAAAAAAAAAAAAPA/AAAAAAAAAAAAAAAAAAAAAAAAAAAAAPA/AAAAAACATkAAAAAAAAAAAAAAAAAAAAAAAAAAAAAA8D8AAAAAAAAAAAAAAAAAAAAAAAAAAAAAAAAAAAAAAAAAAAAAAAAAAAAAAAAAAAAA8D8AAAAAAAAAAAAAAAAAAPA/AAAAAACASUAAAAAAAAAAAAAAAAAAAAAAAAAAAAAAAAAAAAAAAAAAAAAAAAAAAPA/AAAAAAAAAAAAAAAAAAAAAAAAAAAAAPA/AAAAAAAAAAAAAAAAAAAAAAAAAAAAAAAAAAAAAAAAUUAAAAAAAAAAAAAAAAAAAAAAAAAAAAAAAAAAAAAAAADwPwAAAAAAAAAAAAAAAAAAAAAAAAAAAADwPwAAAAAAAPA/AAAAAAAAAAAAAAAAAAAAAAAAAAAAAPA/AAAAAACAUkAAAAAAAAAAAAAAAAAAAAAAAAAAAAAA8D8AAAAAAAAAAAAAAAAAAAAAAAAAAAAAAAAAAAAAAADwPwAAAAAAAPA/AAAAAAAAAAAAAAAAAAAAAAAAAAAAAPA/AAAAAAAAVEAAAAAAAAAAAAAAAAAAAAAAAAAAAAAAAAAAAAAAAADwPwAAAAAAAAAAAAAAAAAAAAAAAAAAAAAAAAAAAAAAAPA/AAAAAAAAAAAAAAAAAAAAAAAAAAAAAAAAAAAAAAAAUEAAAAAAAAAAAAAAAAAAAAAAAAAAAAAAAAAAAAAAAADwPwAAAAAAAAAAAAAAAAAAAAAAAAAAAAAAAAAAAAAAAPA/AAAAAAAAAAAAAAAAAAAAAAAAAAAAAPA/AAAAAACAUUAAAAAAAAAAAAAAAAAAAAAAAAAAAAAAAAAAAAAAAAAAAAAAAAAAAAAAAAAAAAAA8D8AAAAAAAAAAAAAAAAAAPA/AAAAAAAAAAAAAAAAAAAAAAAAAAAAAAAAAAAAAADAU0AAAAAAAAAAAAAAAAAAAAAAAAAAAAAAAAAAAAAAAAAAAAAAAAAAAAAAAAAAAAAA8D8AAAAAAAAAAAAAAAAAAPA/AAAAAAAAAAAAAAAAAAAAAAAAAAAAAAAAAAAAAAAAUkAAAAAAAAAAAAAAAAAAAAAAAAAAAAAAAAAAAAAAAADwPwAAAAAAAAAAAAAAAAAAAAAAAAAAAAAAAAAAAAAAAPA/AAAAAAAAAAAAAAAAAAAAAAAAAAAAAPA/AAAAAACASUAAAAAAAAAAAAAAAAAAAAAAAAAAAAAAAAAAAAAAAAAAAAAAAAAAAPA/AAAAAAAAAAAAAAAAAADwPwAAAAAAAPA/AAAAAAAAAAAAAAAAAAAAAAAAAAAAAAAAAAAAAAAAUEAAAAAAAAAAAAAAAAAAAAAAAAAAAAAAAAAAAAAAAAAAAAAAAAAAAPA/AAAAAAAAAAAAAAAAAADwPwAAAAAAAPA/AAAAAAAAAAAAAAAAAAAAAAAAAAAAAAAAAAAAAACAUkAAAAAAAAAAAAAAAAAAAAAAAAAAAAAAAAAAAAAAAADwPwAAAAAAAAAAAAAAAAAAAAAAAAAAAADwPwAAAAAAAPA/AAAAAAAAAAAAAAAAAAAAAAAAAAAAAAAAAAAAAAAAVUAAAAAAAAAAAAAAAAAAAAAAAAAAAAAAAAAAAAAAAAAAAAAAAAAAAPA/AAAAAAAAAAAAAAAAAADwPwAAAAAAAPA/AAAAAAAAAAAAAAAAAAAAAAAAAAAAAAAAAAAAAAAASkAAAAAAAAAAAAAAAAAAAAAAAAAAAAAAAAAAAAAAAADwPwAAAAAAAAAAAAAAAAAAAAAAAAAAAADwPwAAAAAAAPA/AAAAAAAAAAAAAAAAAAAAAAAAAAAAAPA/AAAAAACARkAAAAAAAAAAAAAAAAAAAAAAAAAAAAAAAAAAAAAAAAAAAAAAAAAAAPA/AAAAAAAAAAAAAAAAAADwPwAAAAAAAPA/AAAAAAAAAAAAAAAAAAAAAAAAAAAAAAAAAAAAAAAAS0AAAAAAAAAAAAAAAAAAAAAAAAAAAAAAAAAAAAAAAAAAAAAAAAAAAPA/AAAAAAAAAAAAAAAAAADwPwAAAAAAAPA/AAAAAAAAAAAAAAAAAAAAAAAAAAAAAAAAAAAAAACATUAAAAAAAAAAAAAAAAAAAAAAAAAAAAAAAAAAAAAAAADwPwAAAAAAAAAAAAAAAAAAAAAAAAAAAADwPwAAAAAAAPA/AAAAAAAAAAAAAAAAAAAAAAAAAAAAAAAAAAAAAACAVEAAAAAAAAAAAAAAAAAAAAAAAAAAAAAAAAAAAAAAAAAAAAAAAAAAAPA/AAAAAAAAAAAAAAAAAAAAAAAAAAAAAPA/AAAAAAAAAAAAAAAAAAAAAAAAAAAAAAAAAAAAAABAUkAAAAAAAAAAAAAAAAAAAAAAAAAAAAAAAAAAAAAAAAAAAAAAAAAAAPA/AAAAAAAAAAAAAAAAAADwPwAAAAAAAPA/AAAAAAAAAAAAAAAAAAAAAAAAAAAAAAAAAAAAAAAAUkAAAAAAAAAAAAAAAAAAAAAAAAAAAAAA8D8AAAAAAAAAAAAAAAAAAAAAAAAAAAAAAAAAAAAAAADwPwAAAAAAAPA/AAAAAAAAAAAAAAAAAAAAAAAAAAAAAPA/AAAAAAAAUkAAAAAAAAAAAAAAAAAAAAAAAAAAAAAA8D8AAAAAAAAAAAAAAAAAAAAAAAAAAAAAAAAAAAAAAADwPwAAAAAAAPA/AAAAAAAAAAAAAAAAAAAAAAAAAAAAAAAAAAAAAACAU0AAAAAAAAAAAAAAAAAAAAAAAAAAAAAAAAAAAAAAAAAAAAAAAAAAAPA/AAAAAAAAAAAAAAAAAAAAAAAAAAAAAPA/AAAAAAAAAAAAAAAAAAAAAAAAAAAAAAAAAAAAAAAASkAAAAAAAAAAAAAAAAAAAAAAAAAAAAAAAAAAAAAAAAAAAAAAAAAAAPA/AAAAAAAAAAAAAAAAAAAAAAAAAAAAAAAAAAAAAAAAAAAAAAAAAADwPwAAAAAAAAAAAAAAAACATkAAAAAAAAAAAAAAAAAAAAAAAAAAAAAAAAAAAAAAAADwPwAAAAAAAAAAAAAAAAAAAAAAAAAAAAAAAAAAAAAAAPA/AAAAAAAAAAAAAAAAAAAAAAAAAAAAAPA/AAAAAABAU0AAAAAAAAAAAAAAAAAAAAAAAAAAAAAAAAAAAAAAAADwPwAAAAAAAAAAAAAAAAAAAAAAAAAAAADwPwAAAAAAAPA/AAAAAAAAAAAAAAAAAAAAAAAAAAAAAAAAAAAAAABAVEAAAAAAAAAAAAAAAAAAAAAAAAAAAAAAAAAAAAAAAADwPwAAAAAAAAAAAAAAAAAAAAAAAAAAAAAAAAAAAAAAAPA/AAAAAAAAAAAAAAAAAAAAAAAAAAAAAPA/AAAAAABAUUAAAAAAAAAAAAAAAAAAAAAAAAAAAAAAAAAAAAAAAADwPwAAAAAAAAAAAAAAAAAAAAAAAAAAAADwPwAAAAAAAAAAAAAAAAAA8D8AAAAAAAAAAAAAAAAAAAAAAAAAAACAUkAAAAAAAAAAAAAAAAAAAAAAAAAAAAAAAAAAAAAAAAAAAAAAAAAAAAAAAAAAAAAA8D8AAAAAAADwPwAAAAAAAAAAAAAAAAAAAAAAAAAAAADwPwAAAAAAAAAAAAAAAADAUEAAAAAAAAAAAAAAAAAAAAAAAAAAAAAA8D8AAAAAAAAAAAAAAAAAAAAAAAAAAAAAAAAAAAAAAADwPwAAAAAAAPA/AAAAAAAAAAAAAAAAAAAAAAAAAAAAAPA/AAAAAACAUUAAAAAAAAAAAAAAAAAAAAAAAAAAAAAAAAAAAAAAAAAAAAAAAAAAAPA/AAAAAAAAAAAAAAAAAAAAAAAAAAAAAPA/AAAAAAAAAAAAAAAAAAAAAAAAAAAAAAAAAAAAAADAUkAAAAAAAAAAAAAAAAAAAAAAAAAAAAAAAAAAAAAAAAAAAAAAAAAAAAAAAAAAAAAA8D8AAAAAAADwPwAAAAAAAAAAAAAAAAAA8D8AAAAAAAAAAAAAAAAAAAAAAAAAAACAU0AAAAAAAAAAAAAAAAAAAAAAAAAAAAAAAAAAAAAAAAAAAAAAAAAAAPA/AAAAAAAAAAAAAAAAAADwPwAAAAAAAPA/AAAAAAAAAAAAAAAAAAAAAAAAAAAAAAAAAAAAAACAUUAAAAAAAAAAAAAAAAAAAAAAAAAAAAAAAAAAAAAAAAAAAAAAAAAAAPA/AAAAAAAAAAAAAAAAAADwPwAAAAAAAAAAAAAAAAAA8D8AAAAAAAAAAAAAAAAAAAAAAAAAAACAU0AAAAAAAAAAAAAAAAAAAAAAAAAAAAAAAAAAAAAAAADwPwAAAAAAAAAAAAAAAAAAAAAAAAAAAAAAAAAAAAAAAPA/AAAAAAAAAAAAAAAAAAAAAAAAAAAAAPA/AAAAAADAUkAAAAAAAAAAAAAAAAAAAAAAAAAAAAAAAAAAAAAAAAAAAAAAAAAAAAAAAAAAAAAA8D8AAAAAAAAAAAAAAAAAAAAAAAAAAAAAAAAAAAAAAADwPwAAAAAAAAAAAAAAAACASUAAAAAAAAAAAAAAAAAAAAAAAAAAAAAAAAAAAAAAAAAAAAAAAAAAAAAAAAAAAAAA8D8AAAAAAAAAAAAAAAAAAPA/AAAAAAAAAAAAAAAAAAAAAAAAAAAAAAAAAAAAAACAUUAAAAAAAAAAAAAAAAAAAAAAAAAAAAAAAAAAAAAAAADwPwAAAAAAAAAAAAAAAAAAAAAAAAAAAAAAAAAAAAAAAAAAAAAAAAAA8D8AAAAAAAAAAAAAAAAAAAAAAAAAAAAATkAAAAAAAAAAAAAAAAAAAAAAAAAAAAAAAAAAAAAAAADwPwAAAAAAAAAAAAAAAAAAAAAAAAAAAAAAAAAAAAAAAPA/AAAAAAAAAAAAAAAAAAAAAAAAAAAAAAAAAAAAAACAU0AAAAAAAAAAAAAAAAAAAAAAAAAAAAAAAAAAAAAAAADwPwAAAAAAAAAAAAAAAAAAAAAAAAAAAAAAAAAAAAAAAAAAAAAAAAAA8D8AAAAAAAAAAAAAAAAAAPA/AAAAAABAUkAAAAAAAAAAAAAAAAAAAAAAAAAAAAAAAAAAAAAAAAAAAAAAAAAAAAAAAAAAAAAA8D8AAAAAAADwPwAAAAAAAAAAAAAAAAAA8D8AAAAAAAAAAAAAAAAAAAAAAAAAAAAAT0AAAAAAAAAAAAAAAAAAAAAAAAAAAAAAAAAAAAAAAAAAAAAAAAAAAPA/AAAAAAAAAAAAAAAAAAAAAAAAAAAAAPA/AAAAAAAAAAAAAAAAAAAAAAAAAAAAAAAAAAAAAACAU0AAAAAAAAAAAAAAAAAAAAAAAAAAAAAAAAAAAAAAAADwPwAAAAAAAAAAAAAAAAAAAAAAAAAAAAAAAAAAAAAAAPA/AAAAAAAAAAAAAAAAAAAAAAAAAAAAAPA/AAAAAACAUkAAAAAAAAAAAAAAAAAAAAAAAAAAAAAAAAAAAAAAAAAAAAAAAAAAAAAAAAAAAAAA8D8AAAAAAAAAAAAAAAAAAAAAAAAAAAAA8D8AAAAAAAAAAAAAAAAAAAAAAAAAAADAU0AAAAAAAAAAAAAAAAAAAAAAAAAAAAAA8D8AAAAAAAAAAAAAAAAAAAAAAAAAAAAAAAAAAAAAAADwPwAAAAAAAPA/AAAAAAAAAAAAAAAAAAAAAAAAAAAAAPA/AAAAAACAVEAAAAAAAAAAAAAAAAAAAAAAAAAAAAAAAAAAAAAAAAAAAAAAAAAAAPA/AAAAAAAAAAAAAAAAAAAAAAAAAAAAAAAAAAAAAAAA8D8AAAAAAAAAAAAAAAAAAAAAAAAAAACAT0AAAAAAAAAAAAAAAAAAAAAAAAAAAAAAAAAAAAAAAAAAAAAAAAAAAPA/AAAAAAAAAAAAAAAAAAAAAAAAAAAAAAAAAAAAAAAA8D8AAAAAAAAAAAAAAAAAAAAAAAAAAAAAUEAAAAAAAAAAAAAAAAAAAAAAAAAAAAAA8D8AAAAAAAAAAAAAAAAAAAAAAAAAAAAAAAAAAAAAAAAAAAAAAAAAAAAAAAAAAAAA8D8AAAAAAAAAAAAAAAAAAPA/AAAAAACAT0AAAAAAAAAAAAAAAAAAAAAAAAAAAAAA8D8AAAAAAAAAAAAAAAAAAAAAAAAAAAAAAAAAAAAAAAAAAAAAAAAAAPA/AAAAAAAAAAAAAAAAAAAAAAAAAAAAAPA/AAAAAADAU0AAAAAAAAAAAAAAAAAAAAAAAAAAAAAAAAAAAAAAAAAAAAAAAAAAAPA/AAAAAAAAAAAAAAAAAAAAAAAAAAAAAPA/AAAAAAAAAAAAAAAAAAAAAAAAAAAAAPA/AAAAAACAS0AAAAAAAAAAAAAAAAAAAAAAAAAAAAAAAAAAAAAAAAAAAAAAAAAAAPA/AAAAAAAAAAAAAAAAAAAAAAAAAAAAAPA/AAAAAAAAAAAAAAAAAAAAAAAAAAAAAAAAAAAAAADAVEAAAAAAAAAAAAAAAAAAAAAAAAAAAAAA8D8AAAAAAAAAAAAAAAAAAAAAAAAAAAAAAAAAAAAAAADwPwAAAAAAAAAAAAAAAAAA8D8AAAAAAAAAAAAAAAAAAPA/AAAAAACAUkAAAAAAAAAAAAAAAAAAAAAAAAAAAAAAAAAAAAAAAAAAAAAAAAAAAAAAAAAAAAAA8D8AAAAAAAAAAAAAAAAAAPA/AAAAAAAAAAAAAAAAAAAAAAAAAAAAAAAAAAAAAACARkAAAAAAAAAAAAAAAAAAAAAAAAAAAAAA8D8AAAAAAAAAAAAAAAAAAAAAAAAAAAAAAAAAAAAAAAAAAAAAAAAAAPA/AAAAAAAAAAAAAAAAAAAAAAAAAAAAAPA/AAAAAABAUUAAAAAAAAAAAAAAAAAAAAAAAAAAAAAAAAAAAAAAAAAAAAAAAAAAAPA/AAAAAAAAAAAAAAAAAAAAAAAAAAAAAAAAAAAAAAAA8D8AAAAAAAAAAAAAAAAAAAAAAAAAAABAUkAAAAAAAAAAAAAAAAAAAAAAAAAAAAAAAAAAAAAAAADwPwAAAAAAAAAAAAAAAAAAAAAAAAAAAAAAAAAAAAAAAPA/AAAAAAAAAAAAAAAAAAAAAAAAAAAAAAAAAAAAAAAASkAAAAAAAAAAAAAAAAAAAAAAAAAAAAAAAAAAAAAAAADwPwAAAAAAAAAAAAAAAAAAAAAAAAAAAADwPwAAAAAAAPA/AAAAAAAAAAAAAAAAAAAAAAAAAAAAAPA/AAAAAADAVEAAAAAAAAAAAAAAAAAAAAAAAAAAAAAAAAAAAAAAAADwPwAAAAAAAAAAAAAAAAAAAAAAAAAAAAAAAAAAAAAAAPA/AAAAAAAAAAAAAAAAAAAAAAAAAAAAAAAAAAAAAACAUkAAAAAAAAAAAAAAAAAAAAAAAAAAAAAAAAAAAAAAAADwPwAAAAAAAAAAAAAAAAAAAAAAAAAAAAAAAAAAAAAAAAAAAAAAAAAA8D8AAAAAAAAAAAAAAAAAAAAAAAAAAABAUEAAAAAAAAAAAAAAAAAAAAAAAAAAAAAA8D8AAAAAAAAAAAAAAAAAAAAAAAAAAAAAAAAAAAAAAAAAAAAAAAAAAPA/AAAAAAAAAAAAAAAAAAAAAAAAAAAAAPA/AAAAAACATUAAAAAAAAAAAAAAAAAAAAAAAAAAAAAAAAAAAAAAAADwPwAAAAAAAAAAAAAAAAAAAAAAAAAAAAAAAAAAAAAAAPA/AAAAAAAAAAAAAAAAAAAAAAAAAAAAAPA/AAAAAAAAU0AAAAAAAAAAAAAAAAAAAAAAAAAAAAAAAAAAAAAAAAAAAAAAAAAAAAAAAAAAAAAA8D8AAAAAAADwPwAAAAAAAAAAAAAAAAAA8D8AAAAAAAAAAAAAAAAAAAAAAAAAAAAATUAAAAAAAAAAAAAAAAAAAAAAAAAAAAAAAAAAAAAAAADwPwAAAAAAAAAAAAAAAAAAAAAAAAAAAAAAAAAAAAAAAAAAAAAAAAAA8D8AAAAAAAAAAAAAAAAAAAAAAAAAAABAUkAAAAAAAAAAAAAAAAAAAAAAAAAAAAAAAAAAAAAAAAAAAAAAAAAAAAAAAAAAAAAA8D8AAAAAAAAAAAAAAAAAAPA/AAAAAAAAAAAAAAAAAAAAAAAAAAAAAAAAAAAAAADAUkAAAAAAAAAAAAAAAAAAAAAAAAAAAAAAAAAAAAAAAADwPwAAAAAAAAAAAAAAAAAAAAAAAAAAAAAAAAAAAAAAAPA/AAAAAAAAAAAAAAAAAAAAAAAAAAAAAAAAAAAAAADAU0AAAAAAAAAAAAAAAAAAAAAAAAAAAAAAAAAAAAAAAADwPwAAAAAAAAAAAAAAAAAAAAAAAAAAAADwPwAAAAAAAPA/AAAAAAAAAAAAAAAAAAAAAAAAAAAAAAAAAAAAAAAAVEAAAAAAAAAAAAAAAAAAAAAAAAAAAAAAAAAAAAAAAADwPwAAAAAAAAAAAAAAAAAAAAAAAAAAAAAAAAAAAAAAAPA/AAAAAAAAAAAAAAAAAAAAAAAAAAAAAAAAAAAAAAAAUkAAAAAAAAAAAAAAAAAAAAAAAAAAAAAAAAAAAAAAAADwPwAAAAAAAAAAAAAAAAAAAAAAAAAAAAAAAAAAAAAAAPA/AAAAAAAAAAAAAAAAAAAAAAAAAAAAAAAAAAAAAADAUEAAAAAAAAAAAAAAAAAAAAAAAAAAAAAA8D8AAAAAAAAAAAAAAAAAAAAAAAAAAAAAAAAAAAAAAADwPwAAAAAAAPA/AAAAAAAAAAAAAAAAAAAAAAAAAAAAAPA/AAAAAACASUAAAAAAAAAAAAAAAAAAAAAAAAAAAAAAAAAAAAAAAADwPwAAAAAAAAAAAAAAAAAAAAAAAAAAAADwPwAAAAAAAAAAAAAAAAAA8D8AAAAAAAAAAAAAAAAAAAAAAAAAAACAU0AAAAAAAAAAAAAAAAAAAAAAAAAAAAAAAAAAAAAAAAAAAAAAAAAAAPA/AAAAAAAAAAAAAAAAAAAAAAAAAAAAAPA/AAAAAAAAAAAAAAAAAAAAAAAAAAAAAAAAAAAAAADAUEAAAAAAAAAAAAAAAAAAAAAAAAAAAAAAAAAAAAAAAADwPwAAAAAAAAAAAAAAAAAAAAAAAAAAAAAAAAAAAAAAAPA/AAAAAAAAAAAAAAAAAAAAAAAAAAAAAPA/AAAAAACAUEAAAAAAAAAAAAAAAAAAAAAAAAAAAAAAAAAAAAAAAAAAAAAAAAAAAPA/AAAAAAAAAAAAAAAAAAAAAAAAAAAAAAAAAAAAAAAA8D8AAAAAAAAAAAAAAAAAAAAAAAAAAACAU0AAAAAAAAAAAAAAAAAAAAAAAAAAAAAA8D8AAAAAAAAAAAAAAAAAAAAAAAAAAAAAAAAAAAAAAAAAAAAAAAAAAPA/AAAAAAAAAAAAAAAAAAAAAAAAAAAAAPA/AAAAAABAVUAAAAAAAAAAAAAAAAAAAAAAAAAAAAAAAAAAAAAAAAAAAAAAAAAAAPA/AAAAAAAAAAAAAAAAAADwPwAAAAAAAPA/AAAAAAAAAAAAAAAAAAAAAAAAAAAAAPA/AAAAAACAUkAAAAAAAAAAAAAAAAAAAAAAAAAAAAAAAAAAAAAAAADwPwAAAAAAAAAAAAAAAAAAAAAAAAAAAADwPwAAAAAAAPA/AAAAAAAAAAAAAAAAAAAAAAAAAAAAAPA/AAAAAADAU0AAAAAAAAAAAAAAAAAAAAAAAAAAAAAAAAAAAAAAAAAAAAAAAAAAAPA/AAAAAAAAAAAAAAAAAAAAAAAAAAAAAPA/AAAAAAAAAAAAAAAAAAAAAAAAAAAAAPA/AAAAAADAUkAAAAAAAAAAAAAAAAAAAAAAAAAAAAAAAAAAAAAAAAAAAAAAAAAAAPA/AAAAAAAAAAAAAAAAAAAAAAAAAAAAAAAAAAAAAAAAAAAAAAAAAADwPwAAAAAAAAAAAAAAAAAAUUAAAAAAAAAAAAAAAAAAAAAAAAAAAAAAAAAAAAAAAAAAAAAAAAAAAPA/AAAAAAAAAAAAAAAAAADwPwAAAAAAAPA/AAAAAAAAAAAAAAAAAAAAAAAAAAAAAAAAAAAAAADAUkAAAAAAAAAAAAAAAAAAAAAAAAAAAAAA8D8AAAAAAAAAAAAAAAAAAAAAAAAAAAAAAAAAAAAAAAAAAAAAAAAAAAAAAAAAAAAA8D8AAAAAAAAAAAAAAAAAAAAAAAAAAABAU0AAAAAAAAAAAAAAAAAAAAAAAAAAAAAAAAAAAAAAAAAAAAAAAAAAAPA/AAAAAAAAAAAAAAAAAADwPwAAAAAAAAAAAAAAAAAA8D8AAAAAAAAAAAAAAAAAAAAAAAAAAADAUEAAAAAAAAAAAAAAAAAAAAAAAAAAAAAAAAAAAAAAAADwPwAAAAAAAAAAAAAAAAAAAAAAAAAAAADwPwAAAAAAAPA/AAAAAAAAAAAAAAAAAAAAAAAAAAAAAPA/AAAAAAAAUkAAAAAAAAAAAAAAAAAAAAAAAAAAAAAAAAAAAAAAAAAAAAAAAAAAAPA/AAAAAAAAAAAAAAAAAAAAAAAAAAAAAPA/AAAAAAAAAAAAAAAAAAAAAAAAAAAAAPA/AAAAAADAU0AAAAAAAAAAAAAAAAAAAAAAAAAAAAAAAAAAAAAAAADwPwAAAAAAAAAAAAAAAAAAAAAAAAAAAADwPwAAAAAAAAAAAAAAAAAA8D8AAAAAAAAAAAAAAAAAAAAAAAAAAACAVEAAAAAAAAAAAAAAAAAAAAAAAAAAAAAAAAAAAAAAAAAAAAAAAAAAAAAAAAAAAAAA8D8AAAAAAADwPwAAAAAAAAAAAAAAAAAA8D8AAAAAAAAAAAAAAAAAAAAAAAAAAADAVEAAAAAAAAAAAAAAAAAAAAAAAAAAAAAAAAAAAAAAAAAAAAAAAAAAAPA/AAAAAAAAAAAAAAAAAADwPwAAAAAAAPA/AAAAAAAAAAAAAAAAAAAAAAAAAAAAAAAAAAAAAACAT0AAAAAAAAAAAAAAAAAAAAAAAAAAAAAA8D8AAAAAAAAAAAAAAAAAAAAAAAAAAAAAAAAAAAAAAADwPwAAAAAAAPA/AAAAAAAAAAAAAAAAAAAAAAAAAAAAAPA/AAAAAADAUEAAAAAAAAAAAAAAAAAAAAAAAAAAAAAAAAAAAAAAAAAAAAAAAAAAAPA/AAAAAAAAAAAAAAAAAADwPwAAAAAAAAAAAAAAAAAA8D8AAAAAAAAAAAAAAAAAAAAAAAAAAABAVUAAAAAAAAAAAAAAAAAAAAAAAAAAAAAAAAAAAAAAAAAAAAAAAAAAAPA/AAAAAAAAAAAAAAAAAAAAAAAAAAAAAPA/AAAAAAAAAAAAAAAAAAAAAAAAAAAAAPA/AAAAAAAAUkAAAAAAAAAAAAAAAAAAAAAAAAAAAAAAAAAAAAAAAAAAAAAAAAAAAPA/AAAAAAAAAAAAAAAAAAAAAAAAAAAAAPA/AAAAAAAAAAAAAAAAAAAAAAAAAAAAAPA/AAAAAACAU0AAAAAAAAAAAAAAAAAAAAAAAAAAAAAA8D8AAAAAAAAAAAAAAAAAAAAAAAAAAAAAAAAAAAAAAADwPwAAAAAAAPA/AAAAAAAAAAAAAAAAAAAAAAAAAAAAAPA/AAAAAADAVEAAAAAAAAAAAAAAAAAAAAAAAAAAAAAAAAAAAAAAAADwPwAAAAAAAAAAAAAAAAAAAAAAAAAAAAAAAAAAAAAAAPA/AAAAAAAAAAAAAAAAAAAAAAAAAAAAAPA/AAAAAACATUAAAAAAAAAAAAAAAAAAAAAAAAAAAAAAAAAAAAAAAAAAAAAAAAAAAAAAAAAAAAAA8D8AAAAAAAAAAAAAAAAAAPA/AAAAAAAAAAAAAAAAAAAAAAAAAAAAAPA/AAAAAACAUkAAAAAAAAAAAAAAAAAAAAAAAAAAAAAAAAAAAAAAAADwPwAAAAAAAAAAAAAAAAAAAAAAAAAAAAAAAAAAAAAAAPA/AAAAAAAAAAAAAAAAAAAAAAAAAAAAAPA/AAAAAAAAVEAAAAAAAAAAAAAAAAAAAAAAAAAAAAAAAAAAAAAAAAAAAAAAAAAAAAAAAAAAAAAA8D8AAAAAAAAAAAAAAAAAAPA/AAAAAAAAAAAAAAAAAAAAAAAAAAAAAAAAAAAAAACAT0AAAAAAAAAAAAAAAAAAAAAAAAAAAAAAAAAAAAAAAAAAAAAAAAAAAPA/AAAAAAAAAAAAAAAAAAAAAAAAAAAAAPA/AAAAAAAAAAAAAAAAAAAAAAAAAAAAAAAAAAAAAAAATEAAAAAAAAAAAAAAAAAAAAAAAAAAAAAAAAAAAAAAAAAAAAAAAAAAAPA/AAAAAAAAAAAAAAAAAAAAAAAAAAAAAAAAAAAAAAAA8D8AAAAAAAAAAAAAAAAAAAAAAAAAAABAVEAAAAAAAAAAAAAAAAAAAAAAAAAAAAAAAAAAAAAAAADwPwAAAAAAAAAAAAAAAAAAAAAAAAAAAADwPwAAAAAAAAAAAAAAAAAAAAAAAAAAAADwPwAAAAAAAPA/AAAAAAAATkAAAAAAAAAAAAAAAAAAAAAAAAAAAAAAAAAAAAAAAAAAAAAAAAAAAAAAAAAAAAAA8D8AAAAAAAAAAAAAAAAAAPA/AAAAAAAAAAAAAAAAAAAAAAAAAAAAAAAAAAAAAAAAUUAAAAAAAAAAAAAAAAAAAAAAAAAAAAAAAAAAAAAAAAAAAAAAAAAAAPA/AAAAAAAAAAAAAAAAAAAAAAAAAAAAAAAAAAAAAAAA8D8AAAAAAAAAAAAAAAAAAAAAAAAAAABAVUAAAAAAAAAAAAAAAAAAAAAAAAAAAAAAAAAAAAAAAAAAAAAAAAAAAAAAAAAAAAAA8D8AAAAAAAAAAAAAAAAAAAAAAAAAAAAAAAAAAAAAAADwPwAAAAAAAAAAAAAAAAAAUkAAAAAAAAAAAAAAAAAAAAAAAAAAAAAAAAAAAAAAAAAAAAAAAAAAAAAAAAAAAAAA8D8AAAAAAAAAAAAAAAAAAPA/AAAAAAAAAAAAAAAAAAAAAAAAAAAAAAAAAAAAAACAUkAAAAAAAAAAAAAAAAAAAAAAAAAAAAAAAAAAAAAAAAAAAAAAAAAAAPA/AAAAAAAAAAAAAAAAAAAAAAAAAAAAAPA/AAAAAAAAAAAAAAAAAAAAAAAAAAAAAAAAAAAAAABAU0AAAAAAAAAAAAAAAAAAAAAAAAAAAAAAAAAAAAAAAAAAAAAAAAAAAPA/AAAAAAAAAAAAAAAAAADwPwAAAAAAAPA/AAAAAAAAAAAAAAAAAAAAAAAAAAAAAPA/AAAAAADAU0AAAAAAAAAAAAAAAAAAAAAAAAAAAAAAAAAAAAAAAAAAAAAAAAAAAAAAAAAAAAAA8D8AAAAAAAAAAAAAAAAAAAAAAAAAAAAA8D8AAAAAAAAAAAAAAAAAAAAAAAAAAAAAUUAAAAAAAAAAAAAAAAAAAAAAAAAAAAAAAAAAAAAAAADwPwAAAAAAAAAAAAAAAAAAAAAAAAAAAAAAAAAAAAAAAPA/AAAAAAAAAAAAAAAAAAAAAAAAAAAAAPA/AAAAAAAAUkAAAAAAAAAAAAAAAAAAAAAAAAAAAAAAAAAAAAAAAAAAAAAAAAAAAAAAAAAAAAAA8D8AAAAAAAAAAAAAAAAAAPA/AAAAAAAAAAAAAAAAAAAAAAAAAAAAAAAAAAAAAADAUEAAAAAAAAAAAAAAAAAAAAAAAAAAAAAAAAAAAAAAAAAAAAAAAAAAAAAAAAAAAAAA8D8AAAAAAAAAAAAAAAAAAAAAAAAAAAAA8D8AAAAAAAAAAAAAAAAAAAAAAAAAAABAU0AAAAAAAAAAAAAAAAAAAAAAAAAAAAAAAAAAAAAAAAAAAAAAAAAAAPA/AAAAAAAAAAAAAAAAAAAAAAAAAAAAAPA/AAAAAAAAAAAAAAAAAAAAAAAAAAAAAPA/AAAAAACATEAAAAAAAAAAAAAAAAAAAAAAAAAAAAAA8D8AAAAAAAAAAAAAAAAAAAAAAAAAAAAAAAAAAAAAAADwPwAAAAAAAAAAAAAAAAAA8D8AAAAAAAAAAAAAAAAAAAAAAAAAAAAAVEAAAAAAAAAAAAAAAAAAAAAAAAAAAAAAAAAAAAAAAAAAAAAAAAAAAAAAAAAAAAAA8D8AAAAAAAAAAAAAAAAAAAAAAAAAAAAAAAAAAAAAAADwPwAAAAAAAAAAAAAAAACAUkAAAAAAAAAAAAAAAAAAAAAAAAAAAAAAAAAAAAAAAAAAAAAAAAAAAAAAAAAAAAAA8D8AAAAAAAAAAAAAAAAAAPA/AAAAAAAAAAAAAAAAAAAAAAAAAAAAAPA/AAAAAACAT0AAAAAAAAAAAAAAAAAAAAAAAAAAAAAAAAAAAAAAAAAAAAAAAAAAAAAAAAAAAAAA8D8AAAAAAAAAAAAAAAAAAPA/AAAAAAAAAAAAAAAAAAAAAAAAAAAAAAAAAAAAAABAUUAAAAAAAAAAAAAAAAAAAAAAAAAAAAAAAAAAAAAAAAAAAAAAAAAAAPA/AAAAAAAAAAAAAAAAAAAAAAAAAAAAAPA/AAAAAAAAAAAAAAAAAAAAAAAAAAAAAPA/AAAAAAAAU0AAAAAAAAAAAAAAAAAAAAAAAAAAAAAAAAAAAAAAAAAAAAAAAAAAAPA/AAAAAAAAAAAAAAAAAADwPwAAAAAAAPA/AAAAAAAAAAAAAAAAAAAAAAAAAAAAAAAAAAAAAABAVEAAAAAAAAAAAAAAAAAAAAAAAAAAAAAAAAAAAAAAAAAAAAAAAAAAAPA/AAAAAAAAAAAAAAAAAAAAAAAAAAAAAPA/AAAAAAAAAAAAAAAAAAAAAAAAAAAAAPA/AAAAAADAUEAAAAAAAAAAAAAAAAAAAAAAAAAAAAAA8D8AAAAAAAAAAAAAAAAAAAAAAAAAAAAAAAAAAAAAAAAAAAAAAAAAAPA/AAAAAAAAAAAAAAAAAAAAAAAAAAAAAAAAAAAAAAAAUUAAAAAAAAAAAAAAAAAAAAAAAAAAAAAAAAAAAAAAAADwPwAAAAAAAAAAAAAAAAAAAAAAAAAAAADwPwAAAAAAAPA/AAAAAAAAAAAAAAAAAAAAAAAAAAAAAAAAAAAAAAAAUEAAAAAAAAAAAAAAAAAAAAAAAAAAAAAAAAAAAAAAAAAAAAAAAAAAAPA/AAAAAAAAAAAAAAAAAADwPwAAAAAAAAAAAAAAAAAA8D8AAAAAAAAAAAAAAAAAAAAAAAAAAACAUUAAAAAAAAAAAAAAAAAAAAAAAAAAAAAAAAAAAAAAAAAAAAAAAAAAAPA/AAAAAAAAAAAAAAAAAAAAAAAAAAAAAPA/AAAAAAAAAAAAAAAAAAAAAAAAAAAAAAAAAAAAAAAAUkAAAAAAAAAAAAAAAAAAAAAAAAAAAAAAAAAAAAAAAAAAAAAAAAAAAAAAAAAAAAAA8D8AAAAAAAAAAAAAAAAAAPA/AAAAAAAAAAAAAAAAAAAAAAAAAAAAAAAAAAAAAAAAU0AAAAAAAAAAAAAAAAAAAAAAAAAAAAAAAAAAAAAAAADwPwAAAAAAAAAAAAAAAAAAAAAAAAAAAAAAAAAAAAAAAPA/AAAAAAAAAAAAAAAAAAAAAAAAAAAAAPA/AAAAAACATEAAAAAAAAAAAAAAAAAAAAAAAAAAAAAA8D8AAAAAAAAAAAAAAAAAAAAAAAAAAAAAAAAAAAAAAAAAAAAAAAAAAPA/AAAAAAAAAAAAAAAAAAAAAAAAAAAAAAAAAAAAAAAAT0AAAAAAAAAAAAAAAAAAAAAAAAAAAAAAAAAAAAAAAADwPwAAAAAAAAAAAAAAAAAAAAAAAAAAAADwPwAAAAAAAPA/AAAAAAAAAAAAAAAAAAAAAAAAAAAAAPA/AAAAAACAUUAAAAAAAAAAAAAAAAAAAAAAAAAAAAAA8D8AAAAAAAAAAAAAAAAAAAAAAAAAAAAAAAAAAAAAAAAAAAAAAAAAAPA/AAAAAAAAAAAAAAAAAAAAAAAAAAAAAPA/AAAAAAAAVEAAAAAAAAAAAAAAAAAAAAAAAAAAAAAAAAAAAAAAAAAAAAAAAAAAAPA/AAAAAAAAAAAAAAAAAADwPwAAAAAAAAAAAAAAAAAAAAAAAAAAAADwPwAAAAAAAAAAAAAAAAAAUkAAAAAAAAAAAAAAAAAAAAAAAAAAAAAAAAAAAAAAAADwPwAAAAAAAAAAAAAAAAAAAAAAAAAAAADwPwAAAAAAAPA/AAAAAAAAAAAAAAAAAAAAAAAAAAAAAAAAAAAAAACAT0AAAAAAAAAAAAAAAAAAAAAAAAAAAAAA8D8AAAAAAAAAAAAAAAAAAAAAAAAAAAAAAAAAAAAAAAAAAAAAAAAAAAAAAAAAAAAA8D8AAAAAAAAAAAAAAAAAAPA/AAAAAADAU0AAAAAAAAAAAAAAAAAAAAAAAAAAAAAAAAAAAAAAAADwPwAAAAAAAAAAAAAAAAAAAAAAAAAAAADwPwAAAAAAAPA/AAAAAAAAAAAAAAAAAAAAAAAAAAAAAAAAAAAAAADAVEAAAAAAAAAAAAAAAAAAAAAAAAAAAAAAAAAAAAAAAAAAAAAAAAAAAAAAAAAAAAAA8D8AAAAAAADwPwAAAAAAAAAAAAAAAAAA8D8AAAAAAAAAAAAAAAAAAAAAAAAAAAAAU0AAAAAAAAAAAAAAAAAAAAAAAAAAAAAA8D8AAAAAAAAAAAAAAAAAAAAAAAAAAAAAAAAAAAAAAAAAAAAAAAAAAPA/AAAAAAAAAAAAAAAAAAAAAAAAAAAAAAAAAAAAAABAUUAAAAAAAAAAAAAAAAAAAAAAAAAAAAAA8D8AAAAAAAAAAAAAAAAAAAAAAAAAAAAAAAAAAAAAAADwPwAAAAAAAPA/AAAAAAAAAAAAAAAAAAAAAAAAAAAAAPA/AAAAAACAUkAAAAAAAAAAAAAAAAAAAAAAAAAAAAAAAAAAAAAAAAAAAAAAAAAAAAAAAAAAAAAA8D8AAAAAAADwPwAAAAAAAAAAAAAAAAAAAAAAAAAAAADwPwAAAAAAAAAAAAAAAAAAVEAAAAAAAAAAAAAAAAAAAAAAAAAAAAAAAAAAAAAAAADwPwAAAAAAAAAAAAAAAAAAAAAAAAAAAADwPwAAAAAAAAAAAAAAAAAA8D8AAAAAAAAAAAAAAAAAAAAAAAAAAADAVEAAAAAAAAAAAAAAAAAAAAAAAAAAAAAAAAAAAAAAAADwPwAAAAAAAAAAAAAAAAAAAAAAAAAAAADwPwAAAAAAAPA/AAAAAAAAAAAAAAAAAAAAAAAAAAAAAPA/AAAAAABAUkAAAAAAAAAAAAAAAAAAAAAAAAAAAAAA8D8AAAAAAAAAAAAAAAAAAAAAAAAAAAAAAAAAAAAAAAAAAAAAAAAAAPA/AAAAAAAAAAAAAAAAAAAAAAAAAAAAAPA/AAAAAADAUkAAAAAAAAAAAAAAAAAAAAAAAAAAAAAA8D8AAAAAAAAAAAAAAAAAAAAAAAAAAAAAAAAAAAAAAAAAAAAAAAAAAPA/AAAAAAAAAAAAAAAAAAAAAAAAAAAAAPA/AAAAAABAVEAAAAAAAAAAAA87AAAADwEAAAYAAAAAAAAAAAAAAAAAAKA8AAAAAAAAoDwAAAAAAACgPAAAAAAAAKA8AAAAAAAA8D8AAAAAAACgPAAAAAAAAKA8AAAAAAAAAEAAAAAAAACgPAAAAAAAAAhAAAAAAAAAoDwAAAAAAACgPAAAAAAAAKA8AAAAAAAAoDwAAAAAAACgPAAAAAAAAKA8AAAAAAAAoDwAAAAAAACgPAAAAAAAAKA8AAAAAAAAoDwAAAAAAACgPAAAAAAAAPA/AAAAAAAAoDwAAAAAAACgPAAAAAAAAKA8AAAAAAAAoDwAAAAAAACgPAAAAAAAAABAAAAAAAAAoDwAAAAAAACgPAAAAAAAAKA8AAAAAAAAoDwAAAAAAACgPAAAAAAAAKA8AAAAAAAAAEAAAAAAAACgPAAAAAAAAKA8AAAAAAAAoDwAAAAAAADwPwAAAAAAAKA8AAAAAAAAoDwAAAAAAADwPwAAAAAAAKA8AAAAAAAAoDwAAAAAAADwPwAAAAAAAKA8AAAAAAAA8D8AAAAAAACgPAAAAAAAAABAAAAAAAAAoDwAAAAAAACgPAAAAAAAAKA8AAAAAAAAoDwAAAAAAACgPAAAAAAAAPA/AAAAAAAAoDwAAAAAAADwPwAAAAAAAKA8AAAAAAAAoDwAAAAAAACgPAAAAAAAAKA8AAAAAAAAoDwAAAAAAACgPAAAAAAAAKA8AAAAAAAAoDwAAAAAAACgPAAAAAAAAKA8AAAAAAAAoDwAAAAAAADwPwAAAAAAAKA8AAAAAAAAoDwAAAAAAACgPAAAAAAAAKA8AAAAAAAA8D8AAAAAAADwPwAAAAAAAKA8AAAAAAAAoDwAAAAAAACgPAAAAAAAAKA8AAAAAAAAoDwAAAAAAADwPwAAAAAAAKA8AAAAAAAA8D8AAAAAAACgPAAAAAAAAKA8AAAAAAAAoDwAAAAAAACgPAAAAAAAAKA8AAAAAAAAoDwAAAAAAACgPAAAAAAAAKA8AAAAAAAAoDwAAAAAAAAAQAAAAAAAAKA8AAAAAAAAoDwAAAAAAACgPAAAAAAAAKA8AAAAAAAA8D8AAAAAAACgPAAAAAAAAPA/AAAAAAAAAEAAAAAAAACgPAAAAAAAAKA8AAAAAAAA8D8AAAAAAACgPAAAAAAAAKA8AAAAAAAAoDwAAAAAAACgPAAAAAAAAKA8AAAAAAAAoDwAAAAAAACgPAAAAAAAAKA8AAAAAAAAoDwAAAAAAADwPwAAAAAAAABAAAAAAAAAoDwAAAAAAADwPwAAAAAAAKA8AAAAAAAAoDwAAAAAAACgPAAAAAAAAKA8AAAAAAAAoDwAAAAAAACgPAAAAAAAAPA/AAAAAAAAoDwAAAAAAACgPAAAAAAAAKA8AAAAAAAAoDwAAAAAAACgPAAAAAAAAKA8AAAAAAAAoDwAAAAAAACgPAAAAAAAABBAAAAAAAAAoDwAAAAAAACgPAAAAAAAAKA8AAAAAAAAoDwAAAAAAACgPAAAAAAAAPA/AAAAAAAA8D8AAAAAAADwPwAAAAAAAKA8AAAAAAAAoDwAAAAAAADwPwAAAAAAAKA8AAAAAAAA8D8AAAAAAACgPAAAAAAAAKA8AAAAAAAAoDwAAAAAAACgPAAAAAAAAPA/AAAAAAAA8D8AAAAAAACgPAAAAAAAAKA8AAAAAAAAoDwAAAAAAACgPAAAAAAAAKA8AAAAAAAA8D8AAAAAAACgPAAAAAAAAPA/AAAAAAAAoDwAAAAAAACgPAAAAAAAAKA8AAAAAAAA8D8AAAAAAADwPwAAAAAAAPA/AAAAAAAAoDwAAAAAAACgPAAAAAAAAKA8AAAAAAAA8D8AAAAAAACgPAAAAAAAAKA8AAAAAAAAoDwAAAAAAACgPAAAAAAAAPA/AAAAAAAAoDwAAAAAAACgPAAAAAAAAPA/AAAAAAAAoDwAAAAAAACgPAAAAAAAAKA8AAAAAAAAoDwAAAAAAACgPAAAAAAAAPA/AAAAAAAAoDwAAAAAAACgPAAAAAAAAABAAAAAAAAAoDwAAAAAAACgPAAAAAAAAKA8AAAAAAAAoDwAAAAAAACgPAAAAAAAAKA8AAAAAAAAAEAAAAAAAACgPAAAAAAAAKA8AAAAAAAAoDwAAAAAAACgPAAAAAAAAKA8AAAAAAAAoDwAAAAAAACgPAAAAAAAAKA8AAAAAAAAoDwAAAAAAACgPAAAAAAAAKA8AAAAAAAAAED////////vP////////+8/////////7z/////////vP////////+8/////////7z/////////vP////////+8/AAAAAAAAAEAAAAAAAAAAQP///////+8/////////7z/////////vP////////+8/////////7z8AAAAAAAAIQP///////+8/////////7z/////////vP////////+8/////////7z/////////vPwAAAAAAAABA////////7z/////////vP////////+8/////////7z/////////vP////////+8/////////7z/////////vP////////+8/AAAAAAAAAEAAAAAAAAAAQP///////+8/////////7z/////////vP////////+8/////////7z/////////vP////////+8/AAAAAAAAAED////////vP////////+8/////////7z8AAAAAAAAAQP///////+8/////////7z/////////vP////////+8/////////7z/////////vP////////+8/////////7z/////////vP////////+8/////////7z/////////vP////////+8/////////7z/////////vP////////+8/////////7z8AAAAAAAAAQAQ8AAAAgwFTeXN0ZW0uQ29sbGVjdGlvbnMuR2VuZXJpYy5MaXN0YDFbW1N0YXRzRGlyZWN0LkRhdGEuSVZhcmlhYmxlLCBTdGF0c0RpcmVjdCwgVmVyc2lvbj0zLjMuNS4wLCBDdWx0dXJlPW5ldXRyYWwsIFB1YmxpY0tleVRva2VuPW51bGxdXQMAAAAGX2l0ZW1zBV9zaXplCF92ZXJzaW9uBAAAHFN0YXRzRGlyZWN0LkRhdGEuSVZhcmlhYmxlW10CAAAACAgJTAAAAAMAAAADAAAAAT4AAAA8AAAACU0AAAAKAAAACgAAAAFAAAAAPAAAAAlOAAAAAQAAAAEAAAAHTAAAAAABAAAABAAAAAQaU3RhdHNEaXJlY3QuRGF0YS5JVmFyaWFibGUCAAAACU8AAAAJUAAAAAlRAAAACgdNAAAAAAEAAAAQAAAABBpTdGF0c0RpcmVjdC5EYXRhLklWYXJpYWJsZQIAAAAJUgAAAAlTAAAACVQAAAAJVQAAAAlWAAAACVcAAAAJWAAAAAlZAAAACVoAAAAJWwAAAA0GB04AAAAAAQAAAAQAAAAEGlN0YXRzRGlyZWN0LkRhdGEuSVZhcmlhYmxlAgAAAAlcAAAADQMFTwAAAB9TdGF0c0RpcmVjdC5EYXRhLlN0cmluZ1ZhcmlhYmxlAwAAABZHZW5lcmljVmFyaWFibGVgMStkYXRhKEdlbmVyaWNWYXJpYWJsZWAxKzxUaXRsZT5rX19CYWNraW5nRmllbGQpR2VuZXJpY1ZhcmlhYmxlYDErPE9yaWdpbj5rX19CYWNraW5nRmllbGQGAQQYU3RhdHNEaXJlY3QuRGF0YS5JT3JpZ2luAgAAAAIAAAAJXQAAAAZeAAAABE5hbWUKAVAAAABPAAAACV8AAAAGYAAAAAVWYWx1ZQoFUQAAAB9TdGF0c0RpcmVjdC5EYXRhLkRvdWJsZVZhcmlhYmxlBwAAAANzdW0DbWluA21heAxoYXNTdW1tYXJpZXMWR2VuZXJpY1ZhcmlhYmxlYDErZGF0YShHZW5lcmljVmFyaWFibGVgMSs8VGl0bGU+a19fQmFja2luZ0ZpZWxkKUdlbmVyaWNWYXJpYWJsZWAxKzxPcmlnaW4+a19fQmFja2luZ0ZpZWxkAAAAAAcBBAYGBgEGGFN0YXRzRGlyZWN0LkRhdGEuSU9yaWdpbgIAAAACAAAAAAAAAAAAAAAAAAAAAAAAAAAAAAAAAAAAAAlhAAAABmIAAAAJT2xkIHZhbHVlCgFSAAAAUQAAAAAAAAAAAAAAAAAAAAAAAAAAAAAAAAAAAAAJYwAAAAlCAAAACgFTAAAAUQAAAAAAAAAAAAAAAAAAAAAAAAAAAAAAAAAAAAAJZQAAAAlDAAAACgFUAAAAUQAAAAAAAAAAAAAAAAAAAAAAAAAAAAAAAAAAAAAJZwAAAAlEAAAACgFVAAAAUQAAAAAAAAAAAAAAAAAAAAAAAAAAAAAAAAAAAAAJaQAAAAlFAAAACgFWAAAAUQAAAAAAAAAAAAAAAAAAAAAAAAAAAAAAAAAAAAAKCUYAAAAJbAAAAAFXAAAAUQAAAAAAAAAAAAAAAAAAAAAAAAAAAAAAAAAAAAAJbQAAAAlHAAAACgFYAAAAUQAAAAAAAAAAAAAAAAAAAAAAAAAAAAAAAAAAAAAJbwAAAAlIAAAACgFZAAAAUQAAAAAAAAAAAAAAAAAAAAAAAAAAAAAAAAAAAAAJcQAAAAlJAAAACgFaAAAAUQAAAAAAAAAAAAAAAAAAAAAAAAAAAAAAAAAAAAAKCUoAAAAJdAAAAAFbAAAAUQAAAAAAAAAAAAAAAAAAAAAAAAAAAAAAAAAAAAAKCUsAAAAJdgAAAAFcAAAAUQAAAAAAAAAAAAAAAAAAAAAAAAAAAAAAAAAAAAAKCUEAAAAJeAAAABFdAAAACgAAAAlCAAAACUMAAAAJRAAAAAlFAAAACUYAAAAJRwAAAAlIAAAACUkAAAAJSgAAAAlLAAAAEV8AAAAKAAAABoMAAAADMCwyBoQAAAADMCwyBoUAAAADMCwyBoYAAAADMCwyBocAAAADMCw1BogAAAAEMCwyNQaJAAAABDAsMjUGigAAAAQwLDI1BosAAAADMCw1BowAAAAQNjksNDAzNzAzNzAzNzAzNw9hAAAACgAAAAaamZmZmZnJP5qZmZmZmck/mpmZmZmZyT+amZmZmZnJPwAAAAAAAOA/AAAAAAAA0D8AAAAAAADQPwAAAAAAANA/AAAAAAAA4D+dKw9I1llRQA9jAAAAsAEAAAYAAAAAAADwPwAAAAAAAPA/AAAAAAAAAAAAAAAAAAAAAAAAAAAAAAAAAAAAAAAAAAAAAAAAAAAAAAAAAAAAAAAAAAAAAAAAAAAAAAAAAAAAAAAAAAAAAAAAAAAAAAAA8D8AAAAAAAAAAAAAAAAAAAAAAAAAAAAA8D8AAAAAAAAAAAAAAAAAAAAAAAAAAAAAAAAAAAAAAAAAAAAAAAAAAAAAAAAAAAAA8D8AAAAAAADwPwAAAAAAAAAAAAAAAAAAAAAAAAAAAAAAAAAAAAAAAAAAAAAAAAAA8D8AAAAAAAAAAAAAAAAAAAAAAAAAAAAAAAAAAAAAAAAAAAAAAAAAAAAAAAAAAAAAAAAAAAAAAAAAAAAAAAAAAAAAAAAAAAAA8D8AAAAAAAAAAAAAAAAAAPA/AAAAAAAAAAAAAAAAAADwPwAAAAAAAAAAAAAAAAAAAAAAAAAAAAAAAAAAAAAAAAAAAAAAAAAAAAAAAAAAAAAAAAAAAAAAAAAAAAAAAAAAAAAAAAAAAAAAAAAAAAAAAAAAAAAAAAAA8D8AAAAAAAAAAAAAAAAAAPA/AAAAAAAAAAAAAAAAAAAAAAAAAAAAAAAAAAAAAAAA8D8AAAAAAAAAAAAAAAAAAPA/AAAAAAAAAAAAAAAAAAAAAAAAAAAAAAAAAAAAAAAAAAAAAAAAAADwPwAAAAAAAAAAAAAAAAAAAAAAAAAAAAAAAAAAAAAAAAAAAAAAAAAAAAAAAAAAAAAAAAAAAAAAAPA/AAAAAAAAAAAAAAAAAADwPwAAAAAAAAAAAAAAAAAAAAAAAAAAAAAAAAAAAAAAAAAAAAAAAAAAAAAAAAAAAADwPwAAAAAAAAAAAAAAAAAAAAAAAAAAAADwPwAAAAAAAAAAAAAAAAAAAAAAAAAAAAAAAAAAAAAAAAAAAAAAAAAAAAAAAAAAAAAAAAAAAAAAAAAAAAAAAAAA8D8AAAAAAAAAAAAAAAAAAAAAAAAAAAAAAAAAAAAAAADwPwAAAAAAAAAAAAAAAAAAAAAAAAAAAAAAAAAAAAAAAAAAAAAAAAAAAAAAAAAAAAAAAAAAAAAAAAAAAAAAAAAAAAAAAAAAAAAAAAAAAAAAAAAAAAAAAAAAAAAAAAAAAAAAAAAAAAAAAAAAAAAAAAAAAAAAAAAAAADwPwAAAAAAAAAAAAAAAAAAAAAAAAAAAAAAAAAAAAAAAAAAAAAAAAAAAAAAAAAAAAAAAAAAAAAAAAAAAAAAAAAAAAAAAAAAAAAAAAAAAAAAAAAAAAAAAAAAAAAAAAAAAAAAAAAAAAAAAAAAAAAAAAAAAAAAAAAAAADwPwAAAAAAAAAAAAAAAAAAAAAAAAAAAAAAAAAAAAAAAAAAAAAAAAAAAAAAAAAAAAAAAAAAAAAAAAAAAAAAAAAA8D8AAAAAAAAAAAAAAAAAAAAAAAAAAAAAAAAAAAAAAAAAAAAAAAAAAAAAAAAAAAAAAAAAAAAAAAAAAAAAAAAAAAAAAAAAAAAAAAAAAAAAAAAAAAAAAAAAAAAAAAAAAAAAAAAAAAAAAAAAAAAAAAAAAPA/AAAAAAAAAAAAAAAAAADwPwAAAAAAAAAAAAAAAAAA8D8AAAAAAADwPwAAAAAAAAAAAAAAAAAA8D8AAAAAAADwPwAAAAAAAAAAAAAAAAAAAAAAAAAAAAAAAAAAAAAAAAAAAAAAAAAAAAAAAAAAAAAAAAAAAAAAAPA/AAAAAAAAAAAAAAAAAAAAAAAAAAAAAAAAAAAAAAAA8D8AAAAAAADwPwAAAAAAAAAAAAAAAAAAAAAAAAAAAAAAAAAAAAAAAAAAAAAAAAAAAAAAAAAAAADwPwAAAAAAAAAAAAAAAAAAAAAAAAAAAAAAAAAAAAAAAAAAAAAAAAAA8D8AAAAAAAAAAAAAAAAAAAAAAAAAAAAAAAAAAAAAAAAAAAAAAAAAAAAAAAAAAAAA8D8AAAAAAAAAAAAAAAAAAAAAAAAAAAAAAAAAAAAAAAAAAAAAAAAAAAAAAAAAAAAAAAAAAAAAAAAAAAAAAAAAAAAAAAAAAAAAAAAAAAAAAAAAAAAAAAAAAAAAAAAAAAAAAAAAAAAAAAAAAAAAAAAAAAAAAAAAAAAAAAAAAAAAAAAAAAAAAAAAAAAAAAAAAAAAAAAAAAAAAAAAAAAAAAAAAAAAAAAAAAAAAAAAAAAAAAAAAAAAAAAAAAAAAAAAAAAAAAAAAAAAAAAAAAAAAAAAAPA/AAAAAAAAAAAAAAAAAADwPwAAAAAAAAAAAAAAAAAAAAAAAAAAAAAAAAAAAAAAAAAAAAAAAAAAAAAAAAAAAAAAAAAAAAAAAAAAAAAAAAAAAAAAAAAAAADwPwAAAAAAAAAAAAAAAAAAAAAAAAAAAADwPwAAAAAAAAAAAAAAAAAAAAAAAAAAAAAAAAAAAAAAAAAAAAAAAAAAAAAAAAAAAADwPwAAAAAAAAAAAAAAAAAAAAAAAAAAAAAAAAAAAAAAAAAAAAAAAAAAAAAAAAAAAAAAAAAAAAAAAAAAAAAAAAAAAAAAAAAAAAAAAAAAAAAAAAAAAAAAAAAAAAAAAAAAAAAAAAAAAAAAAAAAAAAAAAAA8D8AAAAAAAAAAAAAAAAAAAAAAAAAAAAAAAAAAAAAAADwPwAAAAAAAAAAAAAAAAAA8D8AAAAAAAAAAAAAAAAAAAAAAAAAAAAA8D8AAAAAAAAAAAAAAAAAAAAAAAAAAAAAAAAAAAAAAAAAAAAAAAAAAAAAAAAAAAAAAAAAAAAAAADwPwAAAAAAAAAAAAAAAAAA8D8AAAAAAAAAAAAAAAAAAAAAAAAAAAAAAAAAAAAAAAAAAAAAAAAAAAAAAAAAAAAAAAAAAAAAAAAAAAAAAAAAAAAAAAAAAAAA8D8AAAAAAAAAAAAAAAAAAAAAAAAAAAAAAAAAAAAAAAAAAAAAAAAAAAAAAAAAAAAAAAAAAAAAAAAAAAAAAAAAAPA/AAAAAAAA8D8AAAAAAAAAAAAAAAAAAAAAAAAAAAAAAAAAAAAAAAAAAAAAAAAAAAAAAAAAAAAAAAAAAAAAAAAAAAAAAAAAAAAAAAAAAAAA8D8AAAAAAAAAAAAAAAAAAAAAAAAAAAAAAAAAAAAAAAAAAAAAAAAAAAAAAAAAAAAAAAAAAAAAAAAAAAAAAAAAAAAAAAAAAAAA8D8AAAAAAAAAAAAAAAAAAPA/AAAAAAAAAAAAAAAAAAAAAAAAAAAAAAAAAAAAAAAAAAAAAAAAAAAAAAAAAAAAAAAAAAAAAAAAAAAAAAAAAAAAAAAAAAAAAAAAAAAAAAAAAAAAAAAAAAAAAAAAAAAAAAAAAAAAAAAA8D8AAAAAAAAAAAAAAAAAAAAAAAAAAAAAAAAAAAAAAAAAAAAAAAAAAPA/AAAAAAAAAAAAAAAAAAAAAAAAAAAAAAAAAAAAAAAAAAAAAAAAAAAAAAAAAAAAAAAAAAAAAAAA8D8AAAAAAAAAAAAAAAAAAAAAAAAAAAAAAAAAAAAAAAAAAAAAAAAAAPA/AAAAAAAAAAAAAAAAAAAAAAAAAAAAAAAAAAAAAAAAAAAAAAAAAAAAAAAAAAAAAAAAAAAAAAAAAAAAAAAAAAAAAAAAAAAAAAAAAAAAAAAAAAAAAAAAAAAAAAAAAAAAAAAAAAAAAAAAAAAAAAAAAAAAAAAAAAAAAAAAAAAAAAAAAAAAAAAAAAAAAAAAAAAAAAAAAAAAAAAAAAAAAAAAAAAAAAAAAAAAAAAAAAAAAAAAAAAAAAAAAAAAAAAAAAAAAAAAAAAAAAAAAAAAAAAAAAAAAAAAAAAAAAAAAAAAAAAAAAAAAAAAAAAAAAAAAAAAAAAAAAAAAAAAAAAAAAAAAAAAAAAAAAAAAAAAAAAAAAAAAAAAAAAAAAAAAAAAAAAAAAAAAAAAAAAA8D8AAAAAAAAAAAAAAAAAAPA/AAAAAAAAAAAAAAAAAAAAAAAAAAAAAAAAAAAAAAAAAAAAAAAAAAAAAAAAAAAAAAAAAAAAAAAAAAAAAAAAAAAAAAAAAAAAAAAAAAAAAAAAAAAAAAAAAAAAAAAAAAAAAAAAAAAAAAAAAAAAAAAAAAAAAAAAAAAAAAAAAAAAAAAA8D8AAAAAAAAAAAAAAAAAAAAAAAAAAAAAAAAAAAAAAAAAAAAAAAAAAAAAAAAAAAAAAAAAAAAAAAAAAAAAAAAAAPA/AAAAAAAAAAAAAAAAAAAAAAAAAAAAAAAAAAAAAAAAAAAAAAAAAADwPwAAAAAAAAAAAAAAAAAAAAAAAAAAAAAAAAAAAAAAAAAAAAAAAAAA8D8AAAAAAADwPwAAAAAAAAAAAAAAAAAA8D8AAAAAAAAAAAAAAAAAAAAAAAAAAAAA8D8AAAAAAAAAAAAAAAAAAAAAAAAAAAAAAAAAAAAAAAAAAAAAAAAAAAAAAAAAAAAA8D8AAAAAAAAAAAAAAAAAAAAAAAAAAAAA8D8AAAAAAAAAAAAAAAAAAAAAAAAAAAAAAAAAAAAAAADwPwAAAAAAAAAAAAAAAAAAAAAAAAAAAADwPwAAAAAAAPA/AAAAAAAAAAAAAAAAAADwPwAAAAAAAPA/AAAAAAAAAAAAAAAAAAAAAAAAAAAAAAAAAAAAAAAA8D8PZQAAALABAAAGAAAAAAAAAAAAAAAAAAAAAAAAAAAAAAAAAAAAAAAAAAAAAAAAAADwPwAAAAAAAPA/AAAAAAAA8D8AAAAAAAAAAAAAAAAAAPA/AAAAAAAA8D8AAAAAAADwPwAAAAAAAAAAAAAAAAAA8D8AAAAAAADwPwAAAAAAAAAAAAAAAAAA8D8AAAAAAAAAAAAAAAAAAPA/AAAAAAAA8D8AAAAAAADwPwAAAAAAAAAAAAAAAAAAAAAAAAAAAADwPwAAAAAAAPA/AAAAAAAA8D8AAAAAAADwPwAAAAAAAAAAAAAAAAAA8D8AAAAAAADwPwAAAAAAAPA/AAAAAAAA8D8AAAAAAADwPwAAAAAAAAAAAAAAAAAA8D8AAAAAAADwPwAAAAAAAAAAAAAAAAAA8D8AAAAAAAAAAAAAAAAAAPA/AAAAAAAAAAAAAAAAAADwPwAAAAAAAAAAAAAAAAAA8D8AAAAAAADwPwAAAAAAAPA/AAAAAAAA8D8AAAAAAADwPwAAAAAAAPA/AAAAAAAAAAAAAAAAAADwPwAAAAAAAAAAAAAAAAAAAAAAAAAAAAAAAAAAAAAAAAAAAAAAAAAA8D8AAAAAAADwPwAAAAAAAAAAAAAAAAAA8D8AAAAAAAAAAAAAAAAAAAAAAAAAAAAAAAAAAAAAAADwPwAAAAAAAPA/AAAAAAAAAAAAAAAAAADwPwAAAAAAAAAAAAAAAAAA8D8AAAAAAAAAAAAAAAAAAAAAAAAAAAAA8D8AAAAAAAAAAAAAAAAAAAAAAAAAAAAAAAAAAAAAAADwPwAAAAAAAAAAAAAAAAAAAAAAAAAAAAAAAAAAAAAAAPA/AAAAAAAAAAAAAAAAAAAAAAAAAAAAAAAAAAAAAAAAAAAAAAAAAADwPwAAAAAAAPA/AAAAAAAAAAAAAAAAAAAAAAAAAAAAAAAAAAAAAAAA8D8AAAAAAADwPwAAAAAAAAAAAAAAAAAA8D8AAAAAAADwPwAAAAAAAPA/AAAAAAAAAAAAAAAAAAAAAAAAAAAAAPA/AAAAAAAAAAAAAAAAAADwPwAAAAAAAAAAAAAAAAAA8D8AAAAAAAAAAAAAAAAAAAAAAAAAAAAAAAAAAAAAAAAAAAAAAAAAAPA/AAAAAAAA8D8AAAAAAADwPwAAAAAAAAAAAAAAAAAAAAAAAAAAAAAAAAAAAAAAAAAAAAAAAAAAAAAAAAAAAADwPwAAAAAAAPA/AAAAAAAA8D8AAAAAAAAAAAAAAAAAAAAAAAAAAAAAAAAAAAAAAAAAAAAAAAAAAPA/AAAAAAAAAAAAAAAAAADwPwAAAAAAAPA/AAAAAAAAAAAAAAAAAAAAAAAAAAAAAPA/AAAAAAAAAAAAAAAAAADwPwAAAAAAAPA/AAAAAAAAAAAAAAAAAAAAAAAAAAAAAAAAAAAAAAAAAAAAAAAAAAAAAAAAAAAAAPA/AAAAAAAAAAAAAAAAAAAAAAAAAAAAAPA/AAAAAAAAAAAAAAAAAAAAAAAAAAAAAAAAAAAAAAAA8D8AAAAAAAAAAAAAAAAAAAAAAAAAAAAAAAAAAAAAAAAAAAAAAAAAAPA/AAAAAAAAAAAAAAAAAAAAAAAAAAAAAAAAAAAAAAAAAAAAAAAAAADwPwAAAAAAAAAAAAAAAAAAAAAAAAAAAAAAAAAAAAAAAPA/AAAAAAAA8D8AAAAAAADwPwAAAAAAAAAAAAAAAAAA8D8AAAAAAAAAAAAAAAAAAPA/AAAAAAAAAAAAAAAAAAAAAAAAAAAAAAAAAAAAAAAAAAAAAAAAAAAAAAAAAAAAAPA/AAAAAAAAAAAAAAAAAAAAAAAAAAAAAAAAAAAAAAAAAAAAAAAAAAAAAAAAAAAAAPA/AAAAAAAAAAAAAAAAAAAAAAAAAAAAAAAAAAAAAAAAAAAAAAAAAADwPwAAAAAAAAAAAAAAAAAAAAAAAAAAAADwPwAAAAAAAAAAAAAAAAAAAAAAAAAAAADwPwAAAAAAAPA/AAAAAAAA8D8AAAAAAADwPwAAAAAAAAAAAAAAAAAA8D8AAAAAAAAAAAAAAAAAAPA/AAAAAAAAAAAAAAAAAAAAAAAAAAAAAPA/AAAAAAAA8D8AAAAAAAAAAAAAAAAAAPA/AAAAAAAAAAAAAAAAAADwPwAAAAAAAAAAAAAAAAAAAAAAAAAAAADwPwAAAAAAAAAAAAAAAAAA8D8AAAAAAAAAAAAAAAAAAAAAAAAAAAAA8D8AAAAAAAAAAAAAAAAAAPA/AAAAAAAAAAAAAAAAAAAAAAAAAAAAAAAAAAAAAAAA8D8AAAAAAADwPwAAAAAAAPA/AAAAAAAA8D8AAAAAAADwPwAAAAAAAPA/AAAAAAAAAAAAAAAAAAAAAAAAAAAAAPA/AAAAAAAAAAAAAAAAAAAAAAAAAAAAAAAAAAAAAAAAAAAAAAAAAAAAAAAAAAAAAPA/AAAAAAAAAAAAAAAAAADwPwAAAAAAAAAAAAAAAAAAAAAAAAAAAAAAAAAAAAAAAPA/AAAAAAAA8D8AAAAAAADwPwAAAAAAAAAAAAAAAAAAAAAAAAAAAADwPwAAAAAAAPA/AAAAAAAA8D8AAAAAAAAAAAAAAAAAAAAAAAAAAAAAAAAAAAAAAAAAAAAAAAAAAAAAAAAAAAAAAAAAAAAAAADwPwAAAAAAAAAAAAAAAAAA8D8AAAAAAAAAAAAAAAAAAAAAAAAAAAAAAAAAAAAAAAAAAAAAAAAAAAAAAAAAAAAA8D8AAAAAAADwPwAAAAAAAAAAAAAAAAAAAAAAAAAAAAAAAAAAAAAAAAAAAAAAAAAA8D8AAAAAAAAAAAAAAAAAAPA/AAAAAAAA8D8AAAAAAAAAAAAAAAAAAAAAAAAAAAAA8D8AAAAAAADwPwAAAAAAAAAAAAAAAAAA8D8AAAAAAADwPwAAAAAAAAAAAAAAAAAA8D8AAAAAAADwPwAAAAAAAPA/AAAAAAAAAAAAAAAAAAAAAAAAAAAAAAAAAAAAAAAAAAAAAAAAAADwPwAAAAAAAPA/AAAAAAAA8D8AAAAAAADwPwAAAAAAAPA/AAAAAAAA8D8AAAAAAADwPwAAAAAAAAAAAAAAAAAAAAAAAAAAAADwPwAAAAAAAPA/AAAAAAAAAAAAAAAAAADwPwAAAAAAAAAAAAAAAAAAAAAAAAAAAADwPwAAAAAAAAAAAAAAAAAAAAAAAAAAAAAAAAAAAAAAAAAAAAAAAAAA8D8AAAAAAADwPwAAAAAAAPA/AAAAAAAAAAAAAAAAAADwPwAAAAAAAAAAAAAAAAAAAAAAAAAAAAAAAAAAAAAAAAAAAAAAAAAAAAAAAAAAAADwPwAAAAAAAAAAAAAAAAAAAAAAAAAAAADwPwAAAAAAAPA/AAAAAAAAAAAAAAAAAAAAAAAAAAAAAPA/AAAAAAAAAAAAAAAAAAAAAAAAAAAAAAAAAAAAAAAA8D8AAAAAAAAAAAAAAAAAAAAAAAAAAAAAAAAAAAAAAAAAAAAAAAAAAAAAAAAAAAAA8D8AAAAAAAAAAAAAAAAAAPA/AAAAAAAAAAAAAAAAAADwPwAAAAAAAAAAAAAAAAAA8D8AAAAAAAAAAAAAAAAAAAAAAAAAAAAAAAAAAAAAAADwPwAAAAAAAPA/AAAAAAAAAAAAAAAAAAAAAAAAAAAAAPA/AAAAAAAAAAAAAAAAAAAAAAAAAAAAAAAAAAAAAAAAAAAAAAAAAADwPwAAAAAAAPA/AAAAAAAAAAAAAAAAAADwPwAAAAAAAAAAAAAAAAAAAAAAAAAAAADwPwAAAAAAAAAAAAAAAAAA8D8AAAAAAAAAAAAAAAAAAAAAAAAAAAAAAAAAAAAAAADwPwAAAAAAAAAAAAAAAAAAAAAAAAAAAAAAAAAAAAAAAAAAAAAAAAAA8D8AAAAAAADwPwAAAAAAAAAAAAAAAAAAAAAAAAAAAAAAAAAAAAAAAAAAAAAAAAAAAAAAAAAAAAAAAAAAAAAAAAAAAAAAAAAA8D8AAAAAAAAAAAAAAAAAAPA/AAAAAAAAAAAAAAAAAADwPwAAAAAAAAAAAAAAAAAAAAAAAAAAAADwPwAAAAAAAPA/AAAAAAAAAAAAAAAAAAAAAAAAAAAAAAAAAAAAAAAA8D8AAAAAAAAAAAAAAAAAAAAAAAAAAAAAAAAAAAAAAAAAAAAAAAAAAPA/AAAAAAAAAAAAAAAAAAAAAAAAAAAAAAAAAAAAAAAA8D8AAAAAAAAAAAAAAAAAAAAAAAAAAAAAAAAAAAAAAADwPwAAAAAAAPA/AAAAAAAA8D8AAAAAAADwPwAAAAAAAAAAAAAAAAAAAAAAAAAAAADwPwAAAAAAAAAAAAAAAAAAAAAAAAAAAADwPwAAAAAAAAAAAAAAAAAA8D8AAAAAAADwPwAAAAAAAAAAAAAAAAAAAAAAAAAAAAAAAAAAAAAAAAAAAAAAAAAA8D8AAAAAAAAAAAAAAAAAAAAAAAAAAAAAAAAAAAAAAADwPwAAAAAAAPA/AAAAAAAAAAAAAAAAAADwPwAAAAAAAAAAAAAAAAAAAAAAAAAAAAAAAAAAAAAAAAAAAAAAAAAAAAAAAAAAAAAAAAAAAAAAAAAAAAAAAAAAAAAAAAAAAAAAAAAAAAAAAAAAD2cAAACwAQAABgAAAAAAAAAAAAAAAAAAAAAAAAAAAADwPwAAAAAAAPA/AAAAAAAAAAAAAAAAAAAAAAAAAAAAAAAAAAAAAAAA8D8AAAAAAAAAAAAAAAAAAAAAAAAAAAAAAAAAAAAAAAAAAAAAAAAAAAAAAAAAAAAAAAAAAAAAAAAAAAAAAAAAAAAAAAAAAAAA8D8AAAAAAAAAAAAAAAAAAAAAAAAAAAAAAAAAAAAAAAAAAAAAAAAAAAAAAAAAAAAAAAAAAAAAAAAAAAAAAAAAAAAAAAAAAAAAAAAAAAAAAAAAAAAAAAAAAAAAAAAAAAAAAAAAAAAAAAAAAAAAAAAAAAAAAAAAAAAAAAAAAAAAAADwPwAAAAAAAAAAAAAAAAAAAAAAAAAAAAAAAAAAAAAAAAAAAAAAAAAAAAAAAAAAAAAAAAAAAAAAAAAAAAAAAAAAAAAAAAAAAADwPwAAAAAAAAAAAAAAAAAAAAAAAAAAAAAAAAAAAAAAAAAAAAAAAAAAAAAAAAAAAAAAAAAAAAAAAPA/AAAAAAAAAAAAAAAAAAAAAAAAAAAAAPA/AAAAAAAAAAAAAAAAAADwPwAAAAAAAAAAAAAAAAAAAAAAAAAAAAAAAAAAAAAAAAAAAAAAAAAAAAAAAAAAAADwPwAAAAAAAPA/AAAAAAAAAAAAAAAAAAAAAAAAAAAAAAAAAAAAAAAAAAAAAAAAAADwPwAAAAAAAAAAAAAAAAAA8D8AAAAAAADwPwAAAAAAAAAAAAAAAAAAAAAAAAAAAADwPwAAAAAAAAAAAAAAAAAAAAAAAAAAAADwPwAAAAAAAPA/AAAAAAAA8D8AAAAAAAAAAAAAAAAAAAAAAAAAAAAAAAAAAAAAAADwPwAAAAAAAAAAAAAAAAAAAAAAAAAAAAAAAAAAAAAAAPA/AAAAAAAA8D8AAAAAAAAAAAAAAAAAAAAAAAAAAAAAAAAAAAAAAAAAAAAAAAAAAAAAAAAAAAAAAAAAAAAAAAAAAAAAAAAAAAAAAAAAAAAA8D8AAAAAAAAAAAAAAAAAAPA/AAAAAAAAAAAAAAAAAADwPwAAAAAAAAAAAAAAAAAA8D8AAAAAAADwPwAAAAAAAPA/AAAAAAAA8D8AAAAAAAAAAAAAAAAAAAAAAAAAAAAAAAAAAAAAAAAAAAAAAAAAAAAAAAAAAAAA8D8AAAAAAAAAAAAAAAAAAPA/AAAAAAAAAAAAAAAAAAAAAAAAAAAAAAAAAAAAAAAA8D8AAAAAAADwPwAAAAAAAPA/AAAAAAAA8D8AAAAAAAAAAAAAAAAAAPA/AAAAAAAAAAAAAAAAAAAAAAAAAAAAAAAAAAAAAAAA8D8AAAAAAAAAAAAAAAAAAPA/AAAAAAAAAAAAAAAAAAAAAAAAAAAAAAAAAAAAAAAA8D8AAAAAAAAAAAAAAAAAAAAAAAAAAAAAAAAAAAAAAAAAAAAAAAAAAPA/AAAAAAAA8D8AAAAAAAAAAAAAAAAAAPA/AAAAAAAA8D8AAAAAAAAAAAAAAAAAAAAAAAAAAAAA8D8AAAAAAADwPwAAAAAAAPA/AAAAAAAAAAAAAAAAAAAAAAAAAAAAAAAAAAAAAAAA8D8AAAAAAAAAAAAAAAAAAAAAAAAAAAAAAAAAAAAAAAAAAAAAAAAAAAAAAAAAAAAA8D8AAAAAAAAAAAAAAAAAAAAAAAAAAAAAAAAAAAAAAADwPwAAAAAAAAAAAAAAAAAAAAAAAAAAAAAAAAAAAAAAAPA/AAAAAAAA8D8AAAAAAAAAAAAAAAAAAAAAAAAAAAAA8D8AAAAAAAAAAAAAAAAAAAAAAAAAAAAA8D8AAAAAAAAAAAAAAAAAAAAAAAAAAAAA8D8AAAAAAAAAAAAAAAAAAPA/AAAAAAAA8D8AAAAAAAAAAAAAAAAAAPA/AAAAAAAAAAAAAAAAAADwPwAAAAAAAPA/AAAAAAAAAAAAAAAAAAAAAAAAAAAAAPA/AAAAAAAAAAAAAAAAAAAAAAAAAAAAAAAAAAAAAAAAAAAAAAAAAAAAAAAAAAAAAAAAAAAAAAAAAAAAAAAAAAAAAAAAAAAAAPA/AAAAAAAA8D8AAAAAAAAAAAAAAAAAAAAAAAAAAAAA8D8AAAAAAAAAAAAAAAAAAPA/AAAAAAAAAAAAAAAAAADwPwAAAAAAAPA/AAAAAAAAAAAAAAAAAADwPwAAAAAAAAAAAAAAAAAA8D8AAAAAAADwPwAAAAAAAAAAAAAAAAAAAAAAAAAAAAAAAAAAAAAAAAAAAAAAAAAA8D8AAAAAAADwPwAAAAAAAAAAAAAAAAAAAAAAAAAAAAAAAAAAAAAAAAAAAAAAAAAAAAAAAAAAAAAAAAAAAAAAAAAAAAAAAAAAAAAAAAAAAAAAAAAAAAAAAAAAAAAAAAAA8D8AAAAAAAAAAAAAAAAAAPA/AAAAAAAA8D8AAAAAAAAAAAAAAAAAAAAAAAAAAAAAAAAAAAAAAAAAAAAAAAAAAPA/AAAAAAAAAAAAAAAAAAAAAAAAAAAAAAAAAAAAAAAAAAAAAAAAAAAAAAAAAAAAAPA/AAAAAAAAAAAAAAAAAAAAAAAAAAAAAAAAAAAAAAAAAAAAAAAAAAAAAAAAAAAAAPA/AAAAAAAA8D8AAAAAAADwPwAAAAAAAAAAAAAAAAAAAAAAAAAAAAAAAAAAAAAAAAAAAAAAAAAA8D8AAAAAAAAAAAAAAAAAAPA/AAAAAAAA8D8AAAAAAADwPwAAAAAAAAAAAAAAAAAAAAAAAAAAAADwPwAAAAAAAAAAAAAAAAAAAAAAAAAAAAAAAAAAAAAAAAAAAAAAAAAA8D8AAAAAAAAAAAAAAAAAAAAAAAAAAAAA8D8AAAAAAADwPwAAAAAAAAAAAAAAAAAAAAAAAAAAAAAAAAAAAAAAAAAAAAAAAAAAAAAAAAAAAAAAAAAAAAAAAAAAAAAAAAAAAAAAAAAAAAAAAAAAAAAAAPA/AAAAAAAAAAAAAAAAAAAAAAAAAAAAAAAAAAAAAAAAAAAAAAAAAAAAAAAAAAAAAAAAAAAAAAAAAAAAAAAAAAAAAAAAAAAAAAAAAAAAAAAAAAAAAAAAAAAAAAAAAAAAAPA/AAAAAAAAAAAAAAAAAAAAAAAAAAAAAPA/AAAAAAAAAAAAAAAAAADwPwAAAAAAAPA/AAAAAAAAAAAAAAAAAAAAAAAAAAAAAPA/AAAAAAAAAAAAAAAAAADwPwAAAAAAAAAAAAAAAAAAAAAAAAAAAAAAAAAAAAAAAPA/AAAAAAAAAAAAAAAAAADwPwAAAAAAAPA/AAAAAAAA8D8AAAAAAADwPwAAAAAAAPA/AAAAAAAAAAAAAAAAAAAAAAAAAAAAAPA/AAAAAAAAAAAAAAAAAAAAAAAAAAAAAPA/AAAAAAAAAAAAAAAAAAAAAAAAAAAAAAAAAAAAAAAA8D8AAAAAAAAAAAAAAAAAAAAAAAAAAAAA8D8AAAAAAAAAAAAAAAAAAPA/AAAAAAAA8D8AAAAAAADwPwAAAAAAAAAAAAAAAAAAAAAAAAAAAAAAAAAAAAAAAAAAAAAAAAAAAAAAAAAAAAAAAAAAAAAAAAAAAAAAAAAA8D8AAAAAAADwPwAAAAAAAPA/AAAAAAAAAAAAAAAAAAAAAAAAAAAAAAAAAAAAAAAA8D8AAAAAAAAAAAAAAAAAAAAAAAAAAAAAAAAAAAAAAADwPwAAAAAAAPA/AAAAAAAAAAAAAAAAAAAAAAAAAAAAAPA/AAAAAAAAAAAAAAAAAADwPwAAAAAAAPA/AAAAAAAAAAAAAAAAAADwPwAAAAAAAAAAAAAAAAAAAAAAAAAAAAAAAAAAAAAAAPA/AAAAAAAAAAAAAAAAAAAAAAAAAAAAAPA/AAAAAAAAAAAAAAAAAADwPwAAAAAAAAAAAAAAAAAAAAAAAAAAAAAAAAAAAAAAAPA/AAAAAAAAAAAAAAAAAADwPwAAAAAAAPA/AAAAAAAAAAAAAAAAAADwPwAAAAAAAAAAAAAAAAAAAAAAAAAAAAAAAAAAAAAAAPA/AAAAAAAAAAAAAAAAAAAAAAAAAAAAAPA/AAAAAAAAAAAAAAAAAAAAAAAAAAAAAPA/AAAAAAAA8D8AAAAAAAAAAAAAAAAAAAAAAAAAAAAA8D8AAAAAAADwPwAAAAAAAPA/AAAAAAAAAAAAAAAAAAAAAAAAAAAAAAAAAAAAAAAAAAAAAAAAAADwPwAAAAAAAAAAAAAAAAAA8D8AAAAAAADwPwAAAAAAAAAAAAAAAAAAAAAAAAAAAAAAAAAAAAAAAAAAAAAAAAAAAAAAAAAAAAAAAAAAAAAAAAAAAAAAAAAAAAAAAAAAAAAAAAAAAAAAAPA/AAAAAAAAAAAAAAAAAAAAAAAAAAAAAAAAAAAAAAAAAAAAAAAAAAAAAAAAAAAAAPA/AAAAAAAAAAAAAAAAAAAAAAAAAAAAAAAAAAAAAAAA8D8AAAAAAAAAAAAAAAAAAAAAAAAAAAAAAAAAAAAAAAAAAAAAAAAAAAAAAAAAAAAAAAAAAAAAAADwPwAAAAAAAAAAAAAAAAAAAAAAAAAAAADwPwAAAAAAAAAAAAAAAAAAAAAAAAAAAADwPwAAAAAAAPA/AAAAAAAAAAAAAAAAAAAAAA9pAAAAsAEAAAYAAAAAAAAAAAAAAAAAAAAAAAAAAAAAAAAAAAAAAAAAAAAAAAAAAAAAAAAAAAAAAAAAAAAAAAAAAAAAAAAAAAAAAAAAAAAAAAAAAAAAAAAAAAAAAAAAAAAAAAAAAAAAAAAAAAAAAAAAAAAAAAAAAAAAAAAAAAAAAAAAAAAAAAAAAAAAAAAAAAAAAAAAAAAAAAAAAAAAAAAAAAAAAAAAAAAAAAAAAAAAAAAAAAAAAAAAAAAAAAAAAAAAAAAAAAAAAAAAAAAAAAAAAAAAAAAAAAAAAAAAAAAAAAAAAAAAAAAAAAAAAAAAAAAAAAAAAAAAAAAAAAAAAAAAAAAAAAAAAAAAAAAAAAAAAAAAAAAAAAAAAAAAAAAAAAAAAAAAAAAAAAAAAAAAAAAAAAAAAAAAAAAAAAAAAAAAAAAAAAAAAAAAAAAAAAAAAAAAAAAAAAAAAAAAAAAAAAAAAAAAAAAAAAAAAAAAAAAAAAAAAAAAAAAAAAAAAAAAAAAAAAAAAAAAAAAAAAAAAAAAAAAAAAAAAAAAAAAAAAAAAAAAAAAAAAAAAAAAAAAAAAAAAAAAAAAAAAAAAAAAAAAAAAAAAAAAAAAAAAAAAAAAAAAAAAAAAAAAAAAAAAAAAAAAAAAAAAAAAAAAAAAAAAAAAAAAAAAAAAAAAAAAAAAAAAAAAAAAAAAAAAAAAAAAAAAAAAAAAAAAAAAAAAAAAAAAAAAAAAAAAAAAAAAAAAAAAAAAAAAAAAAAAAAAAAAAAAAAAAAAAAAAAAAAAAAAAAAAAAAAAAAAAAAAAAAAAAAAAAAAAAAAAAAAAAAAAAAAAAAAAAAAAAAAAAAAAAAAAAAAAAAAAAAAAPA/AAAAAAAAAAAAAAAAAAAAAAAAAAAAAAAAAAAAAAAAAAAAAAAAAAAAAAAAAAAAAAAAAAAAAAAA8D8AAAAAAAAAAAAAAAAAAAAAAAAAAAAAAAAAAAAAAAAAAAAAAAAAAAAAAAAAAAAAAAAAAAAAAAAAAAAAAAAAAAAAAAAAAAAAAAAAAAAAAAAAAAAAAAAAAAAAAAAAAAAAAAAAAAAAAAAAAAAAAAAAAAAAAAAAAAAAAAAAAAAAAAAAAAAAAAAAAAAAAAAAAAAAAAAAAAAAAAAAAAAAAAAAAAAAAAAAAAAA8D8AAAAAAAAAAAAAAAAAAAAAAAAAAAAA8D8AAAAAAAAAAAAAAAAAAAAAAAAAAAAAAAAAAAAAAAAAAAAAAAAAAAAAAAAAAAAAAAAAAAAAAAAAAAAAAAAAAAAAAAAAAAAAAAAAAAAAAAAAAAAAAAAAAAAAAAAAAAAAAAAAAAAAAAAAAAAAAAAAAAAAAAAAAAAAAAAAAAAAAAAAAAAAAAAAAAAAAAAAAAAAAAAAAAAAAADwPwAAAAAAAAAAAAAAAAAAAAAAAAAAAADwPwAAAAAAAPA/AAAAAAAAAAAAAAAAAAAAAAAAAAAAAAAAAAAAAAAAAAAAAAAAAAAAAAAAAAAAAAAAAAAAAAAA8D8AAAAAAAAAAAAAAAAAAAAAAAAAAAAAAAAAAAAAAAAAAAAAAAAAAAAAAAAAAAAAAAAAAAAAAAAAAAAAAAAAAAAAAAAAAAAAAAAAAAAAAAAAAAAAAAAAAAAAAAAAAAAAAAAAAAAAAAAAAAAAAAAAAAAAAAAAAAAAAAAAAAAAAAAAAAAAAAAAAAAAAAAAAAAAAAAAAAAAAAAAAAAAAAAAAAAAAAAAAAAAAAAAAAAAAAAAAAAAAAAAAAAAAAAAAAAAAAAAAAAAAAAAAAAAAAAAAAAAAAAAAAAAAAAAAAAAAADwPwAAAAAAAAAAAAAAAAAA8D8AAAAAAAAAAAAAAAAAAAAAAAAAAAAAAAAAAAAAAAAAAAAAAAAAAAAAAAAAAAAAAAAAAAAAAAAAAAAAAAAAAAAAAAAAAAAAAAAAAAAAAAAAAAAAAAAAAAAAAAAAAAAAAAAAAAAAAAAAAAAAAAAAAAAAAAAAAAAAAAAAAAAAAAAAAAAAAAAAAAAAAAAAAAAA8D8AAAAAAAAAAAAAAAAAAPA/AAAAAAAAAAAAAAAAAAAAAAAAAAAAAAAAAAAAAAAAAAAAAAAAAAAAAAAAAAAAAAAAAAAAAAAAAAAAAAAAAAAAAAAAAAAAAAAAAAAAAAAAAAAAAAAAAAAAAAAAAAAAAAAAAAAAAAAAAAAAAAAAAAAAAAAAAAAAAAAAAAAAAAAAAAAAAAAAAAAAAAAAAAAAAAAAAAAAAAAAAAAAAAAAAAAAAAAAAAAAAAAAAAAAAAAAAAAAAAAAAAAAAAAAAAAAAAAAAAAAAAAAAAAAAAAAAAAAAAAAAAAAAAAAAAAAAAAAAAAAAAAAAAAAAAAAAAAAAPA/AAAAAAAAAAAAAAAAAAAAAAAAAAAAAAAAAAAAAAAA8D8AAAAAAAAAAAAAAAAAAAAAAAAAAAAAAAAAAAAAAAAAAAAAAAAAAAAAAAAAAAAA8D8AAAAAAAAAAAAAAAAAAPA/AAAAAAAAAAAAAAAAAAAAAAAAAAAAAAAAAAAAAAAA8D8AAAAAAAAAAAAAAAAAAAAAAAAAAAAAAAAAAAAAAAAAAAAAAAAAAPA/AAAAAAAAAAAAAAAAAAAAAAAAAAAAAAAAAAAAAAAAAAAAAAAAAAAAAAAAAAAAAAAAAAAAAAAAAAAAAAAAAAAAAAAAAAAAAAAAAAAAAAAAAAAAAAAAAAAAAAAAAAAAAAAAAAAAAAAAAAAAAAAAAAAAAAAAAAAAAAAAAAAAAAAAAAAAAAAAAAAAAAAAAAAAAPA/AAAAAAAAAAAAAAAAAAAAAAAAAAAAAAAAAAAAAAAAAAAAAAAAAAAAAAAAAAAAAAAAAAAAAAAAAAAAAAAAAAAAAAAAAAAAAAAAAAAAAAAAAAAAAAAAAAAAAAAAAAAAAAAAAAAAAAAA8D8AAAAAAAAAAAAAAAAAAAAAAAAAAAAAAAAAAAAAAAAAAAAAAAAAAAAAAAAAAAAAAAAAAAAAAADwPwAAAAAAAAAAAAAAAAAAAAAAAAAAAAAAAAAAAAAAAAAAAAAAAAAAAAAAAAAAAAAAAAAAAAAAAAAAAAAAAAAAAAAAAAAAAAAAAAAAAAAAAAAAAAAAAAAAAAAAAAAAAAAAAAAAAAAAAAAAAAAAAAAAAAAAAAAAAAAAAAAAAAAAAAAAAAAAAAAAAAAAAAAAAAAAAAAAAAAAAAAAAAAAAAAAAAAAAAAAAAAAAAAAAAAAAAAAAAAAAAAAAAAAAAAAAAAAAAAAAAAAAAAAAAAAAAAAAAAAAAAAAAAAAAAAAAAAAAAAAAAAAAAAAAAAAAAAAAAAAAAAAAAAAAAAAAAAAAAAAAAAAAAAAAAAAAAAAAAAAAAAAAAAAAAAAAAAAAAAAAAAAAAAAAAAAAAAAAAAAAAAAAAAAAAAAADwPwAAAAAAAAAAAAAAAAAA8D8AAAAAAAAAAAAAAAAAAAAAAAAAAAAAAAAAAAAAAAAAAAAAAAAAAAAAAAAAAAAAAAAAAAAAAAAAAAAAAAAAAAAAAAAAAAAAAAAAAAAAAADwPwAAAAAAAAAAAAAAAAAA8D8AAAAAAAAAAAAAAAAAAAAAAAAAAAAAAAAAAAAAAAAAAAAAAAAAAAAAAAAAAAAAAAAAAAAAAADwPwAAAAAAAAAAAAAAAAAAAAAAAAAAAADwPwAAAAAAAPA/AAAAAAAAAAAAAAAAAAAAAAAAAAAAAAAAAAAAAAAAAAAAAAAAAAAAAAAAAAAAAAAAAAAAAAAAAAAAAAAAAAAAAAAAAAAAAAAAAAAAAAAAAAAAAAAAAAAAAAAAAAAAAPA/AAAAAAAA8D8AAAAAAAAAAAAAAAAAAAAAAAAAAAAA8D8AAAAAAAAAAAAAAAAAAPA/AAAAAAAAAAAAAAAAAAAAAAAAAAAAAAAAAAAAAAAAAAAAAAAAAAAAAAAAAAAAAAAAAAAAAAAAAAAAAAAAAAAAAAAAAAAAAPA/AAAAAAAAAAAAAAAAAAAAAAAAAAAAAPA/AAAAAAAAAAAAAAAAAAAAAAAAAAAAAAAAAAAAAAAA8D8AAAAAAAAAAAAAAAAAAAAAAAAAAAAAAAAAAAAAAAAAAAAAAAAAAAAAAAAAAAAAAAAAAAAAAAAAAAAAAAAAAAAAAAAAAAAAAAAAAAAAAAAAAAAAAAAAAPA/AAAAAAAAAAAAAAAAAADwPwAAAAAAAAAAAAAAAAAAAAAAAAAAAAAAAAAAAAAAAAAAAAAAAAAAAAAAAAAAAAAAAAAAAAAAAAAAAAAAAAAAAAAAAAAAAAAAAAAAAAAAAAAAAAAAAAAAAAAAAAAAAAAAAAAAAAAAAAAAAAAAAAAAAAAAAAAAAAAAAAAAAAAAAAAAAAAAAAAAAAAAAAAAAAAAAAAAAAAAAAAAAAAAAAAA8D8AAAAAAAAAAAAAAAAAAPA/AAAAAAAAAAAAAAAAAAAAAAAAAAAAAAAAAAAAAAAAAAAAAAAAAADwPwAAAAAAAAAAAAAAAAAAAAAAAAAAAAAAAAAAAAAAAAAAAAAAAAAAAAAAAAAAAAAAAAAAAAAAAAAAAAAAAAAAAAAAAAAAAAAAAAAAAAAAAAAAAAAAAAAAAAAAAAAAAAAAAAAAAAAAAPA/AAAAAAAAAAAFbAAAACBTdGF0c0RpcmVjdC5EYXRhLldvcmtzaGVldE9yaWdpbgkAAAAXPENvbHVtbj5rX19CYWNraW5nRmllbGQVPE1vZGU+a19fQmFja2luZ0ZpZWxkFzxUb3BSb3c+a19fQmFja2luZ0ZpZWxkFTxSb3dzPmtfX0JhY2tpbmdGaWVsZB08V29ya2Jvb2tQYXRoPmtfX0JhY2tpbmdGaWVsZB48V29ya3NoZWV0TmFtZT5rX19CYWNraW5nRmllbGQZPEhhc1RpdGxlPmtfX0JhY2tpbmdGaWVsZBw8V2FzRmlsdGVyZWQ+a19fQmFja2luZ0ZpZWxkHDxPcmlnaW5Hcm91cD5rX19CYWNraW5nRmllbGQABAAAAQEAAAAIKVN0YXRzRGlyZWN0LlV0aWxpdGllcy5EYXRhQWNxdWlzaXRpb25Nb2RlAgAAAAgIAQEIAgAAABwAAAAFc////ylTdGF0c0RpcmVjdC5VdGlsaXRpZXMuRGF0YUFjcXVpc2l0aW9uTW9kZQEAAAAHdmFsdWVfXwAIAgAAAGoAAAAAAAAAsQEAAAaOAAAALEM6XFVzZXJzXGhhbnNcQXBwRGF0YVxMb2NhbFxUZW1wXH5FeGNlbC54bHN4Bo8AAAAITWF0Y2hpbmcAAAEAAAAPbQAAALABAAAGAAAAAAAA8D8AAAAAAADwPwAAAAAAAPA/AAAAAAAA8D8AAAAAAADwPwAAAAAAAAAAAAAAAAAA8D8AAAAAAADwPwAAAAAAAPA/AAAAAAAA8D8AAAAAAAAAAAAAAAAAAPA/AAAAAAAAAAAAAAAAAADwPwAAAAAAAPA/AAAAAAAA8D8AAAAAAAAAAAAAAAAAAAAAAAAAAAAA8D8AAAAAAAAAAAAAAAAAAPA/AAAAAAAA8D8AAAAAAADwPwAAAAAAAPA/AAAAAAAA8D8AAAAAAAAAAAAAAAAAAPA/AAAAAAAAAAAAAAAAAADwPwAAAAAAAAAAAAAAAAAA8D8AAAAAAADwPwAAAAAAAAAAAAAAAAAA8D8AAAAAAADwPwAAAAAAAPA/AAAAAAAA8D8AAAAAAADwPwAAAAAAAPA/AAAAAAAA8D8AAAAAAADwPwAAAAAAAPA/AAAAAAAA8D8AAAAAAADwPwAAAAAAAPA/AAAAAAAA8D8AAAAAAAAAAAAAAAAAAPA/AAAAAAAA8D8AAAAAAADwPwAAAAAAAPA/AAAAAAAA8D8AAAAAAADwPwAAAAAAAPA/AAAAAAAA8D8AAAAAAADwPwAAAAAAAPA/AAAAAAAA8D8AAAAAAADwPwAAAAAAAPA/AAAAAAAA8D8AAAAAAAAAAAAAAAAAAPA/AAAAAAAA8D8AAAAAAADwPwAAAAAAAAAAAAAAAAAA8D8AAAAAAADwPwAAAAAAAAAAAAAAAAAA8D8AAAAAAAAAAAAAAAAAAPA/AAAAAAAA8D8AAAAAAADwPwAAAAAAAPA/AAAAAAAA8D8AAAAAAADwPwAAAAAAAPA/AAAAAAAAAAAAAAAAAAAAAAAAAAAAAPA/AAAAAAAA8D8AAAAAAADwPwAAAAAAAPA/AAAAAAAAAAAAAAAAAADwPwAAAAAAAPA/AAAAAAAA8D8AAAAAAADwPwAAAAAAAPA/AAAAAAAA8D8AAAAAAADwPwAAAAAAAPA/AAAAAAAA8D8AAAAAAADwPwAAAAAAAAAAAAAAAAAA8D8AAAAAAADwPwAAAAAAAPA/AAAAAAAA8D8AAAAAAADwPwAAAAAAAPA/AAAAAAAA8D8AAAAAAADwPwAAAAAAAPA/AAAAAAAA8D8AAAAAAADwPwAAAAAAAPA/AAAAAAAA8D8AAAAAAAAAAAAAAAAAAAAAAAAAAAAA8D8AAAAAAAAAAAAAAAAAAPA/AAAAAAAAAAAAAAAAAADwPwAAAAAAAAAAAAAAAAAA8D8AAAAAAADwPwAAAAAAAPA/AAAAAAAA8D8AAAAAAADwPwAAAAAAAPA/AAAAAAAA8D8AAAAAAADwPwAAAAAAAAAAAAAAAAAA8D8AAAAAAADwPwAAAAAAAAAAAAAAAAAA8D8AAAAAAADwPwAAAAAAAPA/AAAAAAAAAAAAAAAAAAAAAAAAAAAAAPA/AAAAAAAAAAAAAAAAAADwPwAAAAAAAPA/AAAAAAAA8D8AAAAAAAAAAAAAAAAAAPA/AAAAAAAAAAAAAAAAAADwPwAAAAAAAPA/AAAAAAAA8D8AAAAAAADwPwAAAAAAAPA/AAAAAAAA8D8AAAAAAADwPwAAAAAAAAAAAAAAAAAA8D8AAAAAAADwPwAAAAAAAPA/AAAAAAAA8D8AAAAAAAAAAAAAAAAAAPA/AAAAAAAA8D8AAAAAAADwPwAAAAAAAPA/AAAAAAAA8D8AAAAAAADwPwAAAAAAAPA/AAAAAAAA8D8AAAAAAADwPwAAAAAAAPA/AAAAAAAA8D8AAAAAAADwPwAAAAAAAPA/AAAAAAAA8D8AAAAAAADwPwAAAAAAAPA/AAAAAAAA8D8AAAAAAADwPwAAAAAAAPA/AAAAAAAA8D8AAAAAAADwPwAAAAAAAAAAAAAAAAAA8D8AAAAAAADwPwAAAAAAAPA/AAAAAAAA8D8AAAAAAADwPwAAAAAAAPA/AAAAAAAA8D8AAAAAAADwPwAAAAAAAPA/AAAAAAAA8D8AAAAAAADwPwAAAAAAAPA/AAAAAAAA8D8AAAAAAADwPwAAAAAAAPA/AAAAAAAA8D8AAAAAAADwPwAAAAAAAPA/AAAAAAAA8D8AAAAAAADwPwAAAAAAAPA/AAAAAAAA8D8AAAAAAADwPwAAAAAAAPA/AAAAAAAA8D8AAAAAAADwPwAAAAAAAPA/AAAAAAAA8D8AAAAAAADwPwAAAAAAAPA/AAAAAAAA8D8AAAAAAADwPwAAAAAAAPA/AAAAAAAA8D8AAAAAAADwPwAAAAAAAAAAAAAAAAAA8D8AAAAAAADwPwAAAAAAAPA/AAAAAAAA8D8AAAAAAADwPwAAAAAAAAAAAAAAAAAA8D8AAAAAAAAAAAAAAAAAAPA/AAAAAAAA8D8AAAAAAADwPwAAAAAAAAAAAAAAAAAA8D8AAAAAAAAAAAAAAAAAAPA/AAAAAAAA8D8AAAAAAADwPwAAAAAAAAAAAAAAAAAA8D8AAAAAAADwPwAAAAAAAAAAAAAAAAAA8D8AAAAAAAAAAAAAAAAAAAAAAAAAAAAA8D8AAAAAAADwPwAAAAAAAPA/AAAAAAAA8D8AAAAAAAAAAAAAAAAAAPA/AAAAAAAAAAAAAAAAAAAAAAAAAAAAAPA/AAAAAAAAAAAAAAAAAADwPwAAAAAAAPA/AAAAAAAA8D8AAAAAAADwPwAAAAAAAPA/AAAAAAAA8D8AAAAAAADwPwAAAAAAAPA/AAAAAAAA8D8AAAAAAADwPwAAAAAAAPA/AAAAAAAAAAAAAAAAAADwPwAAAAAAAPA/AAAAAAAA8D8AAAAAAAAAAAAAAAAAAPA/AAAAAAAA8D8AAAAAAADwPwAAAAAAAPA/AAAAAAAA8D8AAAAAAAAAAAAAAAAAAPA/AAAAAAAA8D8AAAAAAADwPwAAAAAAAAAAAAAAAAAA8D8AAAAAAADwPwAAAAAAAAAAAAAAAAAAAAAAAAAAAADwPwAAAAAAAPA/AAAAAAAA8D8AAAAAAADwPwAAAAAAAPA/AAAAAAAA8D8AAAAAAADwPwAAAAAAAPA/AAAAAAAA8D8AAAAAAADwPwAAAAAAAPA/AAAAAAAA8D8AAAAAAAAAAAAAAAAAAPA/AAAAAAAA8D8AAAAAAADwPwAAAAAAAPA/AAAAAAAA8D8AAAAAAADwPwAAAAAAAPA/AAAAAAAAAAAAAAAAAADwPwAAAAAAAPA/AAAAAAAA8D8AAAAAAADwPwAAAAAAAPA/AAAAAAAA8D8AAAAAAADwPwAAAAAAAAAAAAAAAAAA8D8AAAAAAADwPwAAAAAAAPA/AAAAAAAA8D8AAAAAAADwPwAAAAAAAAAAAAAAAAAAAAAAAAAAAADwPwAAAAAAAPA/AAAAAAAA8D8AAAAAAADwPwAAAAAAAAAAAAAAAAAA8D8AAAAAAADwPwAAAAAAAAAAAAAAAAAA8D8AAAAAAADwPwAAAAAAAPA/AAAAAAAAAAAAAAAAAADwPwAAAAAAAPA/AAAAAAAA8D8AAAAAAADwPwAAAAAAAPA/AAAAAAAA8D8AAAAAAADwPwAAAAAAAPA/AAAAAAAA8D8AAAAAAADwPwAAAAAAAPA/AAAAAAAAAAAAAAAAAAAAAAAAAAAAAPA/AAAAAAAA8D8AAAAAAAAAAAAAAAAAAPA/AAAAAAAAAAAAAAAAAADwPwAAAAAAAPA/AAAAAAAA8D8AAAAAAADwPwAAAAAAAPA/AAAAAAAA8D8AAAAAAADwPwAAAAAAAPA/AAAAAAAA8D8AAAAAAADwPwAAAAAAAPA/AAAAAAAA8D8AAAAAAADwPwAAAAAAAAAAAAAAAAAA8D8AAAAAAADwPwAAAAAAAPA/AAAAAAAA8D8AAAAAAAAAAAAAAAAAAPA/AAAAAAAA8D8AAAAAAADwPwAAAAAAAPA/AAAAAAAA8D8AAAAAAAAAAAAAAAAAAPA/AAAAAAAA8D8AAAAAAAAAAAAAAAAAAPA/AAAAAAAA8D8AAAAAAADwPwAAAAAAAPA/AAAAAAAA8D8AAAAAAADwPwAAAAAAAPA/AAAAAAAA8D8AAAAAAADwPwAAAAAAAPA/AAAAAAAA8D8AAAAAAADwPwAAAAAAAPA/AAAAAAAA8D8AAAAAAADwPwAAAAAAAAAAAAAAAAAA8D8AAAAAAADwPwAAAAAAAPA/AAAAAAAAAAAAAAAAAADwPwAAAAAAAPA/AAAAAAAA8D8AAAAAAAAAAAAAAAAAAPA/AAAAAAAA8D8AAAAAAADwPwAAAAAAAPA/AAAAAAAA8D8AAAAAAADwPwAAAAAAAPA/AAAAAAAA8D8AAAAAAADwPwAAAAAAAPA/AAAAAAAAAAAAAAAAAADwPwAAAAAAAAAAAAAAAAAA8D8AAAAAAADwPwAAAAAAAPA/AAAAAAAA8D8AAAAAAAAAAAAAAAAAAPA/AAAAAAAA8D8AAAAAAADwPwAAAAAAAPA/AAAAAAAAAAAAAAAAAAAAAAAAAAAAAPA/AAAAAAAA8D8AAAAAAADwPwAAAAAAAPA/AAAAAAAA8D8AAAAAAADwPwAAAAAAAPA/AAAAAAAA8D8AAAAAAADwPwAAAAAAAPA/AAAAAAAAAAAAAAAAAADwPwAAAAAAAPA/D28AAACwAQAABgAAAAAAAAAAAAAAAAAAAAAAAAAAAAAAAAAAAAAAAAAAAAAAAAAAAAAAAAAAAADwPwAAAAAAAAAAAAAAAAAAAAAAAAAAAAAAAAAAAAAAAAAAAAAAAAAA8D8AAAAAAAAAAAAAAAAAAPA/AAAAAAAAAAAAAAAAAAAAAAAAAAAAAAAAAAAAAAAA8D8AAAAAAADwPwAAAAAAAAAAAAAAAAAA8D8AAAAAAAAAAAAAAAAAAAAAAAAAAAAAAAAAAAAAAAAAAAAAAAAAAAAAAAAAAAAA8D8AAAAAAAAAAAAAAAAAAPA/AAAAAAAAAAAAAAAAAADwPwAAAAAAAAAAAAAAAAAAAAAAAAAAAAAAAAAAAAAAAAAAAAAAAAAAAAAAAAAAAAAAAAAAAAAAAAAAAAAAAAAAAAAAAAAAAAAAAAAAAAAAAAAAAAAAAAAAAAAAAAAAAAAAAAAAAAAAAAAAAAAAAAAAAAAAAAAAAAAAAAAAAAAAAAAAAAAAAAAA8D8AAAAAAAAAAAAAAAAAAAAAAAAAAAAAAAAAAAAAAAAAAAAAAAAAAAAAAAAAAAAAAAAAAAAAAAAAAAAAAAAAAAAAAAAAAAAAAAAAAAAAAAAAAAAAAAAAAAAAAAAAAAAAAAAAAAAAAAAAAAAAAAAAAAAAAAAAAAAA8D8AAAAAAAAAAAAAAAAAAAAAAAAAAAAAAAAAAAAAAADwPwAAAAAAAAAAAAAAAAAAAAAAAAAAAADwPwAAAAAAAAAAAAAAAAAAAAAAAAAAAAAAAAAAAAAAAAAAAAAAAAAAAAAAAAAAAAAAAAAAAAAAAAAAAAAAAAAAAAAAAAAAAAAAAAAAAAAAAAAAAAAAAAAA8D8AAAAAAAAAAAAAAAAAAAAAAAAAAAAAAAAAAAAAAAAAAAAAAAAAAPA/AAAAAAAAAAAAAAAAAAAAAAAAAAAAAAAAAAAAAAAAAAAAAAAAAAAAAAAAAAAAAAAAAAAAAAAAAAAAAAAAAAAAAAAAAAAAAAAAAAAAAAAAAAAAAAAAAADwPwAAAAAAAAAAAAAAAAAAAAAAAAAAAAAAAAAAAAAAAAAAAAAAAAAAAAAAAAAAAAAAAAAAAAAAAAAAAAAAAAAAAAAAAAAAAAAAAAAAAAAAAAAAAAAAAAAAAAAAAAAAAAAAAAAAAAAAAAAAAAAAAAAAAAAAAAAAAADwPwAAAAAAAAAAAAAAAAAA8D8AAAAAAAAAAAAAAAAAAPA/AAAAAAAAAAAAAAAAAADwPwAAAAAAAAAAAAAAAAAAAAAAAAAAAAAAAAAAAAAAAAAAAAAAAAAAAAAAAAAAAAAAAAAAAAAAAAAAAAAAAAAAAAAAAAAAAAAAAAAAAAAAAAAAAAAAAAAAAAAAAAAAAADwPwAAAAAAAAAAAAAAAAAAAAAAAAAAAAAAAAAAAAAAAPA/AAAAAAAA8D8AAAAAAAAAAAAAAAAAAPA/AAAAAAAAAAAAAAAAAAAAAAAAAAAAAAAAAAAAAAAA8D8AAAAAAAAAAAAAAAAAAPA/AAAAAAAAAAAAAAAAAAAAAAAAAAAAAAAAAAAAAAAAAAAAAAAAAAAAAAAAAAAAAAAAAAAAAAAAAAAAAAAAAADwPwAAAAAAAAAAAAAAAAAAAAAAAAAAAAAAAAAAAAAAAAAAAAAAAAAA8D8AAAAAAAAAAAAAAAAAAAAAAAAAAAAAAAAAAAAAAAAAAAAAAAAAAAAAAAAAAAAAAAAAAAAAAAAAAAAAAAAAAAAAAAAAAAAAAAAAAAAAAAAAAAAAAAAAAAAAAAAAAAAAAAAAAAAAAAAAAAAAAAAAAAAAAAAAAAAAAAAAAAAAAAAAAAAAAAAAAAAAAAAAAAAAAAAAAAAAAAAAAAAAAAAAAAAAAAAAAAAAAAAAAAAAAADwPwAAAAAAAAAAAAAAAAAAAAAAAAAAAAAAAAAAAAAAAAAAAAAAAAAAAAAAAAAAAAAAAAAAAAAAAAAAAAAAAAAAAAAAAAAAAAAAAAAAAAAAAAAAAAAAAAAAAAAAAAAAAAAAAAAAAAAAAAAAAAAAAAAAAAAAAAAAAAAAAAAAAAAAAAAAAAAAAAAAAAAAAAAAAAAAAAAAAAAAAAAAAAAAAAAAAAAAAAAAAAAAAAAAAAAAAAAAAAAAAAAAAAAAAAAAAAAAAAAAAAAAAAAAAAAAAAAAAAAAAAAAAAAAAAAAAAAAAAAAAAAAAAAAAAAAAAAAAAAAAAAAAAAAAAAAAAAAAAAAAAAAAAAAAAAAAAAAAAAAAAAAAAAAAAAAAAAAAAAAAAAAAAAAAAAAAAAAAAAAAAAAAAAAAAAAAAAAAAAAAAAAAAAAAAAAAAAAAAAAAAAAAADwPwAAAAAAAAAAAAAAAAAAAAAAAAAAAAAAAAAAAAAAAAAAAAAAAAAAAAAAAAAAAADwPwAAAAAAAAAAAAAAAAAA8D8AAAAAAAAAAAAAAAAAAAAAAAAAAAAAAAAAAAAAAAAAAAAAAAAAAAAAAAAAAAAAAAAAAAAAAADwPwAAAAAAAAAAAAAAAAAA8D8AAAAAAADwPwAAAAAAAAAAAAAAAAAAAAAAAAAAAAAAAAAAAAAAAAAAAAAAAAAA8D8AAAAAAAAAAAAAAAAAAPA/AAAAAAAA8D8AAAAAAAAAAAAAAAAAAPA/AAAAAAAAAAAAAAAAAAAAAAAAAAAAAAAAAAAAAAAAAAAAAAAAAAAAAAAAAAAAAAAAAAAAAAAAAAAAAAAAAAAAAAAAAAAAAAAAAAAAAAAAAAAAAAAAAAAAAAAAAAAAAPA/AAAAAAAAAAAAAAAAAAAAAAAAAAAAAAAAAAAAAAAA8D8AAAAAAAAAAAAAAAAAAAAAAAAAAAAAAAAAAAAAAAAAAAAAAAAAAAAAAAAAAAAA8D8AAAAAAAAAAAAAAAAAAAAAAAAAAAAAAAAAAAAAAADwPwAAAAAAAAAAAAAAAAAAAAAAAAAAAADwPwAAAAAAAPA/AAAAAAAAAAAAAAAAAAAAAAAAAAAAAAAAAAAAAAAAAAAAAAAAAAAAAAAAAAAAAAAAAAAAAAAAAAAAAAAAAAAAAAAAAAAAAAAAAAAAAAAAAAAAAAAAAAAAAAAAAAAAAAAAAAAAAAAA8D8AAAAAAAAAAAAAAAAAAAAAAAAAAAAAAAAAAAAAAAAAAAAAAAAAAAAAAAAAAAAAAAAAAAAAAAAAAAAAAAAAAPA/AAAAAAAAAAAAAAAAAAAAAAAAAAAAAAAAAAAAAAAAAAAAAAAAAAAAAAAAAAAAAAAAAAAAAAAAAAAAAAAAAAAAAAAAAAAAAAAAAAAAAAAAAAAAAAAAAAAAAAAAAAAAAAAAAAAAAAAAAAAAAAAAAADwPwAAAAAAAPA/AAAAAAAAAAAAAAAAAAAAAAAAAAAAAAAAAAAAAAAAAAAAAAAAAADwPwAAAAAAAAAAAAAAAAAAAAAAAAAAAADwPwAAAAAAAAAAAAAAAAAAAAAAAAAAAAAAAAAAAAAAAPA/AAAAAAAAAAAAAAAAAAAAAAAAAAAAAAAAAAAAAAAAAAAAAAAAAAAAAAAAAAAAAAAAAAAAAAAAAAAAAAAAAAAAAAAAAAAAAAAAAAAAAAAAAAAAAAAAAAAAAAAAAAAAAPA/AAAAAAAAAAAAAAAAAAAAAAAAAAAAAAAAAAAAAAAA8D8AAAAAAAAAAAAAAAAAAAAAAAAAAAAAAAAAAAAAAAAAAAAAAAAAAAAAAAAAAAAAAAAAAAAAAAAAAAAAAAAAAAAAAAAAAAAAAAAAAAAAAAAAAAAAAAAAAAAAAAAAAAAAAAAAAAAAAAAAAAAAAAAAAAAAAAAAAAAAAAAAAAAAAADwPwAAAAAAAAAAAAAAAAAAAAAAAAAAAAAAAAAAAAAAAAAAAAAAAAAA8D8AAAAAAAAAAAAAAAAAAAAAAAAAAAAAAAAAAAAAAAAAAAAAAAAAAAAAAAAAAAAA8D8AAAAAAAAAAAAAAAAAAAAAAAAAAAAAAAAAAAAAAAAAAAAAAAAAAAAAAAAAAAAAAAAAAAAAAAAAAAAAAAAAAAAAAAAAAAAAAAAAAAAAAAAAAAAAAAAAAAAAAAAAAAAAAAAAAAAAAAAAAAAAAAAAAAAAAAAAAAAAAAAAAAAAAAAAAAAAAAAAAAAAAAAAAAAAAAAAAAAAAADwPwAAAAAAAAAAAAAAAAAAAAAAAAAAAAAAAAAAAAAAAPA/AAAAAAAAAAAAAAAAAAAAAAAAAAAAAAAAAAAAAAAA8D8AAAAAAAAAAAAAAAAAAAAAAAAAAAAAAAAAAAAAAAAAAAAAAAAAAAAAAAAAAAAAAAAAAAAAAAAAAAAAAAAAAAAAAAAAAAAAAAAAAAAAAAAAAAAAAAAAAAAAAAAAAAAAAAAAAAAAAADwPwAAAAAAAAAAAAAAAAAAAAAAAAAAAAAAAAAAAAAAAAAAAAAAAAAA8D8AAAAAAAAAAAAAAAAAAAAAAAAAAAAAAAAAAAAAAAAAAAAAAAAAAAAAAAAAAAAA8D8AAAAAAAAAAAAAAAAAAAAAAAAAAAAAAAAAAAAAAAAAAAAAAAAAAAAAAAAAAAAAAAAAAAAAAAAAAAAAAAAAAAAAAAAAAAAAAAAAAAAAAAAAAAAAAAAAAPA/AAAAAAAAAAAAAAAAAAAAAA9xAAAAsAEAAAYAAAAAAAAAAAAAAAAAAAAAAAAAAAAAAAAAAAAAAAAAAAAAAAAAAAAAAAAAAAAAAAAAAAAAAAAAAAAAAAAAAAAAAAAAAAAAAAAAAAAAAAAAAAAAAAAAAAAAAAAAAAAAAAAAAAAAAAAAAAAAAAAAAAAAAAAAAAAAAAAAAAAAAAAAAAAAAAAAAAAAAAAAAAAAAAAAAAAAAAAAAAAAAAAAAAAAAAAAAAAAAAAAAAAAAAAAAAAAAAAAAAAAAAAAAAAAAAAAAAAAAAAAAAAAAAAAAAAAAAAAAAAAAAAAAAAAAAAAAAAAAAAAAAAAAAAAAAAAAAAAAAAAAAAAAAAAAAAAAAAAAAAAAAAA8D8AAAAAAAAAAAAAAAAAAAAAAAAAAAAAAAAAAAAAAAAAAAAAAAAAAAAAAAAAAAAAAAAAAAAAAAAAAAAAAAAAAAAAAAAAAAAAAAAAAAAAAAAAAAAAAAAAAAAAAAAAAAAAAAAAAAAAAAAAAAAAAAAAAAAAAAAAAAAAAAAAAAAAAAAAAAAAAAAAAAAAAAAAAAAAAAAAAAAAAAAAAAAAAAAAAAAAAAAAAAAAAAAAAAAAAAAAAAAAAAAAAAAAAAAAAAAAAAAAAAAAAAAAAAAAAAAAAAAAAAAAAAAAAAAAAAAAAAAAAAAAAAAAAAAAAAAAAAAAAAAAAAAAAAAAAAAAAAAAAAAAAAAAAAAAAAAAAAAAAAAAAAAAAAAAAAAAAAAAAAAAAAAAAAAAAAAAAAAAAAAAAPA/AAAAAAAAAAAAAAAAAAAAAAAAAAAAAAAAAAAAAAAAAAAAAAAAAAAAAAAAAAAAAAAAAAAAAAAAAAAAAAAAAADwPwAAAAAAAAAAAAAAAAAAAAAAAAAAAAAAAAAAAAAAAAAAAAAAAAAAAAAAAAAAAAAAAAAAAAAAAAAAAAAAAAAAAAAAAAAAAAAAAAAAAAAAAAAAAAAAAAAAAAAAAAAAAAAAAAAAAAAAAAAAAAAAAAAAAAAAAAAAAAAAAAAAAAAAAAAAAAAAAAAAAAAAAAAAAAAAAAAAAAAAAAAAAAAAAAAAAAAAAAAAAAAAAAAAAAAAAAAAAAAAAAAAAAAAAAAAAAAAAAAAAAAAAAAAAAAAAAAAAAAAAAAAAAAAAAAAAAAAAAAAAAAAAAAAAAAAAAAAAAAAAAAAAAAAAPA/AAAAAAAAAAAAAAAAAAAAAAAAAAAAAAAAAAAAAAAAAAAAAAAAAAAAAAAAAAAAAAAAAAAAAAAAAAAAAAAAAAAAAAAAAAAAAAAAAAAAAAAAAAAAAAAAAAAAAAAAAAAAAAAAAAAAAAAAAAAAAAAAAAAAAAAAAAAAAAAAAAAAAAAA8D8AAAAAAAAAAAAAAAAAAAAAAAAAAAAAAAAAAAAAAAAAAAAAAAAAAAAAAAAAAAAAAAAAAAAAAAAAAAAAAAAAAAAAAAAAAAAAAAAAAAAAAAAAAAAAAAAAAAAAAAAAAAAAAAAAAAAAAAAAAAAAAAAAAAAAAAAAAAAAAAAAAAAAAAAAAAAAAAAAAAAAAAAAAAAAAAAAAAAAAAAAAAAAAAAAAAAAAAAAAAAAAAAAAAAAAAAAAAAAAAAAAAAAAAAAAAAAAAAAAAAAAAAAAAAAAAAAAAAAAAAAAAAAAAAAAAAAAAAAAAAAAAAAAAAAAAAAAAAAAAAAAAAAAAAAAAAAAAAAAAAAAAAAAAAAAAAAAAAAAAAAAAAAAAAAAAAAAAAAAAAAAAAAAAAAAAAAAAAAAAAAAAAAAAAAAAAAAAAAAAAAAAAAAAAAAAAAAAAAAAAAAAAAAAAAAAAAAAAAAAAAAAAAAAAAAAAAAAAAAAAAAAAAAAAAAAAAAAAAAAAAAAAAAAAAAAAAAAAAAAAAAAAAAAAAAAAAAAAAAAAAAAAAAAAAAAAAAAAAAAAAAAAAAAAAAAAAAAAAAAAAAAAAAAAAAAAAAAAAAAAAAAAAAAAAAAAAAAAAAAAAAAAAAAAAAAAAAAAAAAAAAAAAAAAAAAAAAAAAAAAAAAAAAAAAAAAAAAAAAAAAAAAAAAAAAAAAAAAAAAAAAAAAAAAAAAAAAAAAAAAAAAAAAAAAAAAAAAAAAAAAAAAAAAAAAAAAAAAAAAAAAAAAAAAAAAAAAAAAAAAAAAAAAAAAAAAAAAAAAAAAAAAAAAAAAAAAAAAAAAAAAAAAAAAAAAAAAAAAAAAAAAAAAAAAAAAAAAAAAAAAAAAAAAAAAAAAAAAAAAAAAAAAAAAAAAAAAAAAAAAAAAAAAAAAAAAAAAAAAAAAAAAA8D8AAAAAAAAAAAAAAAAAAAAAAAAAAAAAAAAAAAAAAAAAAAAAAAAAAAAAAAAAAAAAAAAAAAAAAAAAAAAAAAAAAPA/AAAAAAAAAAAAAAAAAAAAAAAAAAAAAAAAAAAAAAAAAAAAAAAAAAAAAAAAAAAAAAAAAAAAAAAAAAAAAAAAAAAAAAAAAAAAAAAAAAAAAAAA8D8AAAAAAAAAAAAAAAAAAAAAAAAAAAAAAAAAAAAAAAAAAAAAAAAAAAAAAAAAAAAAAAAAAAAAAAAAAAAAAAAAAAAAAAAAAAAAAAAAAAAAAAAAAAAAAAAAAAAAAAAAAAAAAAAAAAAAAAAAAAAAAAAAAAAAAAAAAAAAAAAAAAAAAAAAAAAAAAAAAAAAAAAAAAAAAAAAAAAAAAAAAAAAAAAAAAAAAAAAAAAAAAAAAAAAAAAAAAAAAAAAAAAAAAAAAAAAAAAAAAAAAAAAAAAAAAAAAAAAAAAAAAAAAAAAAAAAAAAAAAAAAAAAAAAAAAAAAAAAAAAAAAAAAAAAAAAAAAAAAAAAAAAAAAAAAAAAAAAAAAAAAAAAAAAAAAAAAAAAAAAAAAAAAAAAAAAAAAAAAAAAAAAAAAAAAAAAAAAAAAAAAAAAAAAAAAAAAAAAAAAAAAAAAAAAAAAAAAAAAAAAAAAAAAAAAAAAAAAAAAAAAAAAAAAAAAAAAAAAAAAAAAAAAAAAAAAAAAAAAAAAAAAAAAAAAAAAAAAAAAAAAAAAAAAAAAAAAAAAAAAAAAAAAAAAAAAAAAAAAAAAAAAAAAAAAAAAAAAAAAAAAAAAAAAAAAAAAAAAAAAAAAAAAAAAAAAAAAAAAAAAAAAAAAAAAAAAAAAAAAAAAAAAAAAAAAAAAAAAAAAAAAAAAAAAAAAAAAAAAAAAAAAAAAAAAAAAAAAAAAAAAAAAAAAAAAAAAAAAAAAAAAAAAAAAAAAAAAAAAAAAAAAAAAAAAAAAAAAAAAAAAAAAAAAAAAAAAAAA8D8AAAAAAAAAAAAAAAAAAAAAAAAAAAAAAAAAAAAAAAAAAAAAAAAAAAAAAAAAAAAAAAAAAAAAAAAAAAAAAAAAAAAAAAAAAAAAAAAAAAAAAAAAAAAAAAAAAAAAAAAAAAAAAAAAAAAAAAAAAAAAAAAAAAAAAAAAAAAAAAAAAAAAAAAAAAAAAAAAAAAAAAAAAAAAAAAAAAAAAAAAAAAAAAAAAAAAAAAAAAAAAAAAAAAAAAAAAAAAAAAAAAAAAAAAAAAAAAAAAAAAAAAAAAAAAAAAAAAAAAAAAAAAAAAAAAAAAAAAAAAAAAAAAAAAAAAAAAAAAAAAAAAAAAAAAAAAAAAAAPA/AAAAAAAAAAAAAAAAAAAAAAAAAAAAAAAAAAAAAAAAAAAAAAAAAADwPwAAAAAAAAAAAAAAAAAAAAAAAAAAAAAAAAAAAAAAAAAAAAAAAAAAAAAAAAAAAAAAAAAAAAAAAAAAAAAAAAAAAAAAAAAAAAAAAAAAAAAAAAAAAAAAAAAAAAAAAAAAAAAAAAAAAAAAAAAAAAAAAAAAAAAAAAAAAAAAAAAAAAAAAAAAAAAAAAAAAAAAAAAAAAAAAAAAAAAAAAAAAAAAAAAAAAAAAAAAAAAAAAAAAAAAAAAAAAAAAAAAAAAAAAAAAAAAAAAAAAAAAAAAAAAAAAAAAAAAAAAAAAAAAAAAAAAAAPA/AAAAAAAAAAAAAAAAAAAAAAAAAAAAAAAAAAAAAAAAAAAAAAAAAAAAAAAAAAAAAAAAAAAAAAAAAAAAAAAAAAAAAAAAAAAAAAAAAAAAAAAAAAAAAAAAAAAAAAAAAAAAAAAAAAAAAAAAAAAAAAAAAAAAAAAAAAAAAAAAAAAAAAAAAAAAAAAAAAAAAAAAAAAAAAAAAAAAAAAAAAAAAAAAAAAAAAAAAAAAAAAAAAAAAAAAAAAAAAAAAAAAAAAAAAAAAAAAAAAAAAAAAAAAAAAAAAAAAAAAAAAAAAAAAAAAAAAAAAAAAAAAAAAAAAAAAAAAAAAAAAAAAAAAAAAAAAAAAAAAAAAAAAAAAAAAAAAAAAAAAAAAAAAAAADwPwAAAAAAAAAAAAAAAAAAAAAAAAAAAAAAAAAAAAAAAAAAAAAAAAAAAAAAAAAAAAAAAAAAAAAAAAAAAAAAAAAAAAAAAAAAAAAAAAAAAAAAAAAAAAAAAAAAAAAAAAAAAADwPwAAAAAAAAAAAAAAAAAAAAAAAAAAAAAAAAAAAAAAAAAAAAAAAAAAAAAAAAAAAAAAAAAAAAAAAAAAAAAAAAAAAAAAAAAAAAAAAAAAAAAAAAAAAAAAAAAAAAAAAAAAAAAAAAAAAAAAAAAAAAAAAAAAAAABdAAAAGwAAAAEAAAAAXD///9z////agAAAAAAAACxAQAACY4AAAAGkgAAAAhNYXRjaGluZwAAAQAAAAF2AAAAbAAAAAMAAAABbf///3P///9qAAAAAAAAALEBAAAJjgAAAAaVAAAACE1hdGNoaW5nAAABAAAAAXgAAABsAAAAGgAAAAFq////c////wIAAAAAAAAAsQEAAAmOAAAABpgAAAAITWF0Y2hpbmcAAAAAAAAL <-!redo!!

**Logistic regression**

Warning: result not of full rank, there is more than one solution for the model. Look for correlated predictor variables that you might drop: the mutliple linear regression function does this automatically.

| Deviance goodness of fit chi-square = | 322,438185 | df = 261 | P = 0,0057 | * |
| --- | --- | --- | --- | --- |
| Deviance (likelihood ratio) chi-square = | 46,684531 | df = 8 | P < 0,0001 |  |

| Parameter | Odds Ratio | 95% Conf. Int. | Z Value | P (>\|Z\|) |
| --- | --- | --- | --- | --- |
| (intercept) | n/a |  | -5,191919 | P < 0,0001 |
| Valve or ring size(21) | 0,189768 | (0,095906 to 0,375491) | -4,773165 | P < 0,0001 |
| Valve or ring size(23) | 0,229466 | (0,137033 to 0,384248) | -5,596262 | P < 0,0001 |
| Valve or ring size(25) | 0,529044 | (0,322948 to 0,866664) | -2,528219 | P = 0,0115 |
| Valve or ring size(27) | 1,172803 | (0,626707 to 2,194752) | 0,498524 | P = 0,6181 |
| Cardiac procedures | 1,08389 | (0,677882 to 1,733071) | 0,336406 | P = 0,7366 |
| EF(1) | 0,280887 | (0,151845 to 0,519594) | -4,046147 | P < 0,0001 |
| EF(2) | 0,217244 | (0,104993 to 0,449505) | -4,115301 | P < 0,0001 |
| EF(3) | 0,442771 | (0,1715 to 1,143129) | -1,683537 | P = 0,0923 |
| Gender | 2,73848 | (1,52732 to 4,910086) | 3,381594 | P = 0,0007 |
| Age at operation | 1,069231 | (1,038213 to 1,101177) | 4,456626 | P < 0,0001 |

logit Valve type = -3,611241 -1,661953 Valve or ring size(21) -1,472001 Valve or ring size(23) -0,636684 Valve or ring size(25) +0,159396 Valve or ring size(27) +0,080557 Cardiac procedures -1,269803 EF(1) -1,526736 EF(2) -0,814703 EF(3) +1,007403 Gender +0,06694 Age at operation

!!help!-> 1043 <-!help!! !!redo!-> "UnivariateSummary" AAEAAAD/////AQAAAAAAAAAMAgAAAEJTdGF0c0RpcmVjdCwgVmVyc2lvbj0zLjMuNS4wLCBDdWx0dXJlPW5ldXRyYWwsIFB1YmxpY0tleVRva2VuPW51bGwFAQAAACJTdGF0c0RpcmVjdC5UZW1wbGF0ZXMuUGFyYW1ldGVyQmFnAQAAABBmaWxsZWRQYXJhbWV0ZXJzA/EBU3lzdGVtLkNvbGxlY3Rpb25zLkdlbmVyaWMuRGljdGlvbmFyeWAyW1tTeXN0ZW0uU3RyaW5nLCBtc2NvcmxpYiwgVmVyc2lvbj00LjAuMC4wLCBDdWx0dXJlPW5ldXRyYWwsIFB1YmxpY0tleVRva2VuPWI3N2E1YzU2MTkzNGUwODldLFtTdGF0c0RpcmVjdC5UZW1wbGF0ZXMuRmlsbGVkUGFyYW1ldGVyLCBTdGF0c0RpcmVjdCwgVmVyc2lvbj0zLjMuNS4wLCBDdWx0dXJlPW5ldXRyYWwsIFB1YmxpY0tleVRva2VuPW51bGxdXQIAAAAJAwAAAAQDAAAA8QFTeXN0ZW0uQ29sbGVjdGlvbnMuR2VuZXJpYy5EaWN0aW9uYXJ5YDJbW1N5c3RlbS5TdHJpbmcsIG1zY29ybGliLCBWZXJzaW9uPTQuMC4wLjAsIEN1bHR1cmU9bmV1dHJhbCwgUHVibGljS2V5VG9rZW49Yjc3YTVjNTYxOTM0ZTA4OV0sW1N0YXRzRGlyZWN0LlRlbXBsYXRlcy5GaWxsZWRQYXJhbWV0ZXIsIFN0YXRzRGlyZWN0LCBWZXJzaW9uPTMuMy41LjAsIEN1bHR1cmU9bmV1dHJhbCwgUHVibGljS2V5VG9rZW49bnVsbF1dBAAAAAdWZXJzaW9uCENvbXBhcmVyCEhhc2hTaXplDUtleVZhbHVlUGFpcnMAAwADCJIBU3lzdGVtLkNvbGxlY3Rpb25zLkdlbmVyaWMuR2VuZXJpY0VxdWFsaXR5Q29tcGFyZXJgMVtbU3lzdGVtLlN0cmluZywgbXNjb3JsaWIsIFZlcnNpb249NC4wLjAuMCwgQ3VsdHVyZT1uZXV0cmFsLCBQdWJsaWNLZXlUb2tlbj1iNzdhNWM1NjE5MzRlMDg5XV0I9QFTeXN0ZW0uQ29sbGVjdGlvbnMuR2VuZXJpYy5LZXlWYWx1ZVBhaXJgMltbU3lzdGVtLlN0cmluZywgbXNjb3JsaWIsIFZlcnNpb249NC4wLjAuMCwgQ3VsdHVyZT1uZXV0cmFsLCBQdWJsaWNLZXlUb2tlbj1iNzdhNWM1NjE5MzRlMDg5XSxbU3RhdHNEaXJlY3QuVGVtcGxhdGVzLkZpbGxlZFBhcmFtZXRlciwgU3RhdHNEaXJlY3QsIFZlcnNpb249My4zLjUuMCwgQ3VsdHVyZT1uZXV0cmFsLCBQdWJsaWNLZXlUb2tlbj1udWxsXV1bXRsAAAAJBAAAACUAAAAJBQAAAAQEAAAAkgFTeXN0ZW0uQ29sbGVjdGlvbnMuR2VuZXJpYy5HZW5lcmljRXF1YWxpdHlDb21wYXJlcmAxW1tTeXN0ZW0uU3RyaW5nLCBtc2NvcmxpYiwgVmVyc2lvbj00LjAuMC4wLCBDdWx0dXJlPW5ldXRyYWwsIFB1YmxpY0tleVRva2VuPWI3N2E1YzU2MTkzNGUwODldXQAAAAAHBQAAAAABAAAAGwAAAAPzAVN5c3RlbS5Db2xsZWN0aW9ucy5HZW5lcmljLktleVZhbHVlUGFpcmAyW1tTeXN0ZW0uU3RyaW5nLCBtc2NvcmxpYiwgVmVyc2lvbj00LjAuMC4wLCBDdWx0dXJlPW5ldXRyYWwsIFB1YmxpY0tleVRva2VuPWI3N2E1YzU2MTkzNGUwODldLFtTdGF0c0RpcmVjdC5UZW1wbGF0ZXMuRmlsbGVkUGFyYW1ldGVyLCBTdGF0c0RpcmVjdCwgVmVyc2lvbj0zLjMuNS4wLCBDdWx0dXJlPW5ldXRyYWwsIFB1YmxpY0tleVRva2VuPW51bGxdXQT6////8wFTeXN0ZW0uQ29sbGVjdGlvbnMuR2VuZXJpYy5LZXlWYWx1ZVBhaXJgMltbU3lzdGVtLlN0cmluZywgbXNjb3JsaWIsIFZlcnNpb249NC4wLjAuMCwgQ3VsdHVyZT1uZXV0cmFsLCBQdWJsaWNLZXlUb2tlbj1iNzdhNWM1NjE5MzRlMDg5XSxbU3RhdHNEaXJlY3QuVGVtcGxhdGVzLkZpbGxlZFBhcmFtZXRlciwgU3RhdHNEaXJlY3QsIFZlcnNpb249My4zLjUuMCwgQ3VsdHVyZT1uZXV0cmFsLCBQdWJsaWNLZXlUb2tlbj1udWxsXV0CAAAAA2tleQV2YWx1ZQEELlN0YXRzRGlyZWN0LlRlbXBsYXRlcy5GaWxsZWREYXRhRnJhbWVQYXJhbWV0ZXICAAAABgcAAAAEZGF0YQkIAAAAAff////6////BgoAAAATcmVwb3J0LWNlbnRpbGUtdHlwZQkLAAAAAfT////6////Bg0AAAALcmVwb3J0LXVkY2EJDgAAAAHx////+v///wYQAAAAC3JlcG9ydC11ZGNiCREAAAAB7v////r///8GEwAAAA9vdXRwdXQtdG8tZnJhbWUJFAAAAAHr////+v///wYWAAAABWdhbW1hCRcAAAAB6P////r///8GGQAAABFyZXBvcnQtdmFsaWQtZGF0YQkaAAAAAeX////6////BhwAAAATcmVwb3J0LW1pc3NpbmctZGF0YQkdAAAAAeL////6////Bh8AAAAKcmVwb3J0LXN1bQkgAAAAAd/////6////BiIAAAALcmVwb3J0LW1lYW4JIwAAAAHc////+v///wYlAAAAD3JlcG9ydC12YXJpYW5jZQkmAAAAAdn////6////BigAAAAJcmVwb3J0LXNkCSkAAAAB1v////r///8GKwAAABVyZXBvcnQtdmFyaWFuY2UtY29lZmYJLAAAAAHT////+v///wYuAAAACnJlcG9ydC1zZW0JLwAAAAHQ////+v///wYxAAAADHJlcG9ydC11OTVjbAkyAAAAAc3////6////BjQAAAAMcmVwb3J0LWw5NWNsCTUAAAAByv////r///8GNwAAABVyZXBvcnQtZ2VvbWV0cmljLW1lYW4JOAAAAAHH////+v///wY6AAAAD3JlcG9ydC1za2V3bmVzcwk7AAAAAcT////6////Bj0AAAAPcmVwb3J0LWt1cnRvc2lzCT4AAAABwf////r///8GQAAAAA5yZXBvcnQtbWF4aW11bQlBAAAAAb7////6////BkMAAAAJcmVwb3J0LXVxCUQAAAABu/////r///8GRgAAAA1yZXBvcnQtbWVkaWFuCUcAAAABuP////r///8GSQAAAAlyZXBvcnQtbHEJSgAAAAG1////+v///wZMAAAACnJlcG9ydC1pcXIJTQAAAAGy////+v///wZPAAAADnJlcG9ydC1taW5pbXVtCVAAAAABr/////r///8GUgAAAAxyZXBvcnQtcmFuZ2UJUwAAAAGs////+v///wZVAAAACnJlcG9ydC11ZGMJVgAAAAUIAAAALlN0YXRzRGlyZWN0LlRlbXBsYXRlcy5GaWxsZWREYXRhRnJhbWVQYXJhbWV0ZXICAAAAFTxEYXRhPmtfX0JhY2tpbmdGaWVsZCpGaWxsZWRQYXJhbWV0ZXIrPERpcmVjdGlvbj5rX19CYWNraW5nRmllbGQEBBpTdGF0c0RpcmVjdC5EYXRhLkRhdGFGcmFtZQIAAAAuU3RhdHNEaXJlY3QuVGVtcGxhdGVzLkZpbGxlZFBhcmFtZXRlckRpcmVjdGlvbgIAAAACAAAACVcAAAAFqP///y5TdGF0c0RpcmVjdC5UZW1wbGF0ZXMuRmlsbGVkUGFyYW1ldGVyRGlyZWN0aW9uAQAAAAd2YWx1ZV9fAAgCAAAAAQAAAAULAAAAK1N0YXRzRGlyZWN0LlRlbXBsYXRlcy5GaWxsZWRTdHJpbmdQYXJhbWV0ZXICAAAAFTxEYXRhPmtfX0JhY2tpbmdGaWVsZCpGaWxsZWRQYXJhbWV0ZXIrPERpcmVjdGlvbj5rX19CYWNraW5nRmllbGQBBC5TdGF0c0RpcmVjdC5UZW1wbGF0ZXMuRmlsbGVkUGFyYW1ldGVyRGlyZWN0aW9uAgAAAAIAAAAGWQAAAAExAab///+o////AQAAAAUOAAAAK1N0YXRzRGlyZWN0LlRlbXBsYXRlcy5GaWxsZWREb3VibGVQYXJhbWV0ZXICAAAAFTxEYXRhPmtfX0JhY2tpbmdGaWVsZCpGaWxsZWRQYXJhbWV0ZXIrPERpcmVjdGlvbj5rX19CYWNraW5nRmllbGQABAYuU3RhdHNEaXJlY3QuVGVtcGxhdGVzLkZpbGxlZFBhcmFtZXRlckRpcmVjdGlvbgIAAAACAAAAmpmZmZmZqT8Bpf///6j///8BAAAAAREAAAAOAAAAZmZmZmZm7j8BpP///6j///8BAAAABRQAAAAsU3RhdHNEaXJlY3QuVGVtcGxhdGVzLkZpbGxlZEJvb2xlYW5QYXJhbWV0ZXICAAAAFTxEYXRhPmtfX0JhY2tpbmdGaWVsZCpGaWxsZWRQYXJhbWV0ZXIrPERpcmVjdGlvbj5rX19CYWNraW5nRmllbGQABAEuU3RhdHNEaXJlY3QuVGVtcGxhdGVzLkZpbGxlZFBhcmFtZXRlckRpcmVjdGlvbgIAAAACAAAAAAGj////qP///wEAAAABFwAAAA4AAABmZmZmZmbuPwGi////qP///wEAAAABGgAAABQAAAABAaH///+o////AQAAAAEdAAAAFAAAAAEBoP///6j///8BAAAAASAAAAAUAAAAAQGf////qP///wEAAAABIwAAABQAAAABAZ7///+o////AQAAAAEmAAAAFAAAAAEBnf///6j///8BAAAAASkAAAAUAAAAAQGc////qP///wEAAAABLAAAABQAAAABAZv///+o////AQAAAAEvAAAAFAAAAAEBmv///6j///8BAAAAATIAAAAUAAAAAQGZ////qP///wEAAAABNQAAABQAAAABAZj///+o////AQAAAAE4AAAAFAAAAAEBl////6j///8BAAAAATsAAAAUAAAAAQGW////qP///wEAAAABPgAAABQAAAABAZX///+o////AQAAAAFBAAAAFAAAAAEBlP///6j///8BAAAAAUQAAAAUAAAAAQGT////qP///wEAAAABRwAAABQAAAABAZL///+o////AQAAAAFKAAAAFAAAAAEBkf///6j///8BAAAAAU0AAAAUAAAAAQGQ////qP///wEAAAABUAAAABQAAAABAY////+o////AQAAAAFTAAAAFAAAAAEBjv///6j///8BAAAAAVYAAAAUAAAAAQGN////qP///wEAAAAFVwAAABpTdGF0c0RpcmVjdC5EYXRhLkRhdGFGcmFtZQIAAAAVPE5hbWU+a19fQmFja2luZ0ZpZWxkGjxWYXJpYWJsZXM+a19fQmFja2luZ0ZpZWxkAQODAVN5c3RlbS5Db2xsZWN0aW9ucy5HZW5lcmljLkxpc3RgMVtbU3RhdHNEaXJlY3QuRGF0YS5JVmFyaWFibGUsIFN0YXRzRGlyZWN0LCBWZXJzaW9uPTMuMy41LjAsIEN1bHR1cmU9bmV1dHJhbCwgUHVibGljS2V5VG9rZW49bnVsbF1dAgAAAAZ0AAAABkRhdGEgMQl1AAAABHUAAACDAVN5c3RlbS5Db2xsZWN0aW9ucy5HZW5lcmljLkxpc3RgMVtbU3RhdHNEaXJlY3QuRGF0YS5JVmFyaWFibGUsIFN0YXRzRGlyZWN0LCBWZXJzaW9uPTMuMy41LjAsIEN1bHR1cmU9bmV1dHJhbCwgUHVibGljS2V5VG9rZW49bnVsbF1dAwAAAAZfaXRlbXMFX3NpemUIX3ZlcnNpb24EAAAcU3RhdHNEaXJlY3QuRGF0YS5JVmFyaWFibGVbXQIAAAAICAl2AAAAAgAAAAIAAAAHdgAAAAABAAAABAAAAAQaU3RhdHNEaXJlY3QuRGF0YS5JVmFyaWFibGUCAAAACXcAAAAJeAAAAA0CBXcAAAAfU3RhdHNEaXJlY3QuRGF0YS5Eb3VibGVWYXJpYWJsZQcAAAADc3VtA21pbgNtYXgMaGFzU3VtbWFyaWVzFkdlbmVyaWNWYXJpYWJsZWAxK2RhdGEoR2VuZXJpY1ZhcmlhYmxlYDErPFRpdGxlPmtfX0JhY2tpbmdGaWVsZClHZW5lcmljVmFyaWFibGVgMSs8T3JpZ2luPmtfX0JhY2tpbmdGaWVsZAAAAAAHAQQGBgYBBiBTdGF0c0RpcmVjdC5EYXRhLldvcmtzaGVldE9yaWdpbgIAAAACAAAAAAAAAAAAAAAAAAAAAAAAAAAAAAAAAAAAAAoGeQAAAA9TY29yZSBQZXJpbW91bnQJegAAAAF4AAAAdwAAAAAAAAAAAAAAAAAAAAAAAAAAAAAAAAAAAAAKBnsAAAAOU2NvcmUgUGVyY2V2YWwJfAAAAAV6AAAAIFN0YXRzRGlyZWN0LkRhdGEuV29ya3NoZWV0T3JpZ2luCQAAABc8Q29sdW1uPmtfX0JhY2tpbmdGaWVsZBU8TW9kZT5rX19CYWNraW5nRmllbGQXPFRvcFJvdz5rX19CYWNraW5nRmllbGQVPFJvd3M+a19fQmFja2luZ0ZpZWxkHTxXb3JrYm9va1BhdGg+a19fQmFja2luZ0ZpZWxkHjxXb3Jrc2hlZXROYW1lPmtfX0JhY2tpbmdGaWVsZBk8SGFzVGl0bGU+a19fQmFja2luZ0ZpZWxkHDxXYXNGaWx0ZXJlZD5rX19CYWNraW5nRmllbGQcPE9yaWdpbkdyb3VwPmtfX0JhY2tpbmdGaWVsZAAEAAABAQAAAAgpU3RhdHNEaXJlY3QuVXRpbGl0aWVzLkRhdGFBY3F1aXNpdGlvbk1vZGUCAAAACAgBAQgCAAAAPAAAAAWD////KVN0YXRzRGlyZWN0LlV0aWxpdGllcy5EYXRhQWNxdWlzaXRpb25Nb2RlAQAAAAd2YWx1ZV9fAAgCAAAAAgAAAAAAAACBAAAABn4AAAAsQzpcVXNlcnNcaGFuc1xBcHBEYXRhXExvY2FsXFRlbXBcfkV4Y2VsLnhsc3gGfwAAAA1NYXRjaGVkIFBhaXJzAAAAAAAAAXwAAAB6AAAAPQAAAAGA////g////wIAAAAAAAAAgQAAAAl+AAAABoIAAAANTWF0Y2hlZCBQYWlycwAAAAAAAAs= <-!redo!!

**Descriptive statistics**

| Variables | Score Perimount | Score Perceval |
| --- | --- | --- |
| Valid data | 128 | 128 |
| Missing data | 0 | 0 |
| Sum | 46,426386 | 47,380416 |
| Mean | 0,362706 | 0,37016 |
| Variance | 0,018769 | 0,019928 |
| Standard deviation | 0,136998 | 0,141166 |
| Variance coefficient | 0,377711 | 0,381366 |
| Standard error of mean | 0,012109 | 0,012477 |
| Upper 95% CL of mean | 0,386668 | 0,39485 |
| Lower 95% CL of mean | 0,338745 | 0,345469 |
| Geometric mean | 0,329578 | 0,335938 |
| Skewness | -0,209736 | -0,16154 |
| Kurtosis | 2,396425 | 2,390138 |
| Maximum | 0,640478 | 0,640403 |
| Upper quartile | 0,459457 | 0,472996 |
| Median | 0,387218 | 0,374514 |
| Lower quartile | 0,269469 | 0,270758 |
| Interquartile range | 0,189989 | 0,202239 |
| Minimum | 0,050254 | 0,050254 |
| Range | 0,590224 | 0,590149 |
| Centile 95 | 0,579244 | 0,606526 |
| Centile 5 | 0,122399 | 0,120143 |

!!help!-> 1043 <-!help!! !!redo!-> "UnivariateSummary" AAEAAAD/////AQAAAAAAAAAMAgAAAEJTdGF0c0RpcmVjdCwgVmVyc2lvbj0zLjMuNS4wLCBDdWx0dXJlPW5ldXRyYWwsIFB1YmxpY0tleVRva2VuPW51bGwFAQAAACJTdGF0c0RpcmVjdC5UZW1wbGF0ZXMuUGFyYW1ldGVyQmFnAQAAABBmaWxsZWRQYXJhbWV0ZXJzA/EBU3lzdGVtLkNvbGxlY3Rpb25zLkdlbmVyaWMuRGljdGlvbmFyeWAyW1tTeXN0ZW0uU3RyaW5nLCBtc2NvcmxpYiwgVmVyc2lvbj00LjAuMC4wLCBDdWx0dXJlPW5ldXRyYWwsIFB1YmxpY0tleVRva2VuPWI3N2E1YzU2MTkzNGUwODldLFtTdGF0c0RpcmVjdC5UZW1wbGF0ZXMuRmlsbGVkUGFyYW1ldGVyLCBTdGF0c0RpcmVjdCwgVmVyc2lvbj0zLjMuNS4wLCBDdWx0dXJlPW5ldXRyYWwsIFB1YmxpY0tleVRva2VuPW51bGxdXQIAAAAJAwAAAAQDAAAA8QFTeXN0ZW0uQ29sbGVjdGlvbnMuR2VuZXJpYy5EaWN0aW9uYXJ5YDJbW1N5c3RlbS5TdHJpbmcsIG1zY29ybGliLCBWZXJzaW9uPTQuMC4wLjAsIEN1bHR1cmU9bmV1dHJhbCwgUHVibGljS2V5VG9rZW49Yjc3YTVjNTYxOTM0ZTA4OV0sW1N0YXRzRGlyZWN0LlRlbXBsYXRlcy5GaWxsZWRQYXJhbWV0ZXIsIFN0YXRzRGlyZWN0LCBWZXJzaW9uPTMuMy41LjAsIEN1bHR1cmU9bmV1dHJhbCwgUHVibGljS2V5VG9rZW49bnVsbF1dBAAAAAdWZXJzaW9uCENvbXBhcmVyCEhhc2hTaXplDUtleVZhbHVlUGFpcnMAAwADCJIBU3lzdGVtLkNvbGxlY3Rpb25zLkdlbmVyaWMuR2VuZXJpY0VxdWFsaXR5Q29tcGFyZXJgMVtbU3lzdGVtLlN0cmluZywgbXNjb3JsaWIsIFZlcnNpb249NC4wLjAuMCwgQ3VsdHVyZT1uZXV0cmFsLCBQdWJsaWNLZXlUb2tlbj1iNzdhNWM1NjE5MzRlMDg5XV0I9QFTeXN0ZW0uQ29sbGVjdGlvbnMuR2VuZXJpYy5LZXlWYWx1ZVBhaXJgMltbU3lzdGVtLlN0cmluZywgbXNjb3JsaWIsIFZlcnNpb249NC4wLjAuMCwgQ3VsdHVyZT1uZXV0cmFsLCBQdWJsaWNLZXlUb2tlbj1iNzdhNWM1NjE5MzRlMDg5XSxbU3RhdHNEaXJlY3QuVGVtcGxhdGVzLkZpbGxlZFBhcmFtZXRlciwgU3RhdHNEaXJlY3QsIFZlcnNpb249My4zLjUuMCwgQ3VsdHVyZT1uZXV0cmFsLCBQdWJsaWNLZXlUb2tlbj1udWxsXV1bXRsAAAAJBAAAACUAAAAJBQAAAAQEAAAAkgFTeXN0ZW0uQ29sbGVjdGlvbnMuR2VuZXJpYy5HZW5lcmljRXF1YWxpdHlDb21wYXJlcmAxW1tTeXN0ZW0uU3RyaW5nLCBtc2NvcmxpYiwgVmVyc2lvbj00LjAuMC4wLCBDdWx0dXJlPW5ldXRyYWwsIFB1YmxpY0tleVRva2VuPWI3N2E1YzU2MTkzNGUwODldXQAAAAAHBQAAAAABAAAAGwAAAAPzAVN5c3RlbS5Db2xsZWN0aW9ucy5HZW5lcmljLktleVZhbHVlUGFpcmAyW1tTeXN0ZW0uU3RyaW5nLCBtc2NvcmxpYiwgVmVyc2lvbj00LjAuMC4wLCBDdWx0dXJlPW5ldXRyYWwsIFB1YmxpY0tleVRva2VuPWI3N2E1YzU2MTkzNGUwODldLFtTdGF0c0RpcmVjdC5UZW1wbGF0ZXMuRmlsbGVkUGFyYW1ldGVyLCBTdGF0c0RpcmVjdCwgVmVyc2lvbj0zLjMuNS4wLCBDdWx0dXJlPW5ldXRyYWwsIFB1YmxpY0tleVRva2VuPW51bGxdXQT6////8wFTeXN0ZW0uQ29sbGVjdGlvbnMuR2VuZXJpYy5LZXlWYWx1ZVBhaXJgMltbU3lzdGVtLlN0cmluZywgbXNjb3JsaWIsIFZlcnNpb249NC4wLjAuMCwgQ3VsdHVyZT1uZXV0cmFsLCBQdWJsaWNLZXlUb2tlbj1iNzdhNWM1NjE5MzRlMDg5XSxbU3RhdHNEaXJlY3QuVGVtcGxhdGVzLkZpbGxlZFBhcmFtZXRlciwgU3RhdHNEaXJlY3QsIFZlcnNpb249My4zLjUuMCwgQ3VsdHVyZT1uZXV0cmFsLCBQdWJsaWNLZXlUb2tlbj1udWxsXV0CAAAAA2tleQV2YWx1ZQEELlN0YXRzRGlyZWN0LlRlbXBsYXRlcy5GaWxsZWREYXRhRnJhbWVQYXJhbWV0ZXICAAAABgcAAAAEZGF0YQkIAAAAAff////6////BgoAAAATcmVwb3J0LWNlbnRpbGUtdHlwZQkLAAAAAfT////6////Bg0AAAALcmVwb3J0LXVkY2EJDgAAAAHx////+v///wYQAAAAC3JlcG9ydC11ZGNiCREAAAAB7v////r///8GEwAAAA9vdXRwdXQtdG8tZnJhbWUJFAAAAAHr////+v///wYWAAAABWdhbW1hCRcAAAAB6P////r///8GGQAAABFyZXBvcnQtdmFsaWQtZGF0YQkaAAAAAeX////6////BhwAAAATcmVwb3J0LW1pc3NpbmctZGF0YQkdAAAAAeL////6////Bh8AAAAKcmVwb3J0LXN1bQkgAAAAAd/////6////BiIAAAALcmVwb3J0LW1lYW4JIwAAAAHc////+v///wYlAAAAD3JlcG9ydC12YXJpYW5jZQkmAAAAAdn////6////BigAAAAJcmVwb3J0LXNkCSkAAAAB1v////r///8GKwAAABVyZXBvcnQtdmFyaWFuY2UtY29lZmYJLAAAAAHT////+v///wYuAAAACnJlcG9ydC1zZW0JLwAAAAHQ////+v///wYxAAAADHJlcG9ydC11OTVjbAkyAAAAAc3////6////BjQAAAAMcmVwb3J0LWw5NWNsCTUAAAAByv////r///8GNwAAABVyZXBvcnQtZ2VvbWV0cmljLW1lYW4JOAAAAAHH////+v///wY6AAAAD3JlcG9ydC1za2V3bmVzcwk7AAAAAcT////6////Bj0AAAAPcmVwb3J0LWt1cnRvc2lzCT4AAAABwf////r///8GQAAAAA5yZXBvcnQtbWF4aW11bQlBAAAAAb7////6////BkMAAAAJcmVwb3J0LXVxCUQAAAABu/////r///8GRgAAAA1yZXBvcnQtbWVkaWFuCUcAAAABuP////r///8GSQAAAAlyZXBvcnQtbHEJSgAAAAG1////+v///wZMAAAACnJlcG9ydC1pcXIJTQAAAAGy////+v///wZPAAAADnJlcG9ydC1taW5pbXVtCVAAAAABr/////r///8GUgAAAAxyZXBvcnQtcmFuZ2UJUwAAAAGs////+v///wZVAAAACnJlcG9ydC11ZGMJVgAAAAUIAAAALlN0YXRzRGlyZWN0LlRlbXBsYXRlcy5GaWxsZWREYXRhRnJhbWVQYXJhbWV0ZXICAAAAFTxEYXRhPmtfX0JhY2tpbmdGaWVsZCpGaWxsZWRQYXJhbWV0ZXIrPERpcmVjdGlvbj5rX19CYWNraW5nRmllbGQEBBpTdGF0c0RpcmVjdC5EYXRhLkRhdGFGcmFtZQIAAAAuU3RhdHNEaXJlY3QuVGVtcGxhdGVzLkZpbGxlZFBhcmFtZXRlckRpcmVjdGlvbgIAAAACAAAACVcAAAAFqP///y5TdGF0c0RpcmVjdC5UZW1wbGF0ZXMuRmlsbGVkUGFyYW1ldGVyRGlyZWN0aW9uAQAAAAd2YWx1ZV9fAAgCAAAAAQAAAAULAAAAK1N0YXRzRGlyZWN0LlRlbXBsYXRlcy5GaWxsZWRTdHJpbmdQYXJhbWV0ZXICAAAAFTxEYXRhPmtfX0JhY2tpbmdGaWVsZCpGaWxsZWRQYXJhbWV0ZXIrPERpcmVjdGlvbj5rX19CYWNraW5nRmllbGQBBC5TdGF0c0RpcmVjdC5UZW1wbGF0ZXMuRmlsbGVkUGFyYW1ldGVyRGlyZWN0aW9uAgAAAAIAAAAGWQAAAAExAab///+o////AQAAAAUOAAAAK1N0YXRzRGlyZWN0LlRlbXBsYXRlcy5GaWxsZWREb3VibGVQYXJhbWV0ZXICAAAAFTxEYXRhPmtfX0JhY2tpbmdGaWVsZCpGaWxsZWRQYXJhbWV0ZXIrPERpcmVjdGlvbj5rX19CYWNraW5nRmllbGQABAYuU3RhdHNEaXJlY3QuVGVtcGxhdGVzLkZpbGxlZFBhcmFtZXRlckRpcmVjdGlvbgIAAAACAAAAmpmZmZmZqT8Bpf///6j///8BAAAAAREAAAAOAAAAZmZmZmZm7j8BpP///6j///8BAAAABRQAAAAsU3RhdHNEaXJlY3QuVGVtcGxhdGVzLkZpbGxlZEJvb2xlYW5QYXJhbWV0ZXICAAAAFTxEYXRhPmtfX0JhY2tpbmdGaWVsZCpGaWxsZWRQYXJhbWV0ZXIrPERpcmVjdGlvbj5rX19CYWNraW5nRmllbGQABAEuU3RhdHNEaXJlY3QuVGVtcGxhdGVzLkZpbGxlZFBhcmFtZXRlckRpcmVjdGlvbgIAAAACAAAAAAGj////qP///wEAAAABFwAAAA4AAABmZmZmZmbuPwGi////qP///wEAAAABGgAAABQAAAABAaH///+o////AQAAAAEdAAAAFAAAAAEBoP///6j///8BAAAAASAAAAAUAAAAAQGf////qP///wEAAAABIwAAABQAAAABAZ7///+o////AQAAAAEmAAAAFAAAAAEBnf///6j///8BAAAAASkAAAAUAAAAAQGc////qP///wEAAAABLAAAABQAAAABAZv///+o////AQAAAAEvAAAAFAAAAAEBmv///6j///8BAAAAATIAAAAUAAAAAQGZ////qP///wEAAAABNQAAABQAAAABAZj///+o////AQAAAAE4AAAAFAAAAAEBl////6j///8BAAAAATsAAAAUAAAAAQGW////qP///wEAAAABPgAAABQAAAABAZX///+o////AQAAAAFBAAAAFAAAAAEBlP///6j///8BAAAAAUQAAAAUAAAAAQGT////qP///wEAAAABRwAAABQAAAABAZL///+o////AQAAAAFKAAAAFAAAAAEBkf///6j///8BAAAAAU0AAAAUAAAAAQGQ////qP///wEAAAABUAAAABQAAAABAY////+o////AQAAAAFTAAAAFAAAAAEBjv///6j///8BAAAAAVYAAAAUAAAAAQGN////qP///wEAAAAFVwAAABpTdGF0c0RpcmVjdC5EYXRhLkRhdGFGcmFtZQIAAAAVPE5hbWU+a19fQmFja2luZ0ZpZWxkGjxWYXJpYWJsZXM+a19fQmFja2luZ0ZpZWxkAQODAVN5c3RlbS5Db2xsZWN0aW9ucy5HZW5lcmljLkxpc3RgMVtbU3RhdHNEaXJlY3QuRGF0YS5JVmFyaWFibGUsIFN0YXRzRGlyZWN0LCBWZXJzaW9uPTMuMy41LjAsIEN1bHR1cmU9bmV1dHJhbCwgUHVibGljS2V5VG9rZW49bnVsbF1dAgAAAAZ0AAAABkRhdGEgMQl1AAAABHUAAACDAVN5c3RlbS5Db2xsZWN0aW9ucy5HZW5lcmljLkxpc3RgMVtbU3RhdHNEaXJlY3QuRGF0YS5JVmFyaWFibGUsIFN0YXRzRGlyZWN0LCBWZXJzaW9uPTMuMy41LjAsIEN1bHR1cmU9bmV1dHJhbCwgUHVibGljS2V5VG9rZW49bnVsbF1dAwAAAAZfaXRlbXMFX3NpemUIX3ZlcnNpb24EAAAcU3RhdHNEaXJlY3QuRGF0YS5JVmFyaWFibGVbXQIAAAAICAl2AAAAAQAAAAEAAAAHdgAAAAABAAAABAAAAAQaU3RhdHNEaXJlY3QuRGF0YS5JVmFyaWFibGUCAAAACXcAAAANAwV3AAAAH1N0YXRzRGlyZWN0LkRhdGEuRG91YmxlVmFyaWFibGUHAAAAA3N1bQNtaW4DbWF4DGhhc1N1bW1hcmllcxZHZW5lcmljVmFyaWFibGVgMStkYXRhKEdlbmVyaWNWYXJpYWJsZWAxKzxUaXRsZT5rX19CYWNraW5nRmllbGQpR2VuZXJpY1ZhcmlhYmxlYDErPE9yaWdpbj5rX19CYWNraW5nRmllbGQAAAAABwEEBgYGAQYgU3RhdHNEaXJlY3QuRGF0YS5Xb3Jrc2hlZXRPcmlnaW4CAAAAAgAAAAAAAAAAAAAAAAAAAAAAAAAAAAAAAAAAAAAKBngAAAAKRGlmZiBTY29yZQl5AAAABXkAAAAgU3RhdHNEaXJlY3QuRGF0YS5Xb3Jrc2hlZXRPcmlnaW4JAAAAFzxDb2x1bW4+a19fQmFja2luZ0ZpZWxkFTxNb2RlPmtfX0JhY2tpbmdGaWVsZBc8VG9wUm93PmtfX0JhY2tpbmdGaWVsZBU8Um93cz5rX19CYWNraW5nRmllbGQdPFdvcmtib29rUGF0aD5rX19CYWNraW5nRmllbGQePFdvcmtzaGVldE5hbWU+a19fQmFja2luZ0ZpZWxkGTxIYXNUaXRsZT5rX19CYWNraW5nRmllbGQcPFdhc0ZpbHRlcmVkPmtfX0JhY2tpbmdGaWVsZBw8T3JpZ2luR3JvdXA+a19fQmFja2luZ0ZpZWxkAAQAAAEBAAAACClTdGF0c0RpcmVjdC5VdGlsaXRpZXMuRGF0YUFjcXVpc2l0aW9uTW9kZQIAAAAICAEBCAIAAAA+AAAABYb///8pU3RhdHNEaXJlY3QuVXRpbGl0aWVzLkRhdGFBY3F1aXNpdGlvbk1vZGUBAAAAB3ZhbHVlX18ACAIAAAACAAAAAAAAAIEAAAAGewAAACxDOlxVc2Vyc1xoYW5zXEFwcERhdGFcTG9jYWxcVGVtcFx+RXhjZWwueGxzeAZ8AAAADU1hdGNoZWQgUGFpcnMAAAAAAAAL <-!redo!!

**Descriptive statistics**

| Variables | Diff Score |
| --- | --- |
| Valid data | 128 |
| Missing data | 0 |
| Sum | -0,95403 |
| Mean | -0,007453 |
| Variance | 0,000128 |
| Standard deviation | 0,011318 |
| Variance coefficient | -1,518555 |
| Standard error of mean | 0,001 |
| Upper 95% CL of mean | -0,005474 |
| Lower 95% CL of mean | -0,009433 |
| Geometric mean | * |
| Skewness | -0,48087 |
| Kurtosis | 3,755782 |
| Maximum | 0,015865 |
| Upper quartile | 0 |
| Median | -0,005963 |
| Lower quartile | -0,014827 |
| Interquartile range | 0,014827 |
| Minimum | -0,04472 |
| Range | 0,060585 |
| Centile 95 | 0,012456 |
| Centile 5 | -0,022871 |
